# Supplementary material for: Guanidines: Synthesis of Novel Histamine H3R Antagonists with Additional Breast Anticancer Activity and Cholinesterases Inhibitory Effect
Source: Pharmaceuticals (Basel). 2023 Apr 30;16(5):675. doi: 10.3390/ph16050675 (PMC10223552; doi:10.3390/ph16050675)
Supplement: Supplementary file 1 [file pharmaceuticals-16-00675-s001.zip › pharmaceuticals-2366364-supplementary.pdf]

Supplementary materials

Guanidines: Synthesis of Novel Histamine H_3R Antagonists with Additional Breast Anticancer Activity and Cholinesterases Inhibitory Effect

Marek Staszewski*[a], Magdalena Iwan[b], Tobias Werner[c], Marek Bajda[d], Justyna Godyń[d], Gniewomir Latacz[e], Agnieszka Korga-Plewko[f], Joanna Kubik[f], Natalia Szałaj[d], Holger Stark[c], Barbara Malawska[d], Anna Więckowska[d], Krzysztof Walczyński[a]

[a] Department of Synthesis and Technology of Drugs, Medical University of Lodz, Muszyńskiego 1, 90-151 Łódź, Poland
[b] Department of Toxicology, Medical University of Lublin, Chodźki 8, 20-093 Lublin, Poland
[c] Institute of Pharmaceutical and Medicinal Chemistry, Heinrich Heine University Düsseldorf, Universitaetsstr. 1, 40225 Duesseldorf, Germany
[d] Department of Physicochemical Drug Analysis, Jagiellonian University Medical College, Medyczna 9, 30–688 Kraków, Poland
[e] Department of Technology and Biotechnology of Drugs, Jagiellonian University Medical College, Medyczna 9, 30-688 Kraków, Poland
[f] Independent Medical Biology Unit, Medical University of Lublin, Jaczewskiego 8b, 20-090 Lublin, Poland

Table of contents

Entry	Section	Page
1	**Chemical synthesis and data analysis.**	**S2**
2	**NMR spectra.**	**S36**
3	**Pharmacological assay results.**	**S62**
3.1	*Ex vivo* assay for histamine H_3R receptor antagonists on guinea pig ileum.	**S62**
3.2	*Ex vivo* assay for histamine H_1R receptor antagonists on guinea pig ileum.	**S70**
3.3	*h*H_3R radioligand displacement binding assay.	**S79**
3.4	Cell viability.	**S88**
3.5	Inhibition of electric eel AChE and equine serum BuChE	**S90**
4	***In vitro* metabolic stability.**	**S93**

1. Chemical synthesis and data analysis

Preparation of 1-[(7-bromoheptyl)oxy]-4-chlorobenzene (**1**)

4-Chlorophenol (0.75 g; 5.83·10-3 mol) was added to the freshly-prepared sodium ethoxide (0.134 g; 5.83·10-3 mol) solution in anhydrous ethanol (15 mL) and stirred for 30 minutes at room temperature. Then, the previously-prepared sodium 4-chlorophenoxide solution was added dropwise to a solution of 1,7-dibromoheptane (1.5 g; $5.81 \cdot 10^{-3}$ mol) in 30 mL anhydrous ethanol heated to 65 °C. The reaction was stirred overnight at 80 °C. The solvent was removed under vacuum and the residue was diluted by 30 mL water, and extracted 3x30 mL with DCM. The combined organic phases were dried over anhydrous Na_2SO_4. The solvent was removed under vacuum and the crude product was purified by column chromatography (hexane/DCM 17:3) to yield the pure product.

1-[(7-bromoheptyl)oxy]-4-chlorobenzene (**1**): $C_{13}H_{18}BrClO$. M=305.64. Colourless solid. 47.17 % yield. R_f=0.49 (hexane/DCM 17:3). mp: 47.5-48.5 °C. ^1H NMR (600 MHz, *CDCl₃*) δ ppm 7.23-7.21 (m, $2H^{phenoxy.}$, $C(CHC\underline{H})_2CCl$), 6.83-6.79 (m, $2H^{phenoxy.}$, $C(C\underline{H}CH)_2CCl$), 3.92 (t, 2H, $OC\underline{H_2}$; *J*=6.48Hz), 3.42 (t, 2H, $BrC\underline{H_2}$; *J*=6.81Hz), 1.89-1.85 (m, 2H, $C\underline{H_2}CH_2Br$), 1.79-1.75 (m, 2H, $OCH_2C\underline{H_2}$), 1.49-1.45 (m, 4H, $CH_2C\underline{H_2}CH_2$), 1.42-1.36 (m, 2H, $CH_2C\underline{H_2}CH_2$). ^{13}C NMR (150.95 MHz, *CDCl₃*) δ ppm 157.66 ($1C^{quat./phenoxy.}$, $\underline{C}O$), 129.25 ($2C^{phenoxy.}$, $C(CH\underline{C}H)_2CCl$), 125.31 ($1C^{quat./phenoxy.}$, $C(CHCH)_2\underline{C}Cl$), 115.71 ($2C^{phenoxy.}$, $C(\underline{C}HCH)_2CCl$), 68.13 (1C, $O\underline{C}H_2$), 33.93 (1C, $\underline{C}H_2Br$), 32.68 (1C, $\underline{C}H_2CH_2Br$), 29.07 (1C, $OCH_2\underline{C}H_2$), 28.49 (1C, $CH_2\underline{C}H_2CH_2$), 28.06 (1C, $CH_2\underline{C}H_2CH_2$), 25.86 (1C, $CH_2\underline{C}H_2CH_2$).

Preparation of 1-[7-(4-chlorophenoxy)heptyl]piperazine (**2**)

1-[(7-bromoheptyl)oxy]-4-chlorobenzene (**1**) (0.737 g; $2.41 \cdot 10^{-3}$ mol) in 20 mL methanol heated to 40 °C was added dropwise to a solution of piperazine (1.04 g; $1.21 \cdot 10^{-2}$ mol) in 70 mL methanol heated to 60 °C. The reaction was stirred overnight at 70 °C. The solvent was removed under vacuum and the residue was diluted with 20 mL DCM. The precipitate was discarded. The solvent was removed under vacuum and the crude product was purified by column chromatography (DCM/MeOH/25% NH_3aq. 89:10:1) to yield the pure product.

1-[7-(4-chlorophenoxy)heptyl]piperazine (**2**): $C_{17}H_{27}ClN_2O$. M=310.86. Colourless sticky oil. 86.65 % yield. R_f=0.53 (DCM/MeOH/25% NH_3aq. 89:10:1). ^1H NMR (600 MHz, *CDCl₃*) δ ppm 7.22-7.21 (m, $2H^{phenoxy.}$, $C(CHC\underline{H})_2CCl$), 6.82-6.80 (m, $2H^{phenoxy.}$, $C(C\underline{H}CH)_2CCl$), 3.91 (t, 2H, $OC\underline{H_2}$; *J*=6.53Hz), 2.91-2.90 (m, $4H^{piperazine}$), 2.41 (br, $4H^{piperazine}$), 2.31 (t, 2H, $C\underline{H_2}N^{piperazine}$, *J* = 7.80Hz), 1.78-1.74 (m, 2H, $OCH_2C\underline{H_2}$), 1.69 (br. 1H, $N\underline{H}$*), 1.53-1.48 (m, 2H, $CH_2C\underline{H_2}CH_2$), 1.46-1.42 (m, 2H, $CH_2C\underline{H_2}CH_2$), 1.39-1.31 (m, 4H, $CH_2C\underline{H_2}CH_2$). ^{13}C NMR (150.95 MHz, *CDCl₃*) δ ppm 157.69 ($1C^{quat./phenoxy.}$, $\underline{C}O$), 129.24 ($2C^{phenoxy.}$, $C(CH\underline{C}H)_2CCl$), 125.27 ($1C^{quat./phenoxy.}$, $C(CHCH)_2\underline{C}Cl$), 115.72 ($2C^{phenoxy.}$, $C(\underline{C}HCH)_2CCl$), 68.24 (1C, $O\underline{C}H_2$), 59.43 (1C, $\underline{C}H_2N^{piperazine}$), 54.67 ($2C^{piperazine}$), 46.13 ($2C^{piperazine}$), 29.30 (1C, $CH_2\underline{C}H_2CH_2$), 29.13 (1C, $CH_2\underline{C}H_2CH_2$), 27.53 (1C, $CH_2\underline{C}H_2CH_2$), 26.61 (1C, $CH_2\underline{C}H_2CH_2$), 25.94 (1C, $CH_2\underline{C}H_2CH_2$).

Preparation of 4-{4-[7-(4-chlorophenoxy)heptyl]piperazin-1-yl}butanenitrile (**3**)

Potassium carbonate (1.39 g; $1.00 \cdot 10^{-2}$ mol) and 4-bromobutyronitrile (0.40 g; $2.70 \cdot 10^{-3}$ mol) was added to a solution of 1-[7-(4-chlorophenoxy)heptyl]piperazine (**2**) (0.626 g; $2.01 \cdot 10^{-3}$ mol) in 20 mL acetonitrile. The reaction was stirred overnight at 80 °C, then filtered. The precipitate was discarded. The solvent was removed under vacuum and the crude product was purified by column chromatography (EtOAc/MeOH/Triethylamine 89:10:1) to yield the pure product.

4-{4-[7-(4-chlorophenoxy)heptyl]piperazin-1-yl}butanenitrile (**3**): $C_{21}H_{32}ClN_3O$. M=377.95. Colourless sticky oil. 85.40 % yield. R_f=0.37 (EtOAc/MeOH/Triethylamine 89:10:1). ^1H NMR (600 MHz, *$CDCl_3$*) δ ppm 7.22-7.21 (m, $2H^{phenoxy.}$, $C(CHC\underline{H})_2CCl$), 6.82-6.80 (m, $2H^{phenoxy.}$, $C(C\underline{H}CH)_2CCl$), 3.91 (t, 2H, $OC\underline{H}_2$; *J*=6.52Hz), 2.53-2.41 (m, 12H, $8H^{piperazine}$, $N^{piperazine}C\underline{H}_2CH_2C\underline{H}_2CN$), 2.32 (t, 2H, $O(CH_2)_6C\underline{H}_2N^{piperazine}$, *J* = 7.80Hz), 1.82 (qt, 2H, $C\underline{H}_2CH_2CN$), 1.76 (qt, 2H, $OCH_2C\underline{H}_2$), 1.54-1.48 (m, 2H, $CH_2C\underline{H}_2CH_2$), 1.48-1.42 (m, 2H, $OCH_2CH_2C\underline{H}_2$), 1.39-1.29 (m, 4H, $CH_2C\underline{H}_2CH_2$). ^{13}C NMR (150.95 MHz, *$CDCl_3$*) δ ppm 157.70 ($1C^{quat./phenoxy.}$, $\underline{C}O$), 129.24 ($2C^{phenoxy.}$, $C(CH\underline{C}H)_2CCl$), 125.27 ($1C^{quat./phenoxy.}$, $C(CHCH)_2\underline{C}Cl$), 119.79 ($1C^{quat.}$, $\underline{C}{\equiv}N$), 115.73 ($2C^{phenoxy.}$, $C(\underline{C}HCH)_2CCl$), 68.23 (1C, $O\underline{C}H_2$), 58.75 (1C, $O(CH_2)_6\underline{C}H_2N^{piperazine}$), 56.32 (1C, $N^{piperazine}\underline{C}H_2CH_2CH_2CN$), 53.23 ($2C^{piperazine}$), 53.08 ($2C^{piperazine}$), 29.29 (1C, $CH_2\underline{C}H_2CH_2$), 29.13 (1C, $OCH_2\underline{C}H_2$), 27.51 (1C, $CH_2\underline{C}H_2CH_2$), 26.84 (1C, $CH_2\underline{C}H_2CH_2$), 25.94 (1C, $CH_2\underline{C}H_2CH_2$), 22.75 (1C, $\underline{C}H_2CH_2CN$), 14.93 (1C, $\underline{C}H_2CN$).

Preparation of 4-{4-[7-(4-chlorophenoxy)heptyl]piperazin-1-yl}butan-1-amine (**4**)

$LiAlH_4$ (0.26 g; $6.85 \cdot 10^{-3}$ mol) was added to a solution of 4-{4-[7-(4-chlorophenoxy)heptyl]piperazin-1-yl}butanenitrile (**3**) (0.65 g; $1.72 \cdot 10^{-3}$ mol) in 30 mL anhydrous diethyl ether. The reaction was stirred overnight at room temperature, then the mixture was quenched by dropwise addition of water (16 equiv.) and 10 % NaOH solution (16 equiv.) stirred for two hours, then filtered. The precipitate was discarded. The organic layer was dried over Na_2SO_4, then the solvent was removed under vacuum and the crude product was purified by column chromatography (DCM/MeOH/25% NH_3 aq. 39:10:1) to yield the pure product.

4-{4-[7-(4-chlorophenoxy)heptyl]piperazin-1-yl}butan-1-amine (**4**): $C_{21}H_{36}ClN_3O$. M=381.98. Beige solid. 84.63 % yield. R_f=0.47 (DCM/MeOH/25% NH_3 aq. 39:10:1). mp: 74.6-76.3 °C. ^1H NMR (600 MHz, *$CDCl_3$*) δ ppm 7.22-7.21 (m, $2H^{phenoxy.}$, $C(CHC\underline{H})_2CCl$), 6.81-6.80 (m, $2H^{phenoxy.}$, $C(C\underline{H}CH)_2CCl$), 3.91 (t, 2H, $OC\underline{H}_2$; *J*=6.53Hz), 2.71 (t, 2H, $C\underline{H}_2NH_2$, *J* = 6.88Hz), 2.48 (br, $8H^{piperazine}$), 2.36-2.31 (m, 4H, $C\underline{H}_2N^{piperazine}$), 1.76 (qt, 2H, $OCH_2C\underline{H}_2$), 1.57 (br, 2H, *, $N\underline{H}_2$), 1.55-1.42 (m, 8H, $CH_2C\underline{H}_2CH_2$), 1.39-1.29 (m, 4H, $CH_2C\underline{H}_2CH_2$). ^{13}C NMR (150.95 MHz, *$CDCl_3$*) δ ppm 157.68 ($1C^{quat./phenoxy.}$, $\underline{C}O$), 129.21 ($2C^{phenoxy.}$, $C(CH\underline{C}H)_2CCl$), 125.24 ($1C^{quat./phenoxy.}$, $C(CHCH)_2\underline{C}Cl$), 115.71 ($2C^{phenoxy.}$, $C(\underline{C}HCH)_2CCl$), 68.21 (1C, $O\underline{C}H_2$), 58.79 (1C, $\underline{C}H_2N^{piperazine}$), 58.54 (1C, $\underline{C}H_2N^{piperazine}$), 53.27 ($2C^{piperazine}$), 53.25 ($2C^{piperazine}$), 42.11 (1C, $\underline{C}H_2NH_2$), 31.78 (1C, $CH_2\underline{C}H_2CH_2$), 29.27 (1C, $CH_2\underline{C}H_2CH_2$), 29.10 (1C, $OCH_2\underline{C}H_2$), 27.51 (1C, $CH_2\underline{C}H_2CH_2$), 26.84 (1C, $CH_2\underline{C}H_2CH_2$), 25.91 (1C, $CH_2\underline{C}H_2CH_2$), 24.32 (1C, $CH_2\underline{C}H_2CH_2$).

Preparation of *N*-{4-{4-[7-(4-chlorophenoxy)heptyl]piperazin-1-yl}butyl}benzamide (**5a**)

Benzoyl chloride (0.117 g; $8.32\cdot10^{-4}$ mol) in 10 mL DCM was added dropwise to a solution of 4-{4-[7-(4-chlorophenoxy)heptyl]piperazin-1-yl}butan-1-amine (**4**) (0.276 g; $7.23\cdot10^{-4}$ mol) and triethylamine (0.30 g; $2.96\cdot10^{-3}$ mol) in 15 mL DCM. The reaction was stirred for three hours at room temperature. The mixture was washed three-times with 15 mL water and dried over Na_2SO_4. The solvent was removed under vacuum and the crude product was purified by column chromatography (EtOAc/MeOH/Triethylamine 89:10:1) to yield the pure product.

N-{4-{4-[7-(4-chlorophenoxy)heptyl]piperazin-1-yl}butyl}benzamide (**5a**): $C_{28}H_{40}ClN_3O_2$. M=486.09. Yellowish solid. 92.86 % yield. R_f=0.22 (EtOAc/MeOH/Triethylamine 89:10:1). mp: 104.8-106.8 °C. ^{1}H NMR (600 MHz, *CDCl₃*) δ ppm 7.76-7.75 (m, 2$H^{arom.}$, $C(C\underline{H}CH)_2CH$), 7.50-7.47 (m, 1$H^{arom.}$, $C(CHCH)_2C\underline{H}$), 7.43-7.41 (m, 2$H^{arom.}$, $C(CHC\underline{H})_2CH$), 7.22-7.21 (m, 2$H^{phenoxy.}$, $C(CHC\underline{H})_2CCl$), 6.82-6.80 (m, 2$H^{phenoxy.}$, $C(C\underline{H}CH)_2CCl$), 6.69 (br, 1H, $N\underline{H}$), 3.91 (t, 2H, $OC\underline{H}_2$; *J*=6.52Hz), 3.48 (dt, 2H, $C\underline{H}_2NH$), 2.47 (br, 8$H^{piperazine}$), 2.41-2.38 (m, 2H, $C\underline{H}_2N^{piperazine}$; *J*=7.00 Hz), 2.31-2.28 (m, 2H, $C\underline{H}_2N^{piperazine}$; *J*=7.80 Hz), 1.77 (qt, 2H, $OCH_2C\underline{H}_2$), 1.68-1.61 (m, 4H, $CH_2C\underline{H}_2CH_2$), 1.54-1.42 (m, 4H, $CH_2C\underline{H}_2CH_2$), 1.38-1.31 (m, 4H, $CH_2C\underline{H}_2CH_2$). ^{13}C NMR (150.95 MHz, *CDCl₃*) δ ppm 167.70 (1$C^{quat.}$, C=O), 157.71 (1$C^{quat./phenoxy.}$, $\underline{C}O$), 135.04 (1$C^{quat./arom.}$), 131.24 (1$C^{arom.}$, $C(CHCH)_2\underline{C}H$), 129.25 (2$C^{phenoxy.}$, $C(CH\underline{C}H)_2CCl$), 128.50 (2$C^{arom.}$, $C(CH\underline{C}H)_2CH$), 126.94 (2$C^{arom.}$, $C(\underline{C}HCH)_2CH$), 125.29 (1$C^{quat./phenoxy.}$, $C(CHCH)_2\underline{C}Cl$), 115.74 (2$C^{phenoxy.}$, $C(\underline{C}HCH)_2CCl$), 68.24 (1C, $O\underline{C}H_2$), 58.71 (1C, $\underline{C}H_2N^{piperazine}$), 57.99 (1C, $\underline{C}H_2N^{piperazine}$), 53.19 (2$C^{piperazine}$), 53.07 (2$C^{piperazine}$), 39.45 (1C, $\underline{C}H_2NH$), 29.28 (1C, $CH_2\underline{C}H_2CH_2$), 29.13 (1C, $OCH_2\underline{C}H_2$), 27.50 (1C, $CH_2\underline{C}H_2CH_2$), 27.46 (1C, $CH_2\underline{C}H_2CH_2$), 26.79 (1C, $CH_2\underline{C}H_2CH_2$), 25.94 (1C, $CH_2\underline{C}H_2CH_2$), 24.48 (1C, $CH_2\underline{C}H_2CH_2$).

Preparation of *N*-{4-{4-[7-(4-chlorophenoxy)heptyl]piperazin-1-yl}butyl}-4-(trifluoromethyl)benzamide (**5b**)

4-(Trifluoromethyl)benzoyl chloride (0.157 g; $7.53\cdot10^{-4}$ mol) in 10 mL DCM was added dropwise to a solution of 4-{4-[7-(4-chlorophenoxy)heptyl]piperazin-1-yl}butan-1-amine (**4**) (0.25 g; $6.54\cdot10^{-4}$ mol) and triethylamine (0.25 g; $2.47\cdot10^{-3}$ mol) in 15 mL DCM. The reaction was stirred for three hours at room temperature. The mixture was washed three-times with 15 mL water and dried over Na_2SO_4. The solvent was removed under vacuum and the crude product was purified by column chromatography (EtOAc/MeOH/Triethylamine 39:10:1) to yield the pure product.

N-{4-{4-[7-(4-chlorophenoxy)heptyl]piperazin-1-yl}butyl}-4-(trifluoromethyl)benzamide (**5b**): $C_{29}H_{39}ClF_3N_3O_2$. M=554.09. Yellowish solid. 97.24 % yield. R_f=0.42 (EtOAc/MeOH/Triethylamine 39:10:1). mp: 113.0-115.0 °C ^{1}H NMR (600 MHz, *CDCl₃*) δ ppm 7.86-7.85 (m, 2$H^{arom.}$, $(C\underline{H}CH)_2CCF_3$), 7.69-7.67 (m, 2$H^{arom.}$, $(CHC\underline{H})_2CCF_3$), 7.22-7.20 (m, 2$H^{phenoxy.}$, $C(CHC\underline{H})_2CCl$), 6.99 (br, 1H, $N\underline{H}$), 6.81-6.80 (m, 2$H^{phenoxy.}$, $C(C\underline{H}CH)_2CCl$), 3.91 (t, 2H, $OC\underline{H}_2$, *J*=6.50Hz), 3.48 (dt, 2H, $C\underline{H}_2NH$), 2.46-2.68 (m, 10H: 8$H^{piperazine}$, $C\underline{H}_2N^{piperazine}$), 2.26 (t, 2H, $C\underline{H}_2N^{piperazine}$, *J*=7.50Hz), 1.76 (qt, 2H, $CH_2C\underline{H}_2CH_2$), 1.69 (qt, 2H, $CH_2C\underline{H}_2CH_2$), 1.63 (qt, 2H, $CH_2C\underline{H}_2CH_2$), 1.48-1.42 (m, 4H, $CH_2C\underline{H}_2CH_2$), 1.38-1.29 (qt, 4H, $CH_2C\underline{H}_2CH_2$). ^{13}C NMR (150.95 MHz, *CDCl₃*) δ ppm 166.51 (1$C^{quat.}$, C=O), 157.77 (1$C^{quat./phenoxy.}$, $\underline{C}O$), 138.57 (1$C^{quat./arom.}$, $\underline{C}C(O)$), 133.33, 133.12, 132.89, 132.68 (1$C^{quat./arom.}$, $\underline{C}CF_3$), 129.25 (2$C^{phenoxy.}$, $C(CH\underline{C}H)_2CCl$), 127.49 (2$C^{arom.}$, $(\underline{C}HCH)_2CCF_3$), 125.34 (1$C^{quat./phenoxy.}$, $C(CHCH)_2\underline{C}Cl$), 126.43, 124.63, 122.82, 121.01 (1C, $\underline{C}F_3$), 115.81 (2$C^{phenoxy.}$, $C(\underline{C}HCH)_2CCl$), 125.56,125.54 (2$C^{arom.}$, $(\underline{C}H)_2CCF_3$), 68.30 (1C, $O\underline{C}H_2$), 58.69 (1C, $\underline{C}H_2N^{piperazine}$), 57.98 (1C, $\underline{C}H_2N^{piperazine}$), 53.25 (2$C^{piperazine}$), 53.06 (2$C^{piperazine}$), 40.16 (1C,

$\underline{C}H_2NH$), 29.26 (1C, $CH_2\underline{C}H_2CH_2$), 29.14 (1C, $CH_2\underline{C}H_2CH_2$), 27.48 (1C, $CH_2\underline{C}H_2CH_2$), 27.41 (1C, $CH_2\underline{C}H_2CH_2$), 26.79 (1C, $CH_2\underline{C}H_2CH_2$), 25.94 (1C, $CH_2\underline{C}H_2CH_2$), 24.53 (1C, $CH_2\underline{C}H_2CH_2$).

Preparation of *N*-benzyl-4-{4-[7-(4-chlorophenoxy)heptyl]piperazin-1-yl}butan-1-amine (**6a**)

$LiAlH_4$ (0.109 g; $2.87 \cdot 10^{-3}$ mol) was added to a solution of *N*-{4-{4-[7-(4-chlorophenoxy)heptyl]piperazin-1-yl}butyl}benzamide (**5a**) (0.337 g; $6.93 \cdot 10^{-4}$ mol) in 40 mL anhydrous diethyl ether. The reaction was stirred overnight at room temperature, then the mixture was quenched by dropwise addition of water (16 equiv.) and 10 % NaOH solution (16 equiv.) stirred for two hours, then filtered. The precipitate was discarded. The organic layer was dried over Na_2SO_4, then the solvent was removed under vacuum and the crude product was purified by column chromatography (DCM/MeOH/25% NH_3 aq. 89:10:1) to yield the pure product.

N-benzyl-4-{4-[7-(4-chlorophenoxy)heptyl]piperazin-1-yl}butan-1-amine (**6a**): $C_{28}H_{42}ClN_3O$. M=472.11. White waxy solid. 81.96 % yield. R_f=0.71 (DCM/MeOH/25% NH_3 aq. 89:10:1). mp: 53.8-55.8 °C. ^1H NMR (600 MHz, *CDCl₃*) δ ppm 7.32-7.31 (m, $4H^{benz.}$, $C(C\underline{H}C\underline{H})_2CH$), 7.25-7.23 (m, $1H^{benz.}$, $C(CHCH)_2C\underline{H}$), 7.22-7.20 (m, $2H^{phenoxy.}$, $C(CHC\underline{H})_2CCl$), 6.81-6.80 (m, $2H^{phenoxy.}$, $C(C\underline{H}CH)_2CCl$), 3.90 (t, 2H, $OC\underline{H_2}$; *J*=6.52Hz), 3.78 (s, 2H, $PhC\underline{H_2}NH$), 2.66-2.29 (m, 14H, $8H^{piperazine}$; $C\underline{H_2}N^{piperazine}$, $NHC\underline{H_2}CH_2$), 2.00 (br. 1H, $N\underline{H}$*), 1.78-1.73 (m, 2H, $OCH_2C\underline{H_2}$), 1.54-1.52 (m, 4H, $CH_2C\underline{H_2}CH_2$), 1.50-1.42 (m, 4H, $CH_2C\underline{H_2}CH_2$), 1.39-1.30 (m, 4H, $CH_2C\underline{H_2}CH_2$). ^{13}C NMR (150.95 MHz, *CDCl₃*) δ ppm 157.67 ($1C^{quat./phenoxy.}$, $\underline{C}O$), 140.29 ($1C^{quat./benz.}$), 129.20 ($2C^{phenoxy.}$, $C(CH\underline{C}H)_2CCl$), 128.35 ($2C^{benz.}$, $C(CH\underline{C}H)_2CH$), 128.09 ($2C^{benz.}$, $C(\underline{C}HCH)_2CH$), 126.88 ($1C^{benz.}$, $C(CHCH)_2\underline{C}H$), 125.23 ($1C^{quat./phenoxy.}$, $C(CHCH)_2\underline{C}Cl$), 115.69 ($2C^{phenoxy.}$, $C(\underline{C}HCH)_2CCl$), 68.20 (1C, $O\underline{C}H_2$), 58.76 (1C, $\underline{C}H_2N^{piperazine}$), 58.54 (1C, $\underline{C}H_2N^{piperazine}$), 53.95 (1C, $Ph\underline{C}H_2NH$), 53.23 ($2C^{piperazine}$), 53.21 ($2C^{piperazine}$), 49.25 (1C, $CH_2\underline{C}H_2NH$), 29.26 (1C, $CH_2\underline{C}H_2CH_2$), 29.09 (1C, $CH_2\underline{C}H_2CH_2$), 28.05 (1C, $CH_2\underline{C}H_2CH_2$), 27.49 (1C, $CH_2\underline{C}H_2CH_2$), 26.81 (1C, $CH_2\underline{C}H_2CH_2$), 25.90 (1C, $CH_2\underline{C}H_2CH_2$), 24.72 (1C, $CH_2\underline{C}H_2CH_2$).

Preparation of 4-{4-[7-(4-chlorophenoxy)heptyl]piperazin-1-yl}-*N*-[4-(trifluoromethyl)benzyl]butan-1-amine (**6b**)

$LiAlH_4$ (0.095 g; $2.50 \cdot 10^{-3}$ mol) was added to a solution of *N*-{4-{4-[7-(4-chlorophenoxy)heptyl]piperazin-1-yl}butyl}-4-(trifluoromethyl)benzamide (**5b**) (0.338 g; $6.10 \cdot 10^{-4}$ mol) in 40 mL anhydrous diethyl ether. The reaction was stirred overnight at room temperature, then the mixture was quenched by dropwise addition of water (16 equiv.) and 10 % NaOH solution (16 equiv.) stirred for two hours, then filtered. The precipitate was discarded. The organic layer was dried over Na_2SO_4, then the solvent was removed under vacuum and the crude product was purified by column chromatography (DCM/MeOH/25% NH_3 aq. 139:10:1 and 89:10:1) to yield the pure product.

4-{4-[7-(4-chlorophenoxy)heptyl]piperazin-1-yl}-*N*-[4-(trifluoromethyl)benzyl]butan-1-amine (**6b**): $C_{29}H_{41}ClF_3N_3O$. M=540.10. Orange waxy solid. 80.85 % yield. R_f=0.79 (DCM/MeOH/25% NH_3 aq. 89:10:1). mp: 43.0-45.0 °C. ^1H NMR (600 MHz, *CDCl₃*) δ ppm 7.58-7.57 (m, $2H^{arom.}$, $C(CHC\underline{H})_2CCF_3$), 7.45-7.44 (m, $2H^{arom.}$, $(C\underline{H}CH)_2CCF_3$), 7.22-7.21 (m, $2H^{phenoxy.}$, $C(CHC\underline{H})_2CCl$), 6.82-6.80 (m, $2H^{phenoxy.}$, $C(C\underline{H}CH)_2CCl$), 3.91 (t, 2H, $OC\underline{H_2}$; *J*=6.52Hz), 3.85 (s, 2H, $NC\underline{H_2}Ph$), 2.65 (t, 2H, $CH_2C\underline{H_2}NH$, *J* = 6.44 Hz), 2.47 (br, 8H, $8H^{piperazine}$), 2.36-

2.30 (m, 4H, $C\underline{H}_2N^{piperazine}$), 1.86 (br, 1H, $N\underline{H}$*), 1.79-1.74 (m, 2H, $OCH_2C\underline{H}_2$), 1.54-1.42 (m, 8H, $CH_2C\underline{H}_2CH_2$), 1.39-1.29 (m, 4H, $CH_2C\underline{H}_2CH_2$). ^{13}C NMR (150.95 MHz, *CDCl₃*) δ ppm 157.69 ($1C^{quat./phenoxy.}$, $\underline{C}O$), 144.47 ($1C^{quat./arom.}$), 129.49, 129.27, 129.06, 128.85 ($1C^{quat./arom.}$, $\underline{C}CF_3$), 129.22 ($2C^{phenoxy.}$, $C(CH\underline{C}H)_2CCl$), 128.26 ($2C^{arom.}$, $(\underline{C}HCH)_2CCF_3$), 126.93, 125.13, 123.33, 121.53 (1C, $\underline{C}F_3$), 125.30 ($1C^{quat./phenoxy.}$, $C(CHCH)_2\underline{C}Cl$), 125.28, 125.25 ($2C^{arom.}$, $C(CH\underline{C}H)_2CCF_3$), 115.72 ($2C^{phenoxy.}$, $C(\underline{C}HCH)_2CCl$), 68.21 (1C, $O\underline{C}H_2$), 58.75 (1C, $CH_2\underline{C}H_2N^{piperazine}$), 58.49 (1C, $CH_2\underline{C}H_2N^{piperazine}$), 53.39 (1C, $Ph\underline{C}H_2NH$), 53.19 ($4C^{piperazine}$), 49.27 (1C, $CH_2\underline{C}H_2NH$), 29.26 (1C, $CH_2\underline{C}H_2CH_2$), 29.10 (1C, $CH_2\underline{C}H_2CH_2$), 28.04 (1C, $CH_2\underline{C}H_2CH_2$), 27.49 (1C, $CH_2\underline{C}H_2CH_2$), 26.79 (1C, $CH_2\underline{C}H_2CH_2$), 25.91 (1C, $CH_2\underline{C}H_2CH_2$), 24.67 (1C, $CH_2\underline{C}H_2CH_2$).

Preparation of 1-benzyl-1-{4-{4-[7-(4-chlorophenoxy)heptyl]piperazin-1-yl}but-1-yl}-2,3-di(*tert*-butoxycarbonyl)guanidine (**7a**)

1,3-bis(*tert*-butoxycarbonyl)-2-methylisothiourea (0.181 g; $6.23\cdot10^{-4}$ mol) and mercury II chloride (0.170 g; $6.26\cdot10^{-4}$ mol) were sequentially added to an ice-cooled mixture of *N*-benzyl-4-{4-[7-(4-chlorophenoxy)heptyl]piperazin-1-yl}butan-1-amine (**6a**) (0.268 g; $5.68\cdot10^{-4}$ mol) and triethylamine (0.29 g; $2.86\cdot10^{-3}$ mol) in 30 mL DCM. The ice bath was removed and the reaction was stirred for eighteen hours at room temperature, then filtered. The precipitate was discarded. The filtrate was washed sequentially twice with 15 mL H_2O and twice with 15 mL brine. The combined organic phases were dried over Na_2SO_4, then the solvent was removed under vacuum and the crude product was purified by column chromatography (EtOAc/MeOH/Triethylamine 89:10:1) to yield the pure product.

1-benzyl-1-{4-{4-[7-(4-chlorophenoxy)heptyl]piperazin-1-yl}but-1-yl}-2,3-di(*tert*-butoxycarbonyl)guanidine (**7a**): $C_{39}H_{60}ClN_5O_5$. M=714.38. Colourless oil. 67.82 % yield. R_f=0.45 (EtOAc/MeOH/Triethylamine 89:10:1). ^1H NMR (600 MHz, *CDCl₃*) δ ppm 9.98 (br, 1H, $N\underline{H}$), 7.33-7.31 (m, $2H^{arom.}$, $C(CHC\underline{H})_2CH$), 7.28-7.24 (m, $3H^{arom.}$, $C(C\underline{H}CH)_2C\underline{H}$), 7.22-7.21 (m, $2H^{phenoxy.}$, $C(CHC\underline{H})_2CCl$), 6.82-6.80 (m, $2H^{phenoxy.}$, $C(C\underline{H}CH)_2CCl$), 4.65 (br, 2H, $NC\underline{H}_2Ph$), 3.91 (t, 2H, $OC\underline{H}_2$; J=6.51Hz), 3.34 (br, 2H, $CH_2C\underline{H}_2NC(N)$), 2.43 (br, 8H, $8H^{piperazine}$), 2.33-2.26 (m, 4H, $C\underline{H}_2N^{piperazine}$), 1.78-1.73 (m, 2H, $OCH_2C\underline{H}_2$), 1.59-1.54 (m, 2H, $CH_2C\underline{H}_2CH_2$), 1.49 (m, 18H, $C\underline{H}_3$), 1.46-1.30 (m, 10H, $CH_2C\underline{H}_2CH_2$). ^{13}C NMR (150.95 MHz, *CDCl₃*) δ ppm 171.13 (1C, $\underline{C}$=O), 162.72 (1C, $\underline{C}$=O), 156.03 ($1C^{quat./phenoxy.}$, $\underline{C}O$), 156.24 ($1C^{quat.}$, $\underline{C}$=N), 136.37 ($1C^{quat./arom.}$, $\underline{C}(CHCH)_2C$), 129.22 ($2C^{phenoxy.}$, $C(CH\underline{C}H)_2CCl$), 128.62 ($2C^{arom.}$, $C(CH\underline{C}H)_2CH$), 127.86 ($2C^{arom.}$, $C(\underline{C}HCH)_2CH$), 127.55 ($1C^{arom.}$, $C(CHCH)_2\underline{C}H$), 125.25 ($1C^{quat./phenoxy.}$, $C(CHCH)_2\underline{C}Cl$), 115.72 ($2C^{phenoxy.}$, $C(\underline{C}HCH)_2CCl$), 81.89 ($1C^{quat.}$ Boc), 79.37 ($1C^{quat.}$ Boc), 68.22 (1C, $O\underline{C}H_2$), 58.77 (1C, $CH_2\underline{C}H_2N^{piperazine}$), 58.02 (1C, $CH_2\underline{C}H_2N^{piperazine}$), 53.24, 53.12 ($4C^{piperazine}$), 50.52 (1C, $N\underline{C}H_2Ph$), 47.26 (1C, $CH_2\underline{C}H_2NC(N)$), 29.28 (1C, $CH_2\underline{C}H_2CH_2$), 29.11 (1C, $CH_2\underline{C}H_2CH_2$), 28.21 (1C, $CH_2CH_2CH_2$), 28.13 (6C, $\underline{C}H_3$), 27.51 (1C, $CH_2\underline{C}H_2CH_2$), 26.82 (1C, $CH_2\underline{C}H_2CH_2$), 25.10 (1C, $CH_2\underline{C}H_2CH_2$), 23.86 (1C, $CH_2\underline{C}H_2CH_2$).

Preparation of 1-{4-{4-[7-(4-chlorophenoxy)heptyl]piperazin-1-yl}but-1-yl}-2,3-di(*tert*-butoxycarbonyl)-1-[4-(trifluoromethyl)benzyl]guanidine (**7b**)

1,3-bis(*tert*-butoxycarbonyl)-2-methylisothiourea (0.132 g; $4.54 \cdot 10^{-4}$ mol) and mercury II chloride (0.123 g; $4.53 \cdot 10^{-4}$ mol) were sequentially added to an ice-cooled mixture of 4-{4-[7-(4-chlorophenoxy)heptyl]piperazin-1-yl}-*N*-[4-(trifluoromethyl)benzyl]butan-1-amine (**6b**) (0.223 g; $4.13 \cdot 10^{-4}$ mol) and triethylamine (0.21 g; $7.73 \cdot 10^{-4}$ mol) in 30 mL DCM. The ice bath was removed and the reaction was stirred for eighteen hours at room temperature, then filtered. The precipitate was discarded. The filtrate was washed sequentially twice with 15 mL H_2O and twice with 15 mL brine. The combined organic phases were dried over Na_2SO_4, then the solvent was removed under vacuum and the crude product was purified by column chromatography (EtOAc/MeOH/Triethylamine 89:10:1) to yield the pure product.

1-{4-{4-[7-(4-chlorophenoxy)heptyl]piperazin-1-yl}but-1-yl}-2,3-di(*tert*-butoxycarbonyl)-1-[4-(trifluoromethyl)benzyl]guanidine (**7b**): $C_{40}H_{59}ClF_3N_5O_5$. M=782.38. Colourless oil. 78.02 % yield. R_f=0.41 (EtOAc/MeOH/Triethylamine 89:10:1). ^{1}H NMR (600 MHz, *CDCl₃*) δ ppm 10.01 (br, 1H, N<u>H</u>), 7.60-7.59 (m, 2H$^{arom.}$, C(CHC<u>H</u>)$_2$CCF$_3$), 7.43-7.42 (m, 2H$^{arom.}$, C(C<u>H</u>CH)$_2$CCF$_3$), 7.22-7.21 (m, 2H$^{phenoxy.}$, C(CHC<u>H</u>)$_2$CCl), 6.82-6.80 (m, 2H$^{phenoxy.}$, C(C<u>H</u>CH)$_2$CCl), 4.77 (br, 2H, NC<u>H$_2$</u>Ph), 3.91 (t, 2H, OC<u>H$_2$</u>; *J*=6.51Hz), 3.32 (br, 2H, CH$_2$C<u>H$_2$</u>NC(N)), 2.43 (br, 8H, 8H^{piperazine}), 2.32-2.27 (m, 4H, C<u>H$_2$</u>N^{piperazine}), 1.78-1.73 (m, 2H, OCH$_2$C<u>H$_2$</u>), 1.59-1.54 (m, 2H, CH$_2$C<u>H$_2$</u>CH$_2$), 1.50 (m, 18H, C<u>H$_3$</u>), 1.46-1.42 (m, 6H, CH$_2$C<u>H$_2$</u>CH$_2$), 1.39-1.31 (m, 4H, CH$_2$C<u>H$_2$</u>CH$_2$). ^{13}C NMR (150.95 MHz, *CDCl₃*) δ ppm 171.14 (1C, <u>C</u>=O), 162.62 (1C, <u>C</u>=O), 157.70 (1C$^{quat./phenoxy.}$, <u>C</u>O), 156.24 (1C$^{quat.}$, <u>C</u>=N), 140.68 (1C$^{quat./arom.}$, <u>C</u>(CHCH)$_2$CCF$_3$), 130.08, 129.82, 129.55, 129.34 (1C$^{quat./arom.}$, <u>C</u>CF$_3$), 129.23 (2C$^{phenoxy.}$, C(CH<u>C</u>H)$_2$CCl), 127.99 (2C$^{arom.}$, C(<u>C</u>HCH)$_2$CCF$_3$), 126.79, 124.99, 123.19, 121.38 (1C, <u>C</u>F$_3$), 125.59,125.56 (2C$^{arom.}$, C(CH<u>C</u>H)$_2$CCF$_3$), 125.26 (1C$^{quat./phenoxy.}$, C(CHCH)$_2$<u>C</u>Cl), 115.72 (2C$^{phenoxy.}$, C(<u>C</u>HCH)$_2$CCl), 82.13 (1C$^{quat.}$ Boc), 79.63 (1C$^{quat.}$ Boc), 68.23 (1C, O<u>C</u>H$_2$), 58.76 (1C, CH$_2$<u>C</u>H$_2$N^{piperazine}), 57.92 (1C, CH$_2$<u>C</u>H$_2$N^{piperazine}), 53.23, 53.14 (4C^{piperazine}), 50.97 (1C, N<u>C</u>H$_2$Ph), 48.13 (1C, CH$_2$<u>C</u>H$_2$NC(N)), 29.28 (1C, CH$_2$<u>C</u>H$_2$CH$_2$), 29.12 (1C, CH$_2$<u>C</u>H$_2$CH$_2$), 28.15 (6C, <u>C</u>H$_3$), 28.04 (1C, CH$_2$<u>C</u>H$_2$CH$_2$), 27.51 (1C, CH$_2$<u>C</u>H$_2$CH$_2$), 26.82 (1C, CH$_2$<u>C</u>H$_2$CH$_2$), 25.21 (1C, CH$_2$<u>C</u>H$_2$CH$_2$), 23.86 (1C, CH$_2$<u>C</u>H$_2$CH$_2$).

Preparation of 1-benzyl-1-{4-{4-[7-(4-chlorophenoxy)heptyl]piperazin-1-yl}but-1-yl}guanidine trihydrochloride (**ADS10377**)

4M solution HCl-dioxan (1.92 mL; $7.69 \cdot 10^{-3}$ mol) was added dropwise to a solution of the 1-benzyl-1-{4-{4-[7-(4-chlorophenoxy)heptyl]piperazin-1-yl}but-1-yl}-2,3-di(*tert*-butoxycarbonyl)guanidine (**7a**) (0.275 g; $3.85 \cdot 10^{-4}$ mol) in 20 mL chloroform. The reaction was stirred overnight at room temperature, then the solvent was removed under vacuum. The crude product was evaporated twice from chloroform and twice from EtOAc, then recrystallized from anhydrous ethanol to yield the pure product.

1-benzyl-1-{4-{4-[7-(4-chlorophenoxy)heptyl]piperazin-1-yl}but-1-yl}guanidine trihydrochloride (**ADS10377**): $C_{29}H_{44}ClN_5O \cdot 3HCl$. M=623.53. White solid. 71.25 %. mp: 215.1-216.8 °C. ^{1}H NMR (600 MHz, *CD₃OD*) δ ppm 7.46-7.43 (m, 2H$^{arom.}$, C(CHC<u>H</u>)$_2$CH), 7.39-7.36 (m, 1H$^{arom.}$, C(CHCH)$_2$C<u>H</u>), 7.33-7.31 (m, 2H$^{arom.}$, C(C<u>H</u>CH)$_2$CH), 7.27-7.26 (m, 2H$^{phenoxy.}$, C(CHC<u>H</u>)$_2$CCl), 6.92-6.91 (m, 2H$^{phenoxy.}$, C(C<u>H</u>CH)$_2$CCl), 4.72 (s, 2H, NC<u>H$_2$</u>Ph), 4.01-3.99 (t, 2H, OC<u>H$_2$</u>; *J*=6.36Hz), 3.89 (br, 4H^{piperazine}), 3.65 (br, 4H^{piperazine}), 3.47-3.45 (t, 2H, CH$_2$C<u>H$_2$</u>NC(N), *J*=7.80Hz), 3.32-3.29 (m, 4H, CH$_2$C<u>H$_2$</u>N^{piperazine}), 1.89-1.76 (m, 8H, CH$_2$C<u>H$_2$</u>CH$_2$), 1.57-1.55 (m, 2H, CH$_2$C<u>H$_2$</u>CH$_2$), 1.51-1.49 (m, 4H, CH$_2$C<u>H$_2$</u>CH$_2$). ^{13}C NMR (150.95 MHz, *CD₃OD*) δ ppm 159.36 (1C$^{quat./phenoxy.}$,

$\underline{C}O$), 158.40 (1C, $\underline{C}$=N), 136.31 ($1C^{quat./arom.}$, $\underline{C}(CHCH)_2C$), 130.30 ($2C^{phenoxy.}$, $C(CH\underline{C}H)_2CCl$), 130.15 ($2C^{arom.}$, $C(CH\underline{C}H)_2CH$), 129.20 ($1C^{arom.}$, $C(CHCH)_2\underline{C}H$), 128.02 ($2C^{arom.}$, $C(\underline{C}HCH)_2CH$), 126.37 ($1C^{quat./phenoxy.}$, $C(CHCH)_2\underline{C}Cl$), 117.04 ($2C^{phenoxy.}$, $C(\underline{C}HCH)_2CCl$), 69.26 (1C, $O\underline{C}H_2$), 58.21 (1C, $CH_2\underline{C}H_2N^{piperazine}$), 57.52 (1C, $CH_2\underline{C}H_2N^{piperazine}$), 52.78 (1C, $N\underline{C}H_2Ph$), 49.85 ($4C^{piperazine}$), 49.62 (1C, $CH_2\underline{C}H_2NC(N)$), 30.16 (1C, $CH_2\underline{C}H_2CH_2$), 29.81 (1C, $CH_2\underline{C}H_2CH_2$), 27.45 (1C, $CH_2\underline{C}H_2CH_2$), 26.87 (1C, $CH_2\underline{C}H_2CH_2$), 25.27 (1C, $CH_2\underline{C}H_2CH_2$), 24.86 (1C, $CH_2\underline{C}H_2CH_2$), 22.05 (1C, $CH_2\underline{C}H_2CH_2$). Anal. Calcd: C 55.86 %; H 7.60 %; N 11.23 %. Found: C 55.89 %; H 7.71 %; N 11.07 %.

Preparation of 1-{4-{4-[7-(4-chlorophenoxy)heptyl]piperazin-1-yl}but-1-yl}-1-[4-(trifluoromethyl)benzyl]guanidine trihydrochloride (**ADS10376**)

4M solution HCl-dioxan (1.78 mL; $7.13 \cdot 10^{-3}$ mol) was added dropwise to a solution of 1-{4-{4-[7-(4-chlorophenoxy)heptyl]piperazin-1-yl}but-1-yl}-2,3-di(*tert*-butoxycarbonyl)-1-[4-(trifluoromethyl)benzyl]guanidine (**7b**) (0.279 g; $3.57 \cdot 10^{-4}$ mol) in 20 mL chloroform. The reaction was stirred overnight at room temperature, then the solvent was removed under vacuum. The crude product was evaporated twice from chloroform and twice from EtOAc, then recrystallized from anhydrous ethanol to yield the pure product.

1-{4-{4-[7-(4-chlorophenoxy)heptyl]piperazin-1-yl}but-1-yl}-1-[4-(trifluoromethyl)benzyl]guanidine trihydrochloride (**ADS10376**): $C_{30}H_{43}ClF_3N_5O \cdot 3HCl$. M=691.53. White solid. 75.83 %. mp: 233.5-235.1 °C. ^1H NMR (600 MHz, *CD_3OD*) δ ppm 7.76-7.75 (m, $2H^{arom.}$, $C(CHC\underline{H})_2CCF_3$), 7.52-7.50 (m, $2H^{arom.}$, $C(C\underline{H}CH)_2CCF_3$), 7.27-7.26 (m, $2H^{phenoxy.}$, $C(CHC\underline{H})_2CCl$), 6.92-6.91 (m, $2H^{phenoxy.}$, $C(C\underline{H}CH)_2CCl$), 4.79 (br, 2H, $NC\underline{H}_2Ph$), 4.01-3.99 (t, 2H, $OC\underline{H}_2$; *J*=6.35Hz), 3.91 (br, $4H^{piperazine}$), 3.65 (br, $4H^{piperazine}$), 3.51-3.49 (t, 2H, $CH_2C\underline{H}_2NC(N)$, *J*=7.8Hz), 3.33-3.31 (m, 4H, $CH_2C\underline{H}_2N^{piperazine}$;), 1.88-1.80 (m, 8H, $CH_2C\underline{H}_2CH_2$), 1.57-1.54 (m, 2H, $CH_2C\underline{H}_2CH_2$), 1.51-1.49 (m, 4H, $CH_2C\underline{H}_2CH_2$). ^{13}C NMR (150.95 MHz, *CD_3OD*) δ ppm 159.58 ($1C^{quat./phenoxy.}$, $\underline{C}O$), 158.53 (1C, $\underline{C}$=N), 141.07 ($1C^{quat./arom.}$, $\underline{C}(CHCH)_2CCF_3$), 131.56, 131.35, 131.14, 130.94 ($1C^{quat./arom.}$, $\underline{C}CF_3$), 130.30 ($2C^{phenoxy.}$, $C(CH\underline{C}H)_2CCl$), 128.44 ($2C^{arom.}$, $C(\underline{C}HCH)_2CCF_3$), 128.28, 126.48, 124.68, 122.88 (1C, $\underline{C}F_3$), 126.97, 126.95 ($2C^{arom.}$, $C(CH\underline{C}H)_2CCF_3$), 126.37 ($1C^{quat./phenoxy.}$, $C(CHCH)_2\underline{C}Cl$), 117.03 ($2C^{phenoxy.}$, $C(\underline{C}HCH)_2CCl$), 69.26 (1C, $O\underline{C}H_2$), 57.52 (1C, $CH_2\underline{C}H_2N^{piperazine}$), 54.13 (1C, $\underline{C}H_2N^{piperazine}$), 52.39 (1C, $N\underline{C}H_2Ph$), 50.03 ($4C^{piperazine}$), 49.72 (1C, $CH_2\underline{C}H_2NC(N)$), 30.17 (1C, $CH_2\underline{C}H_2CH_2$), 29.81 (1C, $CH_2\underline{C}H_2CH_2$), 27.45 (1C, $CH_2\underline{C}H_2CH_2$), 26.88 (1C, $CH_2\underline{C}H_2CH_2$), 25.33 (1C, $CH_2\underline{C}H_2CH_2$), 24.87 (1C, $CH_2\underline{C}H_2CH_2$), 22.02 (1C, $CH_2\underline{C}H_2CH_2$). Anal. Calcd: C 52.11 %; H 6.70 %; N 10.13 %. Found: C 52.25 %; H 6.91 %; N 9.95 %.

Preparation of (3-bromopropyl)benzene (**8**)

3-Phenyl-1-propanol (0.50 g; $3.67 \cdot 10^{-3}$ mol) in 7 mL toluene was slowly added to an ice-cooled mixture of phosphorus tribromide (1.39 g; $5.14 \cdot 10^{-3}$ mol) in 10 mL toluene. The ice bath was removed and the reaction was stirred overnight at room temperature. Then 50 g of ice-cubes was added and the mixture was extracted 3x20 mL with DCM. The combined organic phases were dried over anhydrous Na_2SO_4. The solvent was removed under vacuum and the crude product was purified by column chromatography (Hexane/DCM 10:1) to yield the pure product.

(3-bromopropyl)benzene (**8**): $C_9H_{11}Br$. M=199.09. Colourless liquid. 65.45 % yield. R_f=0.78 (Hexane/DCM 10:1). ^{1}H NMR (600 MHz, *CDCl₃*) δ ppm 7.31-7.28 (m, 2H$^{phenyl.}$, C(CHC<u>H</u>)$_2$CH), 7.22-7.19 (m, 3H$^{phenyl.}$, C(C<u>H</u>CH)$_2$C<u>H</u>), 3.40 (t, 2H, C<u>H</u>$_2$Br, *J* = 6.59Hz), 2.78 (t, 2H, PhC<u>H</u>$_2$, *J* = 7.37Hz), 2.17 (qt, 4H, CH$_2$C<u>H</u>$_2$CH$_2$). ^{13}C NMR (150.95 MHz, *CDCl₃*) δ ppm 140.53 (1C$^{quat./phenyl}$, <u>C</u>CH$_2$), 128.55 (2C$^{phenyl.}$, C(<u>C</u>HCH)$_2$CH), 128.49 (2C$^{phenyl.}$, C(CH<u>C</u>H)$_2$CH), 126.16 (1C$^{phenyl.}$, C(CHCH)$_2$<u>C</u>H), 34.16 (1C, CH$_2$<u>C</u>H$_2$CH$_2$), 39.97 (1C, Ph<u>C</u>H$_2$), 33.12 (1C, <u>C</u>H$_2$Br).

Preparation of 3-(4-chlorophenyl)propyl methanesulfonate (**9**)

Methanesulfonyl chloride (0.56 g; $4.89 \cdot 10^{-3}$ mol) and triethylamine (0.69 g; $6.82 \cdot 10^{-3}$ mol) were sequentially added to an ice-cooled 3-(4-Chlorophenyl)-1-propanol (0.60 g; $3.52 \cdot 10^{-3}$ mol) in 15 mL DCM. The ice bath was removed and the reaction was stirred for one hour at room temperature. The mixture was sequentially washed with 10 mL water and 10 mL brine and dried over Na_2SO_4. The solvent was removed under vacuum and the crude product was purified by column chromatography (DCM) to yield the pure product.

3-(4-chlorophenyl)propyl methanesulfonate (**9**): $C_{10}H_{13}ClO_3S$. M=248.73. Colourless oil. 88.10 % yield. R_f=0.78 (DCM). ^{1}H NMR (600 MHz, *CDCl₃*) δ ppm 7.27-7.26 (m, 2H$^{phenyl.}$, C(CHC<u>H</u>)$_2$CCl), 7.13-7.12 (m, 2H$^{phenyl.}$, C(C<u>H</u>CH)$_2$CCl), 4.22 (t, 2H, OC<u>H</u>$_2$, *J* = 6.31Hz), 2.99 (s, 3H, CH$_3$), 2.73 (t, 2H, PhC<u>H</u>$_2$), 2.06 (qt, 2H, CH$_2$C<u>H</u>$_2$CH$_2$). ^{13}C NMR (150.95 MHz, *CDCl₃*) δ ppm 138.74 (1C$^{quat./phenyl}$, <u>C</u>CH$_2$), 132.08 (1C$^{quat./phenyl.}$, <u>C</u>Cl), 129.77 (2C$^{phenyl.}$, C(<u>C</u>HCH)$_2$CCl), 128.68 (2C$^{phenyl.}$, C(CH<u>C</u>H)$_2$CCl), 68.78 (1C, O<u>C</u>H$_2$), 37.40 (1C, S<u>C</u>H$_3$), 30.91(1C, Ph<u>C</u>H$_2$), 30.58 (1C, CH$_2$<u>C</u>H$_2$CH$_2$).

Preparation of 4-(piperazin-1-yl)butanenitrile (**10**)

4-bromobutyronitrile (1.00 g; $6.76 \cdot 10^{-3}$ mol) in 20 mL chloroform was added dropwise to a solution of piperazine (2.91 g; $3.38 \cdot 10^{-2}$ mol) in 60 mL chloroform heated to 60 °C. The reaction was stirred for two hours at 60 °C. The precipitate was discarded. The solvent was removed under vacuum and the crude product was purified by column chromatography (DCM/MeOH/25% NH_3 aq. 89:10:1) to yield the pure product.

4-(piperazin-1-yl)butanenitrile (**10**): $C_8H_{15}N_3$. M=153.22. Colourless oil. 89.86 % yield. R_f=0.34 (DCM/MeOH/25% NH_3 aq. 89:10:1). ^{1}H NMR (600 MHz, *CDCl₃*) δ ppm 2.89-2.87 (m, 4H^{piperazine}), 2.45-2.41 (m, 8H: 4H^{piperazine}, N^{piperazine}C<u>H</u>$_2$CH$_2$C<u>H</u>$_2$CN), 2.17 (s, 1H, N<u>H</u>), 1.84-1.79 (qt, 2H, C<u>H</u>$_2$CH$_2$CH$_2$). ^{13}C NMR (150.95 MHz, *CDCl₃*) δ ppm 119.63 (1C$^{quat.}$, <u>C</u>≡N), 56.78 (1C, N^{piperazine}<u>C</u>H$_2$CH$_2$CH$_2$CN), 54.22 (2C^{piperazine}), 45.86 (2C^{piperazine}), 22.48 (1C, <u>C</u>H$_2$CH$_2$CN), 14.79 (1C, <u>C</u>H$_2$CN).

Preparation of 4-[4-(3-hydroxypropyl)piperazin-1-yl]butanenitrile (**11**)

3-Bromo-1-propanol (0.81 g; $5.83 \cdot 10^{-3}$ mol) was added to a solution of Triethylamine (2.24 g; $2.21 \cdot 10^{-2}$ mol) and 4-(piperazin-1-yl)butanenitrile (**10**) (0.85 g; $5.42 \cdot 10^{-3}$ mol) in 30 mL acetonitrile. The reaction was stirred overnight at 80 °C. The solvent was removed under vacuum and the residue was diluted with 15 mL of 5 % potassium carbonate solution and extracted 2x20 mL with chloroform. The combined organic phases were dried over anhydrous Na_2SO_4. The solvent was removed under vacuum and the crude product was purified by column chromatography (EtOAc/MeOH/Triethylamine 30:10:1) to yield the pure product.

4-[4-(3-hydroxypropyl)piperazin-1-yl]butanenitrile (**11**): $C_{11}H_{21}N_3O$. M=211.30. Yellowish sticky oil. 85.38 % yield. R_f=0.22 (EtOAc/MeOH/Triethylamine 30:10:1). ^{1}H NMR (600 MHz, *CDCl₃*) δ ppm 4.95 (br, 1H, $O\underline{H}$), 3.79 (t, 2H, $OC\underline{H}_2$), 2.61 (t, 2H, $HOCH_2CH_2C\underline{H}_2N^{piperazine}$), 2.55-2.40 (m, 12H: $8H^{piperazine}$, $N^{piperazine}C\underline{H}_2CH_2C\underline{H}_2CN$), 1.80 (qt, 2H, $HOCH_2C\underline{H}_2$), 1.71 (qt, 2H, $C\underline{H}_2CH_2CN$). ^{13}C NMR (150.95 MHz, *$CDCl_3$*) δ ppm 119.62 ($1C^{quat.}$, $\underline{C}{\equiv}N$), 64.46 (1C, $O\underline{C}H_2$), 58.62 (1C, $HOCH_2CH_2C\underline{H}_2N^{piperazine}$), 56.08 (1C, $N^{piperazine}\underline{C}H_2CH_2CH_2CN$), 53.22 ($2C^{piperazine}$), 52.99 ($2C^{piperazine}$), 27.11 (1C, $HOCH_2\underline{C}H_2$), 22.69 (1C, $\underline{C}H_2CH_2CN$), 14.83 (1C, $\underline{C}H_2CN$).

Preparation of 4-{4-[3-(3-phenylpropoxy)propyl]piperazin-1-yl}butanenitrile (**12a**)

TBAI (tetra-n-butylammonium iodide) (0.017 g; $4.60{\cdot}10^{-5}$ mol) was added to a solution of 4-[4-(3-hydroxypropyl)piperazin-1-yl]butanenitrile (**11**) (0.197 g; $9.32{\cdot}10^{-4}$ mol) and (3-bromopropyl)benzene (**8**) (0.186 g; $9.34{\cdot}10^{-4}$ mol) in 2.05 mL 40 % NaOH. The reaction was stirred overnight at room temperature, then the mixture was extracted 4x2.5 mL with diethyl ether. The combined organic phases were dried over anhydrous Na_2SO_4. The solvent was removed under vacuum and the crude product was purified by column chromatography (EtOAc/MeOH/Triethylamine 30:5:1) to yield the pure product.

4-{4-[3-(3-phenylpropoxy)propyl]piperazin-1-yl}butanenitrile (**12a**): $C_{20}H_{31}N_3O$. M=329.48. Yellowish sticky oil. 40.72 % yield. R_f=0.43 (EtOAc/MeOH/Triethylamine 30:5:1). ^{1}H NMR (600 MHz, *$CDCl_3$*) δ ppm 7.29-7.26 (m, $2H^{phenyl.}$, $C(CHC\underline{H})_2CH$), 7.19-7.17 (m, $3H^{phenyl.}$, $C(C\underline{H}CH)_2C\underline{H}$), 3.45 (t, 2H, $OC\underline{H}_2CH_2CH_2N^{piperazine}$, J = 6.42Hz), 3.41 (t, 2H, $OC\underline{H}_2CH_2CH_2Ph$, J = 6.42Hz), 2.68 (t, 2H, $PhC\underline{H}_2$, J = 7.50Hz), 2.53-2.41 (m, 14H: $8H^{piperazine}$, $C\underline{H}_2N^{piperazine}$, $C\underline{H}_2CN$), 1.91-1.86 (m, 2H, $C\underline{H}_2CH_2Ph$), 1.85-1.77 (m, 4H, $CH_2C\underline{H}_2CH_2$). ^{13}C NMR (150.95 MHz, *$CDCl_3$*) δ ppm 141.99 ($1C^{quat./phenyl}$, $\underline{C}CH_2$), 128.46 ($2C^{phenyl.}$, $C(\underline{C}HCH)_2CH$), 128.29 ($2C^{phenyl.}$, $C(CH\underline{C}H)_2CH$), 125.74 ($1C^{phenyl.}$, $C(CHCH)_2\underline{C}H$), 119.77 ($1C^{quat.}$, $\underline{C}{\equiv}N$), 69.95 (1C, $O\underline{C}H_2CH_2CH_2Ph$), 69.04 (1C, $OC\underline{H}_2CH_2CH_2N^{piperazine}$), 56.25 (1C, $N{\equiv}CCH_2CH_2\underline{C}H_2N^{piperazine}$), 55.53 (1C, $OCH_2CH_2\underline{C}H_2N^{piperazine}$), 53.09 ($2C^{piperazine}$), 52.85 ($2C^{piperazine}$), 32.33 (1C, $Ph\underline{C}H_2$), 31.27 (1C, $PhCH_2\underline{C}H_2$), 27.01 (1C, $OCH_2\underline{C}H_2CH_2N^{piperazine}$), 22.71 (1C, $\underline{C}H_2CH_2CN$), 14.93 (1C, $\underline{C}H_2CN$).

Preparation of 4-{4-{3-[3-(4-chlorophenyl)propoxy]propyl}piperazin-1-yl}butanenitrile (**12b**)

Sodium hydride (60 % dispersion in mineral oil) (0.14 g; $3.50{\cdot}10^{-3}$ mol) was slowly added to a solution of 4-[4-(3-hydroxypropyl)piperazin-1-yl]butanenitrile (**11**) (0.458 g; $2.17{\cdot}10^{-3}$ mol) in 10 mL anhydrous *N,N*-dimethylacetamide under an argon atmosphere. The reaction was stirred for 90 minutes at 50 °C. Then, the mixture was cooled at 25 °C and a solution of 3-(4-chlorophenyl)propyl methanesulfonate (**9**) (0.65 g; $2.61{\cdot}10^{-3}$ mol) in 5 mL anhydrous *N,N*-dimethylacetamide was slowly (2 hours) added. The reaction was stirred overnight at room temperature. Then the mixture was cooled at 10 °C and a solution of 0.51 g sodium chloride in 6.1 g water was slowly added. The mixture was extracted 4x20 mL with toluene. The combined organic phases were extracted

1x8mL 2M HCl. The aqueous phase was then washed with 10 mL toluene. The aqueous phase was treated with 0.9 mL 6M sodium hydroxide to pH 12, and then extracted 3x10 mL with water. The organic phase was dried over anhydrous Na_2SO_4. The solvent was removed under vacuum to yield the pure product.

4-{4-{3-[3-(4-chlorophenyl)propoxy]propyl}piperazin-1-yl}butanenitrile (**12b**): $C_{20}H_{30}ClN_3O$. M=363.92. Yellowish oil. 79.43 % yield. R_f=0.59 (EtOAc/MeOH/Triethylamine 30:5:1). ^{1}H NMR (600 MHz, *CDCl₃*) δ ppm 7.24-7.23 (m, $2H^{phenyl.}$, $C(CHC\underline{H})_2CHCl$), 7.12-7.10 (m, $2H^{phenyl.}$, $C(C\underline{H}CH)_2CHCl$), 3.43 (t, 2H, $OC\underline{H}_2CH_2CH_2N^{piperazine}$, J = 6.34Hz), 3.49 (t, 2H, $OC\underline{H}_2CH_2CH_2Ph$, J = 6.23Hz), 2.65 (t, 2H, $PhC\underline{H}_2$, J = 7.61Hz), 2.46-2.40 (m, 14H: $8H^{piperazine}$, $C\underline{H}_2N^{piperazine}$, $C\underline{H}_2CN$), 1.87-1.74 (m, 6H, $CH_2C\underline{H}_2CH_2$). ^{13}C NMR (150.95 MHz, *CDCl₃*) δ ppm 140.45 ($1C^{quat./phenyl}$, $\underline{C}CH_2$), 131.47 ($1C^{quat./phenyl.}$, $\underline{C}Cl$), 129.81 ($2C^{phenyl.}$, $C(\underline{C}HCH)_2CCl$), 128.38 ($2C^{phenyl.}$, $C(CH\underline{C}H)_2CCl$), 119.71 ($1C^{quat.}$, $\underline{C}\equiv N$), 69.65 (1C, $O\underline{C}H_2CH_2CH_2Ph$), 69.17 (1C, $OC\underline{H}_2CH_2CH_2N^{piperazine}$), 56.29 (1C, $\underline{C}H_2N^{piperazine}$), 55.49 (1C, $\underline{C}H_2N^{piperazine}$), 53.21 ($2C^{piperazine}$), 53.11 ($2C^{piperazine}$), 31.70 (1C, $Ph\underline{C}H_2$), 31.17 (1C, $PhCH_2CH_2OCH_2\underline{C}H_2$), 27.23 (1C, $OCH_2\underline{C}H_2CH_2N^{piperazine}$), 22.80 (1C, $\underline{C}H_2CH_2CN$), 14.90 (1C, $\underline{C}H_2CN$).

Preparation of 4-{4-[3-(3-phenylpropoxy)propyl]piperazin-1-yl}butan-1-amine (**13a**)

$LiAlH_4$ (0.34 g; $8.96\cdot10^{-3}$ mol) was added to a solution of 4-{4-[3-(3-phenylpropoxy)propyl]piperazin-1-yl}butanenitrile (**12a**) (0.743 g; $2.26\cdot10^{-3}$ mol) in 30 mL anhydrous diethyl ether. The reaction was stirred overnight at room temperature, then the mixture was quenched by dropwise addition of water (16 equiv.) and 10 % NaOH solution (16 equiv.) stirred for two hours, then filtered. The precipitate was discarded. The organic layer was dried over Na_2SO_4, then the solvent was removed under vacuum and the crude product was purified by column chromatography (DCM/MeOH/25% NH_3aq. 39:10:1) to yield the pure product.

4-{4-[3-(3-phenylpropoxy)propyl]piperazin-1-yl}butan-1-amine (**13a**): $C_{20}H_{35}N_3O$. M=333.51. Yellowish sticky oil. 84.54 % yield. R_f=0.65 (DCM/MeOH/25% NH_3aq. 39:10:1). ^{1}H NMR (600 MHz, *CDCl₃*) δ ppm 7.29-7.27 (m, $2H^{phenyl.}$, $C(CHC\underline{H})_2CH$), 7.19-7.17 (m, $3H^{phenyl.}$, $C(C\underline{H}CH)_2C\underline{H}$), 3.44 (t, 2H, $OC\underline{H}_2CH_2CH_2N^{piperazine}$, J = 6.46Hz), 3.41 (t, 2H, $OC\underline{H}_2CH_2CH_2Ph$, J = 6.44Hz), 2.77 (t, 2H, $C\underline{H}_2NH_2$, J = 6.32Hz), 2.68 (t, 2H, $PhC\underline{H}_2$, J = 7.80 Hz), 2.55-2.37 (m, 14H: $8H^{piperazine}$, $C\underline{H}_2N^{piperazine}$, $N\underline{H}_2$*), 1.89 (qt, 2H, $C\underline{H}_2CH_2Ph$), 1.77 (qt, 2H, $OCH_2C\underline{H}_2CH_2N^{piperazine}$), 1.59-1.55 (m, 4H, $NH_2CH_2C\underline{H}_2C\underline{H}_2$). ^{13}C NMR (150.95 MHz, *CDCl₃*) δ ppm 141.96 ($1C^{quat./phenyl}$, $\underline{C}CH_2$), 128.43 ($2C^{phenyl.}$, $C(\underline{C}HCH)_2CH$), 128.27 ($2C^{phenyl.}$, $C(CH\underline{C}H)_2CH$), 125.69 ($1C^{phenyl.}$, $C(CHCH)_2\underline{C}H$), 69.89 (1C, $O\underline{C}H_2CH_2CH_2Ph$), 69.07 (1C, $OC\underline{H}_2CH_2CH_2N^{piperazine}$), 58.19 (1C, NH_2CH_2 $CH_2CH_2\underline{C}H_2N^{piperazine}$), 55.42 (1C, $OCH_2CH_2\underline{C}H_2N^{piperazine}$), 53.04 ($2C^{piperazine}$), 52.96 ($2C^{piperazine}$), 41.30 (1C, $\underline{C}H_2NH_2$), 32.29 (1C, $Ph\underline{C}H_2$), 31.24 (1C, $PhCH_2\underline{C}H_2$), 30.36, 24.36 (2C, $NH_2CH_2\underline{C}H_2\underline{C}H_2$), 27.14 (1C, $OCH_2\underline{C}H_2CH_2N^{piperazine}$).

Preparation of 4-{4-{3-[3-(4-chlorophenyl)propoxy]propyl}piperazin-1-yl}butan-1-amine (**13b**)

$LiAlH_4$ (0.18 g; $4.74\cdot10^{-3}$ mol) was added to a solution of 4-{4-{3-[3-(4-chlorophenyl)propoxy]propyl}piperazin-1-yl}butanenitrile (**12b**) (0.43 g; $1.18\cdot10^{-3}$ mol) in 50 mL anhydrous diethyl ether. The reaction was stirred

overnight at room temperature, then the mixture was quenched by dropwise addition of water (16 equiv.) and 10 % NaOH solution (16 equiv.) stirred for two hours, then filtered. The precipitate was discarded. The organic layer was dried over Na_2SO_4, then the solvent was removed under vacuum and the crude product was purified by column chromatography (DCM/MeOH/25% NH_3aq. 89:10:1) to yield the pure product.

4-{4-{3-[3-(4-chlorophenyl)propoxy]propyl}piperazin-1-yl}butan-1-amine (**13b**): $C_{20}H_{34}ClN_3O$. M=367.96. Yellowish oil. 82.95 % yield. R_f=0.40 (DCM/MeOH/25% NH_3aq. 89:10:1). ^1H NMR (600 MHz, *CDCl₃*) δ ppm 7.24-7.22 (m, $2H^{phenyl.}$, $C(CHC\underline{H})_2CCl$), 7.11-7.09 (m, $2H^{phenyl.}$, $C(C\underline{H}CH)_2CCl$), 3.43 (t, 2H, $OC\underline{H}_2CH_2CH_2N^{piperazine}$, J = 6.45Hz), 3.38 (t, 2H, $OC\underline{H}_2CH_2CH_2Ph$, J = 6.34Hz), 2.71 (t, 2H, $C\underline{H}_2NH_2$, J = 6.87Hz), 2.65 (t, 2H, $PhC\underline{H}_2$), 2.48 (br, $8H^{piperazine}$), 2.42 (t, 2H, $C\underline{H}_2N^{piperazine}$), 2.34 (t, 2H, $C\underline{H}_2N^{piperazine}$), 1.85 (qt, 2H, $C\underline{H}_2CH_2Ph$), 1.76 (qt, 2H, $OCH_2C\underline{H}_2CH_2N^{piperazine}$), 1.55-1.45 (m, 2H, $CH_2C\underline{H}_2CH_2$), 1.47-1.43 (m, 2H, $CH_2C\underline{H}_2CH_2$), 1.37 (br, 2H, $N\underline{H}_2$). ^{13}C NMR (150.95 MHz, *CDCl₃*) δ ppm 140.46 ($1C^{quat./phenyl}$, $\underline{C}CH_2$), 131.47 ($1C^{quat./phenyl.}$, $\underline{C}Cl$), 129.82 ($2C^{phenyl.}$, $C(\underline{C}HCH)_2CCl$), 128.38 ($2C^{phenyl.}$, $C(CH\underline{C}H)_2CCl$), 69.64 (1C, $O\underline{C}H_2CH_2CH_2Ph$), 69.22 (1C, $OC\underline{H}_2CH_2CH_2N^{piperazine}$), 58.56 (1C, NH_2CH_2 $CH_2CH_2\underline{C}H_2N^{piperazine}$), 55.57 (1C, $OCH_2CH_2\underline{C}H_2N^{piperazine}$), 53.29 ($4C^{piperazine}$), 42.18 (1C, $\underline{C}H_2NH_2$), 31.89 (1C, $CH_2\underline{C}H_2CH_2$), 31.70 (1C, $Ph\underline{C}H_2$), 31.18 (1C, $\underline{C}H_2CH_2Ph$), 27.24 (1C, $OCH_2\underline{C}H_2CH_2N^{piperazine}$), 24.35 (1C, $CH_2\underline{C}H_2CH_2$).

Preparation of *N*-{4-{4-[3-(3-phenylpropoxy)propyl]piperazin-1-yl}butyl}benzamide (**14a**)

Benzoyl chloride (0.14 g; $9.96\cdot10^{-4}$ mol) in 10 mL DCM was added dropwise to a solution of 4-{4-[3-(3-phenylpropoxy)propyl]piperazin-1-yl}butan-1-amine (**13a**) (0.30 g; $8.99\cdot10^{-4}$ mol) and triethylamine (0.45 g; $4.45\cdot10^{-3}$ mol) in 15 mL DCM. The reaction was stirred for three hours at room temperature. The mixture was washed 3-times with 15 mL water and dried over Na_2SO_4. The solvent was removed under vacuum and the crude product was purified by column chromatography (EtOAc/MeOH/Triethylamine 49:10:1) to yield the pure product.

N-{4-{4-[3-(3-phenylpropoxy)propyl]piperazin-1-yl}butyl}benzamide (**14a**): $C_{27}H_{39}N_3O_2$. M=437.62. Yellowish sticky oil. 81.55 % yield. R_f=0.36 (EtOAc/MeOH/Triethylamine 49:10:1). ^1H NMR (600 MHz, *CDCl₃*) δ ppm 7.76-7.75 (m, $2H^{arom.}$, $C(C\underline{H}CH)_2CH$), 7.49-7.45 (m, $1H^{arom.}$, $C(CHCH)_2C\underline{H}$), 7.43-7.41 (m, $2H^{arom.}$, $C(CHC\underline{H})_2CH$), 7.29-7.26 (m, $2H^{phenyl.}$, $C(CHC\underline{H})_2CH$), 7.19-7.17 (m, $3H^{phenyl.}$, $C(C\underline{H}CH)_2C\underline{H}$), 6.73 (br, 1H, $N\underline{H}$), 3.48 (dt, 2H, $C\underline{H}_2NH$), 3.44 (t, 2H, $OC\underline{H}_2CH_2CH_2N^{piperazine}$, J = 6.45Hz), 3.41 (t, 2H, $OC\underline{H}_2CH_2CH_2Ph$, J = 6.43Hz), 2.68 (t, 2H, $PhC\underline{H}_2$, J = 7.20 Hz), 2.51-2.35 (m, 12H: $8H^{piperazine}$, $C\underline{H}_2N^{piperazine}$), 1.89 (qt, 2H, $C\underline{H}_2CH_2Ph$), 1.76 (qt, 2H, $OCH_2C\underline{H}_2CH_2N^{piperazine}$), 1.68-1.61 (m, 4H, $NH_2CH_2C\underline{H}_2C\underline{H}_2$). ^{13}C NMR (150.95 MHz, *CDCl₃*) δ ppm 167.72 ($1C^{quat.}$, C=O), 141.99 ($1C^{quat./phenyl}$, $\underline{C}CH_2$), 134.99 ($1C^{quat./arom.}$), 131.25 ($1C^{arom.}$, $C(CHCH)_2\underline{C}H$), 128.49 ($2C^{arom.}$, $C(CH\underline{C}H)_2CH$), 128.45 ($2C^{phenyl.}$, $C(\underline{C}HCH)_2CH$), 128.29 ($2C^{phenyl.}$, $C(CH\underline{C}H)_2CH$), 126.93 ($2C^{arom.}$, $C(\underline{C}HCH)_2CH$), 125.74 ($1C^{phenyl.}$, $C(CHCH)_2\underline{C}H$), 69.92 (1C, $O\underline{C}H_2CH_2CH_2Ph$), 69.09 (1C, $OC\underline{H}_2CH_2CH_2N^{piperazine}$), 57.91 (1C, $NHCH_2CH_2CH_2\underline{C}H_2N^{piperazine}$), 55.49 (1C, $OCH_2CH_2\underline{C}H_2N^{piperazine}$), 53.11 ($2C^{piperazine}$), 52.95 ($2C^{piperazine}$), 39.88 (1C, $\underline{C}H_2NH$), 32.32 (1C, $Ph\underline{C}H_2$), 31.27 (1C, $PhCH_2\underline{C}H_2$), 27.39, 24.38 (2C, $NH_2CH_2\underline{C}H_2\underline{C}H_2$), 27.11 (1C, $OCH_2\underline{C}H_2CH_2N^{piperazine}$).

Preparation of *N*-{4-{4-[3-(3-phenylpropoxy)propyl]piperazin-1-yl}butyl}-4-(trifluoromethyl)benzamide (**14b**)

4-(Trifluoromethyl)benzoyl chloride (0.25 g; $1.19\cdot10^{-3}$ mol) in 10 mL DCM was added dropwise to a solution of 4-{4-[3-(3-phenylpropoxy)propyl]piperazin-1-yl}butan-1-amine (**13a**) (0.359 g; $1.08\cdot10^{-3}$ mol) and triethylamine (0.53 g; $5.38\cdot10^{-3}$ mol) in 15 mL DCM. The reaction was stirred for three hours at room temperature. The mixture was washed three-times with 15 mL water and dried over Na_2SO_4. The solvent was removed under vacuum and the crude product was purified by column chromatography (EtOAc/MeOH/Triethylamine 39:10:1) to yield the pure product.

N-{4-{4-[3-(3-phenylpropoxy)propyl]piperazin-1-yl}butyl}-4-(trifluoromethyl)benzamide (**14b**): $C_{28}H_{38}F_3N_3O_2$. M=505.62. Yellowish sticky oil. 72.61 % yield. R_f=0.52 (EtOAc/MeOH/Triethylamine 39:10:1). ^{1}H NMR (600 MHz, *CDCl3*) δ ppm 7.88-7.87 (m, $2H^{arom.}$), 7.69-7.68 (m, $2H^{arom.}$), 7.29-7.26 (m, $2H^{phenyl.}$, $C(CHC\underline{H})_2CH$), 7.19-7.18 (m, $3H^{phenyl.}$, $C(C\underline{H}CH)_2C\underline{H}$), 7.05 (br, 1H, N$\underline{H}$), 3.48 (dt, 2H, $C\underline{H}_2NH$), 3.43 (t, 2H, $OC\underline{H}_2CH_2CH_2N^{piperazine}$, *J* = 6.48Hz), 3.41 (t, 2H, $OC\underline{H}_2CH_2CH_2Ph$, *J* = 6.44Hz), 2.68 (t, 2H, $PhC\underline{H}_2$, *J* = 7.80 Hz), 2.50-2.34 (m, 12H: $8H^{piperazine}$, $C\underline{H}_2N^{piperazine}$), 1.89 (qt, 2H, $C\underline{H}_2CH_2Ph$), 1.75 (qt, 2H, $OCH_2C\underline{H}_2CH_2N^{piperazine}$), 1.71-1.67 (m, 2H, $NH_2CH_2CH_2C\underline{H}_2$), 1.66-1.62 (m, 2H, $NH_2CH_2C\underline{H}_2CH_2$). ^{13}C NMR (150.95 MHz, *CDCl3*) δ ppm 166.49 ($1C^{quat.}$, C=O), 141.97 ($1C^{quat./phenyl}$, $\underline{C}CH_2$), 138.37 ($1C^{quat./arom.}$, $\underline{C}C(O)$), 133.28, 133.07, 132.85, 132.64 ($1C^{quat./arom.}$, $\underline{C}CF_3$), 128.45 ($2C^{phenyl.}$, $C(\underline{C}HCH)_2CH$), 128.29 ($2C^{phenyl.}$, $C(CH\underline{C}H)_2CH$), 125.74 ($1C^{phenyl.}$, $C(CHCH)_2\underline{C}H$), 127.49 ($2C^{arom.}$, $(\underline{C}HCH)_2C\ CF_3$), 126.39, 124.57, 122.78, 120.98 (1C, $\underline{C}F_3$), 125.54,125.52 ($2C^{arom.}$, $(\underline{C}H)_2CCF_3$), 69.93 (1C, $O\underline{C}H_2CH_2CH_2Ph$), 69.01 (1C, $OC\underline{H}_2CH_2CH_2N^{piperazine}$), 57.81 (1C, $NHCH_2CH_2CH_2\underline{C}H_2N^{piperazine}$), 55.44 (1C, $OCH_2CH_2\underline{C}H_2N^{piperazine}$), 53.06 ($2C^{piperazine}$), 52.81 ($2C^{piperazine}$), 39.99 (1C, $\underline{C}H_2NH$), 32.31 (1C, $Ph\underline{C}H_2$), 31.25 (1C, $PhCH_2\underline{C}H_2$), 27.25, 24.28 (2C, $NH_2CH_2\underline{C}H_2\underline{C}H_2$), 27.08 (1C, $OCH_2\underline{C}H_2CH_2N^{piperazine}$).

Preparation of *N*-{4-{4-{3-[3-(4-chlorophenyl)propoxy]propyl}piperazin-1-yl}butyl}benzamide (**14c**)

Benzoyl chloride (0.11 g; $7.82\cdot10^{-4}$ mol) in 10 mL DCM was added dropwise to a solution of 4-{4-{3-[3-(4-chlorophenyl)propoxy]propyl}piperazin-1-yl}butan-1-amine (**13b**) (0.25 g; $6.79\cdot10^{-4}$ mol) and triethylamine (0.34 g; $3.36\cdot10^{-3}$ mol) in 15 mL DCM. The reaction was stirred for three hours at room temperature. The mixture was washed three-times with 15 mL water and dried over Na_2SO_4. The solvent was removed under vacuum and the crude product was purified by column chromatography (EtOAc/MeOH/Triethylamine 49:10:1) to yield the pure product.

N-{4-{4-{3-[3-(4-chlorophenyl)propoxy]propyl}piperazin-1-yl}butyl}benzamide (**14c**): $C_{27}H_{38}ClN_3O_2$. M=472.06. Yellowish sticky oil. 90.74 % yield. R_f=0.35 (EtOAc/MeOH/Triethylamine 39:10:1). ^{1}H NMR (600 MHz, *CDCl3*) δ ppm 7.76-7.75 (m, $2H^{arom.}$, $C(C\underline{H}CH)_2CH$), 7.49-7.46 (m, $1H^{arom.}$, $C(CHCH)_2C\underline{H}$), 7.43-7.40 (m, $2H^{arom.}$, $C(CHC\underline{H})_2CH$), 7.24-7.23 (m, $2H^{phenyl.}$, $C(CHC\underline{H})_2CCl$), 7.12-7.10 (m, $2H^{phenyl.}$, $C(C\underline{H}CH)_2CCl$), 6.65 (br, 1H, N$\underline{H}$), 3.48 (dt, 2H, $C\underline{H}_2NH$), 3.43 (t, 2H, $OC\underline{H}_2CH_2CH_2N^{piperazine}$, *J* = 6.42Hz), 3.39 (t, 2H, $OC\underline{H}_2CH_2CH_2Ph$, *J* = 6.35Hz), 2.66 (t, 2H, $ClPhC\underline{H}_2$), 2.48 (br, $8H^{piperazine}$), 2.41 (t, 4H, $C\underline{H}_2N^{piperazine}$), 1.85 (qt, 2H, $C\underline{H}_2CH_2Ph$), 1.75 (qt, 2H, $OCH_2C\underline{H}_2CH_2N^{piperazine}$), 1.70-1.60 (m, 4H, $CH_2C\underline{H}_2CH_2$). ^{13}C NMR (150.95 MHz, *CDCl3*) δ ppm 167.68 ($1C^{quat.}$, C=O), 140.46 ($1C^{quat./phenyl}$, $\underline{C}CH_2$), 135.11 ($1C^{quat./arom.}$), 131.50 ($1C^{quat./phenyl.}$, $\underline{C}Cl$), 131.22 ($1C^{arom.}$, $C(CHCH)_2\underline{C}H$), 129.82 ($2C^{phenyl.}$, $C(\underline{C}HCH)_2CCl$), 128.49 ($2C^{arom.}$, $C(CH\underline{C}H)_2CH$), 128.40 ($2C^{phenyl.}$,

$C(CH\underline{C}H)_2CCl$), 126.95 ($2C^{arom.}$, $C(\underline{C}HCH)_2CH$), 69.68 (1C, $O\underline{C}H_2CH_2CH_2Ph$), 69.15 (1C, $OC\underline{H}_2CH_2CH_2N^{piperazine}$), 57.95 (1C, $\underline{C}H_2N^{piperazine}$), 55.48 (1C, $\underline{C}H_2N^{piperazine}$), 53.18 ($2C^{piperazine}$), 53.02 ($2C^{piperazine}$), 39.93 (1C, $\underline{C}H_2NH$), 31.72 (1C, $ClPh\underline{C}H_2$), 31.18 (1C, $PhCH_2\underline{C}H_2$), 27.49 (1C, $CH_2\underline{C}H_2CH_2$), 27.18 (1C, $CH_2\underline{C}H_2CH_2$), 24.42 (1C, $CH_2\underline{C}H_2CH_2$).

Preparation of *N*-{4-{4-{3-[3-(4-chlorophenyl)propoxy]propyl}piperazin-1-yl}butyl}-4-(trifluoromethyl)benzamide (**14d**)

4-(Trifluoromethyl)benzoyl chloride (0.20 g; $9.59 \cdot 10^{-4}$ mol) in 10 mL DCM was added dropwise to a solution of 4-{4-{3-[3-(4-chlorophenyl)propoxy]propyl}piperazin-1-yl}butan-1-amine (**13b**) (0.28 g; $7.61 \cdot 10^{-4}$ mol) and triethylamine (0.39 g; $3.85 \cdot 10^{-3}$ mol) in 15 mL DCM. The reaction was stirred for three hours at room temperature. The mixture was washed three-times with 15 mL water and dried over Na_2SO_4. The solvent was removed under vacuum and the crude product was purified by column chromatography (EtOAc/MeOH/Triethylamine 49:10:1) to yield the pure product.

N-{4-{4-{3-[3-(4-chlorophenyl)propoxy]propyl}piperazin-1-yl}butyl}-4-(trifluoromethyl)benzamide (**14d**): $C_{28}H_{37}ClF_3N_3O_2$. M=540.06. White solid. 87.35 % yield. R_f=0.26 (EtOAc/MeOH/Triethylamine 39:10:1). mp: 87.0-89.0 °C. ^1H NMR (600 MHz, *CDCl₃*) δ ppm 7.87-7.86 (m, $2H^{arom.}$), 7.67-7.66 (m, $2H^{arom.}$), 7.23-7.22 (m, $2H^{phenyl.}$, $C(CHC\underline{H})_2CCl$), 7.11-7.10 (m, $2H^{phenyl.}$, $C(C\underline{H}CH)_2CCl$), 7.07 (br, 1H, $N\underline{H}$), 3.48-3.45 (m, 2H, $C\underline{H}_2NH$), 3.42 (t, 2H, $OC\underline{H}_2CH_2CH_2N^{piperazine}$, J = 6.46Hz), 3.38 (t, 2H, $OC\underline{H}_2CH_2CH_2Ph$, J = 6.34Hz), 2.65 (t, 2H, $ClPhC\underline{H}_2$), 2.47 (br, $8H^{piperazine}$), 2.40 (t, 2H, $C\underline{H}_2N^{piperazine}$), 2.37 (t, 2H, $C\underline{H}_2N^{piperazine}$), 1.84 (qt, 2H, $C\underline{H}_2CH_2Ph$), 1.73 (qt, 2H, $OCH_2C\underline{H}_2CH_2N^{piperazine}$), 1.69-1.61 (m, 4H, $CH_2C\underline{H}_2CH_2$). ^{13}C NMR (150.95 MHz, *CDCl₃*) δ ppm 166.45 ($1C^{quat.}$, C=O), 140.42 ($1C^{quat./phenyl}$, $\underline{C}CH_2$), 138.45 ($1C^{quat./arom.}$), 133.26, 133.04 132.83, 132.61 ($1C^{quat./arom.}$, $\underline{C}CF_3$), 131.42 ($1C^{quat./phenyl.}$, $\underline{C}Cl$), 129.76 ($2C^{phenyl.}$, $C(\underline{C}HCH)_2CCl$), 128.33 ($2C^{phenyl.}$, $C(CH\underline{C}H)_2CCl$), 127.47 ($2C^{arom.}$, $C(\underline{C}HCH)_2CCF_3$), 126.39, 124.58, 122.78, 120.91 (1C, $\underline{C}F_3$), 125.47, 125.45 ($2C^{arom.}$, $C(CH\underline{C}H)_2CCF_3$), 69.63 (1C, $O\underline{C}H_2CH_2CH_2Ph$), 69.03 (1C, $OC\underline{H}_2CH_2CH_2N^{piperazine}$), 57.84 (1C, $\underline{C}H_2N^{piperazine}$), 55.38 (1C, $\underline{C}H_2N^{piperazine}$), 53.12 ($2C^{piperazine}$), 52.89 ($2C^{piperazine}$), 40.03 (1C, $\underline{C}H_2NH$), 31.65 (1C, $ClPh\underline{C}H_2$), 31.11 (1C, $PhCH_2\underline{C}H_2$), 27.31 (1C, $CH_2\underline{C}H_2CH_2$), 27.10 (1C, $CH_2\underline{C}H_2CH_2$), 24.34 (1C, $CH_2\underline{C}H_2CH_2$).

Preparation of *N*-benzyl-4-{4-[3-(3-phenylpropoxy)propyl]piperazin-1-yl}butan-1-amine (**15a**)

$LiAlH_4$ (0.11 g; $2.89 \cdot 10^{-3}$ mol) was added to a solution of *N*-{4-{4-[3-(3-phenylpropoxy)propyl]piperazin-1-yl}butyl}benzamide (**14a**) (0.321 g; $7.33 \cdot 10^{-4}$ mol) in 30 mL anhydrous diethyl ether. The reaction was stirred overnight at room temperature, then the mixture was quenched by dropwise addition of water (16 equiv.) and 10 % NaOH solution (16 equiv.) stirred for two hours, then filtered. The precipitate was discarded. The organic layer was dried over Na_2SO_4, then the solvent was removed under vacuum and the crude product was purified by column chromatography (DCM/MeOH/25% NH_3 aq. 89:10:1) to yield the pure product.

N-benzyl-4-{4-[3-(3-phenylpropoxy)propyl]piperazin-1-yl}butan-1-amine (**15a**): $C_{27}H_{41}N_3O$. M=423.63. Yellowish sticky oil. 78.85 % yield. R_f=0.81 (DCM/MeOH/25% NH_3 aq. 89:10:11). ^1H NMR (600 MHz, *CDCl₃*) δ ppm 7.33-7.31 (m, $4H^{arom.}$, $C(C\underline{H}C\underline{H})_2CH$), 7.29-7.23 (m, 3H: $1H^{arom.}$, $C(CHCH)_2C\underline{H}$; $2H^{phenyl.}$, $C(CHC\underline{H})_2CH$),

7.19-7.17 (m, $3H^{phenyl.}$, $C(C\underline{H}CH)_2C\underline{H}$), 3.80 (s, 2H, $NC\underline{H}_2Ph$), 3.44 (t, 2H, $OC\underline{H}_2CH_2CH_2N^{piperazine}$, J = 6.48Hz), 3.41 (t, 2H, $OC\underline{H}_2CH_2CH_2Ph$, J = 6.42Hz), 2.67 (m, 4H: $PhC\underline{H}_2$, $CH_2C\underline{H}_2NH$), 2.54-2.33 (m, 12H: $8H^{piperazine}$, $C\underline{H}_2N^{piperazine}$), 2.09 (br, 1H, $N\underline{H}$), 1.89 (qt, 2H, $C\underline{H}_2CH_2Ph$), 1.77 (qt, 2H, $OCH_2C\underline{H}_2CH_2N^{piperazine}$), 1.55-1.53 (m, 4H, $NH_2CH_2C\underline{H}_2C\underline{H}_2$). ^{13}C NMR (150.95 MHz, *CDCl₃*) δ ppm 141.98 ($1C^{quat./phenyl}$, $\underline{C}CH_2$), 140.12 ($1C^{quat./arom.}$), 128.44 ($2C^{phenyl.}$, $C(\underline{C}HCH)_2CH$), 128.38 ($2C^{phenyl.}$, $C(CH\underline{C}H)_2CH$), 128.27 ($2C^{arom.}$, $C(CH\underline{C}H)_2CH$), 128.14 ($2C^{arom.}$, $C(\underline{C}HCH)_2CH$), 126.91 ($1C^{arom.}$, $C(CHCH)_2\underline{C}H$), 125.71 ($1C^{phenyl.}$, $C(CHCH)_2\underline{C}H$), 69.89 (1C, $O\underline{C}H_2CH_2CH_2Ph$), 69.15 (1C, $OC\underline{H}_2CH_2CH_2N^{piperazine}$), 58.49 (1C, $NHCH_2CH_2CH_2\underline{C}H_2N^{piperazine}$), 55.53 (1C, $OCH_2CH_2\underline{C}H_2N^{piperazine}$), 53.92 (1C, $N\underline{C}H_2Ph$), 53.19 ($2C^{piperazine}$), 53.16 ($2C^{piperazine}$), 49.21 (1C, $CH_2\underline{C}H_2NH$), 32.31 (1C, $Ph\underline{C}H_2$), 31.26 (1C, $PhCH_2\underline{C}H_2$), 27.99, 24.69 (2C, $NH_2CH_2\underline{C}H_2\underline{C}H_2$), 27.17 (1C, $OCH_2\underline{C}H_2CH_2N^{piperazine}$).

Preparation of 4-{4-[3-(3-phenylpropoxy)propyl]piperazin-1-yl}-*N*-[4-(trifluoromethyl)benzyl]butan-1-amine (**15b**)

$LiAlH_4$ (0.16 g; $4.22\cdot10^{-3}$ mol) was added to a solution of *N*-{4-{4-[3-(3-phenylpropoxy)propyl]piperazin-1-yl}butyl}-4-(trifluoromethyl)benzamide (**14b**) (0.395 g; $7.81\cdot10^{-4}$ mol) in 30 mL anhydrous diethyl ether. The reaction was stirred overnight at room temperature, then the mixture was quenched by dropwise addition of water (16 equiv.) and 10 % NaOH solution (16 equiv.) stirred for two hours, then filtered. The precipitate was discarded. The organic layer was dried over Na_2SO_4, then the solvent was removed under vacuum and the crude product was purified by column chromatography (DCM/MeOH/25% NH_3 aq. 189:10:1) to yield the pure product.

4-{4-[3-(3-phenylpropoxy)propyl]piperazin-1-yl}-*N*-[4-(trifluoromethyl)benzyl]butan-1-amine (**15b**): $C_{28}H_{40}F_3N_3O$. M=491.63. Colourless sticky oil. 93.22 % yield. R_f=0.63 (DCM/MeOH/25% NH_3 aq. 189:10:11). ^1H NMR (600 MHz, *CDCl₃*) δ ppm 7.58-7.57 (m, $2H^{arom.}$, $C(CHC\underline{H})_2CCF_3$), 7.44-7.43 (m, $2H^{arom.}$, $C(C\underline{H}CH)_2C$ CF_3), 7.29-7.26 (m, $2H^{phenyl.}$, $C(CHC\underline{H})_2CH$), 7.19-7.18 (m, $3H^{phenyl.}$, $C(C\underline{H}CH)_2C\underline{H}$), 3.84 (s, 2H, $NC\underline{H}_2Ph$), 3.44 (t, 2H, $OC\underline{H}_2CH_2CH_2N^{piperazine}$, J = 6.48Hz), 3.41 (t, 2H, $OC\underline{H}_2CH_2CH_2Ph$, J = 6.42Hz), 2.68 (t, 2H, $PhC\underline{H}_2CH_2$, J = 7.80 Hz), 2.63 (t, 2H, $CH_2C\underline{H}_2NH$, J = 6.58 Hz), 2.58-2.32 (m, 12H: $8H^{piperazine}$, $C\underline{H}_2N^{piperazine}$), 1.91-1.82 (m, 3H: $PhCH_2C\underline{H}_2$, $N\underline{H}$), 1.77 (qt, 2H, $OCH_2C\underline{H}_2CH_2N^{piperazine}$), 1.53-1.51 (m, 4H, $NHCH_2C\underline{H}_2C\underline{H}_2$). ^{13}C NMR (150.95 MHz, *CDCl₃*) δ ppm 144.60 ($1C^{quat./arom.}$, $\underline{C}(CHCH)_2CH$), 142.00 ($1C^{quat./phenyl}$, $\underline{C}CH_2$), 129.53, 128.18, 126.77, 125.38 (1C, $\underline{C}F_3$), 129.46, 129.25, 129.03, 128.81 ($1C^{quat./arom.}$, $\underline{C}CF_3$), 128.46 ($2C^{phenyl.}$, $C(\underline{C}HCH)_2CH$), 128.29 ($2C^{phenyl.}$, $C(CH\underline{C}H)_2CH$), 128.23 ($2C^{arom.}$, $C(\underline{C}HCH)_2CCF_3$), 125.73 ($1C^{phenyl.}$, $C(CHCH)_2\underline{C}H$), 125.28,125.26 ($2C^{arom.}$, $C(CH\underline{C}H)_2CCF_3$), 69.92 (1C, $O\underline{C}H_2CH_2CH_2Ph$), 69.17 (1C, $OC\underline{H}_2CH_2CH_2N^{piperazine}$), 58.52 (1C, $NHCH_2CH_2CH_2\underline{C}H_2N^{piperazine}$), 55.59 (1C, $OCH_2CH_2\underline{C}H_2N^{piperazine}$), 53.47 (1C, $N\underline{C}H_2Ph$), 53.23 ($2C^{piperazine}$), 53.21 ($2C^{piperazine}$), 49.31 (1C, $CH_2\underline{C}H_2NH$), 32.33 (1C, $Ph\underline{C}H_2CH_2$), 31.28 (1C, $PhCH_2\underline{C}H_2$), 28.08, 24.68 (2C, $NH_2CH_2\underline{C}H_2\underline{C}H_2$), 27.18 (1C, $OCH_2\underline{C}H_2CH_2N^{piperazine}$).

Preparation of *N*-benzyl-4-{4-{3-[3-(4-chlorophenyl)propoxy]propyl}piperazin-1-yl}butan-1-amine (**15c**)

$LiAlH_4$ (0.10 g; $2.64\cdot10^{-3}$ mol) was added to a solution of *N*-{4-{4-{3-[3-(4-chlorophenyl)propoxy]propyl}piperazin-1-yl}butyl}benzamide (**14c**) (0.321 g; $6.79\cdot10^{-4}$ mol) in 30 mL anhydrous diethyl ether. The reaction was stirred overnight at room temperature, then the mixture was quenched by dropwise addition of water (16 equiv.) and 10 % NaOH solution (16 equiv.) stirred for two hours, then filtered. The precipitate was discarded. The organic layer was dried over Na_2SO_4, then the solvent was removed under vacuum and the crude product was purified by column chromatography (DCM/MeOH/25% NH_3 aq. 89:10:1) to yield the pure product.

N-benzyl-4-{4-{3-[3-(4-chlorophenyl)propoxy]propyl}piperazin-1-yl}butan-1-amine (**15c**): $C_{27}H_{40}ClN_3O$. M=458.08. Yellowish sticky oil. 72.23 % yield. R_f=0.67 (DCM/MeOH/25% NH_3 aq. 89:10:11). ^{1}H NMR (600 MHz, *CDCl₃*) δ ppm 7.33-7.30 (m, $4H^{arom.}$, $C(C\underline{H}C\underline{H})_2CH$), 7.26-7.24 (m, $1H^{arom.}$, $C(CHCH)_2C\underline{H}$), 7.23-7.22 (m, $2H^{phenyl.}$, $C(CHC\underline{H})_2CCl$), 7.11-7.09 (m, $2H^{phenyl.}$, $C(C\underline{H}CH)_2CCl$), 3.79 (s, 2H, $NC\underline{H}_2Ph$), 3.43 (t, 2H, $OC\underline{H}_2CH_2CH_2N^{piperazine}$, *J* = 6.47Hz), 3.38 (t, 2H, $OC\underline{H}_2CH_2CH_2Ph$, *J* = 6.33Hz), 2.66-2.63 (m, 4H, $CH_2C\underline{H}_2NH$, $ClPhC\underline{H}_2$), 2.46-2.33 (br, 9H: $8H^{piperazine}$, $N\underline{H}$), 2.41-2.39 (m, 2H, $OCH_2CH_2C\underline{H}_2N^{piperazine}$), 2.34 (t, 2H, $NCH_2CH_2C\underline{H}_2N^{piperazine}$, *J* = 7.11Hz), 1.85 (qt, 2H, $C\underline{H}_2CH_2Ph$), 1.75 (qt, 2H, $OCH_2C\underline{H}_2CH_2N^{piperazine}$), 1.55-1.52 (m, 4H, $CH_2C\underline{H}_2CH_2$). ^{13}C NMR (150.95 MHz, *CDCl₃*) δ ppm 140.41 ($1C^{quat./phenyl}$, $\underline{C}CH_2$), 140.06 ($1C^{quat./arom.}$), 131.42 ($1C^{quat./phenyl.}$, $\underline{C}Cl$), 129.76 ($2C^{phenyl.}$, $C(\underline{C}HCH)_2CCl$), 128.35 ($2C^{phenyl.}$, $C(CH\underline{C}H)_2CCl$), 128.33 ($2C^{arom.}$, $C(CH\underline{C}H)_2CH$), 128.11 ($1C^{arom.}$, $C(CHCH)_2\underline{C}H$), 126.92 ($2C^{arom.}$, $C(\underline{C}HCH)_2CH$), 69.59 (1C, $O\underline{C}H_2CH_2CH_2Ph$), 69.15 (1C, $OC\underline{H}_2CH_2CH_2N^{piperazine}$), 58.45 (1C, $\underline{C}H_2N^{piperazine}$), 55.47 (1C, $\underline{C}H_2N^{piperazine}$), 53.84 (1C, $N\underline{C}H_2Ph$), 53.19 ($2C^{piperazine}$), 53.16 ($2C^{piperazine}$), 49.16 (1C, $CH_2\underline{C}H_2NH$), 31.65 (1C, $ClPh\underline{C}H_2$), 31.13 (1C, $PhCH_2\underline{C}H_2$), 27.96 (1C, $CH_2\underline{C}H_2CH_2$), 27.17 (1C, $CH_2\underline{C}H_2CH_2$), 24.68 (1C, $CH_2\underline{C}H_2CH_2$).

Preparation of 4-{4-{3-[3-(4-chlorophenyl)propoxy]propyl}piperazin-1-yl}-*N*-[4-(trifluoromethyl)benzyl]butan-1-amine (**15d**)

$LiAlH_4$ (0.10 g; $2.64\cdot10^{-3}$ mol) was added to a solution of *N*-{4-{4-{3-[3-(4-chlorophenyl)propoxy]propyl}piperazin-1-yl}butyl}-4-(trifluoromethyl)benzamide (**14d**) (0.334 g; $6.18\cdot10^{-4}$ mol) in 30 mL anhydrous diethyl ether. The reaction was stirred overnight at room temperature, then the mixture was quenched by dropwise addition of water (16 equiv.) and 10 % NaOH solution (16 equiv.) stirred for two hours, then filtered. The precipitate was discarded. The organic layer was dried over Na_2SO_4, then the solvent was removed under vacuum and the crude product was purified by column chromatography (DCM/MeOH/25% NH_3 aq. 89:10:1) to yield the pure product.

4-{4-{3-[3-(4-chlorophenyl)propoxy]propyl}piperazin-1-yl}-*N*-[4-(trifluoromethyl)benzyl]butan-1-amine (**15d**): $C_{28}H_{39}ClF_3N_3O$. M=526.08. Yellowish sticky oil. 72.0 % yield. R_f=0.63 (DCM/MeOH/25% NH_3 aq. 89:10:1). ^{1}H NMR (600 MHz, *CDCl₃*) δ ppm 7.57-7.56 (m, $2H^{arom.}$), 7.44-7.43 (m, $2H^{arom.}$), 7.24-7.22 (m, $2H^{phenyl.}$, $C(CHC\underline{H})_2CCl$), 7.11-7.10 (m, $2H^{phenyl.}$, $C(C\underline{H}CH)_2CCl$), 3.84 (s, 2H, $NC\underline{H}_2Ph$), 3.43 (t, 2H, $OC\underline{H}_2CH_2CH_2N^{piperazine}$, *J* = 6.47Hz), 3.38 (t, 2H, $OC\underline{H}_2CH_2CH_2Ph$, *J* = 6.32Hz), 2.66-2.62 (m, 4H, $CH_2C\underline{H}_2NH$, $ClPhC\underline{H}_2$), 2.46 (br, $8H^{piperazine}$), 2.41 (t, 2H, $C\underline{H}_2N^{piperazine}$), 2.34 (t, 2H, $C\underline{H}_2N^{piperazine}$, *J* = 7.13Hz), 1.85 (qt, 2H, $C\underline{H}_2CH_2Ph$), 1.76 (qt, 2H, $OCH_2C\underline{H}_2CH_2N^{piperazine}$), 1.63-1.26 (m, 5H, $CH_2C\underline{H}_2CH_2$, $N\underline{H}$). ^{13}C NMR (150.95 MHz, *CDCl₃*) δ ppm 144.74 ($1C^{quat./arom.}$, $\underline{C}(CHCH)_2CH$), 140.59 ($1C^{quat./phenyl}$, $\underline{C}CH_2$), 131.46 ($1C^{quat./phenyl.}$, $\underline{C}Cl$), 129.79

($2C^{phenyl.}$, $C(\underline{C}HCH)_2CCl$), 129.49, 129.28, 129.07, 128.85 ($1C^{quat./arom.}$, $\underline{C}CF_3$), 128.36 ($2C^{phenyl.}$, $C(CH\underline{C}H)_2CCl$), 128.21 ($2C^{arom.}$, $C(\underline{C}HCH)_2CH$), 126.97, 125.17, 123.37, 121.57 (1C, $\underline{C}F_3$), 125.52, 125.23 ($2C^{arom.}$, $C(CH\underline{C}H)_2CCF_3$), 69.63 (1C, $O\underline{C}H_2CH_2CH_2Ph$), 69.19 (1C, $OC\underline{H}_2CH_2CH_2N^{piperazine}$), 58.52 (1C, $\underline{C}H_2N^{piperazine}$), 55.54 (1C, $\underline{C}H_2N^{piperazine}$), 53.45 (1C, $N\underline{C}H_2Ph$), 53.29 ($2C^{piperazine}$), 53.29 ($2C^{piperazine}$), 49.33 (1C, $CH_2\underline{C}H_2NH$), 31.69 (1C, $ClPh\underline{C}H_2$), 31.16 (1C, $PhCH_2\underline{C}H_2$), 28.12 (1C, $CH_2\underline{C}H_2CH_2$), 27.22 (1C, $CH_2\underline{C}H_2CH_2$), 24.71 (1C, $CH_2\underline{C}H_2CH_2$).

Preparation of 1-(benzyl)-2,3-di(*tert*-butoxycarbonyl)-1-{4-{4-[3-(3-phenylpropoxy)prop-1-yl]piperazin-1-yl}but-1-yl}guanidine (**16a**)

1,3-bis(*tert*-butoxycarbonyl)-2-methylisothiourea (0.178 g; $6.13\cdot10^{-4}$ mol) and mercury II chloride (0.166 g; $6.11\cdot10^{-4}$ mol) were sequentially added to an ice-cooled mixture of *N*-benzyl-4-{4-[3-(3-phenylpropoxy)propyl]piperazin-1-yl}butan-1-amine (**15a**) (0.236 g; $5.57\cdot10^{-4}$ mol) and triethylamine (0.28 g; $2.77\cdot10^{-3}$ mol) in 25 mL DCM. The ice bath was removed and the reaction was stirred for eighteen hours at room temperature, then filtered. The precipitate was discarded. The filtrate was washed sequentially twice with 15 mL H_2O and twice with 15 mL brine. The combined organic phases were dried over Na_2SO_4, then the solvent was removed under vacuum and the crude product was purified by column chromatography (EtOAc/MeOH/Triethylamine 89:10:1) to yield the pure product.

1-(benzyl)-2,3-di(*tert*-butoxycarbonyl)-1-{4-{4-[3-(3-phenylpropoxy)prop-1-yl]piperazin-1-yl}but-1-yl}guanidine (**16a**): $C_{38}H_{59}N_5O_5$. M=665.91. Colourless sticky oil. 66.06 % yield. R_f=0.39 (EtOAc/MeOH/Triethylamine 89:10:1). ^1H NMR (600 MHz, *CDCl₃*) δ ppm 9.97 (br, 1H, $N\underline{H}$), 7.34-7.31 (m, $2H^{arom.}$, $C(CHC\underline{H})_2CH$), 7.29-7.25 (m, 5H: $3H^{arom.}$, $C(C\underline{H}CH)_2C\underline{H}$; $2H^{phenyl.}$, $C(CHC\underline{H})_2CH$), 7.19-7.17 (m, $3H^{phenyl.}$, $C(C\underline{H}CH)_2C\underline{H}$), 4.65 (br, 2H, $NC\underline{H}_2Ph$), 3.44 (t, 2H, $OC\underline{H}_2CH_2CH_2N^{piperazine}$, J = 6.48Hz), 3.41 (t, 2H, $OC\underline{H}_2CH_2CH_2Ph$, J = 6.42Hz), 3.35 (br, 2H, $CH_2C\underline{H}_2NC(N)$), 2.69-2.67 (m, 2H, $PhC\underline{H}_2$, J = 7.20Hz), 2.60-2.33 (m, 10H: $8H^{piperazine}$, $OCH_2CH_2C\underline{H}_2N^{piperazine}$), 2.28 (t, 2H, $NCH_2CH_2CH_2\underline{C}H_2N^{piperazine}$, J = 7.50Hz), 1.91-1.86 (m, 2H, $C\underline{H}_2CH_2Ph$), 1.77 (qt, 2H, $OCH_2C\underline{H}_2CH_2N^{piperazine}$), 1.59-1.53 (m, 2H, $NCH_2C\underline{H}_2CH_2$), 1.49 (m, 18H, $C\underline{H}_3$), 1.46-1.31 (m, 2H, $NCH_2CH_2C\underline{H}_2$). ^{13}C NMR (150.95 MHz, *CDCl₃*) δ ppm 162.70 (1C, $\underline{C}$=O), 156.02 ($1C^{quat.}$, $\underline{C}$=N), 150.93 (1C, $\underline{C}$=O), 141.99 ($1C^{quat./phenyl}$, $\underline{C}CH_2$), 136.36 ($1C^{quat./arom.}$), 128.63 ($2C^{arom.}$, $C(CH\underline{C}H)_2CH$), 128.45 ($2C^{phenyl.}$, $C(\underline{C}HCH)_2CH$), 128.28 ($2C^{phenyl.}$, $C(CH\underline{C}H)_2CH$), 127.85 ($2C^{arom.}$, $C(\underline{C}HCH)_2CH$), 127.56 ($1C^{arom.}$, $C(CHCH)_2\underline{C}H$), 125.72 ($1C^{phenyl.}$, $C(CHCH)_2\underline{C}H$), 81.95 ($1C^{quat.}$ Boc), 79.39 ($1C^{quat.}$ Boc), 69.90 (1C, $O\underline{C}H_2CH_2CH_2Ph$), 69.17 (1C, $OC\underline{H}_2CH_2CH_2N^{piperazine}$), 58.01 (1C, $NCH_2CH_2CH_2\underline{C}H_2N^{piperazine}$), 55.56 (1C, $OCH_2CH_2\underline{C}H_2N^{piperazine}$), 53.17 ($2C^{piperazine}$), 53.11 ($2C^{piperazine}$), 51.62 (1C, $N\underline{C}H_2Ph$), 47.33 (1C, $\underline{C}H_2C(N)N$), 32.32 (1C, $Ph\underline{C}H_2$), 31.27 (1C, $PhCH_2\underline{C}H_2$), 28.20, 28.14 (6C, $\underline{C}H_3$), 27.16 (1C, $OCH_2\underline{C}H_2CH_2N^{piperazine}$), 25.07 (1C, $NCH_2\underline{C}H_2CH_2$ $CH_2N^{piperazine}$), 23.82 (1C, $NH_2CH_2\underline{C}H_2\underline{C}H_2CH_2N^{piperazine}$).

Preparation of 2,3-di(*tert*-butoxycarbonyl)-1-{4-{4-[3-(3-phenylpropoxy)prop-1-yl]piperazin-1-yl}but-1-yl}-1-[4-(trifluoromethyl)benzyl]guanidine (**16b**)

1,3-bis(*tert*-butoxycarbonyl)-2-methylisothiourea (0.214 g; $7.37 \cdot 10^{-4}$ mol) and mercury II chloride (0.200 g; $7.37 \cdot 10^{-4}$ mol) were sequentially added to an ice-cooled mixture of 4-{4-[3-(3-phenylpropoxy)propyl]piperazin-1-yl}-*N*-[4-(trifluoromethyl)benzyl]butan-1-amine (**15b**) (0.330 g; $6.71 \cdot 10^{-4}$ mol) and triethylamine (0.338 g; $3.34 \cdot 10^{-3}$ mol) in 30 mL DCM. The ice bath was removed and the reaction was stirred for eighteen hours at room temperature, then filtered. The precipitate was discarded. The filtrate was washed sequentially twice with 15 mL H_2O and twice with 15 mL brine. The combined organic phases were dried over Na_2SO_4, then the solvent was removed under vacuum and the crude product was purified by column chromatography (EtOAc/MeOH/Triethylamine 89:10:1) to yield the pure product.

2,3-di(*tert*-butoxycarbonyl)-1-{4-{4-[3-(3-phenylpropoxy)prop-1-yl]piperazin-1-yl}but-1-yl}-1-[4-(trifluoromethyl)benzyl]guanidine (**16b**): $C_{39}H_{58}F_3N_5O_5$. M=733.90. Colourless sticky oil. 65.57 % yield. R_f=0.45 (EtOAc/MeOH/Triethylamine 89:10:1). ^{1}H NMR (600 MHz, *CDCl₃*) δ ppm 9.99 (br, 1H, $N\underline{H}$), 7.60-7.59 (m, $2H^{arom.}$, $C(CHC\underline{H})_2CCF_3$), 7.42-7.41 (m, $2H^{arom.}$, $C(C\underline{H}CH)_2CCF_3$), 7.29-7.26 (m, $2H^{phenyl.}$, $C(CHC\underline{H})_2CH$), 7.19-7.17 (m, $3H^{phenyl.}$, $C(C\underline{H}CH)_2C\underline{H}$), 4.76 (s, 2H, $NC\underline{H}_2Ph$), 3.44 (t, 2H, $OC\underline{H}_2CH_2CH_2N^{piperazine}$, J = 6.44Hz), 3.41 (t, 2H, $OC\underline{H}_2CH_2CH_2Ph$, J = 6.42Hz), 3.33 (br, 2H, $CH_2C\underline{H}_2NC(N)$), 2.68 (t, 2H, $PhC\underline{H}_2CH_2$, J = 7.80 Hz), 2.57-2.36 (m, 10H: $8H^{piperazine}$, $OCH_2CH_2C\underline{H}_2N^{piperazine}$), 2.30 (t, 2H, $NCH_2CH_2CH_2\underline{C}H_2N^{piperazine}$, J = 7.50Hz), 1.89 (qt, 2H: $PhCH_2C\underline{H}_2$), 1.78 (qt, 2H, $OCH_2C\underline{H}_2CH_2N^{piperazine}$), 1.59-1.54 (m, 2H, $NCH_2C\underline{H}_2CH_2$), 1.49 (m, 18H, $C\underline{H}_3$), 1.46-1.40 (m, 2H, $NCH_2CH_2C\underline{H}_2$). ^{13}C NMR (150.95 MHz, *CDCl₃*) δ ppm 171.16 (1C, $\underline{C}$=O), 161.03 (1C, $\underline{C}$=O), 156.20 ($1C^{quat.}$, $\underline{C}$=N), 141.98 ($1C^{quat./phenyl}$, $\underline{C}CH_2$), 140.64 ($1C^{quat./arom.}$, $\underline{C}(CHCH)_2CH$), 130.09, 129.83, 129.62, 129.39 ($1C^{quat./arom.}$, $\underline{C}CF_3$), 128.45 ($2C^{phenyl.}$, $C(\underline{C}HCH)_2CH$), 128.28 ($2C^{phenyl.}$, $C(CH\underline{C}H)_2CH$), 127.97 ($2C^{arom.}$, $C(\underline{C}HCH)_2CCF_3$), 126.80, 124.97, 123.17, 121.47 (1C, $\underline{C}F_3$), 125.73 ($1C^{phenyl.}$, $C(CHCH)_2\underline{C}H$), 125.59,125.56 ($2C^{arom.}$, $C(CH\underline{C}H)_2CCF_3$), 82.16 (1 $C^{quat.}$ Boc), 79.24 (1 $C^{quat.}$ Boc), 69.92 (1C, $O\underline{C}H_2CH_2CH_2Ph$), 69.09 (1C, $O\underline{C}H_2CH_2CH_2N^{piperazine}$), 57.86 (1C, $NHCH_2CH_2CH_2\underline{C}H_2N^{piperazine}$), 55.52 (1C, $OCH_2CH_2\underline{C}H_2N^{piperazine}$), 53.04 ($2C^{piperazine}$), 52.99 ($2C^{piperazine}$), 50.93 (1C, $N\underline{C}H_2Ph$), 48.12 (1C, $CH_2\underline{C}H_2NC(N)$), 32.31 (1C, $Ph\underline{C}H_2CH_2$), 31.26 (1C, $PhCH_2\underline{C}H_2$), 28.14 (6C, $\underline{C}H_3$), 27.07 (1C, $OCH_2\underline{C}H_2CH_2N^{piperazine}$), 25.16 (1C, $NCH_2\underline{C}H_2CH_2$), 23.71 (1C, $NCH_2CH_2\underline{C}H_2$).

Preparation of 1-(benzyl)-1-{4-{4-{3-[3-(4-chlorophenyl)propoxy]prop-1-yl}piperazin-1-yl}but-1-yl}-2,3-di(*tert*-butoxycarbonyl)guanidine (**16c**)

1,3-bis(*tert*-butoxycarbonyl)-2-methylisothiourea (0.153 g; $5.27 \cdot 10^{-4}$ mol) and mercury II chloride (0.14 g; $5.16 \cdot 10^{-4}$ mol) were sequentially added to an ice-cooled mixture of *N*-benzyl-4-{4-{3-[3-(4-chlorophenyl)propoxy]propyl}piperazin-1-yl}butan-1-amine (**15c**) (0.22 g; $4.80 \cdot 10^{-4}$ mol) and triethylamine (0.24 g; $2.37 \cdot 10^{-3}$ mol) in 30 mL DCM. The ice bath was removed and the reaction was stirred for eighteen hours at room temperature, then filtered. The precipitate was discarded. The filtrate was washed sequentially twice with 15 mL H_2O and twice with 15 mL brine. The combined organic phases were dried over Na_2SO_4, then the solvent was removed under vacuum and the crude product was purified by column chromatography (EtOAc/MeOH/Triethylamine 89:10:1) to yield the pure product.

1-(benzyl)-1-{4-{4-{3-[3-(4-chlorophenyl)propoxy]prop-1-yl}piperazin-1-yl}but-1-yl}-2,3-di(*tert*-butoxycarbonyl)guanidine (**16c**): $C_{38}H_{58}ClN_5O_5$. M=700.35. Colourless sticky oil. 68.45 % yield. R_f=0.39

(EtOAc/MeOH/Triethylamine 89:10:1). ^{1}H NMR (600 MHz, *CDCl₃)* δ ppm 9.91 (br, 1H, N$\underline{H}$), 7.33-7.22 (m, 7H: $5H^{arom.}$, $C(C\underline{H}C\underline{H})_2C\underline{H}$; $2H^{phenyl.}$, $C(CHC\underline{H})_2CCl$), 7.11-7.09 (m, $2H^{phenyl.}$, $C(C\underline{H}CH)_2CCl$), 4.66 (br, 2H, $NC\underline{H}_2Ph$), 3.43 (t, 2H, $OC\underline{H}_2CH_2CH_2N^{piperazine}$, J = 6.43Hz), 3.38 (t, 2H, $OC\underline{H}_2CH_2CH_2Ph$, J = 6.34Hz), 3.35 (br, 2H, $CH_2C\underline{H}_2NC(N)$), 2.65 (t, 2H, $ClPhC\underline{H}_2$), 2.45-2.39 (br, 10H: $8H^{piperazine}$, $OCH_2CH_2\underline{C}H_2N^{piperazine}$), 2.28 (t, 2H, $NCH_2CH_2CH_2\underline{C}H_2N^{piperazine}$), 1.85 (qt, 2H, $PhCH_2C\underline{H}_2$), 1.76 (qt, 2H, $OCH_2C\underline{H}_2CH_2N^{piperazine}$), 1.57 (qt, 2H, $CH_2C\underline{H}_2CH_2$), 1.49 (m, 18H, $C\underline{H}_3$), 1.43-1.39 (m, 2H, $CH_2C\underline{H}_2CH_2$). ^{13}C NMR (150.95 MHz, *CDCl₃)* δ ppm 162.71 (1C, $\underline{C}$=O), 155.94 ($1C^{quat.}$, $\underline{C}$=N), 150.95 (1C, $\underline{C}$=O), 140.43 ($1C^{quat./phenyl}$, $\underline{C}CH_2$), 136.43 ($1C^{quat./arom.}$), 131.45 ($1C^{quat./phenyl.}$, $\underline{C}Cl$), 129.79 ($2C^{phenyl.}$, $C(\underline{C}HCH)_2CCl$), 128.61 ($2C^{arom.}$, $C(CH\underline{C}H)_2CH$), 128.36 ($2C^{phenyl.}$, $C(CH\underline{C}H)_2CCl$), 127.87 ($2C^{arom.}$, $C(\underline{C}HCH)_2CH$), 127.53 ($1C^{arom.}$, $C(CHCH)_2\underline{C}H$), 81.88 ($1C^{quat.}$ Boc), 79.34 ($1C^{quat.}$ Boc), 69.63 (1C, $O\underline{C}H_2CH_2CH_2Ph$), 69.18 (1C, $OC\underline{H}_2CH_2CH_2N^{piperazine}$), 57.97 (1C, $\underline{C}H_2N^{piperazine}$), 55.53 (1C, $\underline{C}H_2N^{piperazine}$), 53.20 ($2C^{piperazine}$), 53.11 ($2C^{piperazine}$), 51.78 (1C, $N\underline{C}H_2Ph$), 47.47 (1C, $\underline{C}H_2C(N)N$), 31.68 (1C, $ClPh\underline{C}H_2$), 31.16 (1C, $PhCH_2\underline{C}H_2$), 28.18 (6C, $\underline{C}H_3$), 27.18 (1C, $OCH_2\underline{C}H_2CH_2N^{piperazine}$), 25.10 (1C, $CH_2\underline{C}H_2CH_2$), 23.85 (1C, $CH_2\underline{C}H_2CH_2$).

Preparation of 1-{4-{4-{3-[3-(4-chlorophenyl)propoxy]propyl}piperazin-1-yl}but-1-yl}-2,3-di(*tert*-butoxycarbonyl)-1-(4-(trifluoromethyl)benzyl)guanidine (**16d**)

1,3-bis(*tert*-butoxycarbonyl)-2-methylisothiourea (0.138 g; $4.75\cdot10^{-4}$ mol) and mercury II chloride (0.129 g; $4.75\cdot10^{-4}$ mol) were sequentially added to an ice-cooled mixture of 4-{4-{3-[3-(4-chlorophenyl)propoxy]propyl}piperazin-1-yl}-*N*-[4-(trifluoromethyl)benzyl]butan-1-amine (**15d**) (0.228 g; $4.33\cdot10^{-4}$ mol) and triethylamine (0.,219 g; $2.16\cdot10^{-3}$ mol) in 40 mL DCM. The ice bath was removed and the reaction was stirred for eighteen hours at room temperature, then filtered. The precipitate was discarded. The filtrate was washed sequentially twice with 15 mL H_2O and twice with 15 mL brine. The combined organic phases were dried over Na_2SO_4, then the solvent was removed under vacuum and the crude product was purified by column chromatography (EtOAc/MeOH/Triethylamine 89:10:1) to yield the pure product.

1-{4-{4-{3-[3-(4-chlorophenyl)propoxy]propyl}piperazin-1-yl}but-1-yl}-2,3-di(*tert*-butoxycarbonyl)-1-(4-(trifluoromethyl)benzyl)guanidine (**16d**): $C_{39}H_{57}ClF_3N_5O_5$. M=768.35. Yellowish sticky oil. 78.40 % yield. R_f=0.43 (EtOAc/MeOH/Triethylamine 89:10:1). ^{1}H NMR (600 MHz, *CDCl₃)* δ ppm 9.95 (br, 1H, N$\underline{H}$), 7.59-7.58 (m, $2H^{arom.}$), 7.43-7.41 (m, $2H^{arom.}$), 7.24-7.22 (m, $2H^{phenyl.}$, $C(CHC\underline{H})_2CCl$), 7.11-7.10 (m, $2H^{phenyl.}$, $C(C\underline{H}CH)_2CCl$), 4.76 (br, 2H, $NC\underline{H}_2Ph$), 3.43 (t, 2H, $OC\underline{H}_2CH_2CH_2N^{piperazine}$, J = 6.48Hz), 3.38 (t, 2H, $OC\underline{H}_2CH_2CH_2Ph$, J = 6.33Hz), 3.33 (br, 2H, $CH_2C\underline{H}_2NC(N)$), 2.65 (t, 2H, $ClPhC\underline{H}_2$), 2.49-2.39 (br, 10H: $8H^{piperazine}$, $OCH_2CH_2\underline{C}H_2N^{piperazine}$), 2.28 (t, 2H, $NCH_2CH_2CH_2\underline{C}H_2N^{piperazine}$), 1.85 (qt, 2H, $PhCH_2C\underline{H}_2$), 1.76 (qt, 2H, $OCH_2C\underline{H}_2CH_2N^{piperazine}$), 1.57 (qt, 2H, $CH_2C\underline{H}_2CH_2$), 1.49 (m, 18H, $C\underline{H}_3$), 1.43-1.41 (m, 2H, $CH_2C\underline{H}_2CH_2$). ^{13}C NMR (150.95 MHz, *CDCl₃)* δ ppm 162.52 (1C, $\underline{C}$=O), 156.13 ($1C^{quat.}$, $\underline{C}$=N), 150.92 (1C, $\underline{C}$=O), 140.75 ($1C^{quat./arom.}$, $\underline{C}(CHCH)_2CH$), 140.44 ($1C^{quat./phenyl}$, $\underline{C}CH_2$), 131.45 ($1C^{quat./phenyl.}$, $\underline{C}Cl$), 130.08, 129.85, 129.64, 129.43 ($1C^{quat./arom.}$, $\underline{C}CF_3$), 129.79 ($2C^{phenyl.}$, $C(\underline{C}HCH)_2CCl$), 128.36 ($2C^{phenyl.}$, $C(CH\underline{C}H)_2CCl$), 128.00 ($2C^{arom.}$, $C(\underline{C}HCH)_2CH$), 126.80, 124.99, 123.19, 121.39 (1C, $\underline{C}F_3$), 125.57, 125.54 ($2C^{arom.}$, $C(CH\underline{C}H)_2CCF_3$), 82.07 ($1C^{quat.}$ Boc), 79.62 ($1C^{quat.}$ Boc), 69.63 (1C, $O\underline{C}H_2CH_2CH_2Ph$), 69.16 (1C, $OC\underline{H}_2CH_2CH_2N^{piperazine}$), 57.87 (1C, $\underline{C}H_2N^{piperazine}$), 55.51 (1C, $\underline{C}H_2N^{piperazine}$), 53.18 ($2C^{piperazine}$), 53.11 ($2C^{piperazine}$), 50.91 (1C, $N\underline{C}H_2Ph$), 48.12 (1C,

$\underline{C}H_2C(N)N$), 31.68 (1C, $ClPh\underline{C}H_2$), 31.16 (1C, $PhCH_2\underline{C}H_2$), 28.16 (6C, $\underline{C}H_3$), 27.17 (1C, $OCH_2\underline{C}H_2CH_2N^{piperazine}$), 25.22 (1C, $CH_2\underline{C}H_2CH_2$), 23.84 (1C, $CH_2\underline{C}H_2CH_2$).

Preparation of 1-(benzyl)-1-{4-{4-[3-(3-phenylpropoxy)prop-1-yl]piperazin-1-yl}but-1-yl}guanidine trihydrochloride (**ADS10349**)

4M solution HCl-dioxan (1.87 mL; $7.48 \cdot 10^{-3}$ mol) was added dropwise to a solution of the 1-(benzyl)-2,3-di(*tert*-butoxycarbonyl)-1-{4-{4-[3-(3-phenylpropoxy)prop-1-yl]piperazin-1-yl}but-1-yl}guanidine (**16a**) (0.245 g; $3.68 \cdot 10^{-4}$ mol) in 20 mL chloroform. The reaction was stirred overnight at room temperature, then the solvent was removed under vacuum. The crude product was evaporated twice from chloroform and twice from EtOAc, then recrystallized from anhydrous 2-propanol to yield the pure product.

1-(benzyl)-1-{4-{4-[3-(3-phenylpropoxy)prop-1-yl]piperazin-1-yl}but-1-yl}guanidine trihydrochloride (**ADS10349**): $C_{28}H_{43}N_5O \cdot 3HCl \cdot 1.5H_2O$. M=602.08. White solid. 58.69 %. mp:174-176 °C. ^1H NMR (600 MHz, *CD_3OD*) δ ppm 7.45-7.42 (m, $2H^{arom.}$, $C(CHC\underline{H})_2CH$), 7.38-7.35 (m, $1H^{arom.}$, $C(CHCH)_2C\underline{H}$), 7.31-7.27 (m, 4H: $2H^{phenyl.}$, $C(CHC\underline{H})_2CH$; $2H^{arom.}$, $C(C\underline{H}CH)_2CH$), 7.22-7.21 (m, $2H^{phenyl.}$, $C(C\underline{H}CH)_2CH$), 7.19-7.17 (m, $1H^{phenyl.}$, $C(CHCH)_2C\underline{H}$), 4.71 (br, 2H, $NC\underline{H}_2Ph$), 3.86 (br, $4H^{piperazine}$), 3.71-3.57 (br, 6H: $4H^{piperazine}$; $OC\underline{H}_2$), 3.50 (t, 2H, $OC\underline{H}_2$, *J*=7.50Hz), 3.45 (t, 2H, $CH_2C\underline{H}_2NC(N)$; *J*=7.50Hz), 3.39 (t, 2H, $C\underline{H}_2N^{piperazine}$, *J*=7.50Hz), 3.28 (m, 2H, $C\underline{H}_2N^{piperazine}$), 2.71 (t, 2H, $PhC\underline{H}_2CH_2$, *J*=7.62Hz), 2.12-2.08 (m, 2H, $CH_2C\underline{H}_2CH_2$), 1.94-1.89 (qt, 2H, $CH_2C\underline{H}_2CH_2$), 1.83-1.75 (m, 4H, $CH_2C\underline{H}_2CH_2$). ^{13}C NMR (150.95 MHz, *CD_3OD*) δ ppm 158.41 (1C, $\underline{C}$=N), 143.25 ($1C^{quat./phenyl}$, $\underline{C}CH_2$), 136.31 ($1C^{quat./arom.}$, $\underline{C}(CHCH)_2C$), 130.16 ($2C^{arom.}$, $C(CH\underline{C}H)_2CH$), 129.49 ($2C^{phenyl.}$, $C(\underline{C}HCH)_2CH$), 129.42 ($2C^{phenyl.}$, $C(CH\underline{C}H)_2CH$), 129.22 ($1C^{arom.}$, $C(CHCH)_2\underline{C}H$), 128.02 ($2C^{arom.}$, $C(\underline{C}HCH)_2CH$), 126.91 ($1C^{phenyl.}$, $C(CHCH)_2\underline{C}H$), 71.49 (1C, $O\underline{C}H_2$), 68.42 (1C, $O\underline{C}H_2$), 57.79 (1C, $N\underline{C}H_2Ph$), 57.52 (1C, $\underline{C}H_2N^{piperazine}$), 56.31 (1C, $\underline{C}H_2N^{piperazine}$), 49.91 ($4C^{piperazine}$), 49.62 (1C, $CH_2\underline{C}H_2NC(N)$), 33.39 (1C, $Ph\underline{C}H_2CH_2$), 32.46 (1C, $CH_2\underline{C}H_2CH_2$), 25.61 (1C, $CH_2\underline{C}H_2CH_2$), 25.30 (1C, $CH_2\underline{C}H_2CH_2$), 22.14 (1C, $CH_2\underline{C}H_2CH_2$). Anal. Calcd: C 55.86 %; H 8.20 %; N 11.63 %. Found: C 55.75 %; H 8.53 %; N 11.65%.

Preparation of 1-{4-{4-[3-(3-phenylpropoxy)prop-1-yl]piperazin-1-yl}but-1-yl}-1-(4-(trifluoromethyl)benzyl)guanidine trihydrochloride (**ADS10350**)

4M solution HCl-dioxan (1.92 mL; $7.68 \cdot 10^{-3}$ mol) was added dropwise to a solution of the 2,3-di(*tert*-butoxycarbonyl)-1-{4-{4-[3-(3-phenylpropoxy)prop-1-yl]piperazin-1-yl}but-1-yl}-1-(4-(trifluoromethyl)benzyl)guanidine trihydrochloride (**16b**) (0.282 g; $3.84 \cdot 10^{-4}$ mol) in 20 mL chloroform. The reaction was stirred overnight at room temperature, then the solvent was removed under vacuum. The crude product was evaporated twice from chloroform and twice from EtOAc. The residue was stirred twice with EtOAc at 70 °C, then the supernatant was carefully decanted from the precipitate. The precipitate was evaporated and dried to yield the pure product.

1-{4-{4-[3-(3-phenylpropoxy)prop-1-yl]piperazin-1-yl}but-1-yl}-1-(4-(trifluoromethyl)benzyl)guanidine trihydrochloride (**ADS10350**): $C_{29}H_{42}F_3N_5O \cdot 3HCl \cdot 0.5H_2O$. M=652.06. White solid. 67.46 %. mp: 211.8-213.2 °C. ^1H NMR (600 MHz, *CD_3OD*) δ ppm 7.76-7.75 (m, $2H^{arom.}$, $C(CHC\underline{H})_2CCF_3$), 7.52-7.50 (m, $2H^{arom.}$,

$C(C\underline{H}CH)_2CCF_3$), 7.30-7.28 (m, $2H^{phenyl.}$, $C(CHC\underline{H})_2CH$), 7.23-7.22 (m, $2H^{phenyl.}$, $C(C\underline{H}CH)_2CH$), 7.20-7.18 (m, $1H^{phenyl.}$, $C(CHCH)_2C\underline{H}$), 4.80 (br, 2H, $NC\underline{H}_2Ph$), 3.92 (br, $4H^{piperazine}$), 3.68 (br, $4H^{piperazine}$), 3.60-3.58 (t, 2H, $OC\underline{H}_2$, *J*=5.60Hz), 3.52-3.49 (m, 4H, $OC\underline{H}_2$; $CH_2C\underline{H}_2NC(N)$), 3.41-3.39 (m, 2H, $C\underline{H}_2N^{piperazine}$), 3.34-3.33 (t, 2H, $C\underline{H}_2N^{piperazine}$; *J*=5.60Hz), 2.73-2.70 (t, 2H, $PhC\underline{H}_2CH_2$, *J*=7.50Hz), 2.14-2.10 (m, 2H, $CH_2C\underline{H}_2CH_2$), 1.95-1.83 (m, 6H, $CH_2C\underline{H}_2CH_2$). ^{13}C NMR (150.95 MHz, *CD₃OD*) δ ppm 158.53 (1C, $\underline{C}$=N), 143.26 ($1C^{quat./phenyl}$, $\underline{C}CH_2$), 141.07 ($1C^{quat./arom.}$, $\underline{C}(CHCH)_2CCF_3$), 131.56, 131.35, 131.14, 130.92 ($1C^{quat./arom.}$, $\underline{C}CF_3$), 129.50 ($2C^{phenyl.}$, $C(\underline{C}HCH)_2CH$), 129.42 ($2C^{phenyl.}$, $C(CH\underline{C}H)_2CH$), 128.46 ($2C^{arom.}$, $C(\underline{C}HCH)_2CCF_3$), 128.28, 126.48, 124.68, 122.88 (1C, $\underline{C}F_3$), 126.98, 126.96 ($2C^{arom.}$, $C(CH\underline{C}H)_2CCF_3$), 126.89 ($1C^{phenyl.}$, $C(CHCH)_2\underline{C}H$), 71.48 (1C, $O\underline{C}H_2$), 68.39 (1C, $O\underline{C}H_2$), 57.52 (1C, $\underline{C}H_2N^{piperazine}$), 56.30 (1C, $\underline{C}H_2N^{piperazine}$), 52.42 (1C, $N\underline{C}H_2Ph$), 49.96 ($4C^{piperazine}$), 49.74 (1C, $CH_2\underline{C}H_2NC(N)$), 33.40 (1C, $Ph\underline{C}H_2CH_2$), 32.46 (1C, $CH_2CH_2CH_2$), 25.58 (1C, $CH_2\underline{C}H_2CH_2$), 25.35 (1C, $CH_2\underline{C}H_2CH_2$), 22.05 (1C, $CH_2\underline{C}H_2CH_2$). Anal. Calcd: C 53.42 %; H 7.11 %; N 10.74 %. Found: C 53.35 %; H 7.15 %; N 10.73 %.

Preparation of 1-(benzyl)-1-{4-{4-{3-[3-(4-chlorophenyl)propoxy]prop-1-yl}piperazin-1-yl}but-1-yl}guanidine trihydrochloride (**ADS10278**)

4M solution HCl-dioxan (1.56 mL; $6.24 \cdot 10^{-3}$ mol) was added dropwise to a solution of the 1-(benzyl)-1-{4-{4-{3-[3-(4-chlorophenyl)propoxy]prop-1-yl}piperazin-1-yl}but-1-yl}-2,3-di(*tert*-butoxycarbonyl)guanidine (**16c**) (0.219 g; $3.13 \cdot 10^{-4}$ mol) in 20 mL chloroform. The reaction was stirred overnight at room temperature, then the solvent was removed under vacuum. The crude product was evaporated twice from chloroform and twice from EtOAc. The residue was stirred twice with EtOAc at 70 °C, then the supernatant was carefully decanted from the precipitate. The precipitate was evaporated and dried to yield the pure product.

1-(benzyl)-1-{4-{4-{3-[3-(4-chlorophenyl)propoxy]prop-1-yl}piperazin-1-yl}but-1-yl}guanidine trihydrochloride (**ADS10278**): $C_{28}H_{42}ClN_5O \cdot 3HCl$. M=652.06. White solid. 53.54 %. mp: 189.7-192.0 °C. ^1H NMR (600 MHz, *CD₃OD*) δ ppm 7.45-7.42 (m, $2H^{arom.}$), 7.37-7.35 (m, $1H^{arom.}$, $C(CHCH)_2C\underline{H}$), 7.31-7.28 (m, 4H: $2H^{arom.}$; $2H^{phenyl.}$, $C(CHC\underline{H})_2CCl$), 7.22-7.21 (m, $2H^{phenyl.}$, $C(C\underline{H}CH)_2CCl$), 4.71 (br, 2H, $NC\underline{H}_2Ph$), 3.92-3.64 (br, 8H, $8H^{piperazine}$), 3.58 (t, 2H, $OC\underline{H}_2$, *J* = 5.68Hz), 3.48 (t, 2H, $OC\underline{H}_2$, *J* = 6.33Hz), 3.45 (t, 2H, $CH_2C\underline{H}_2NC(N)$, *J* = 7.80 Hz), 3.41 (br, 2H, $\underline{C}H_2N^{piperazine}$), 3.31 (br, 2H, $\underline{C}H_2N^{piperazine}$), 2.71-2.69 (m, 2H, $ClPhC\underline{H}_2$), 2.12 (qt, 2H, $PhCH_2C\underline{H}_2$), 1.90 (qt, 2H, $OCH_2C\underline{H}_2CH_2N^{piperazine}$), 1.85 (qt, 2H, $CH_2C\underline{H}_2CH_2$), 1.78 (m, 2H, $CH_2C\underline{H}_2CH_2$). ^{13}C NMR (150.95 MHz, *CD₃OD*) δ ppm 158.37 ($1C^{quat.}$, $\underline{C}$=N), 142.07 ($1C^{quat./phenyl}$, $\underline{C}CH_2$), 136.32 ($1C^{quat./arom.}$), 132.58 ($1C^{quat./phenyl.}$, $\underline{C}Cl$), 131.14 ($2C^{phenyl.}$, $C(\underline{C}HCH)_2CCl$), 130.13 ($2C^{arom.}$, $C(CH\underline{C}H)_2CH$), 129.44 ($2C^{phenyl.}$, $C(CH\underline{C}H)_2CCl$), 129.18 ($2C^{arom.}$, $C(\underline{C}HCH)_2CH$), 128.03 ($1C^{arom.}$, $C(CHCH)_2\underline{C}H$), 71.22 (1C, $O\underline{C}H_2$), 68.38 (1C, $OC\underline{H}_2$), 57.51 (1C, $\underline{C}H_2N^{piperazine}$), 56.32 (1C, $\underline{C}H_2N^{piperazine}$), 52.76 (1C, $N\underline{C}H_2Ph$), 49.94 ($4C^{piperazine}$), 49.62 (1C, $\underline{C}H_2C(N)N$), 32.66 (1C, $ClPh\underline{C}H_2$), 32.28 (1C, $PhCH_2\underline{C}H_2$), 25.53 (1C, $OCH_2\underline{C}H_2CH_2N^{piperazine}$), 25.26 (1C, $CH_2\underline{C}H_2CH_2$), 22.03 (1C, $CH_2\underline{C}H_2CH_2$). Anal. Calcd: C 55.18 %; H 7.44 %; N 11.49 %. Found: C 55.18 %; H 7.54 %; N 11.48 %.

Preparation of 1-{4-{4-{3-[3-(4-chlorophenyl)propoxy]propyl}piperazin-1-yl}but-1-yl}-1-(4-(trifluoromethyl)benzyl)guanidine trihydrochloride (**ADS10279**)

4M solution HCl-dioxan (1.58 mL; $6.32 \cdot 10^{-3}$ mol) was added dropwise to a solution of the 1-{4-{4-{3-[3-(4-chlorophenyl)propoxy]propyl}piperazin-1-yl}but-1-yl}-2,3-di(*tert*-butoxycarbonyl)-1-(4-(trifluoromethyl)benzyl)guanidine (**16d**) (0.243 g; $3.04 \cdot 10^{-4}$ mol) in 20 mL chloroform. The reaction was stirred overnight at room temperature, then the solvent was removed under vacuum. The crude product was evaporated twice from chloroform and twice from EtOAc. The residue was stirred twice with EtOAc at 70 °C, then the supernatant was carefully decanted from the precipitate. The precipitate was evaporated and dried to yield the pure product.

1-{4-{4-{3-[3-(4-chlorophenyl)propoxy]propyl}piperazin-1-yl}but-1-yl}-1-(4-(trifluoromethyl)benzyl)guanidine trihydrochloride (**ADS10279**): $C_{29}H_{41}ClF_3N_5O \cdot 3HCl \cdot H_2O$. M=695.52. White solid. 43.66 %. mp: 210.4-212.4 °C. ^1H NMR (600 MHz, *CD_3OD*) δ ppm 7.76-7.73 (m, $2H^{arom.}$), 7.52-7.50 (m, $2H^{arom.}$), 7.30-7.28 (m, $2H^{phenyl.}$, $C(CHC\underline{H})_2CCl$), 7.23-7.21 (m, $2H^{phenyl.}$, $C(C\underline{H}CH)_2CCl$), 4.79 (br, 2H, $NC\underline{H}_2Ph$), 3.94 (br, $4H^{piperazine}$), 3.67 (br, $4H^{piperazine}$), 3.58 (t, 2H, $OC\underline{H}_2$, J = 5.68Hz), 3.51-3.48 (m, 4H, $OC\underline{H}_2$, $CH_2C\underline{H}_2NC(N)$), 3.42 (br, 2H, $\underline{C}H_2N^{piperazine}$), 3.35 (br, 2H, $\underline{C}H_2N^{piperazine}$), 2.71-2.69 (t, 2H, $ClPhC\underline{H}_2$, J = 7.80Hz), 2.15-2.10 (m, 2H, $CH_2C\underline{H}_2CH_2$), 1.93-1.82 (m, 6H, $CH_2C\underline{H}_2CH_2$). ^{13}C NMR (150.95 MHz, *CD_3OD*) δ ppm 158.46 ($1C^{quat.}$, $\underline{C}$=N), 142.07 ($1C^{quat./arom.}$, $\underline{C}(CHCH)_2CH$), 141.08 ($1C^{quat./phenyl}$, $\underline{C}CH_2$), 132.58 ($1C^{quat./phenyl.}$, $\underline{C}Cl$), 131.51, 131.29, 131.08, 130.86 ($1C^{quat./arom.}$, $\underline{C}CF_3$), 131.14 ($2C^{phenyl.}$, $C(\underline{C}HCH)_2CCl$), 129.42 ($2C^{phenyl.}$, $C(CH\underline{C}H)_2CCl$), 128.45 ($2C^{arom.}$, $C(\underline{C}HCH)_2CH$), 128.28, 126.48, 124.68, 122.89 (1C, $\underline{C}F_3$), 126.95, 126.92 ($2C^{arom.}$, $C(CH\underline{C}H)_2CCF_3$), 71.21 (1C, $O\underline{C}H_2$), 68.37 (1C, $O\underline{C}H_2$), 57.50 (1C, $CH_2N^{piperazine}$), 56.56 (1C, $\underline{C}H_2N^{piperazine}$), 52.37 (1C, $N\underline{C}H_2Ph$), 49.98 ($4C^{piperazine}$), 49.69 (1C, $\underline{C}H_2C(N)N$), 32.60 (1C, $ClPh\underline{C}H_2$), 32.27 (1C, $PhCH_2\underline{C}H_2$), 25.52 (1C, $OCH_2\underline{C}H_2CH_2N^{piperazine}$), 25.30 (1C, $CH_2\underline{C}H_2CH_2$), 21.98 (1C, $CH_2\underline{C}H_2CH_2$). Anal. Calcd: C 50.08 %; H 6.67 %; N 10.07 %. Found: C 50.04 %; H 6.72 %; N 9.81 %.

Preparation of 3-(piperidin-1-yl)propanenitrile (**17a**)

Acrylonitrile (1.37 g; $2.58 \cdot 10^{-2}$ mol) was added dropwise to a solution of piperidine (2.00 g; $2.35 \cdot 10^{-2}$ mol) in 70 mL methanol. The reaction was stirred overnight at room temperature, then the solvent was removed under vacuum to yield the pure product.

3-(piperidin-1-yl)propanenitrile (**17a**): $C_8H_{14}N_2$. M=138.21. Yellowish liquid. 99.80 % yield. R_f=0.52 (EtOAc). ^1H NMR (600 MHz, *$CDCl_3$*) δ ppm 2.67 (t, 2H, $C\underline{H}_2N^{piperidine}$; J=7.20Hz), 2.50 (t, 2H, $C\underline{H}_2CN$; J=7.22Hz), 2.44-2.42 (m, $4H^{piperidine}$, $N(C\underline{H}_2CH_2)_2CH_2$), 1.61-1.57 (m, $4H^{Piperidine}$, $N(CH_2C\underline{H}_2)_2CH_2$), 1.45-1.42 (m, $2H^{piperidine}$, $N(CH_2CH_2)_2C\underline{H}_2$). ^{13}C NMR (150.95 MHz, *$CDCl_3$*) δ ppm 118.96 ($1C^{quat.}$, $\underline{C}$≡N), 54.09 (1C, $\underline{C}H_2N^{piperidine}$), 53.92 ($2C^{piperidine}$, $N(\underline{C}H_2CH_2)_2CH_2$), 25.77 ($2C^{piperidine}$, $N(CH_2\underline{C}H_2)_2CH_2$), 24.02 ($1C^{piperidine}$, $N(CH_2CH_2)_2\underline{C}H_2$), 15.16 (1C, $\underline{C}H_2CN$).

Preparation of 4-(piperidin-1-yl)butanenitrile (**17b**)

4-bromobutyronitrile (1.738 g; $1.17 \cdot 10^{-2}$ mol) in 20 mL acetonitrile was added dropwise to a solution of piperidine (1.00 g; $1.17 \cdot 10^{-2}$ mol) and potassium carbonate (8.10 g; $5.87 \cdot 10^{-2}$ mol) in 30 mL acetonitrile. The reaction was stirred for 72 hours at room temperature. The precipitate was discarded. The solvent was removed under vacuum and the crude product was purified by column chromatography (EtOAc/ Triethylamine 90:1) to yield the pure product.

4-(piperidin-1-yl)butanenitrile (**17b**): $C_9H_{16}N_2$. M=152.24. Colourless liquid. 96.14 % yield. R_f=0.41 (EtOAc/Triethylamine 90:1). ^{1}H NMR (600 MHz, *CDCl$_3$*) δ ppm 2.41 (t, 2H, $C\underline{H}_2N^{piperidine}$; J=7.18Hz), 2.39 (t, 2H, $C\underline{H}_2CN$; J=6.92Hz), 2.35 (br, $4H^{piperidine}$, $N(C\underline{H}_2CH_2)_2CH_2$), 1.82 (qt, 2H, $CH_2C\underline{H}_2CH_2$), 1.58-1.54 (m, $4H^{piperidine}$, $N(CH_2C\underline{H}_2)_2CH_2$), 1.44-1.42 (m, $2H^{piperidine}$, $N(CH_2CH_2)_2C\underline{H}_2$). ^{13}C NMR (150.95 MHz, *CDCl$_3$*) δ ppm 119.84 ($1C^{quat.}$, $\underline{C}{\equiv}N$), 57.09 (1C, $\underline{C}H_2N^{piperidine}$), 54.48 ($2C^{piperidine}$, $N(\underline{C}H_2CH_2)_2CH_2$), 25.94 ($2C^{piperidine}$, $N(CH_2\underline{C}H_2)_2CH_2$), 24.36 ($1C^{piperidine}$, $N(CH_2CH_2)_2\underline{C}H_2$), 22.90 (1C, $CH_2\underline{C}H_2CH_2$), 14.93 (1C, $\underline{C}H_2CN$).

Preparation of 3-(piperidin-1-yl)propan-1-amine (**18a**)

$LiAlH_4$ (3.57 g; $9.41 \cdot 10^{-2}$ mol) was added to a solution of 3-(piperidin-1-yl)propanenitrile (**17a**) (3.25 g; $2.35 \cdot 10^{-2}$ mol) in 120 mL anhydrous diethyl ether. The reaction was stirred overnight at room temperature, then the mixture was quenched by dropwise addition of water (16 equiv.) and 10 % NaOH solution (16 equiv.) stirred for two hours, then filtered. The precipitate was discarded. The organic layer was dried over Na_2SO_4, then the solvent was removed under vacuum and the crude product was purified by column chromatography (DCM/MeOH/25% NH_3aq. 49:10:1) to yield the pure product.

3-(piperidin-1-yl)propan-1-amine (**18a**): $C_8H_{18}N_2$. M=142.24. Yellowish sticky oil. 80.24 % yield. R_f=0.22 (DCM/MeOH/25% NH_3aq. 49:10:1). ^{1}H NMR (600 MHz, *CDCl$_3$*) δ ppm 2.76-2.66 (m, 2H, $C\underline{H}_2N^{piperidine}$), 2.37-2.34 (m, 6H: $4H^{piperidine}$, $N(C\underline{H}_2CH_2)_2CH_2$; $C\underline{H}_2NH_2$), 1.66 (qt, 2H, $CH_2C\underline{H}_2CH_2$), 1.60-1.56 (m, $4H^{piperidine}$, $N(CH_2C\underline{H}_2)_2CH_2$), 1.43 (br, 4H: $2H^{piperidine}$, $N(CH_2CH_2)_2C\underline{H}_2$, $N\underline{H}_2$). ^{13}C NMR (150.95 MHz, *CDCl$_3$*) δ ppm 57.18, (1C, $\underline{C}H_2N^{piperidine}$), 54.57 ($2C^{piperidine}$, $N(\underline{C}H_2CH_2)_2CH_2$), 40.75 (1C, $\underline{C}H_2NH_2$), 29.88 (1C, $CH_2\underline{C}H_2CH_2$), 25.86 ($2C^{piperidine}$, $N(CH_2\underline{C}H_2)_2CH_2$), 24.36 ($1C^{piperidine}$, $N(CH_2CH_2)_2\underline{C}H_2$).

Preparation of 4-(piperidin-1-yl)butan-1-amine (**18b**)

$LiAlH_4$ (1.44 g; $3.79 \cdot 10^{-2}$ mol) was added to a solution of 4-(piperidin-1-yl)butanenitrile (**17b**) (1.446 g; $9.49 \cdot 10^{-3}$ mol) in 50 mL anhydrous diethyl ether. The reaction was stirred overnight at room temperature, then the mixture was quenched by dropwise addition of water (16 equiv.) and 10 % NaOH solution (16 equiv.) stirred for two hours, then filtered. The precipitate was discarded. The organic layer was dried over Na_2SO_4, then the solvent was removed under vacuum and the crude product was purified by column chromatography (DCM/MeOH/25% NH_3aq. 39:10:1) to yield the pure product.

4-(piperidin-1-yl)butan-1-amine (**18b**): $C_9H_{20}N_2$. M=156.27. Yellowish liquid. 53.77 % yield. R_f=0.28 (DCM/MeOH/25% NH_3aq. 39:10:1). ^{1}H NMR (600 MHz, *$CDCl_3$)* δ ppm 2.71 (t, 2H, $C\underline{H}_2NH_2$; *J*=6.79Hz), 2.38 (br, $4H^{piperidine}$, $N(C\underline{H}_2CH_2)_2CH_2$), 2.30 (t, 2H, $C\underline{H}_2N^{piperidine}$; *J*=7.50Hz), 2.22 (br, 2H, $N\underline{H}_2$, *), 1.60-1.56 (m, $4H^{piperidine}$, $N(CH_2C\underline{H}_2)_2CH_2$), 1.55-1.51 (m, 2H, $CH_2C\underline{H}_2CH_2$), 1.49-1.43 (m, 4H: $2H^{piperidine}$, $N(CH_2CH_2)_2C\underline{H}_2$; $CH_2C\underline{H}_2CH_2$). ^{13}C NMR (150.95 MHz, *$CDCl_3$)* δ ppm 59.18, (1C, $\underline{C}H_2N^{piperidine}$), 54.50 ($2C^{piperidine}$, $N(\underline{C}H_2CH_2)_2CH_2$), 41.89 (1C, $\underline{C}H_2NH_2$), 31.59 (1C, $CH_2\underline{C}H_2CH_2$), 25.86 ($2C^{piperidine}$, $N(CH_2\underline{C}H_2)_2CH_2$), 24.38 (1C, $CH_2\underline{C}H_2CH_2$), 24.32 ($1C^{piperidine}$, $N(CH_2CH_2)_2\underline{C}H_2$).

Preparation of 1-(3-bromopropyl)-4-chlorobenzene (**19**)

3-(4-Chlorophenyl)-1-propanol (1.00 g; $5.86 \cdot 10^{-3}$ mol) in 6 mL toluene was slowly added to an ice-cooled mixture of phosphorus tribromide (2.22 g; $8.20 \cdot 10^{-3}$ mol) in 5 mL toluene. The ice bath was removed and the reaction was stirred overnight at room temperature. The 30 g of ice-cubes were added and the mixture was extracted 3x15 mL with DCM. The combined organic phases were dried over anhydrous Na_2SO_4. The solvent was removed under vacuum and the crude product was purified by column chromatography (Hexane/DCM 10:1) to yield the pure product.

1-(3-bromopropyl)-4-chlorobenzene (**19**): $C_9H_{10}BrCl$. M=233.53. Colourless liquid. 53.36 % yield. R_f=0.46 (Hexane/DCM 10:1). ^{1}H NMR (600 MHz, *$CDCl_3$)* δ ppm 7.27-7.25 (m, $2H^{arom.}$, $C(CHC\underline{H})_2CCl$), 7.13-7.12 (m, $2H^{arom.}$, $C(C\underline{H}CH)_2CCl$), 3.37 (t, 2H, $C\underline{H}_2Br$; *J*=6.51Hz), 2.75 (t, 2H, $PhC\underline{H}_2$, *J*=7.38Hz), 2.15 (qt, 2H, $CH_2C\underline{H}_2CH_2$). ^{13}C NMR (150.95 MHz, *$CDCl_3$)* δ ppm 138.94 ($1C^{quat./arom.}$, $\underline{C}CH_2$), 131.94 ($1C^{quat./arom.}$, $\underline{C}Cl$), 129.89 ($2C^{arom.}$, $C(\underline{C}HCH)_2CCl$), 128.60 ($2C^{arom.}$, $C(CH\underline{C}H)_2CCl$), 33.95 (1C, $\underline{C}H_2$), 33.27 (1C, $\underline{C}H_2$), 32.74 (1C, $\underline{C}H_2$).

Preparation of 4-(4-chlorophenyl)butan-1-ol (**20**)

Borane-*tert*-butylamine-complex (4.88 g; $5.61 \cdot 10^{-2}$ mol) was added to an ice-cooled mixture of aluminum chloride (3.74 g; $2.80 \cdot 10^{-2}$ mol) in 40 mL DCM. After 10 minutes 3-(4-chlorobenzoyl)propionic acid (2.00 g; $9.41 \cdot 10^{-3}$ mol) was slowly added to the reaction mixture. The reaction was stirred was refluxed for 9 days at 40 °C under an argon atmosphere. After cooling the mixture was poured under vigorous stirring into 140 g of ice-cubes with 50 mL of 0.2 M HCl. After two hours of stirring the organic phase was separated and washed 3 times with 50 mL of water, saturated aqueous $NaHCO_3$. The organic phase was dried over anhydrous Na_2SO_4. The solvent was removed under vacuum and the crude product was purified by column chromatography (DCM) to yield the pure product.

4-(4-chlorophenyl)butan-1-ol (**20**): $C_{10}H_{13}ClO$. M=184.66. Colourless liquid. 70.35 % yield. R_f=0.35 (DCM). ^{1}H NMR (600 MHz, *$CDCl_3$)* δ ppm 7.24-7.23 (m, $2H^{arom.}$, $C(CHC\underline{H})_2CCl$), 7.11-7.09 (m, $2H^{arom.}$, $C(C\underline{H}CH)_2CCl$), 3.65 (t, 2H, $C\underline{H}_2OH$; *J*=6.20Hz), 2.62 (t, 2H, $PhC\underline{H}_2$, *J*=7.60Hz), 1.68 (qt, 2H, $HOCH_2C\underline{H}_2$), 1.59 (qt, 2H, $PhCH_2C\underline{H}_2$), 1.36 (br, 1H, $O\underline{H}$). ^{13}C NMR (150.95 MHz, *$CDCl_3$)* δ ppm 140.71 ($1C^{quat./arom.}$, $\underline{C}CH_2$), 131.41

($1C^{quat./arom.}$, $\underline{C}Cl$), 129.70 ($2C^{arom.}$, $C(\underline{C}HCH)_2CCl$), 128.34 ($2C^{arom.}$, $C(CH\underline{C}H)_2CCl$), 62.61 (1C, $\underline{C}H_2OH$), 34.94 (1C, $PhC\underline{H}_2$), 32,13 (1C, $PhCH_2\underline{C}H_2$), 27.43 (1C, $HOCH_2\underline{C}H_2$).

Preparation of 1-(4-bromobutyl)-4-chlorobenzene (**21**)

4-(4-chlorophenyl)butan-1-ol (**20**) (0.387 g; $2.09 \cdot 10^{-3}$ mol) in 5 mL toluene was slowly added to an ice-cooled mixture of phosphorus tribromide (0.79 g; $2.92 \cdot 10^{-3}$ mol) in 5 mL toluene. The ice bath was removed and the reaction was stirred overnight at room temperature. The 30 g of ice-cubes were added and the mixture was extracted 3x15 mL with DCM. The combined organic phases were dried over anhydrous Na_2SO_4. The solvent was removed under vacuum and the crude product was purified by column chromatography (Hexane/DCM 10:1) to yield the pure product.

1-(4-bromobutyl)-4-chlorobenzene (**21**): $C_{10}H_{12}BrCl$. M=247.56. Colourless liquid. 78.84 % yield. R_f=0.63 (Hexane/DCM 10:1). ^1H NMR (600 MHz, *CDCl₃*) δ ppm 7.25-7.23 (m, $2H^{arom.}$, $C(CHC\underline{H})_2CCl$), 7.10-7.09 (m, $2H^{arom.}$, $C(C\underline{H}CH)_2CCl$), 3.41 (t, 2H, $C\underline{H}_2Br$; *J*=6.69Hz), 2.61 (t, 2H, $PhC\underline{H}_2$, *J*=7.62Hz), 1.87 (qt, 2H, $PhCH_2CH_2C\underline{H}_2CH_2Br$), 1.75 (qt, 2H, $PhCH_2C\underline{H}_2CH_2CH_2Br$). ^{13}C NMR (150.95 MHz, *CDCl₃*) δ ppm 140.18 ($1C^{quat./arom.}$, $\underline{C}CH_2$), 131.63 ($1C^{quat./arom.}$, $\underline{C}Cl$), 129.69 ($2C^{arom.}$, $C(\underline{C}HCH)_2CCl$), 128.47 ($2C^{arom.}$, $C(CH\underline{C}H)_2CCl$), 34.30 (1C, $\underline{C}H_2C^{arom.}$), 33.43 (1C, $\underline{C}H_2Br$), 32.08 (1C, $PhCH_2CH_2\underline{C}H_2CH_2Br$), 29.69 (1C, $PhCH_2\underline{C}H_2CH_2CH_2Br$).

Preparation of 3-(4-chlorophenyl)-*N*-[3-(piperidin-1-yl)propyl]propan-1-amine (**22a**)

1-(3-bromopropyl)-4-chlorobenzene (**19**) (0.194 g; $8.29 \cdot 10^{-4}$ mol) in 10 mL acetonitrile was added dropwise to a solution of 3-(piperidin-1-yl)propan-1-amine (**18a**) (0.118 g; $8.29 \cdot 10^{-4}$ mol) and potassium carbonate (0.57 g; $4.12 \cdot 10^{-3}$ mol) in 20 mL acetonitrile heated to 60 °C. The reaction was stirred overnight at 60 °C. The precipitate was discarded. The solvent was removed under vacuum and the crude product was purified by column chromatography (DCM/MeOH/25% NH_3aq. 89:10:1) to yield the pure product.

3-(4-chlorophenyl)-*N*-[3-(piperidin-1-yl)propyl]propan-1-amine (**22a**): $C_{17}H_{27}ClN_2$. M=294.86. Yellowish waxy solid. 42.68 % yield. R_f=0.33 (DCM/MeOH/25% NH_3aq. 89:10:1). mp: 114.0-116.0 °C. ^1H NMR (600 MHz, *CDCl₃*) δ ppm 7.26-7.24 (m, $2H^{arom.}$, $C(CHC\underline{H})_2CCl$), 7.12-7.11 (m, $2H^{arom.}$, $C(C\underline{H}CH)_2CCl$), 4.09 (br, 1H, $N\underline{H}$, *), 2.81 (t, 2H, $NHC\underline{H}_2CH_2CH_2N^{piperidine}$, *J*=6.38Hz), 2.71 (t, 2H, $NHC\underline{H}_2CH_2CH_2Ph$, *J*=7.50Hz), 2.66 (t, 2H, $NHCH_2CH_2C\underline{H}_2Ph$, *J*=7.68Hz), 2.47-2.38 (m, 6H: $4H^{piperidine}$, $N(C\underline{H}_2CH_2)_2CH_2$; $NHCH_2CH_2C\underline{H}_2N^{piperidine}$), 1.91 (qt, 2H, $NHCH_2C\underline{H}_2CH_2Ph$), 1.80 (qt, 2H, $NHCH_2C\underline{H}_2CH_2N^{piperidine}$), 1.60-1.57 (m, $4H^{piperidine}$, $N(CH_2C\underline{H}_2)_2CH_2$), 1.45 (m, $2H^{piperidine}$, $N(CH_2CH_2)_2C\underline{H}_2$). ^{13}C NMR (150.95 MHz, *CDCl₃*) δ ppm 140.12 ($1C^{quat./arom.}$, $\underline{C}CH_2$), 131.56

($1C^{quat./arom.}$, $\underline{C}Cl$), 129.65 ($2C^{arom.}$, $C(\underline{C}HCH)_2CCl$), 128.43 ($2C^{arom.}$, $C(CH\underline{C}H)_2CCl$), 57.99, (1C, $\underline{C}H_2N^{piperidine}$), 54.59 ($2C^{piperidine}$, $N(\underline{C}H_2CH_2)_2CH_2$), 49.00 (1C, $\underline{C}H_2NH$), 48.82 (1C, $\underline{C}H_2NH$), 32.82 (1C, $\underline{C}H_2C^{arom.}$), 30.91 (1C, $CH_2\underline{C}H_2CH_2C^{arom.}$), 25.95 ($2C^{piperidine}$, $N(CH_2\underline{C}H_2)_2CH_2$), 25.86 ($1C^{piperidine}$, $N(CH_2CH_2)_2\underline{C}H_2$), 24.38 (1C, $CH_2\underline{C}H_2CH_2N^{piperidine}$).

Preparation of *N*-(3-(4-chlorophenyl)propyl)-4-(piperidin-1-yl)butan-1-amine (**22b**)

1-(3-bromopropyl)-4-chlorobenzene (**19**) (0.298 g; $1.28 \cdot 10^{-3}$ mol) in 10 mL acetonitrile was added dropwise to a solution of 4-(piperidin-1-yl)butan-1-amine (**18b**) (0.200 g; $1.28 \cdot 10^{-3}$ mol) and potassium carbonate (0.884 g; $6.39 \cdot 10^{-3}$ mol) in 20 mL acetonitrile heated to 60 °C. The reaction was stirred overnight at 60 °C. The precipitate was discarded. The solvent was removed under vacuum and the crude product was purified by column chromatography (DCM/MeOH/25% NH_3aq. 139:10:1) to yield the pure product.

N-(3-(4-chlorophenyl)propyl)-4-(piperidin-1-yl)butan-1-amine (**22b**): $C_{18}H_{29}ClN_2$. M=308.89. Yellowish sticky oil. 43.04 % yield. R_f=0.34 (DCM/MeOH/25% NH_3aq. 139:10:1). ^1H NMR (600 MHz, *CDCl₃*) δ ppm 7.24-7.23 (m, $2H^{arom.}$, $C(CHC\underline{H})_2CCl$), 7.12-7.10 (m, $2H^{arom.}$, $C(C\underline{H}CH)_2CCl$), 2.64-2.60 (m, 6H: $C\underline{H}_2C^{arom.}$, $C\underline{H}_2NH$), 2.37 (br, $4H^{piperidine}$, $N(C\underline{H}_2CH_2)_2CH_2$), 2.30 (t, 2H, $C\underline{H}_2N^{piperidine}$, *J*=7.20Hz), 1.81 (qt, 2H, $PhCH_2C\underline{H}_2CH_2$), 1.59-1.56 (m, 5H: (m, $4H^{piperidine}$, $N(CH_2C\underline{H}_2)_2CH_2$, $N\underline{H}$*), 1.43 (br, $2H^{piperidine}$, $N(CH_2CH_2)_2C\underline{H}_2$), 1.53-1.52 (m, 4H, $CH_2CH_2C\underline{H}_2CH_2$). ^{13}C NMR (150.95 MHz, *CDCl₃*) δ ppm 140.41 ($1C^{quat./arom.}$, $\underline{C}CH_2$), 131.44 ($1C^{quat./arom.}$, $\underline{C}Cl$), 129.67 ($2C^{arom.}$, $C(\underline{C}HCH)_2CCl$), 128.37 ($2C^{arom.}$, $C(CH\underline{C}H)_2CCl$), 59.26, (1C, $\underline{C}H_2N^{piperidine}$), 54.53 ($2C^{piperidine}$, $N(\underline{C}H_2CH_2)_2CH_2$), 49.75 (1C, $\underline{C}H_2NH$), 49.09 (1C, $\underline{C}H_2NH$), 32.95 (1C, $\underline{C}H_2C^{arom.}$), 31.37 (1C, $PhCH_2\underline{C}H_2CH_2$), 28.08 (1C, $CH_2CH_2\underline{C}H_2CH_2$), 25.91 ($2C^{piperidine}$, $N(CH_2\underline{C}H_2)_2CH_2$), 24.78 ($1C^{piperidine}$, $N(CH_2CH_2)_2\underline{C}H_2$), 24.38 (1C, $CH_2CH_2\underline{C}H_2CH_2$).

Preparation of 4-(4-chlorophenyl)-*N*-[3-(piperidin-1-yl)propyl]butan-1-amine (**22c**)

1-(4-bromobutyl)-4-chlorobenzene (**21**) (0.409 g; $1.65 \cdot 10^{-3}$ mol) in 10 mL acetonitrile was added dropwise to a solution of 3-(piperidin-1-yl)propan-1-amine (**18a**) (0.258 g; $1.81 \cdot 10^{-3}$ mol) and potassium carbonate (1.14 g; $8.25 \cdot 10^{-3}$ mol) in 20 mL acetonitrile heated to 60 °C. The reaction was stirred overnight at 60 °C. The precipitate was discarded. The solvent was removed under vacuum and the crude product was purified by column chromatography (DCM/MeOH/25% NH_3aq. 49:10:1) to yield the pure product.

4-(4-chlorophenyl)-*N*-[3-(piperidin-1-yl)propyl]butan-1-amine (**22c**): $C_{18}H_{29}ClN_2$. M=308.89. Yellowish sticky oil. 43.33 % yield. R_f=0.69 (DCM/MeOH/25% NH_3aq. 49:10:1). ^1H NMR (600 MHz, *CDCl₃*) δ ppm 7.24-7.23 (m, $2H^{arom.}$, $C(CHC\underline{H})_2CCl$), 7.10-7.09 (m, $2H^{arom.}$, $C(C\underline{H}CH)_2CCl$), 2.72 (t, 2H, $N^{piperidine}CH_2CH_2C\underline{H}_2NH$, *J*=6.61Hz), 2.66 (t, 2H, $PhCH_2CH_2CH_2C\underline{H}_2NH$, *J*=7.09Hz), 2.60 (t, 2H, $C\underline{H}_2Ph$, *J*=7.49Hz), 2.39-2.33 (m, 6H: $4H^{piperidine}$, $N(C\underline{H}_2CH_2)_2CH_2$; $C\underline{H}_2N^{piperidine}$), 1.74 (qt, 2H, $C\underline{H}_2CH_2N^{piperidine}$), 1.64 (qt, 2H, $PhCH_2CH_2C\underline{H}_2CH_2NH$), 1.59-1.52 (m, 6H: $4H^{piperidine}$, $N(CH_2C\underline{H}_2)_2CH_2$; $PhCH_2C\underline{H}_2CH_2CH_2NH$), 1.43-1.42 (m, 3H:, $2H^{piperidine}$,

$N(CH_2CH_2)_2C\underline{H}_2$; $N\underline{H}$*).^{13}C NMR (150.95 MHz, *CDCl₃*) δ ppm 140.52 (1C^quat./arom.^, $\underline{C}CH_2$), 131.32 (1C^quat./arom.^, $\underline{C}Cl$), 129.64 (2C^arom.^, $C(\underline{C}HCH)_2CCl$), 128.26 (2C^arom.^, $C(CH\underline{C}H)_2CCl$), 57.96 (1C, $\underline{C}H_2N^{piperidine}$), 54.51 ($2C^{piperidine}$, $N(\underline{C}H_2CH_2)_2CH_2$), 49.23 (1C, $PhCH_2CH_2CH_2\underline{C}H_2NH$), 49.00 (1C, $N^{piperidine}CH_2CH_2\underline{C}H_2NH$), 34.94 (1C, $\underline{C}H_2C^{arom.}$), 28.29, 28.85 (2C, $PhCH_2\underline{C}H_2\underline{C}H_2CH_2NH$), 25.86 ($2C^{piperidine}$, $N(CH_2\underline{C}H_2)_2CH_2$), 25.76 (1C, $\underline{C}H_2CH_2N^{piperidine}$), 24.21 ($1C^{piperidine}$, $N(CH_2CH_2)_2\underline{C}H_2$).

Preparation of 1-(3-(4-chlorophenyl)propyl)-2,3-di(*tert*-butoxycarbonyl)-1-(3-(piperidin-1-yl)propyl)guanidine (**23a**)

1,3-bis(*tert*-butoxycarbonyl)-2-methylisothiourea (0.317 g; $1.09\cdot10^{-3}$ mol) and mercury II chloride (0.297 g; $1.09\cdot10^{-3}$ mol) were sequentially added to an ice-cooled mixture of 3-(4-chlorophenyl)-*N*-[3-(piperidin-1-yl)propyl]propan-1-amine (**22a**) (0.293 g; $9.94\cdot10^{-4}$ mol) and triethylamine (0.503 g; $4.97\cdot10^{-3}$ mol) in 30 mL DCM. The ice bath was removed and the reaction was stirred for eighteen hours at room temperature, then filtered. The precipitate was discarded. The filtrate was washed sequentially twice with 15 mL H_2O and twice with 15 mL brine. The combined organic phases were dried over Na_2SO_4, then the solvent was removed under vacuum and the crude product was purified by column chromatography (EtOAc/MeOH/Triethylamine 139:10:1) to yield the pure product.

1-(3-(4-chlorophenyl)propyl)-2,3-di(*tert*-butoxycarbonyl)-1-(3-(piperidin-1-yl)propyl)guanidine (**23a**): $C_{28}H_{45}ClN_4O_4$. M=537.13. Yellowish sticky oil. 55.27 % yield. R_f=0.36 (EtOAc/MeOH/Triethylamine 139:10:1). ^{1}H NMR (600 MHz, *CDCl₃*) δ ppm 10.75 (br, 1H, $N\underline{H}$), 7.23-7.22 (m, $2H^{arom.}$, $C(CHC\underline{H})_2CCl$), 7.16-7.15 (m, $2H^{arom.}$, $C(C\underline{H}CH)_2CCl$), 3.32-3.29 (m, 4H, $C\underline{H}_2NC\underline{H}_2$), 2.61 (t, 2H, $ClPhC\underline{H}_2$, J=7.61Hz), 2.37 (br, $4H^{Piperidine}$, $N(\underline{C}H_2CH_2)_2CH_2$), 2.26 (t, 2H, $C\underline{H}_2N^{piperidine}$, J=5.93Hz), 1.97 (qt, 2H, $ClPhCH_2C\underline{H}_2$), 1.72-1.68 (m, 6H: $4H^{Piperidine}$, $N(CH_2C\underline{H}_2)_2CH_2$; $CH_2C\underline{H}_2CH_2N^{Piperidine}$), 1.58-1.47 (m, 20H: $2H^{Piperidine}$, $N(CH_2CH_2)_2C\underline{H}_2$; $C\underline{H}_3$). ^{13}C NMR (150.95 MHz, *CDCl₃*) δ ppm 160.76 (1C, $\underline{C}$=O), 151.89 (1C, $\underline{C}$=O), 151.56 ($1C^{quat.}$, $\underline{C}$=N), 140.01($1C^{quat./arom.}$, $\underline{C}CH_2$), 131.46 ($1C^{quat./arom.}$, $\underline{C}Cl$), 129.75 ($2C^{arom.}$, $C(\underline{C}HCH)_2CCl$), 128.38 ($2C^{arom.}$, $C(CH\underline{C}H)_2CCl$), 80.83 (1Cq Boc), 78.00 (1Cq Boc), 53.97 ($2C^{Piperidine}$, $N(\underline{C}H_2CH_2)_2CH_2$), 53.37 (1C, $\underline{C}H_2N^{piperidine}$), 45.96 (1C, $\underline{C}H_2N$), 44.26 (1C, $\underline{C}H_2N$), 32.43 (1C, $ClPhC\underline{H}_2$), 28.37(3C, $\underline{C}H_3$), 28.31 (3C, $\underline{C}H_3$), 27.46 (1C, $CH_2\underline{C}H_2CH_2$), 24.40 ($2C^{Piperidine}$, $N(CH_2\underline{C}H_2)_2CH_2$), 24.28 (1C, $CH_2\underline{C}H_2CH_2$), 22.97 ($1C^{Piperidine}$, $N(CH_2CH_2)_2\underline{C}H_2$).

Preparation of 1-[3-(4-chlorophenyl)propyl]-2,3-di(*tert*-butoxycarbonyl)-1-[4-(piperidin-1-yl)butyl]guanidine (**23b**)

1,3-bis(*tert*-butoxycarbonyl)-2-methylisothiourea (0.164 g; $5.65\cdot10^{-4}$ mol) and mercury II chloride (0.153 g; $5.65\cdot10^{-4}$ mol) were sequentially added to an ice-cooled mixture of *N*-(3-(4-chlorophenyl)propyl)-4-(piperidin-1-yl)butan-1-amine (**22b**) (0.159 g; $5.15\cdot10^{-4}$ mol) and triethylamine (0.260 g; $2.57\cdot10^{-3}$ mol) in 20 mL DCM. The ice bath was removed and the reaction was stirred for eighteen hours at room temperature, then filtered. The precipitate was discarded. The filtrate was washed sequentially twice with 15 mL H_2O and twice with 15 mL brine.

The combined organic phases were dried over Na_2SO_4, then the solvent was removed under vacuum and the crude product was purified by column chromatography (EtOAc/MeOH/Triethylamine 89:10:1) to yield the pure product.

1-[3-(4-chlorophenyl)propyl]-2,3-di(*tert*-butoxycarbonyl)-1-[4-(piperidin-1-yl)butyl]guanidine (**23b**): $C_{29}H_{47}ClN_4O_4$. M=551.16. Yellowish sticky oil. 78.25 % yield. R_f=0.28 (EtOAc/MeOH/Triethylamine 89:10:1). ^1H NMR (600 MHz, *CDCl₃*) δ ppm 9.79 (br, 1H, $N\underline{H}$), 7.24-7.23 (m, $2H^{arom.}$, $C(CHC\underline{H})_2CCl$), 7.12-7.11 (m, $2H^{arom.}$, $C(C\underline{H}CH)_2CCl$), 3.42 (m, 4H, $C\underline{H}_2NC\underline{H}_2$), 2.59 (m, 2H, $C\underline{H}_2C^{arom.}$), 2.35-2.28 (br, 6H: $4H^{piperidine}$, $N(\underline{C}H_2CH_2)_2CH_2$, $C\underline{H}_2N^{piperidine}$), 1.91 (qt, 2H, $CH_2C\underline{H}_2CH_2C^{arom.}$), 1.58-1.47 (m, 28H: $6H^{piperidine}$, $N(CH_2C\underline{H}_2)_2C\underline{H}_2$; $C\underline{H}_3$; $CH_2C\underline{H}_2C\underline{H}_2CH_2$). ^{13}C NMR (150.95 MHz, *CDCl₃*) δ ppm 162.61 (1C, $\underline{C}$=O), 155.42 ($1C^{quat.}$, $\underline{C}$=N), 150.87 (1C, $\underline{C}$=O), 139.65 ($1C^{quat./arom.}$, $\underline{C}CH_2$), 131.62 ($1C^{quat./arom.}$, $\underline{C}Cl$), 129.70 ($2C^{arom.}$, $C(\underline{C}HCH)_2CCl$), 128.45 ($2C^{arom.}$, $C(CH\underline{C}H)_2CCl$), 81.74($1C^{quat.}$ Boc), 79.17 ($1C^{quat.}$ Boc), 58.76 (1C, $\underline{C}H_2N^{piperidine}$), 54.53 ($2C^{piperidine}$, $N(\underline{C}H_2CH_2)_2CH_2$), 47.75-47.62 (2C, $\underline{C}H_2N\underline{C}H_2$), 32.14 (1C, $\underline{C}H_2C^{arom.}$), 28.69 (1C, $CH_2\underline{C}H_2CH_2C^{arom.}$), 28.21 (3C, $\underline{C}H_3$), 28.08 (3C, $\underline{C}H_3$), 25.87 ($2C^{piperidine}$, $N(CH_2\underline{C}H_2)_2CH_2$), 25.61(1C, $CH_2\underline{C}H_2CH_2CH_2$), 24.37 ($1C^{piperidine}$, $N(CH_2CH_2)_2\underline{C}H_2$), 23.87 (1C, $CH_2\underline{C}H_2CH_2CH_2$).

Preparation of 1-[4-(4-chlorophenyl)butyl]-2,3-di(*tert*-butoxycarbonyl)-1-[3-(piperidin-1-yl)propyl]guanidine (**23c**)

1,3-bis(*tert*-butoxycarbonyl)-2-methylisothiourea (0.221 g; $7.61 \cdot 10^{-4}$ mol) and mercury II chloride (0.207 g; $7.62 \cdot 10^{-4}$ mol) were sequentially added to an ice-cooled mixture of 4-(4-chlorophenyl)-*N*-[3-(piperidin-1-yl)propyl]butan-1-amine (**22c**) (0.214 g; $6.93 \cdot 10^{-4}$ mol) and triethylamine (0.350 g; $3.46 \cdot 10^{-3}$ mol) in 20 mL DCM. The ice bath was removed and the reaction was stirred for eighteen hours at room temperature, then filtered. The precipitate was discarded. The filtrate was washed sequentially twice with 15 mL H_2O and twice with 15 mL brine. The combined organic phases were dried over Na_2SO_4, then the solvent was removed under vacuum and the crude product was purified by column chromatography (DCM/MeOH 20:1) to yield the pure product.

1-[4-(4-chlorophenyl)butyl]-2,3-di(*tert*-butoxycarbonyl)-1-[3-(piperidin-1-yl)propyl]guanidine (**23c**): $C_{29}H_{47}ClN_4O_4$. M=551.16. Colourless sticky oil. 63.64 % yield. R_f=0.35 (DCM/MeOH 20:1). ^1H NMR (600 MHz, *CDCl₃*) δ ppm 10.78 (br, 1H, $N\underline{H}$), 7.23-7.22 (m, $2H^{arom.}$, $C(CHC\underline{H})_2CCl$), 7.12-7.10 (m, $2H^{arom.}$, $C(C\underline{H}CH)_2CCl$), 3.31-3.29 (m, 4H, $C\underline{H}_2NC\underline{H}_2$), 2.60 (t, 2H, $C\underline{H}_2C^{arom.}$, J=7.51Hz), 2.39-2.26 (br, 6H: $4H^{piperidine}$, $N(C\underline{H}_2CH_2)_2CH_2$, $C\underline{H}_2N^{piperidine}$), 1.70-1.64 (m, 8H: $4H^{piperidine}$, $N(CH_2C\underline{H}_2)_2CH_2$; $C\underline{H}_2CH_2N^{piperidine}$, $CH_2C\underline{H}_2CH_2CH_2$), 1.58 (qt, 2H, $CH_2C\underline{H}_2CH_2CH_2$), 1.49-1.48 (m, 20H: $2H^{piperidine}$, $N(CH_2CH_2)_2C\underline{H}_2$; $C\underline{H}_3$). ^{13}C NMR (150.95 MHz, *CDCl₃*) δ ppm 160.89 (1C, $\underline{C}$=O), 151.91 ($1C^{quat.}$, $\underline{C}$=N), 151.62 (1C, $\underline{C}$=O), 140.87 ($1C^{quat./arom.}$, $\underline{C}CH_2$), 131.36 ($1C^{quat./arom.}$, $\underline{C}Cl$), 129.88 ($2C^{arom.}$, $C(\underline{C}HCH)_2CCl$), 128.36 ($2C^{arom.}$, $C(CH\underline{C}H)_2CCl$), 80.77 ($1C^{quat.}$ Boc), 77.99 ($1C^{quat.}$ Boc), 53.99 ($2C^{piperidine}$, $N(\underline{C}H_2CH_2)_2CH_2$), 53.33 (1C, $\underline{C}H_2N^{piperidine}$), 46.39 (1C, $\underline{C}H_2NCH_2$), 44.29 (1C, $\underline{C}H_2NCH_2$), 34.92 (1C, $\underline{C}H_2C^{arom.}$), 28.90 (1C, $CH_2\underline{C}H_2CH_2C^{arom.}$), 28.37 (6C, $\underline{C}H_3$), 26.09 (1C, $\underline{C}H_2CH_2N^{piperidine}$), 24.41, 24.31 ($3C^{piperidine}$, $N(\underline{C}H_2CH_2)_2\underline{C}H_2$), 23.00 (1C, $\underline{C}H_2CH_2CH_2C^{arom.}$).

Preparation of 1-(3-(4-chlorophenyl)propyl)-1-(3-(piperidin-1-yl)propyl)guanidine dihydrochloride (**ADS10292**)

4M solution HCl-dioxan (1.29 mL; $5.16{\cdot}10^{-3}$ mol) was added dropwise to a solution of the 1-(3-(4-chlorophenyl)propyl)-2,3-di(*tert*-butoxycarbonyl)-1-(3-(piperidin-1-yl)propyl)guanidine (**23a**) (0.277 g; $5.16{\cdot}10^{-4}$ mol) in 20 mL chloroform. The reaction was stirred overnight at room temperature, then the solvent was removed under vacuum. The crude product was evaporated twice from chloroform and twice from EtOAc, then recrystallized from anhydrous 2-propanol to yield the pure product.

1-(3-(4-chlorophenyl)propyl)-1-(3-(piperidin-1-yl)propyl)guanidine dihydrochloride (**ADS10292**): $C_{18}H_{29}ClN_4{\cdot}2HCl$. M=409.82. White solid. 75.51 %. mp: 248.0-250.0 °C with decomposition. ^{1}H NMR (600 MHz, *CD_3OD*) δ ppm 7.32-7.31 (m, $2H^{arom.}$, $C(CHC\underline{H})_2CCl$), 7.28-7.27 (m, $2H^{arom.}$, $C(C\underline{H}CH)_2CCl$), 3.57-3.55 (m, $2H^{Piperidine}$), 3.47-3.44 (m, 4H, $C\underline{H}_2NC\underline{H}_2$), 3.15-3.12 (m, 2H, $C\underline{H}_2N^{piperidine}$), 3.00-2.96 (m, $2H^{Piperidine}$), 2.71-2.68 (m, 2H, $ClPhC\underline{H}_2CH_2$), 2.14-2.09 (m, 2H, $C\underline{H}_2CH_2N^{piperidine}$), 2.00-1.96 (m, 4H: $2H^{Piperidine}$; $ClPhCH_2C\underline{H}_2$), 1.90-1.86 (m, $3H^{Piperidine}$), 1.56-1.55 (m, $1H^{Piperidine}$). ^{13}C NMR (150.95 MHz, *CD_3OD*) δ ppm 157.77 (1C, $\underline{C}$=N), 141.22 ($1C^{quat./arom.}$, $\underline{C}(CHCH)_2CH$), 132.96 ($1C^{quat./arom.}$, $\underline{C}Cl$), 131.04 ($2C^{arom.}$, $C(\underline{C}HCH)_2CCl$), 129.62 ($2C^{arom.}$, $C(CH\underline{C}H)_2CCl$), 55.02 (1C, $\underline{C}H_2N^{piperidine}$), 54.61 ($2C^{Piperidine}$, $N(\underline{C}H_2CH_2)_2CH_2$), 49.70 (1C, $\underline{C}H_2N$), 47.07 (1C, $\underline{C}H_2N$), 32.83 (1C, $ClPh\underline{C}H_2$), 29.92 (1C, $ClPhCH_2\underline{C}H_2$), 24.27 ($2C^{Piperidine}$), 23.20 (1C, $\underline{C}H_2CH_2N^{piperidine}$), 22.70 ($1C^{Piperidine}$). Anal. Calcd: C 52.75 %; H 7.62 %; N 13.67 %. Found: C 52.64 %; H 8.00 %; N 13.34 %.

Preparation of 1-[3-(4-chlorophenyl)propyl]-1-[4-(piperidin-1-yl)butyl]guanidine dihydrochloride (**ADS10300**)

4M solution HCl-dioxan (2.01 mL; $8.06{\cdot}10^{-3}$ mol) was added dropwise to a solution of the 1-[3-(4-chlorophenyl)propyl]-2,3-di(*tert*-butoxycarbonyl)-1-[4-(piperidin-1-yl)butyl]guanidine (**23b**) (0.277 g; $4.03{\cdot}10^{-4}$ mol) in 20 mL chloroform. The reaction was stirred overnight at room temperature, then the solvent was removed under vacuum. The crude product was evaporated twice from chloroform and twice from EtOAc, then recrystallized from anhydrous 2-propanol to yield the pure product.

1-[3-(4-chlorophenyl)propyl]-1-[4-(piperidin-1-yl)butyl]guanidine dihydrochloride (**ADS10300**): $C_{19}H_{31}ClN_4{\cdot}2HCl{\cdot}0.5H_2O$. M=432.86. White solid. 33.06 %. mp: 187.8-189.4 °C with decomposition. ^{1}H NMR (600 MHz, *CD_3OD*) δ ppm 7.32-7.30 (m, $2H^{arom.}$, $C(CHC\underline{H})_2CCl$), 7.27-7.25 (m, $2H^{arom.}$, $C(C\underline{H}CH)_2CCl$), 3.55 (br, $2H^{Piperidine}$), 3.44-3.38 (m, 4H, $C\underline{H}_2NC\underline{H}_2$), 3.13-3.11 (m, 2H, $C\underline{H}_2N^{piperidine}$), 2.95 (br, $2H^{Piperidine}$), 2.69-2.67 (m, 2H, $ClPhC\underline{H}_2CH_2$), 1.99-1.93 (m, 4H: $ClPhCH_2C\underline{H}_2$, $CH_2C\underline{H}_2CH_2$), 1.87-1.77 (m, $5H^{Piperidine}$), 1.71-1.66 (m, 2H, $CH_2C\underline{H}_2CH_2$), 1.56 (br, $1H^{Piperidine}$). ^{13}C NMR (150.95 MHz, *CD_3OD*) δ ppm 157.67 (1C, $\underline{C}$=N), 141.25 ($1C^{quat./arom.}$, $\underline{C}(CHCH)_2CH$), 132.97 ($1C^{quat./arom.}$, $\underline{C}Cl$), 131.04 ($2C^{arom.}$, $C(\underline{C}HCH)_2CCl$), 129.63 ($2C^{arom.}$, $C(CH\underline{C}H)_2CCl$), 57.75 (1C, $\underline{C}H_2N^{piperidine}$), 54.41 ($2C^{Piperidine}$, $N(\underline{C}H_2CH_2)_2CH_2$), 49.63 (2C, $\underline{C}H_2N$), 32.84 (1C, $ClPh\underline{C}H_2$), 29.96 (1C, $ClPhCH_2\underline{C}H_2$), 25.51 (1C, $CH_2\underline{C}H_2CH_2$), 24.26 ($2C^{Piperidine}$), 22.78 ($1C^{Piperidine}$), 22.15 (1C, $CH_2\underline{C}H_2CH_2$). Anal. Calcd: C 52.72 %; H 7.92 %; N 12.94 %. Found: C 53.02 %; H 8.16 %; N 12.95 %.

Preparation of 1-[4-(4-chlorophenyl)butyl]-1-[3-(piperidin-1-yl)propyl]guanidine dihydrochloride (**ADS10312**)

4M solution HCl-dioxan (2.35 mL; $9.39{\cdot}10^{-3}$ mol) was added dropwise to a solution of the 1-[4-(4-chlorophenyl)butyl]-2,3-di(*tert*-butoxycarbonyl)-1-[3-(piperidin-1-yl)propyl]guanidine (**23c**) (0.259 g; $4.69{\cdot}10^{-4}$

mol) in 20 mL chloroform. The reaction was stirred overnight at room temperature, then the solvent was removed under vacuum. The crude product was evaporated twice from chloroform and twice from EtOAc, then recrystallized from anhydrous 2-propanol to yield the pure product.

1-[4-(4-chlorophenyl)butyl]-1-[3-(piperidin-1-yl)propyl]guanidine dihydrochloride (**ADS10312**): $C_{19}H_{31}ClN_4 \cdot 2HCl \cdot H_2O$. M=441.87. White solid. 93.13 %. mp: 171.4-173.4 °C with decomposition. ^{1}H NMR (600 MHz, *CD_3OD*) δ ppm 7.30-7.28 (m, $2H^{arom.}$, $C(CH\underline{C}\underline{H})_2CCl$), 7.23-7.22 (m, $2H^{arom.}$, $C(C\underline{H}CH)_2CCl$), 3.57-3.55 (m, $2H^{Piperidine}$), 3.46-3.40 (m, 4H, $C\underline{H}_2NC\underline{H}_2$), 3.15-3.12 (m, 2H, $C\underline{H}_2N^{piperidine}$), 3.00-2.96 (m, $2H^{Piperidine}$), 2.71-2.68 (m, 2H, $ClPhC\underline{H}_2CH_2$), 2.15-2.09 (m, 2H, $CH_2C\underline{H}_2CH_2$), 1.98-1.86 (m, $5H^{Piperidine}$), 1.70-1.67 (m, 4H, $CH_2C\underline{H}_2CH_2$), 1.59-1.51 (m, $1H^{Piperidine}$). ^{13}C NMR (150.95 MHz, *CD_3OD*) δ ppm 157.69 (1C, $\underline{C}$=N), 142.08 ($1C^{quat./arom.}$, $\underline{C}(CHCH)_2CH$), 132.71 ($1C^{quat./arom.}$, $\underline{C}Cl$), 131.12 ($2C^{arom.}$, $C(\underline{C}HCH)_2CCl$), 129.47 ($2C^{arom.}$, $C(CH\underline{C}H)_2CCl$), 55.03 (1C, $\underline{C}H_2N^{piperidine}$), 54.60 ($2C^{Piperidine}$, $N(\underline{C}H_2CH_2)_2CH_2$), 49.95 (1C, $\underline{C}H_2N$), 47.08 (1C, $\underline{C}H_2N$), 35.81 (1C, $ClPh\underline{C}H_2$), 29.18 (1C, $CH_2\underline{C}H_2CH_2$), 27.91 (1C, $CH_2\underline{C}H_2CH_2$), 24.27 ($2C^{Piperidine}$), 23.21 (1C, $CH_2\underline{C}H_2CH_2$), 22.70 ($1C^{Piperidine}$). Anal. Calcd: C 51.65 %; H 7.98 %; N 12.68 %. Found: C 51.96 %; H 7.98 %; N 12.68 %.

Preparation of 1,3-bis(*tert*-butoxycarbonyl)-1-[3-(4-chlorophenyl)propyl]-2-methylisothiourea (**24a**)

Triphenylphosphine (0.507 g; $1.93 \cdot 10^{-3}$ mol) and 1,3-bis(*tert*-butoxycarbonyl)-2-methylisothiourea (0.255 g; $8.78 \cdot 10^{-4}$ mol) were sequentially added to a solution of 3-(4-Chlorophenyl)-1-propanol (0.30 g; $1.76 \cdot 10^{-3}$ mol) in 5 mL dry THF under an argon atmosphere. Then, 94 % diisopropyl azodicarboxylate (DIAD) (0.416 g; $1.93 \cdot 10^{-3}$ mol) was added dropwise. The reaction was stirred overnight at room temperature. The solvent was removed under vacuum and the crude product was purified by column chromatography (Hexane/EtOAc 15:1) to yield the pure product.

1,3-bis(*tert*-butoxycarbonyl)-1-[3-(4-chlorophenyl)propyl]-2-methylisothiourea (**24a**): $C_{21}H_{31}ClN_2O_4S$. M=443.00. Colourless sticky oil. 92.80 % yield. R_f=0.48 (Hexane/EtOAc 15:1). ^{1}H NMR (600 MHz, *$CDCl_3$*) δ ppm 7.25-7.23 (m, $2H^{arom.}$, $C(CHC\underline{H})_2CCl$), 7.13-7.12 (m, $2H^{arom.}$, $C(C\underline{H}CH)_2CCl$), 3.55-3.52 (m, 2H, $C\underline{H}_2N$, *J*=7.50Hz), 2.60 (t, 2H, $C\underline{H}_2C^{arom.}$, *J*=7.78Hz), 2.38 (s, 3H, $SC\underline{H}_3$), 1.97 (qt, 2H, $CH_2C\underline{H}_2CH_2$), 1.50 (s, 9H, $CC\underline{H}_3$), 1.46 (s, 9H, $CC\underline{H}_3$). ^{13}C NMR (150.95 MHz, *$CDCl_3$*) δ ppm 162.82 (1C, $\underline{C}$=O), 157.83 (1C, $\underline{C}$=O), 151.78 ($1C^{quat.}$, $\underline{C}$=N), 139.74 ($1C^{quat./arom.}$, $\underline{C}CH_2$), 131.62 ($1C^{quat./arom.}$, $\underline{C}Cl$), 129.71 ($2C^{arom.}$, $C(\underline{C}HCH)_2CCl$), 128.45 ($2C^{arom.}$, $C(CH\underline{C}H)_2CCl$), 82.32 ($1C^{quat.}$ Boc), 81.85 ($1C^{quat.}$ Boc), 48.40 (1C, $\underline{C}H_2N$), 32.39 (1C, $\underline{C}H_2C^{arom.}$), 30.29 (1C, $CH_2\underline{C}H_2CH_2$), 28.05 (3C, $C\underline{C}H_3$), 27.99 (3C, $C\underline{C}H_3$), 15.58 (1C, $S\underline{C}H_3$).

Preparation of 1,3-bis(*tert*-butoxycarbonyl)-1-[4-(4-chlorophenyl)butyl]-2-methylisothiourea (**24b)**

Triphenylphosphine (1.25 g; $4.76 \cdot 10^{-3}$ mol) and 1,3-bis(*tert*-butoxycarbonyl)-2-methylisothiourea (0.629 g; $2.17 \cdot 10^{-3}$ mol) were sequentially added to a solution of 4-(4-chlorophenyl)butan-1-ol (**20**) (0.80 g; $4.33 \cdot 10^{-3}$ mol) in 12 mL dry THF under an argon atmosphere. Then, 94 % diisopropyl azodicarboxylate (DIAD) (1.02 g; $4.76 \cdot 10^{-3}$ mol) was added dropwise. The reaction was stirred overnight at room temperature. The solvent was removed

under vacuum and the crude product was purified by column chromatography (Hexane/EtOAc 15:1) to yield the pure product.

1,3-bis(*tert*-butoxycarbonyl)-1-[4-(4-chlorophenyl)butyl]-2-methylisothiourea (**24b**): $C_{22}H_{33}ClN_2O_4S$. M=457.03. Colourless sticky oil. 93.63 % yield. R_f=0.52 (Hexane/EtOAc 15:1). ^{1}H NMR (600 MHz, *CDCl3*) δ ppm 7.23-7.22 (m, 2H$^{arom.}$, $C(CHC\underline{H})_2CCl$), 7.10-7.09 (m, 2H$^{arom.}$, $C(C\underline{H}CH)_2CCl$), 3.53-3.51 (m, 2H, $C\underline{H}_2N$), 2.60 (t, 2H, $C\underline{H}_2C^{arom}$), 2.37 (s, 3H, $SC\underline{H}_3$), 1.70 (qt, 2H, $C\underline{H}_2CH_2N$), 1.60 (qt, 2H, $C\underline{H}_2CH_2C^{arom.}$), 1.49 (s, 9H, $CC\underline{H}_3$), 1.46 (s, 9H, $CC\underline{H}_3$). ^{13}C NMR (150.95 MHz, *CDCl3*) δ ppm 162.78 (1C, $\underline{C}$=O), 157.84 (1C, $\underline{C}$=O), 151.87 (1C$^{quat.}$, $\underline{C}$=N), 140.59 (1C$^{quat./arom.}$, $\underline{C}CH_2$), 131.45 (1C$^{quat./arom.}$, $\underline{C}Cl$), 129.71 (2C$^{arom.}$, $C(\underline{C}HCH)_2CCl$), 128.36 (2C$^{arom.}$, $C(CH\underline{C}H)_2CCl$), 82.19 (1C$^{quat.}$ Boc), 81.79 (1C$^{quat.}$ Boc), 48.62 (1C, $\underline{C}H_2N$), 34.75 (1C, $\underline{C}H_2C^{arom.}$), 28.43 (1C, $\underline{C}H_2CH_2C^{arom.}$), 28.35 (1C, $\underline{C}H_2CH_2N$), 28.04 (3C, $C\underline{C}H_3$), 27.99 (3C, $C\underline{C}H_3$), 15.56 (1C, $S\underline{C}H_3$).

Preparation of 1-[3-(4-chlorophenyl)propyl]-1,2-di(*tert*-butoxycarbonyl)-3-[3-(piperidin-1-yl)propyl]guanidine (**25a**)

1,3-bis(*tert*-butoxycarbonyl)-1-[3-(4-chlorophenyl)propyl]-2-methylisothiourea (**24a**) (0.16 g; $3.61\cdot10^{-4}$ mol) was added to a mixture of 3-(piperidin-1-yl)propan-1-amine (**18a**) (0.103 g; $7.24\cdot10^{-4}$ mol) in 8 mL THF and 1 mL water. The reaction was stirred overnight at 70 °C. Then, 10 mL EtOAc and 10 mL water were added. The organic phase was washed with 10 mL brine and dried over Na_2SO_4. The solvent was removed under vacuum and the crude product was purified by column chromatography (DCM/MeOH/25% NH_3aq. 189:10:1) to yield the pure product.

1-[3-(4-chlorophenyl)propyl]-1,2-di(*tert*-butoxycarbonyl)-3-[3-(piperidin-1-yl)propyl]guanidine (**25a**): $C_{28}H_{45}ClN_4O_4$. M=537.13. Yellowish sticky oil. 81.44 % yield. R_f=0.81 (DCM/MeOH/25% NH_3aq. 189:10:1). ^{1}H NMR (600 MHz, *CDCl3*) δ ppm 9.84 (br, 1H, $N\underline{H}$), 7.24-7.22 (m, 2H$^{arom.}$, $C(CHC\underline{H})_2CCl$), 7.12-7.10 (m, 2H$^{arom.}$, $C(C\underline{H}CH)_2CCl$), 3.64 (t, 2H, $C\underline{H}_2NBoc$, *J*=7.18Hz), 3.31 (t, 2H, $C\underline{H}_2NH$, *J*=5.89Hz), 2.59 (t, 2H, $C\underline{H}_2C^{arom.}$, *J*=7.75Hz), 2.35 (br, 6H: 4H^{piperidine}, $N(\underline{C}H_2CH_2)_2CH_2$, $C\underline{H}_2N^{piperidine}$), 1.87 (m, 2H, $CH_2C\underline{H}_2CH_2C^{arom.}$), 1.75-1.73 (m, 2H, $CH_2C\underline{H}_2CH_2N^{piperidine}$), 1.55 (m, 4H^{piperidine}, $N(CH_2C\underline{H}_2)_2CH_2$), 1.49 (s, 9H, $CC\underline{H}_3$), 1.45 (s, 9H, $CC\underline{H}_3$), 1.41 (m, 2H^{piperidine}, $N(CH_2CH_2)_2C\underline{H}_2$). ^{13}C NMR (150.95 MHz, *CDCl3*) δ ppm 163.62 (1C, $\underline{C}$=O), 160.83 (1C, $\underline{C}$=O), 152.88 (1C$^{quat.}$, $\underline{C}$=N), 139.79 (1C$^{quat./arom.}$, $\underline{C}CH_2$), 131.44 (1C$^{quat./arom.}$, $\underline{C}Cl$), 129.58 (2C$^{arom.}$, $C(\underline{C}HCH)_2CCl$), 128.95 (2C$^{arom.}$, $C(CH\underline{C}H)_2CCl$), 81.84 (1C$^{quat.}$ Boc), 79.00 (1C$^{quat.}$ Boc), 56.97 (1C, $\underline{C}H_2N^{piperidine}$), 54.49 (2C^{piperidine}, $N(\underline{C}H_2CH_2)_2CH_2$), 47.07 (1C, $\underline{C}H_2NBoc$), 42.89 (1C, $\underline{C}H_2NH$), 32.38 (1C, $\underline{C}H_2C^{arom.}$), 30.44 (1C, $CH_2\underline{C}H_2CH_2C^{arom.}$), 28.13 (3C, $C\underline{C}H_3$), 28.07 (3C, $C\underline{C}H_3$), 25.63 (3C: 2C^{piperidine}, $N(CH_2\underline{C}H_2)_2CH_2$; $CH_2\underline{C}H_2CH_2N^{piperidine}$), 24.27 (1C^{piperidine}, $N(CH_2CH_2)_2\underline{C}H_2$).

Preparation of 1-[3-(4-chlorophenyl)propyl]-1,2-di(*tert*-butoxycarbonyl)-3-[4-(piperidin-1-yl)butyl]guanidine (**25b**)

1,3-bis(*tert*-butoxycarbonyl)-1-[3-(4-chlorophenyl)propyl]-2-methylisothiourea (**24a**) (0.205 g; $4.63\cdot10^{-4}$ mol) was added to a mixture of 4-(piperidin-1-yl)butan-1-amine (**18b**) (0.144 g; $9.21\cdot10^{-4}$ mol) in 12 mL THF and 1.5

mL water. The reaction was stirred overnight at 70 °C. Then, 10 mL EtOAc and 10 mL water were added. The organic phase was washed with 10 mL brine and dried over Na_2SO_4. The solvent was removed under vacuum and the crude product was purified by column chromatography (DCM/MeOH/25% NH_3aq. 189:10:1) to yield the pure product.

1-[3-(4-chlorophenyl)propyl]-1,2-di(*tert*-butoxycarbonyl)-3-[4-(piperidin-1-yl)butyl]guanidine (**25b**): $C_{29}H_{47}ClN_4O_4$. M=551.16. Yellowish sticky oil. 76.37 % yield. R_f=0.75 (DCM/MeOH/25% NH_3aq. 189:10:1). ^{1}H NMR (600 MHz, *CDCl₃*) δ ppm 9.78 (br, 1H, $N\underline{H}$), 7.24-7.23 (m, $2H^{arom.}$, $C(CHC\underline{H})_2CCl$), 7.11-7.10 (m, $2H^{arom.}$, $C(C\underline{H}CH)_2CCl$), 3.67 (t, 2H, $C\underline{H}_2NBoc$, J=7.80Hz), 3.24 (t, 2H, $C\underline{H}_2NH$), 2.58 (t, 2H, $C\underline{H}_2C^{arom.}$, J=7.73Hz), 2.33 (br, 4H: $4H^{piperidine}$, $N(\underline{C}H_2CH_2)_2CH_2$), 2.28 (t, 2H, $C\underline{H}_2N^{piperidine}$, J=7.50Hz), 1.86 (qt, 2H, $CH_2C\underline{H}_2CH_2C^{arom.}$), 1.62 (qt, 2H, $CH_2C\underline{H}_2CH_2CH_2$), 1.58-1.39 (m, 26H: $6H^{piperidine}$, $N(CH_2C\underline{H}_2)_2C\underline{H}_2$; $CH_2C\underline{H}_2CH_2CH_2$, $CC\underline{H}_3$). ^{13}C NMR (150.95 MHz, *CDCl₃*) δ ppm 163.88 (1C, $\underline{C}$=O), 160.95 (1C, $\underline{C}$=O), 152.95 ($1C^{quat.}$, $\underline{C}$=N), 139.77 ($1C^{quat./arom.}$, $\underline{C}CH_2$), 131.55 ($1C^{quat./arom.}$, $\underline{C}Cl$), 129.61 ($2C^{arom.}$, $C(\underline{C}HCH)_2CCl$), 128.39 ($2C^{arom.}$, $C(CH\underline{C}H)_2CCl$), 82.19 ($1C^{quat.}$ Boc), 79.16 ($1C^{quat.}$ Boc), 58.62 (1C, $\underline{C}H_2N^{piperidine}$), 54.49 ($2C^{piperidine}$, $N(\underline{C}H_2CH_2)_2CH_2$), 47.11 (1C, $\underline{C}H_2NBoc$), 43.73 (1C, $\underline{C}H_2NH$), 32.42 (1C, $\underline{C}H_2C^{arom.}$), 30.51 (1C, $CH_2\underline{C}H_2CH_2C^{arom.}$), 28.18 (3C, $C\underline{C}H_3$), 28.11(3C, $C\underline{C}H_3$), 27.37 (1C, $CH_2\underline{C}H_2CH_2CH_2$), 25.82 ($2C^{piperidine}$, $N(CH_2\underline{C}H_2)_2CH_2$), 24.32 ($1C^{piperidine}$, $N(CH_2CH_2)_2\underline{C}H_2$), 24.28 (1C, $CH_2\underline{C}H_2CH_2CH_2$).

Preparation of 1-[4-(4-chlorophenyl)butyl]-1,2-di(*tert*-butoxycarbonyl)-3-[3-(piperidin-1-yl)propyl]guanidine (**25c**)

1,3-bis(*tert*-butoxycarbonyl)-1-[4-(4-chlorophenyl)butyl]-2-methylisothiourea (**24b)** (0.40 g; $8.75\cdot10^{-4}$ mol) was added to a mixture of 3-(piperidin-1-yl)propan-1-amine (**18a**) (0.249 g; $1.75\cdot10^{-3}$ mol) in 20 mL THF and 2.5 mL water. The reaction was stirred overnight at 70 °C. Then, 15 mL EtOAc and 15 mL water were added. The organic phase was washed with 15 mL brine and dried over Na_2SO_4. The solvent was removed under vacuum and the crude product was purified by column chromatography (EtOAc/MeOH/Triethylamine 89:10:1) to yield the pure product.

1-[4-(4-chlorophenyl)butyl]-1,2-di(*tert*-butoxycarbonyl)-3-[3-(piperidin-1-yl)propyl]guanidine (**25c**): $C_{29}H_{47}ClN_4O_4$. M=551.16. Yellowish sticky oil. 75.31 % yield. R_f=0.43 (EtOAc/MeOH/Triethylamine 89:10:1). ^{1}H NMR (600 MHz, *CDCl₃*) δ ppm 9.78 (br, 1H, $N\underline{H}$), 7.23-7.21 (m, $2H^{arom.}$, $C(CHC\underline{H})_2CCl$), 7.09-7.08 (m, $2H^{arom.}$, $C(C\underline{H}CH)_2CCl$), 3.63 (br, 2H, $C\underline{H}_2NBoc$), 3.29 (t, 2H, $C\underline{H}_2NH$, J=6.08Hz), 2.59 (t, 2H, $C\underline{H}_2C^{arom.}$, J=6.84Hz), 2.34 (br, 6H: $4H^{piperidine}$, $N(\underline{C}H_2CH_2)_2CH_2$, $C\underline{H}_2N^{piperidine}$), 1.72 (qt, 2H, $N^{piperidine}CH_2C\underline{H}_2$), 1.59-1.56 (m, 8H: $4H^{piperidine}$, $N(CH_2C\underline{H}_2)_2CH_2$; $CH_2C\underline{H}_2C\underline{H}_2CH_2C^{arom.}$), 1.49 (s, 9H, $CC\underline{H}_3$), 1.45 (s, 9H, $CC\underline{H}_3$), 1.44-1.43 (m, $2H^{piperidine}$, $N(CH_2CH_2)_2C\underline{H}_2$). ^{13}C NMR (150.95 MHz, *CDCl₃*) δ ppm 164.50 (1C, $\underline{C}$=O), 160.85 (1C, $\underline{C}$=O), 152.97 ($1C^{quat.}$, $\underline{C}$=N), 140.52 ($1C^{quat./arom.}$, $\underline{C}CH_2$), 131.32 ($1C^{quat./arom.}$, $\underline{C}Cl$), 129.62 ($2C^{arom.}$, $C(\underline{C}HCH)_2CCl$), 128.23 ($2C^{arom.}$, $C(CH\underline{C}H)_2CCl$), 82.70 ($1C^{quat.}$ Boc), 78.94 ($1C^{quat.}$ Boc), 58.95 (1C, $\underline{C}H_2N^{piperidine}$), 54.50 ($2C^{piperidine}$, $N(\underline{C}H_2CH_2)_2CH_2$), 47.21 (1C, $\underline{C}H_2NBoc$), 42.92(1C, $\underline{C}H_2NH$), 34.58 (1C, $\underline{C}H_2C^{arom.}$), 28.43 (1C, $\underline{C}H_2CH_2C^{arom.}$), 28.37 (1C, $\underline{C}H_2CH_2CH_2C^{arom.}$), 28.14 (3C, $C\underline{C}H_3$), 28.09 (3C, $C\underline{C}H_3$), 25.66 (3C: $2C^{piperidine}$, $N(CH_2\underline{C}H_2)_2CH_2$; $N^{piperidine}CH_2\underline{C}H_2$), 24.29 ($1C^{piperidine}$, $N(CH_2CH_2)_2\underline{C}H_2$).

Preparation of 1-[4-(4-chlorophenyl)butyl]-1,2-di(*tert*-butoxycarbonyl)-3-[4-(piperidin-1-yl)butyl]guanidine (**25d**)

1,3-bis(*tert*-butoxycarbonyl)-1-[4-(4-chlorophenyl)butyl]-2-methylisothiourea (**24b)** (0.505 g; $1.10\cdot10^{-3}$ mol) was added to a mixture of 4-(piperidin-1-yl)butan-1-amine (**18b**) (0.345 g; $2.21\cdot10^{-3}$ mol) in 25 mL THF and 3.12 mL water. The reaction was stirred overnight at 70 °C. Then, 20 mL EtOAc and 20 mL water were added. The organic phase was washed with 20 mL brine and dried over Na_2SO_4. The solvent was removed under vacuum and the crude product was purified by column chromatography (EtOAc/MeOH/Triethylamine 89:10:1) to yield the pure product.

1-[4-(4-chlorophenyl)butyl]-1,2-di(*tert*-butoxycarbonyl)-3-[4-(piperidin-1-yl)butyl]guanidine (**25d**): $C_{30}H_{49}ClN_4O_4$. M=565.19. Colourless sticky oil. 82.21 % yield. R_f=0.28 (EtOAc/MeOH/Triethylamine 89:10:1). ^{1}H NMR (600 MHz, *CDCl₃*) δ ppm 9.77 (br, 1H, N<u>H</u>), 7.23-7.22 (m, 2H$^{arom.}$, C(CHC<u>H</u>)$_2$CCl), 7.09-7.08 (m, 2H$^{arom.}$, C(C<u>H</u>CH)$_2$CCl), 3.67 (t, 2H, C<u>H</u>$_2$NBoc), 3.22 (t, 2H, C<u>H</u>$_2$NH), 2.59 (t, 2H, C<u>H</u>$_2$C$^{arom.}$, *J*=6.78Hz), 2.34 (br, 4H: 4H^{piperidine}, N(<u>C</u>H$_2$CH$_2$)$_2$CH$_2$), 2.28 (t, 2H, C<u>H</u>$_2$N^{piperidine}), 1.59-1.44 (m, 32H: 6H^{piperidine}, N(CH$_2$C<u>H</u>$_2$)$_2$C<u>H</u>$_2$; CH$_2$C<u>H</u>$_2$C<u>H</u>$_2$CH$_2$C$^{arom.}$; N^{piperidine}CH$_2$C<u>H</u>$_2$C<u>H</u>$_2$CH$_2$, CC<u>H</u>$_3$). ^{13}C NMR (150.95 MHz, *CDCl₃*) δ ppm 163.96 (1C, <u>C</u>=O), 160.97 (1C, <u>C</u>=O), 152.75 (1C$^{quat.}$, <u>C</u>=N), 140.51 (1C$^{quat./arom.}$, <u>C</u>CH$_2$), 131.37 (1C$^{quat./arom.}$, <u>C</u>Cl), 129.65 (2C$^{arom.}$, C(<u>C</u>HCH)$_2$CCl), 128.30 (2C$^{arom.}$, C(CH<u>C</u>H)$_2$CCl), 82.04 (1C$^{quat.}$ Boc), 79.08 (1C$^{quat.}$ Boc), 58.61 (1C, <u>C</u>H$_2$N^{piperidine}), 54.51 (2C^{piperidine}, N(<u>C</u>H$_2$CH$_2$)$_2$CH$_2$), 47.18 (1C, <u>C</u>H$_2$NBoc), 43.17 (1C, <u>C</u>H$_2$NH), 34.63 (1C, <u>C</u>H$_2$C$^{arom.}$), 28.47 (2C, CH$_2$<u>C</u>H$_2$C<u>H</u>$_2$CH$_2$), 28.17 (3C, C<u>C</u>H$_3$), 28.09 (3C, C<u>C</u>H$_3$), 27.37 (1C, CH$_2$<u>C</u>H$_2$CH$_2$CH$_2$), 25.86 (2C^{piperidine}, N(CH$_2$<u>C</u>H$_2$)$_2$CH$_2$), 24.35 (1C^{piperidine}, N(CH$_2$CH$_2$)$_2$<u>C</u>H$_2$), 24.32 (1C, CH$_2$<u>C</u>H$_2$CH$_2$CH$_2$).

Preparation of 1-[3-(4-chlorophenyl)propyl]-3-[3-(piperidin-1-yl)propyl]guanidine dihydrochloride (**ADS10298**)

4M solution HCl-dioxan (1.47 mL; $5.88\cdot10^{-3}$ mol) was added dropwise to a solution of the 1-[3-(4-chlorophenyl)propyl]-1,2-di(*tert*-butoxycarbonyl)-3-[3-(piperidin-1-yl)propyl]guanidine (**25a**) (0.158 g; $2.94\cdot10^{-4}$ mol) in 15 mL chloroform. The reaction was stirred overnight at room temperature, then the solvent was removed under vacuum. The crude product was evaporated twice from chloroform and twice from EtOAc, then recrystallized from anhydrous 2-propanol to yield the pure product.

1-[3-(4-chlorophenyl)propyl]-3-[3-(piperidin-1-yl)propyl]guanidine dihydrochloride (**ADS10298**): $C_{18}H_{29}ClN_4\cdot2HCl$. M=409.82. White solid. 65.83 %. mp: 152.0-153.0 °C. ^{1}H NMR (600 MHz, *CD₃OD*) δ ppm 7.31-7.30 (m, 2H$^{arom.}$, C(CHC<u>H</u>)$_2$CCl), 7.26-7.25 (m, 2H$^{arom.}$, C(C<u>H</u>CH)$_2$CCl), 3.59-3.57 (m, 2H^{Piperidine}), 3.37-3.35 (m, 2H, C<u>H</u>$_2$N), 3.26 (t, 2H, C<u>H</u>$_2$N, *J*=7.11Hz), 3.21-3.18 (m, 2H, C<u>H</u>$_2$N^{piperidine}), 3.01-2.97 (m, 2H^{Piperidine}), 2.74-2.71 (m, 2H, ClPhC<u>H</u>$_2$CH$_2$, *J*=7.80Hz), 2.12-2.07 (m, 2H, C<u>H</u>$_2$CH$_2$N^{piperidine}), 1.97-1.87 (m, 7H: 5H^{Piperidine}; ClPhCH$_2$C<u>H</u>$_2$), 1.57-1.55 (m, 1H^{Piperidine}). ^{13}C NMR (150.95 MHz, *CD₃OD*) δ ppm 157.54 (1C, <u>C</u>=N), 141.24 (1C$^{quat./arom.}$, <u>C</u>(CHCH)$_2$CH), 132.93 (1C$^{quat./arom.}$, <u>C</u>Cl), 131.12 (2C$^{arom.}$, C(<u>C</u>HCH)$_2$CCl), 129.59 (2C$^{arom.}$, C(CH<u>C</u>H)$_2$CCl), 55.47 (1C, <u>C</u>H$_2$N^{piperidine}), 54.54 (2C^{Piperidine}, N(<u>C</u>H$_2$CH$_2$)$_2$CH$_2$), 42.18 (1C, <u>C</u>H$_2$N), 39.85 (1C, <u>C</u>H$_2$N), 33.06 (1C, ClPh<u>C</u>H$_2$), 31.51 (1C, ClPhCH$_2$<u>C</u>H$_2$), 24.87 (1C, <u>C</u>H$_2$CH$_2$N^{piperidine}), 24.28 (2C^{Piperidine}), 22.72 (1C^{Piperidine}). Anal. Calcd: C 52.75 %; H 7.62 %; N 13.67 %. Found: C 52.69 %; H 8.01 %; N 13.59 %.

Preparation of 1-[3-(4-chlorophenyl)propyl]-3-[4-(piperidin-1-yl)butyl]guanidine dihydrochloride (**ADS10301**)

4M solution HCl-dioxan (1.61 mL; $6.46{\cdot}10^{-3}$ mol) was added dropwise to a solution of the 1-[3-(4-chlorophenyl)propyl]-1,2-di(*tert*-butoxycarbonyl)-3-[4-(piperidin-1-yl)butyl]guanidine (**25b**) (0.178 g; $3.23{\cdot}10^{-4}$ mol) in 10 mL chloroform. The reaction was stirred overnight at room temperature, then the solvent was removed under vacuum. The crude product was evaporated twice from chloroform and twice from EtOAc. The residue was dissolved in 2 mL water and freeze-dried to yield the pure product as a salt.

1-[3-(4-chlorophenyl)propyl]-3-[4-(piperidin-1-yl)butyl]guanidine dihydrochloride (**ADS10301**): $C_{19}H_{31}ClN_4{\cdot}2HCl{\cdot}0.5H_2O$. M=432.86. Sticky flakes/oil. 90.84 %. ^{1}H NMR (600 MHz, *CD$_3$OD*) δ ppm 7.31-7.29 (m, 2H$^{arom.}$, $C(CHC\underline{H})_2CCl$), 7.25-7.24 (m, 2H$^{arom.}$, $C(C\underline{H}CH)_2CCl$), 3.39 (br, 2H^{Piperidine}), 3.29-3.27 (m, 2H, $C\underline{H}_2N$, *J*=7.00Hz), 3.25 (t, 2H, $C\underline{H}_2N$, *J*=7.05Hz), 3.13-3.11 (m, 2H, $C\underline{H}_2N^{piperidine}$, *J*=8.01Hz), 3.16 (br, 2H^{Piperidine}), 2.73-2.71 (m, 2H, $ClPhC\underline{H}_2CH_2$, *J*=7.80Hz), 1.96-1.83 (m, 9H: 5H^{Piperidine}; $ClPhCH_2C\underline{H}_2$, $CH_2C\underline{H}_2CH_2$), 1.73-1.65 (m, 3H: 1H^{Piperidine}, $CH_2C\underline{H}_2CH_2$). ^{13}C NMR (150.95 MHz, *CD$_3$OD*) δ ppm 157.55 (1C, $\underline{C}$=N), 141.25 (1C$^{quat./arom.}$, $\underline{C}(CHCH)_2CH$), 132.95 (1C$^{quat./arom.}$, $\underline{C}Cl$), 131.10 (2C$^{arom.}$, $C(\underline{C}HCH)_2CCl$), 129.60 (2C$^{arom.}$, $C(CH\underline{C}H)_2CCl$), 57.75 (1C, $\underline{C}H_2N^{piperidine}$), 54.43 (2C^{Piperidine}, $N(\underline{C}H_2CH_2)_2CH_2$), 42.12 (1C, $\underline{C}H_2N$), 41.39 (1C, $\underline{C}H_2N$), 30.08 (1C, $ClPh\underline{C}H_2$), 31.55 (1C, $ClPhCH_2\underline{C}H_2$), 27.14 (1C,$CH_2\underline{C}H_2CH_2$), 24.34 (2C^{Piperidine}), 22.84 (1C^{Piperidine}), 22.44 (1C, $CH_2\underline{C}H_2CH_2$). Anal. Calcd: C 52.72 %; H 7.92 %; N 12.94 %. Found: C 52.74 %; H 8.32 %; N 12.81 %.

Preparation of 1-[4-(4-chlorophenyl)butyl]-3-[3-(piperidin-1-yl)propyl]guanidine dihydrochloride (**ADS10306**)

4M solution HCl-dioxan (3.26 mL; $1.31{\cdot}10^{-2}$ mol) was added dropwise to a solution of the 1-[4-(4-chlorophenyl)butyl]-1,2-di(*tert*-butoxycarbonyl)-3-[3-(piperidin-1-yl)propyl]guanidine (**25c**) (0.36 g; $6.53{\cdot}10^{-4}$ mol) in 20 mL chloroform. The reaction was stirred overnight at room temperature, then the solvent was removed under vacuum. The crude product was evaporated twice from chloroform and twice from EtOAc. The residue was dissolved in 4 mL water and freeze-dried to yield the pure product as a salt.

1-[4-(4-chlorophenyl)butyl]-3-[3-(piperidin-1-yl)propyl]guanidine dihydrochloride (**ADS10306**): $C_{19}H_{31}ClN_4{\cdot}2HCl{\cdot}H_2O$. M=441.87. Sticky flakes/oil. 80.36 %. ^{1}H NMR (600 MHz, *CD$_3$OD*) δ ppm 7.29-7.28 (m, 2H$^{arom.}$, $C(CHC\underline{H})_2CCl$), 7.23-7.21 (m, 2H$^{arom.}$, $C(C\underline{H}CH)_2CCl$), 3.58-3.55 (m, 2H^{Piperidine}), 3.36-3.34 (m, 2H, $C\underline{H}_2N$), 3.26 (t, 2H, $C\underline{H}_2N$, *J*=6.94Hz), 3.20-3.17 (m, 2H, $C\underline{H}_2N^{piperidine}$), 2.99-2.96 (m, 2H^{Piperidine}), 2.69-2.67 (m, 2H, $ClPhC\underline{H}_2CH_2$, *J*=7.49Hz), 2.10-2.05 (m, 2H, $C\underline{H}_2CH_2N^{piperidine}$), 1.97-1.95 (m, 2H^{Piperidine}), 1.91-1.84 (m, 3H^{Piperidine}), 1.72 (qt, 2H, $CH_2C\underline{H}_2CH_2$), 1.65 (qt, 2H, $CH_2C\underline{H}_2CH_2$), 1.59-1.52 (m, 1H^{Piperidine}). ^{13}C NMR (150.95 MHz, *CD$_3$OD*) δ ppm 157.51 (1C, $\underline{C}$=N), 142.09 (1C$^{quat./arom.}$, $\underline{C}(CHCH)_2CH$), 132.68 (1C$^{quat./arom.}$, $\underline{C}Cl$), 131.14 (2C$^{arom.}$, $C(\underline{C}HCH)_2CCl$), 129.51 (2C$^{arom.}$, $C(CH\underline{C}H)_2CCl$), 55.47 (1C, $\underline{C}H_2N^{piperidine}$), 54.54 (2C^{Piperidine}, $N(\underline{C}H_2CH_2)_2CH_2$), 42.58 (1C, $\underline{C}H_2N$), 39.84 (1C, $\underline{C}H_2N$), 35.66 (1C, $ClPh\underline{C}H_2$), 29.46 (1C, $CH_2\underline{C}H_2CH_2$), 29.43

(1C, $CH_2\underline{C}H_2CH_2$), 24.87 (1C, $\underline{C}H_2CH_2N^{piperidine}$), 24.27 ($2C^{Piperidine}$), 22.71 ($1C^{Piperidine}$). Anal. Calcd: C 51.65 %; H 7.98 %; N 12.68 %. Found: C 51.94 %; H 8.09 %; N 12.38 %.

Preparation of 1-[4-(4-chlorophenyl)butyl]- 3-[4-(piperidin-1-yl)butyl]guanidine dihydrochloride (**ADS10310**)

4M solution HCl-dioxan (2.79 mL; $1.11 \cdot 10^{-2}$ mol) was added dropwise to a solution of the 1-[4-(4-chlorophenyl)butyl]-1,2-di(*tert*-butoxycarbonyl)-3-[4-(piperidin-1-yl)butyl]guanidine (**25d**) (0.315 g; $5.57 \cdot 10^{-4}$ mol) in 15 mL chloroform. The reaction was stirred overnight at room temperature, then the solvent was removed under vacuum. The crude product was evaporated twice from chloroform and twice from EtOAc. The residue was dissolved in 4 mL water and freeze-dried to yield the pure product as a salt.

1-[4-(4-chlorophenyl)butyl]- 3-[4-(piperidin-1-yl)butyl]guanidine dihydrochloride (**ADS10310**): $C_{20}H_{33}ClN_4 \cdot 2HCl \cdot H_2O$. M=455.89. Sticky flakes/oil. 59.84 %. ^{1}H NMR (600 MHz, *CD₃OD*) δ ppm 7.29-7.28 (m, $2H^{arom.}$, $C(CHC\underline{H})_2CCl$), 7.23-7.21 (m, $2H^{arom.}$, $C(C\underline{H}CH)_2CCl$), 3.57-3.55 (m, $2H^{Piperidine}$), 3.30-3.27 (m, 2H, $C\underline{H}_2N$, *J*=6.99Hz), 3.25 (t, 2H, $C\underline{H}_2N$, *J*=6.95Hz), 3.15-3.12 (m, 2H, $C\underline{H}_2N^{piperidine}$), 2.98-2.94 (m, $2H^{Piperidine}$), 2.69-2.67 (m, 2H, $ClPhC\underline{H}_2CH_2$, *J*=7.45Hz), 1.98-1.95 (m, $2H^{Piperidine}$), 1.89-1.83 (m, 5H: $3H^{Piperidine}$, $CH_2C\underline{H}_2CH_2$), 1.74-1.62 (m, 6H, $CH_2C\underline{H}_2CH_2$), 1.58-1.54 (m, $1H^{Piperidine}$). ^{13}C NMR (150.95 MHz, *CD₃OD*) δ ppm 157.51 (1C, $\underline{C}$=N), 142.09 ($1C^{quat./arom.}$, $\underline{C}(CHCH)_2CH$), 132.68 ($1C^{quat./arom.}$, $\underline{C}Cl$), 131.10 ($2C^{arom.}$, $C(\underline{C}HCH)_2CCl$), 129.45 ($2C^{arom.}$, $C(CH\underline{C}H)_2CCl$), 57.69 (1C, $\underline{C}H_2N^{piperidine}$), 54.39 ($2C^{Piperidine}$, $N(\underline{C}H_2CH_2)_2CH_2$), 42.50 (1C, $\underline{C}H_2N$), 41.89 (1C, $\underline{C}H_2N$), 35.65 (1C, $ClPh\underline{C}H_2$), 29.45 (2C, $CH_2\underline{C}H_2CH_2$), 27.12 (1C, $CH_2\underline{C}H_2CH_2$), 24.27 ($2C^{Piperidine}$), 22.77 ($1C^{Piperidine}$), 22.37 (1C, $CH_2\underline{C}H_2CH_2$). Anal. Calcd: C 52.69 %; H 8.18 %; N 12.29 %. Found: C 52.86 %; H 8.41 %; N 11.95 %.

2. NMR spectra

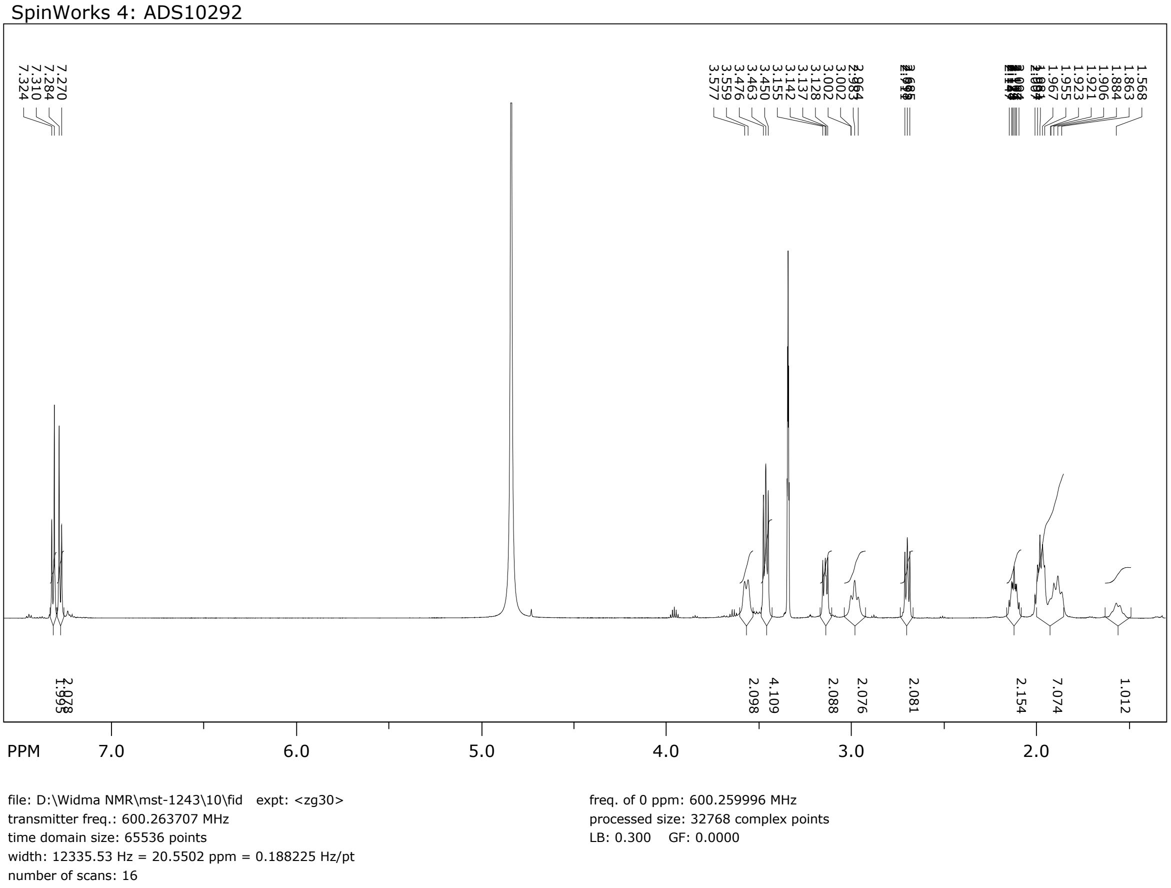

Figure S1.¹H NMR spectra of compound ADS10292.

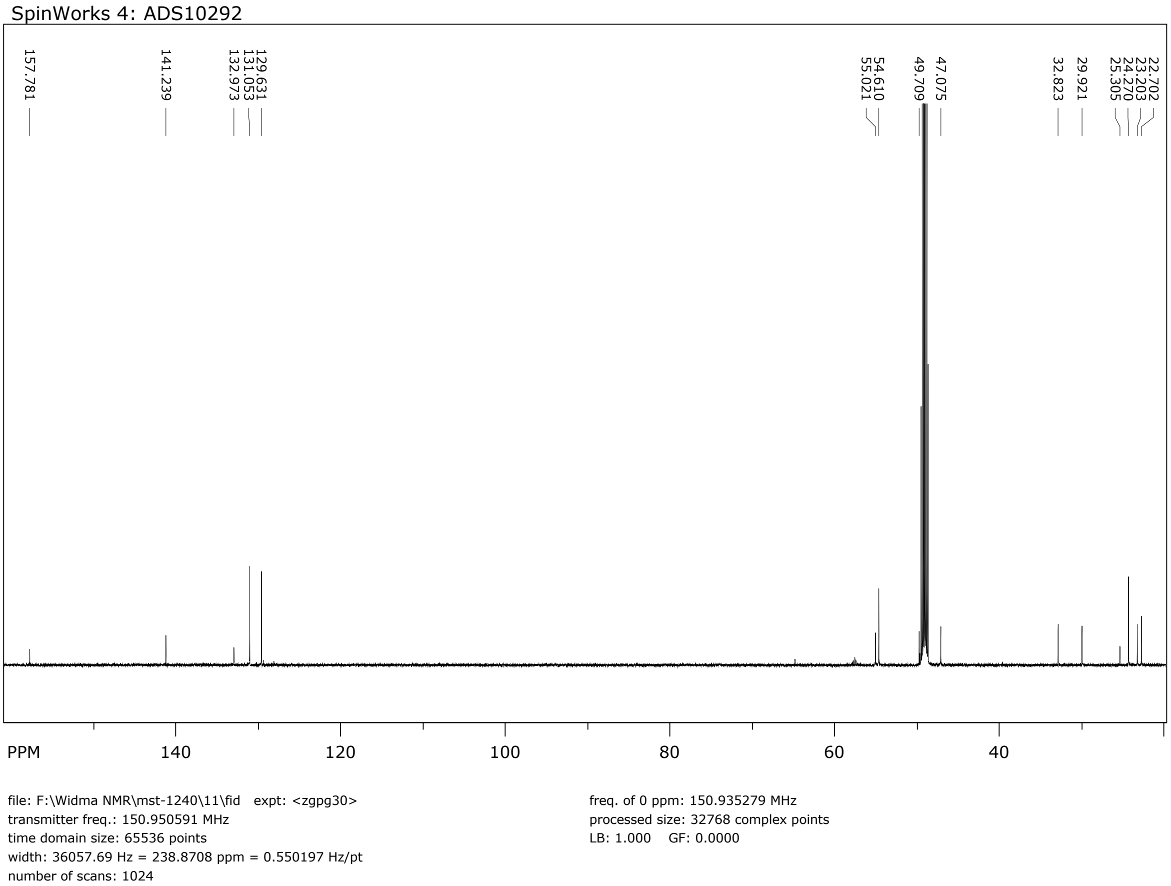

Figure S2. ^{13}C NMR spectra of compound ADS10292.

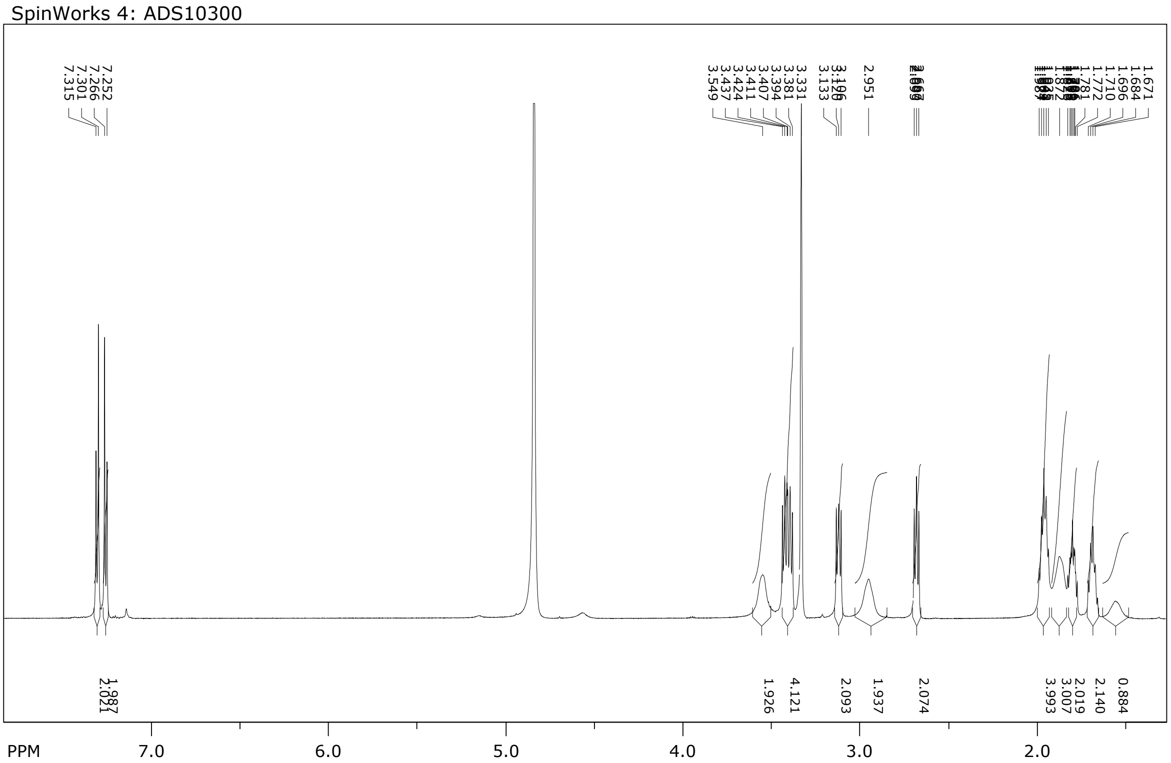

file: D:\Widma NMR\mst-1241\10\fid expt: <zg30>
transmitter freq.: 600.263707 MHz
time domain size: 65536 points
width: 12335.53 Hz = 20.5502 ppm = 0.188225 Hz/pt
number of scans: 16

freq. of 0 ppm: 600.260003 MHz
processed size: 32768 complex points
LB: 0.300 GF: 0.0000

Figure S3.^{1}H NMR spectra of compound ADS10300.

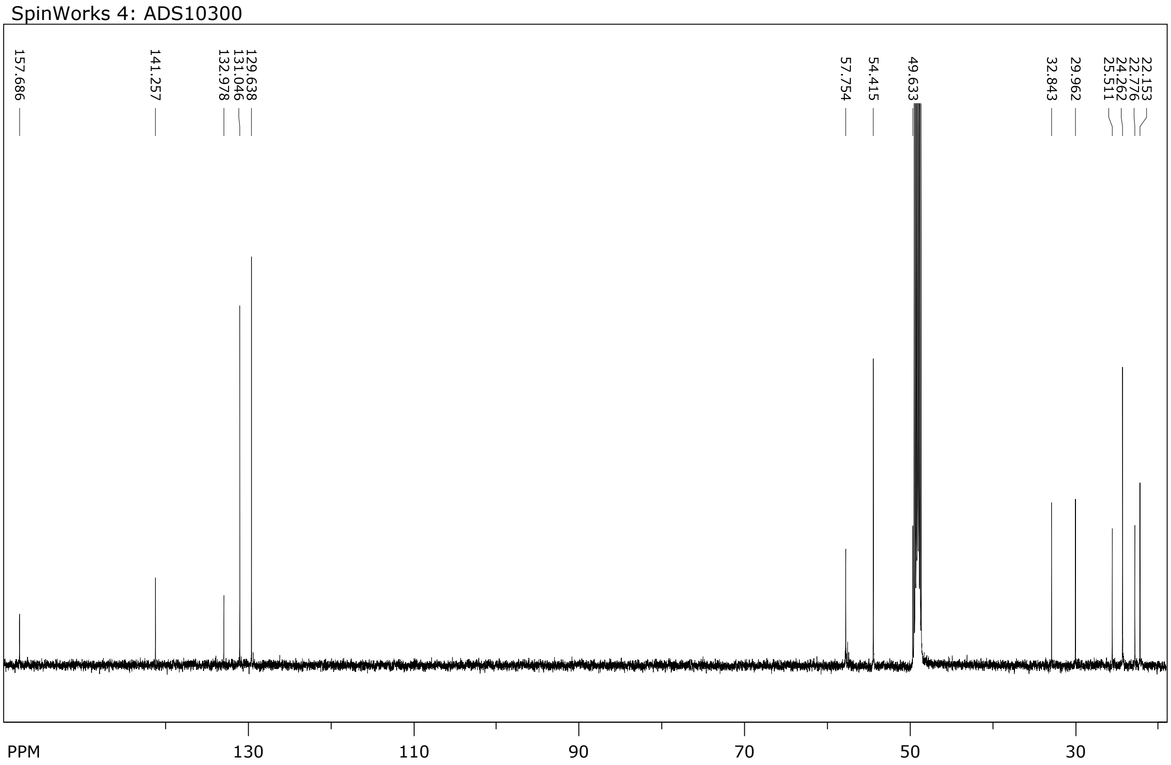

file: F:\Widma NMR\mst-1241\11\fid expt: <zgpg30>
transmitter freq.: 150.950591 MHz
time domain size: 65536 points
width: 36057.69 Hz = 238.8708 ppm = 0.550197 Hz/pt
number of scans: 1024

freq. of 0 ppm: 150.935278 MHz
processed size: 32768 complex points
LB: 1.000 GF: 0.0000

Figure S4. ^{13}C NMR spectra of compound ADS10300.

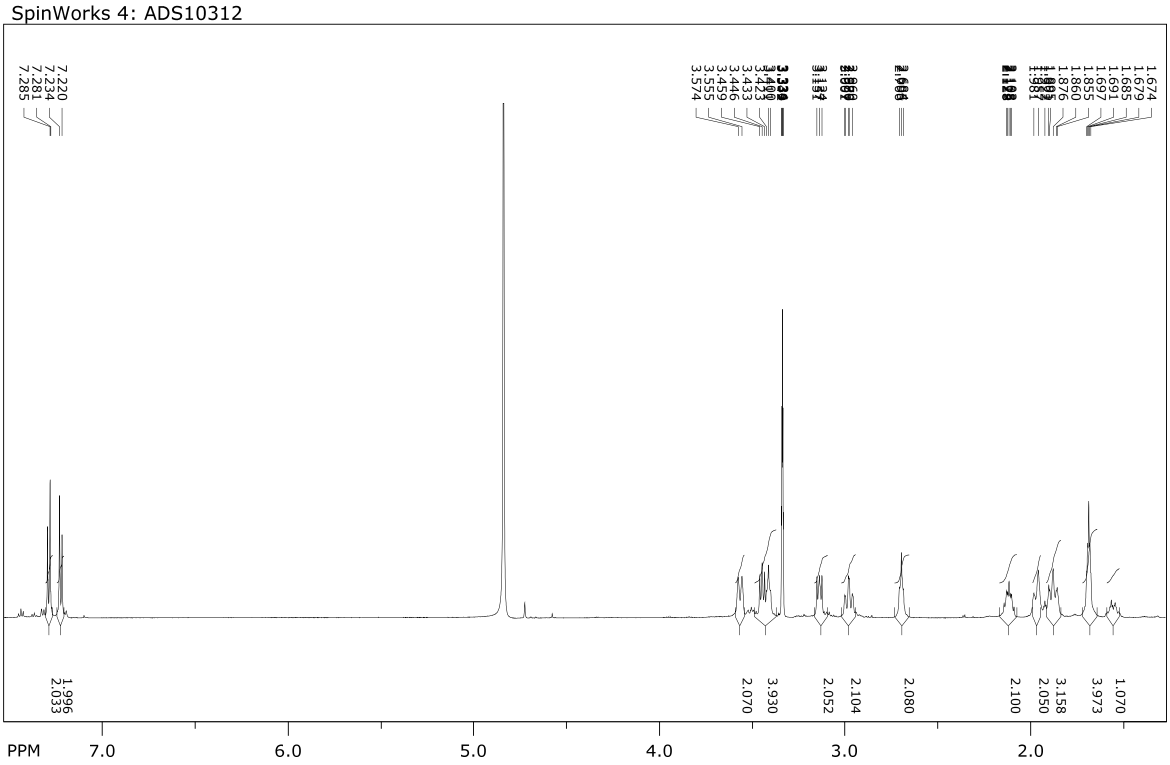

file: F:\Widma NMR\mst-1242\10\fid expt: <zg30>
transmitter freq.: 600.263707 MHz
time domain size: 65536 points
width: 12335.53 Hz = 20.5502 ppm = 0.188225 Hz/pt
number of scans: 16

freq. of 0 ppm: 600.260000 MHz
processed size: 32768 complex points
LB: 0.300 GF: 0.0000

Figure S5.¹H NMR spectra of compound ADS10312.

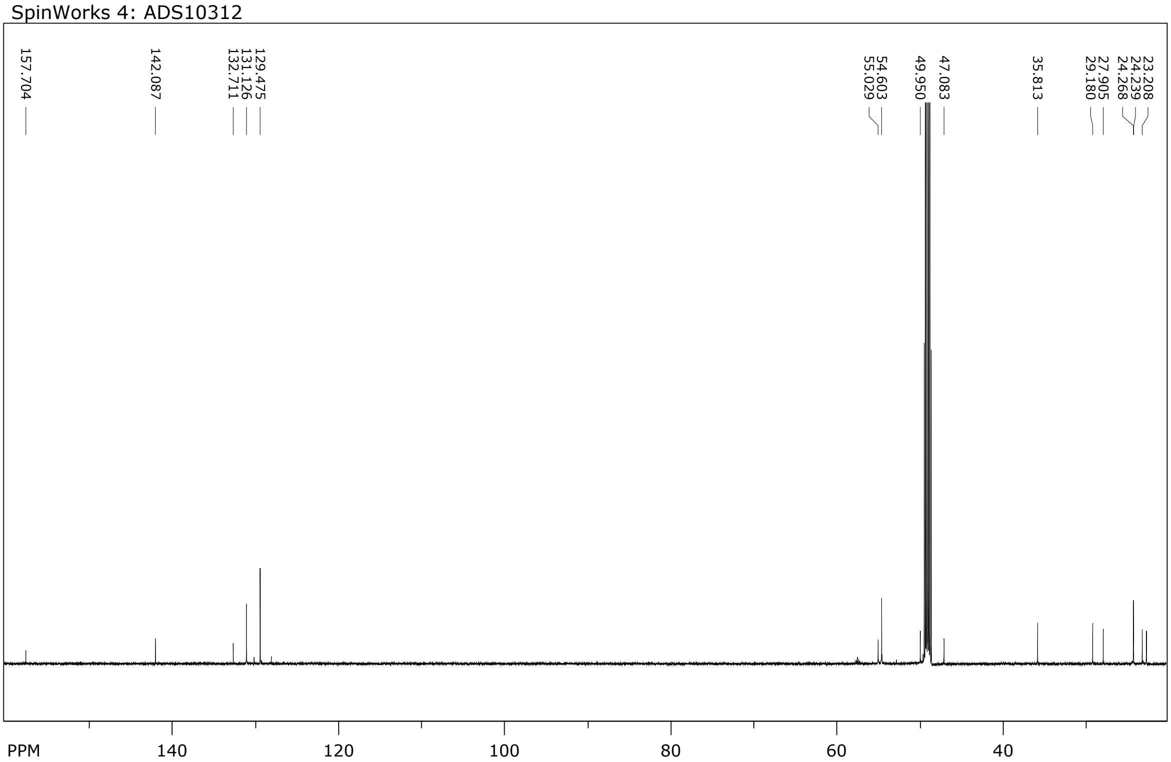

file: F:\Widma NMR\mst-1242\11\fid expt: <zgpg30>
transmitter freq.: 150.950591 MHz
time domain size: 65536 points
width: 36057.69 Hz = 238.8708 ppm = 0.550197 Hz/pt
number of scans: 1024

freq. of 0 ppm: 150.935280 MHz
processed size: 32768 complex points
LB: 1.000 GF: 0.0000

Figure S6. ^{13}C NMR spectra of compound ADS10312.

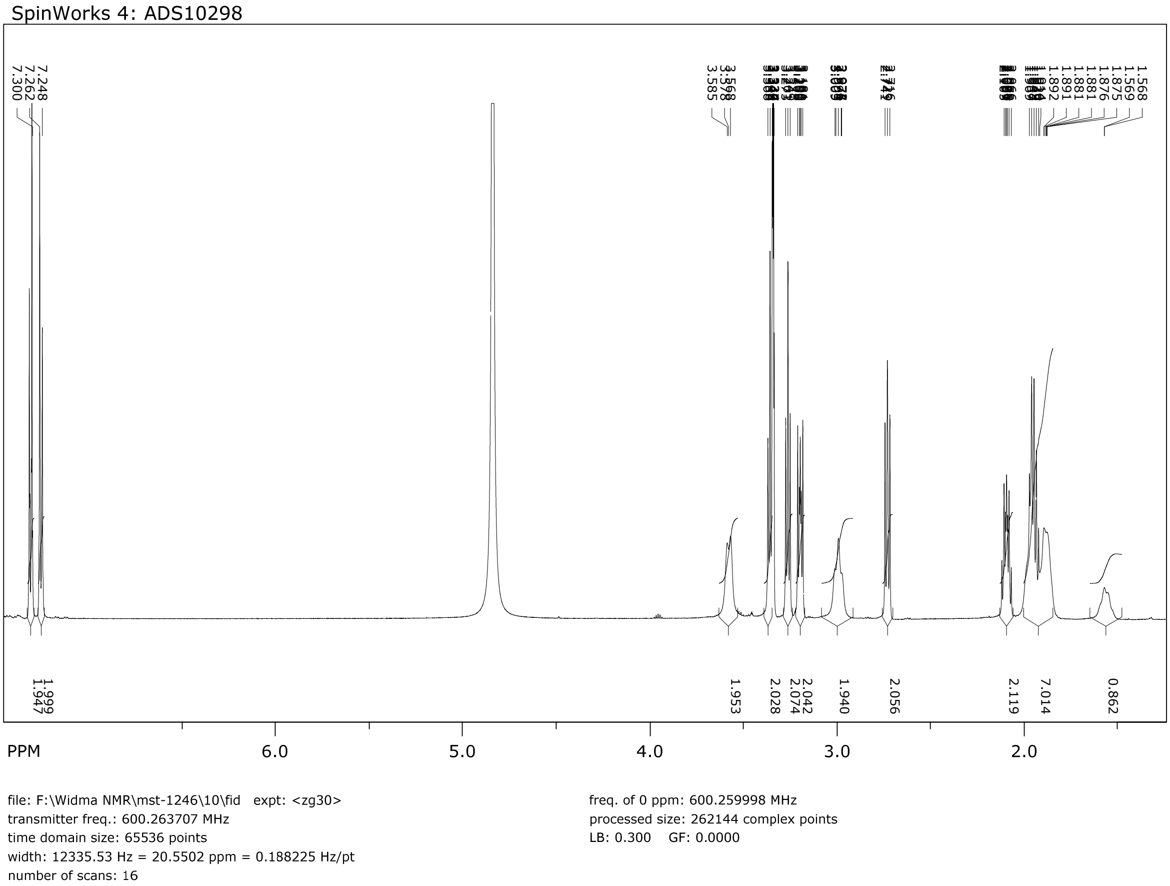

Figure S7.^{1}H NMR spectra of compound ADS10298.

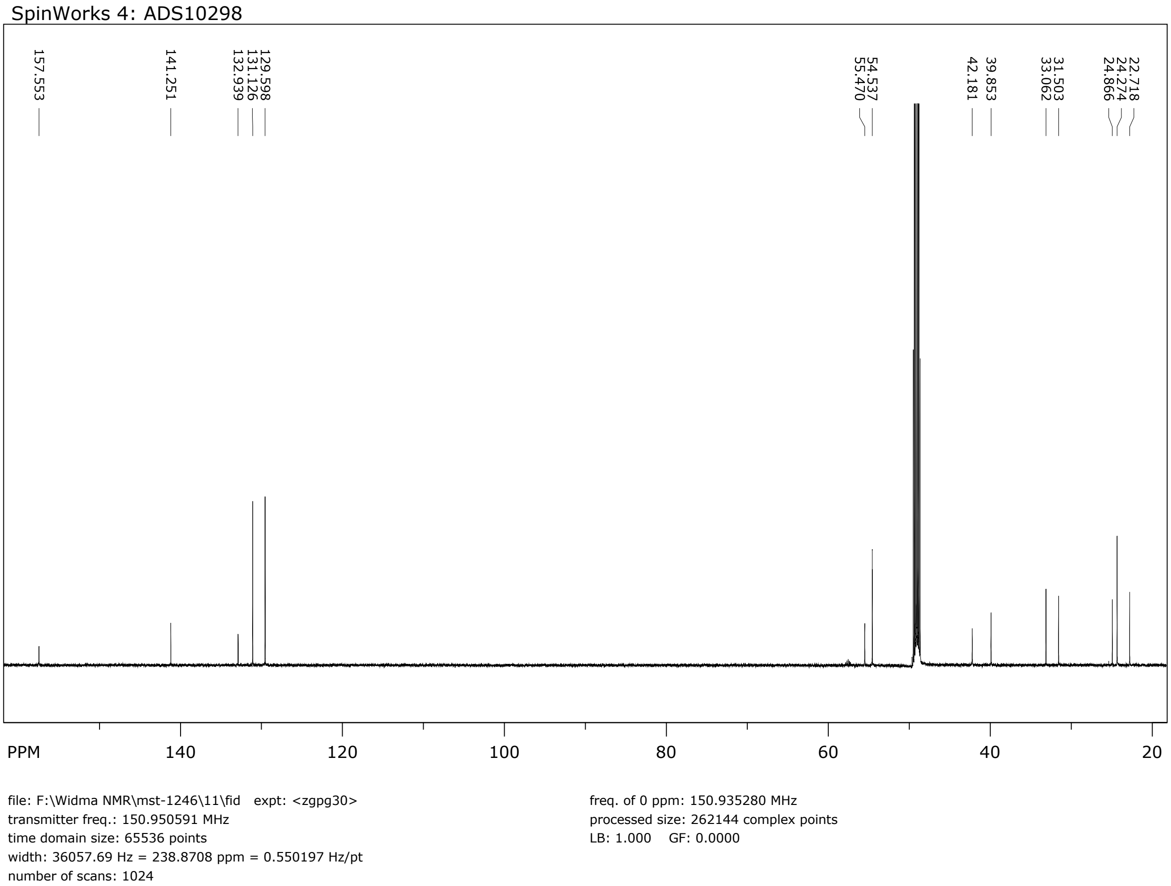

Figure S8. ^{13}C NMR spectra of compound ADS10298.

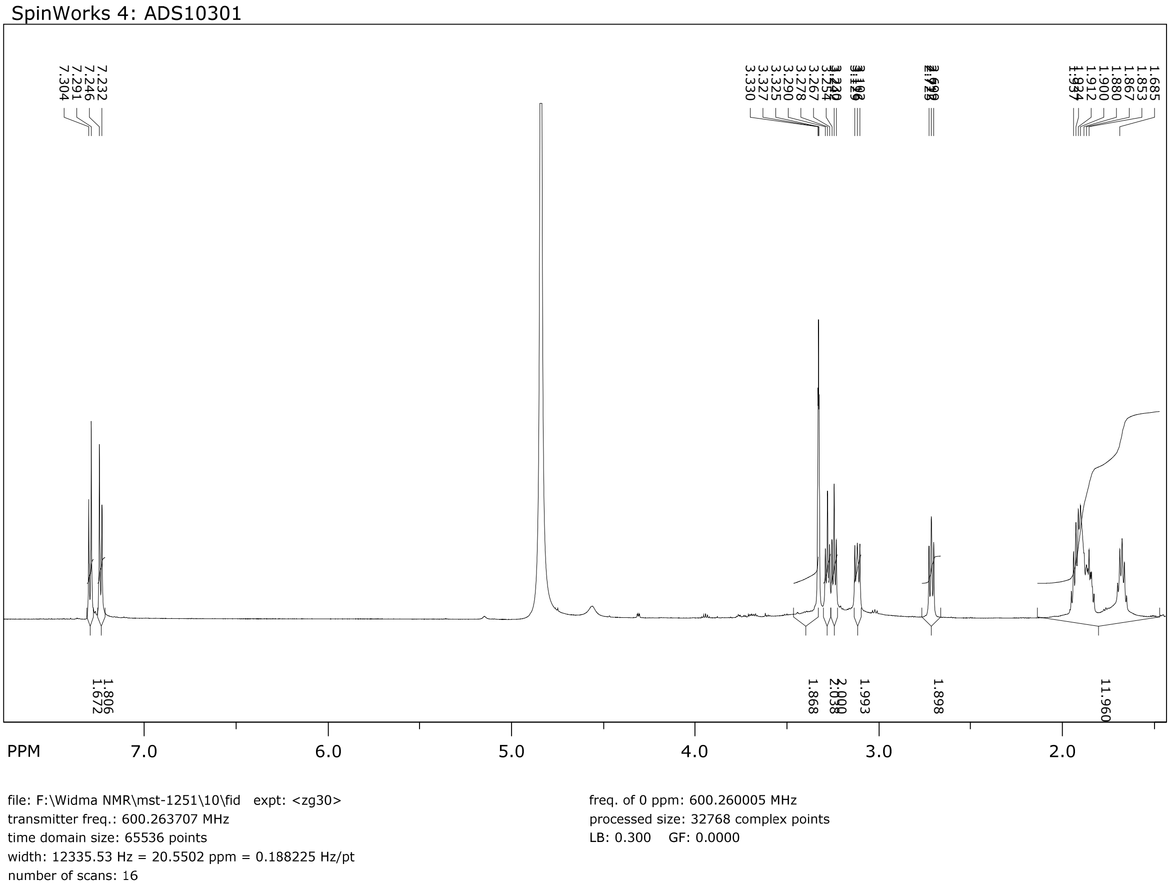

Figure S9.^{1}H NMR spectra of compound 10301

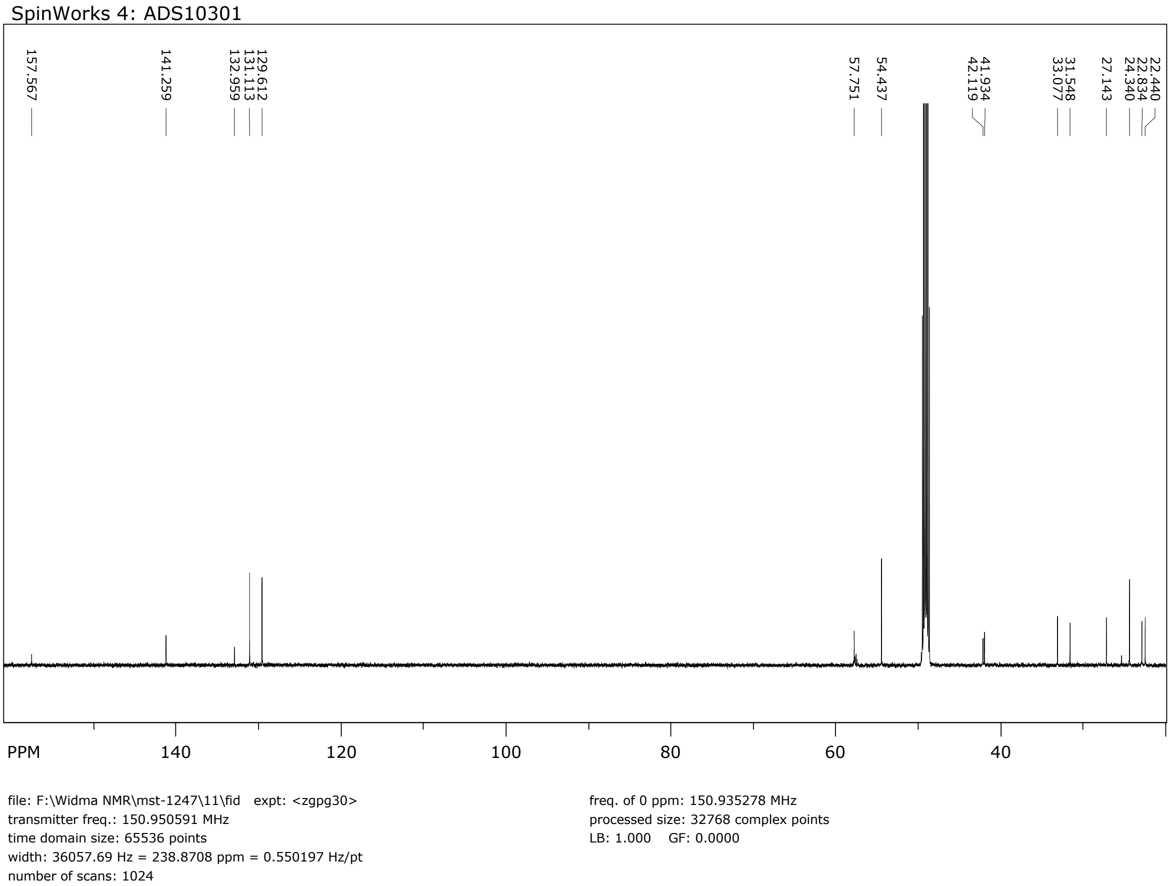

Figure S10. ^{13}C NMR spectra of compound ADS10301.

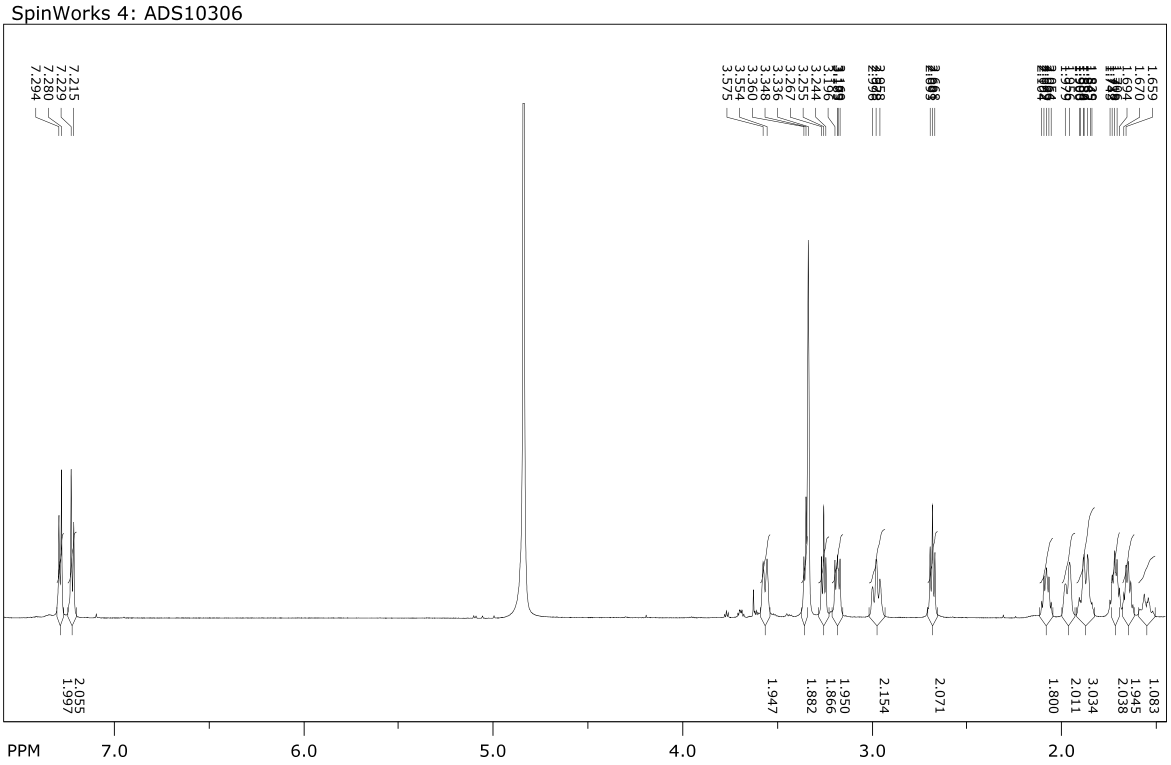

file: F:\Widma NMR\mst-1248\10\fid   expt: <zg30>
transmitter freq.: 600.263707 MHz
time domain size: 65536 points
width: 12335.53 Hz = 20.5502 ppm = 0.188225 Hz/pt
number of scans: 16

freq. of 0 ppm: 600.260000 MHz
processed size: 32768 complex points
LB: 0.300   GF: 0.0000

Figure S11.^{1}H NMR spectra of compound ADS10306.

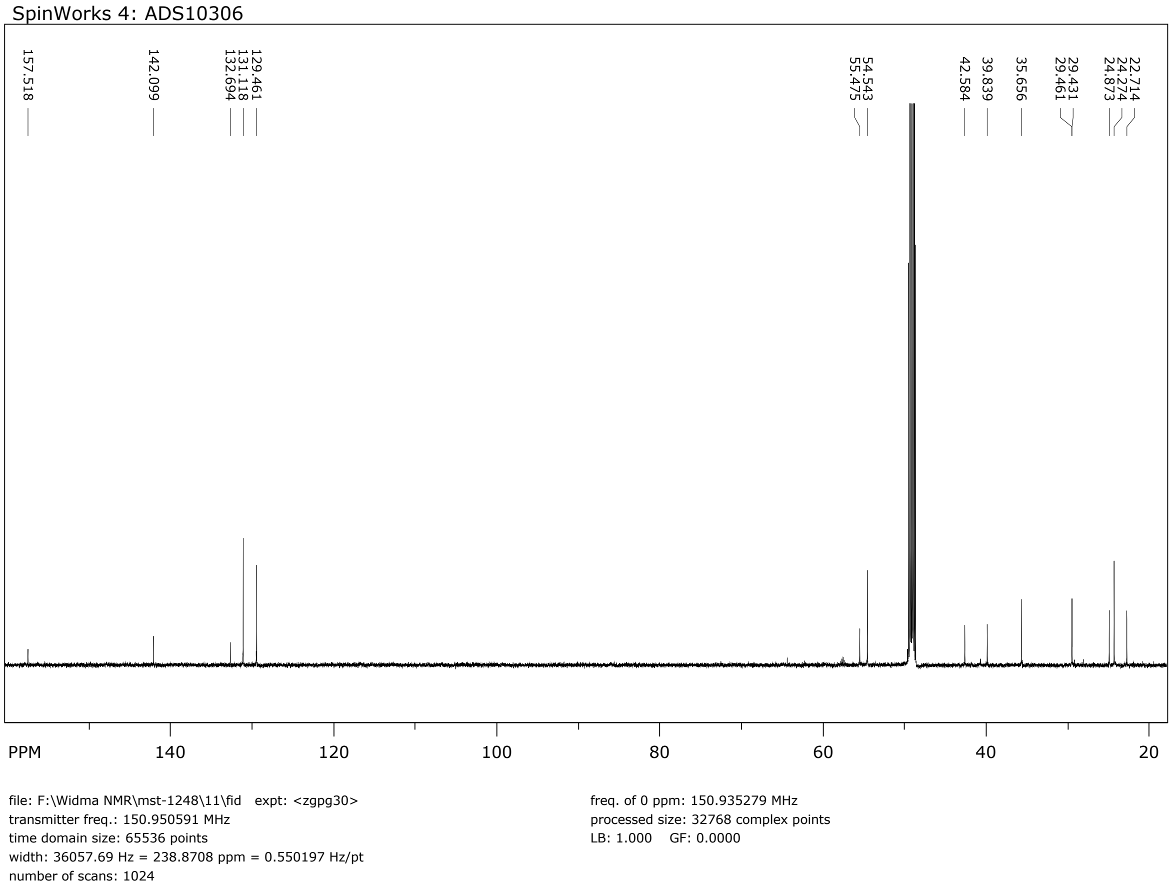

Figure S12. ^{13}C NMR spectra of compound ADS10306.

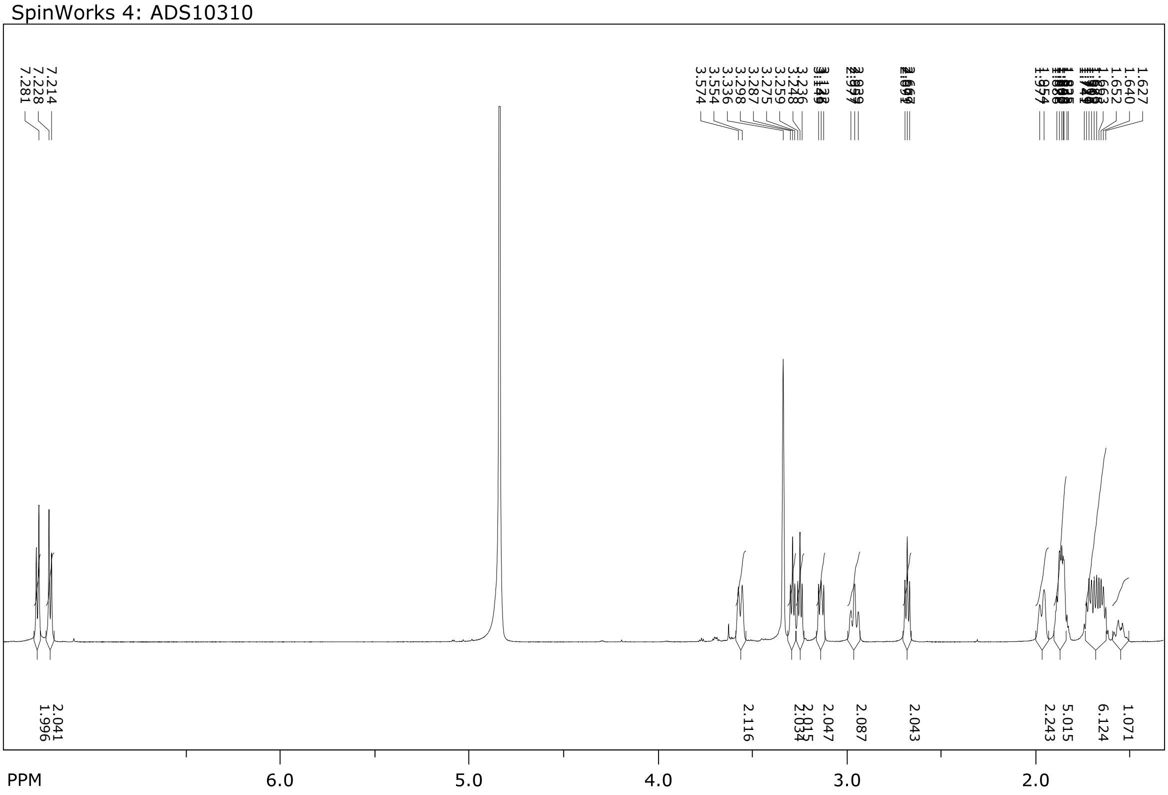

file: F:\Widma NMR\mst-1249\10\fid expt: <zg30>
transmitter freq.: 600.263707 MHz
time domain size: 65536 points
width: 12335.53 Hz = 20.5502 ppm = 0.188225 Hz/pt
number of scans: 16

freq. of 0 ppm: 600.259999 MHz
processed size: 32768 complex points
LB: 0.300 GF: 0.0000

Figure S13.^{1}H NMR spectra of compound ADS10310.

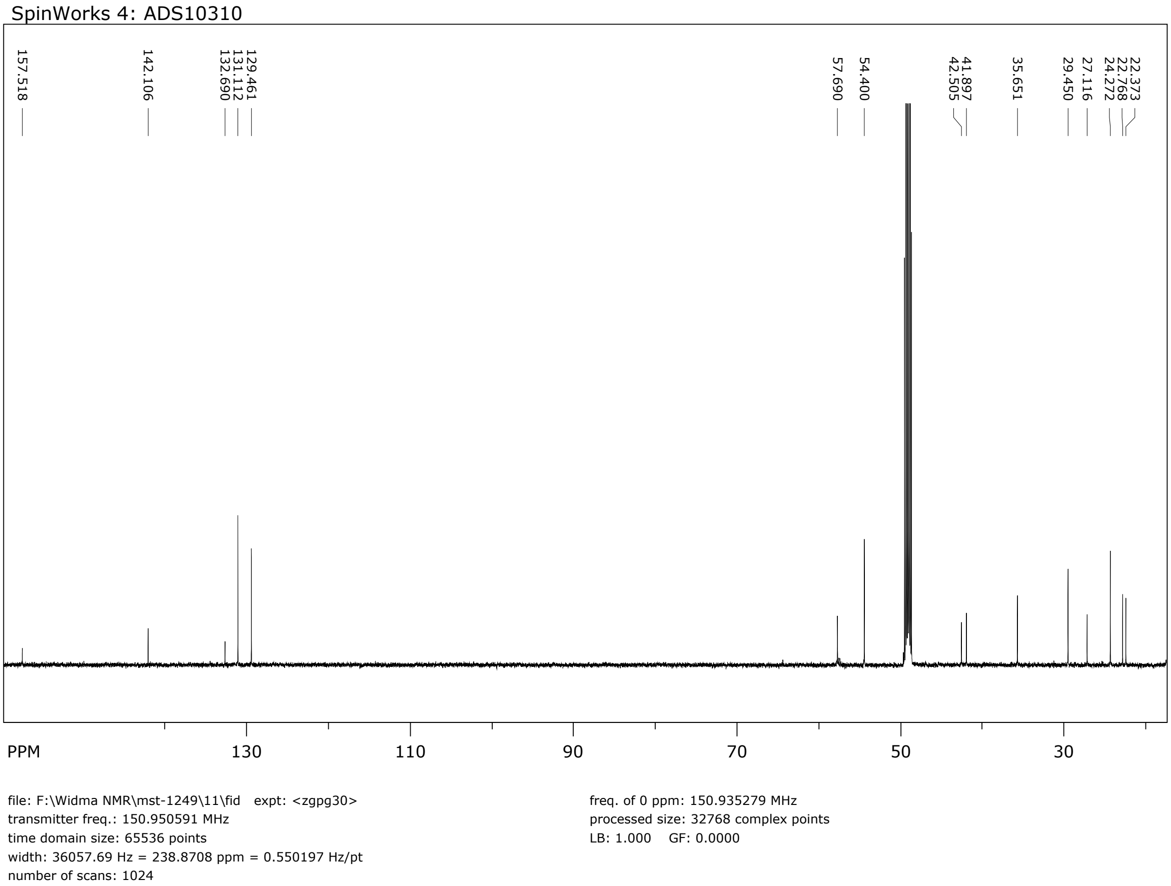

Figure S14. ^{13}C NMR spectra of compound ADS10310.

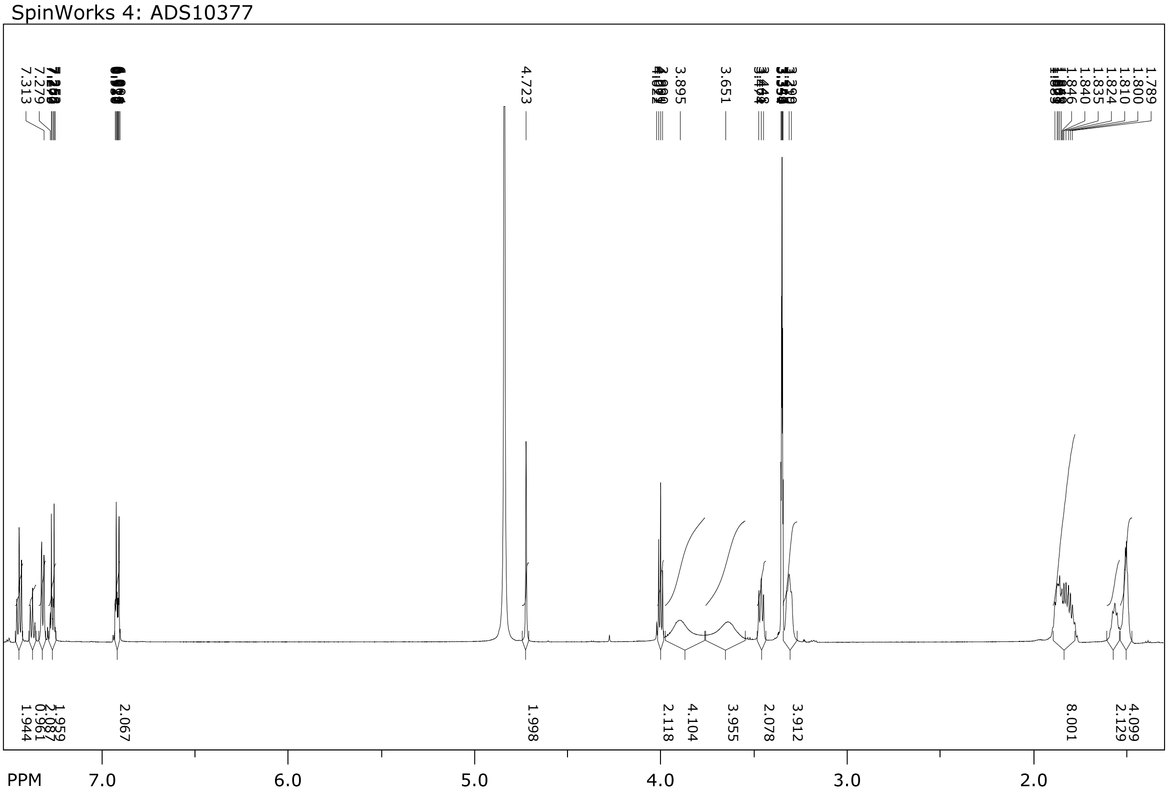

file: F:\Widma NMR\mst-1237\10\fid expt: <zg30>
transmitter freq.: 600.263707 MHz
time domain size: 65536 points
width: 12335.53 Hz = 20.5502 ppm = 0.188225 Hz/pt
number of scans: 16

freq. of 0 ppm: 600.259992 MHz
processed size: 32768 complex points
LB: 0.300 GF: 0.0000

Figure S15.^{1}H NMR spectra of compound ADS10377.

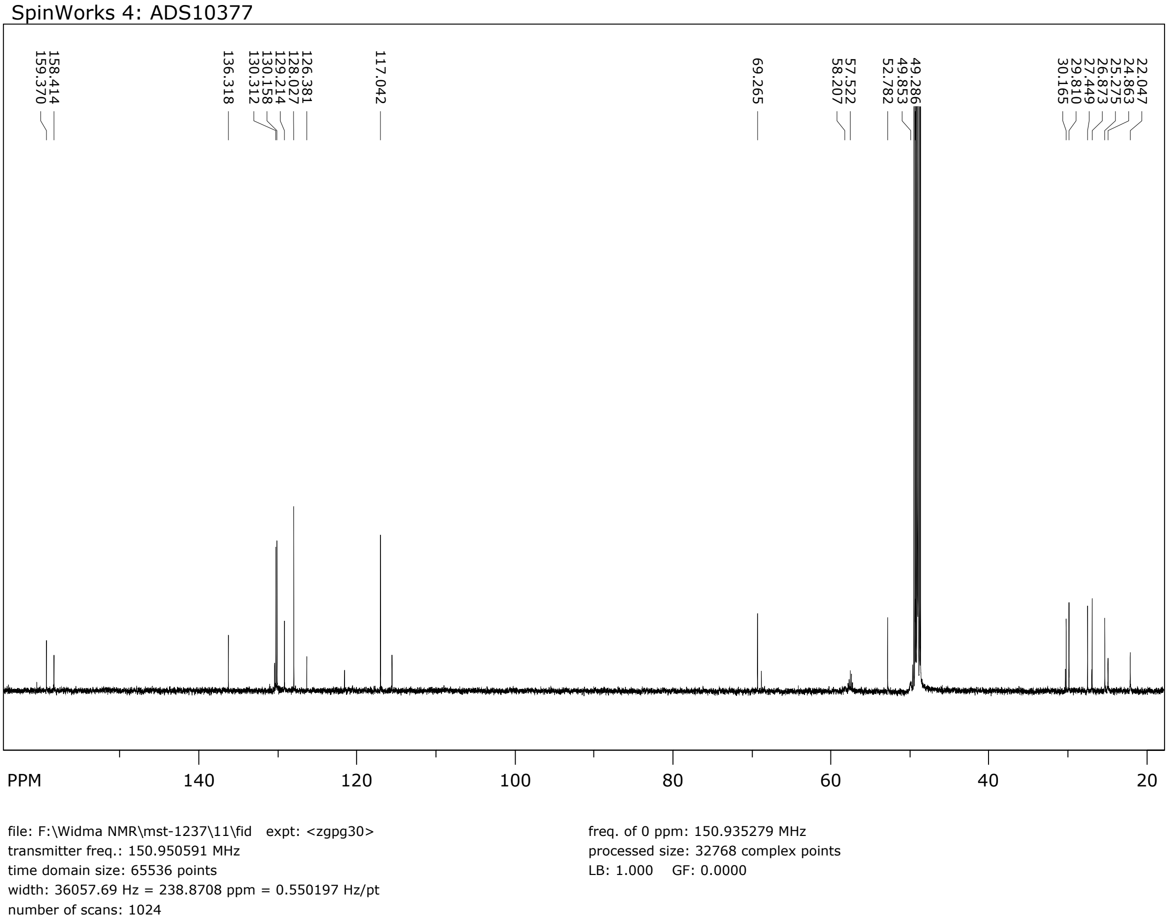

Figure S16. ^{13}C NMR spectra of compound ADS10377.

SpinWorks 4: ADS10376

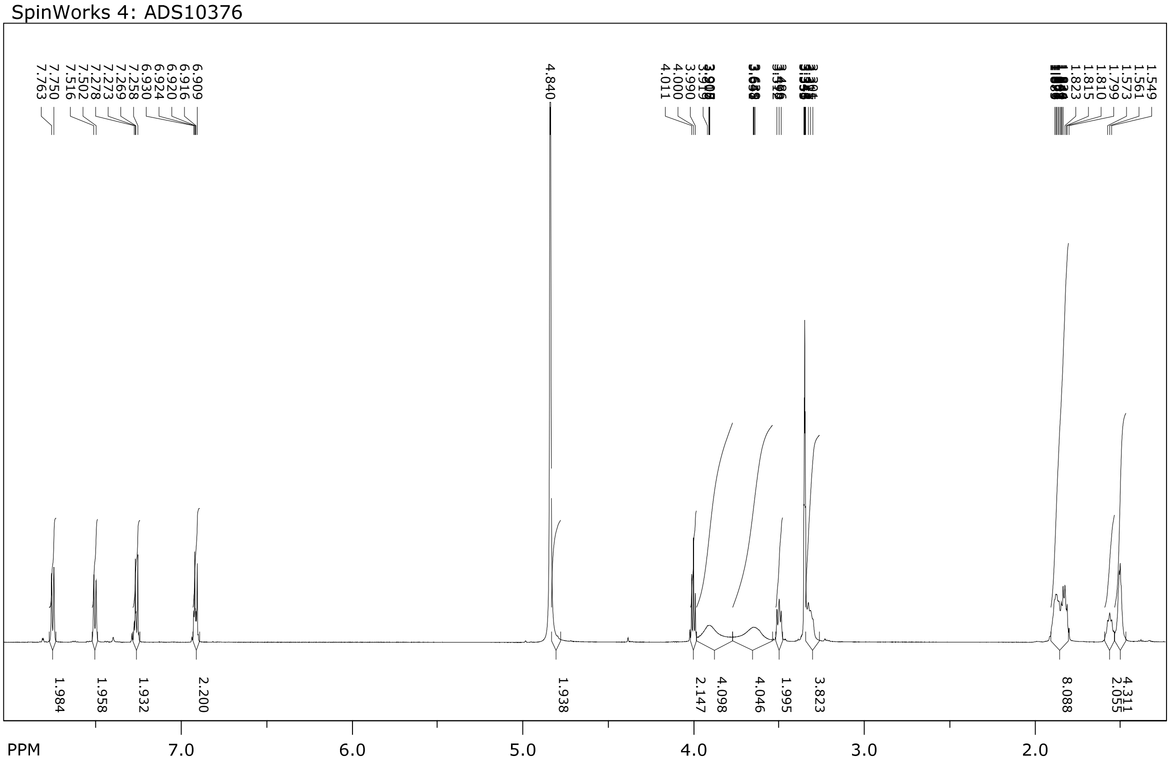

file: F:\Widma NMR\mst-1236\10\fid expt: <zg30>
transmitter freq.: 600.263707 MHz
time domain size: 65536 points
width: 12335.53 Hz = 20.5502 ppm = 0.188225 Hz/pt
number of scans: 16

freq. of 0 ppm: 600.259992 MHz
processed size: 32768 complex points
LB: 0.300 GF: 0.0000

Figure S17.^{1}H NMR spectra of compound ADS10376.

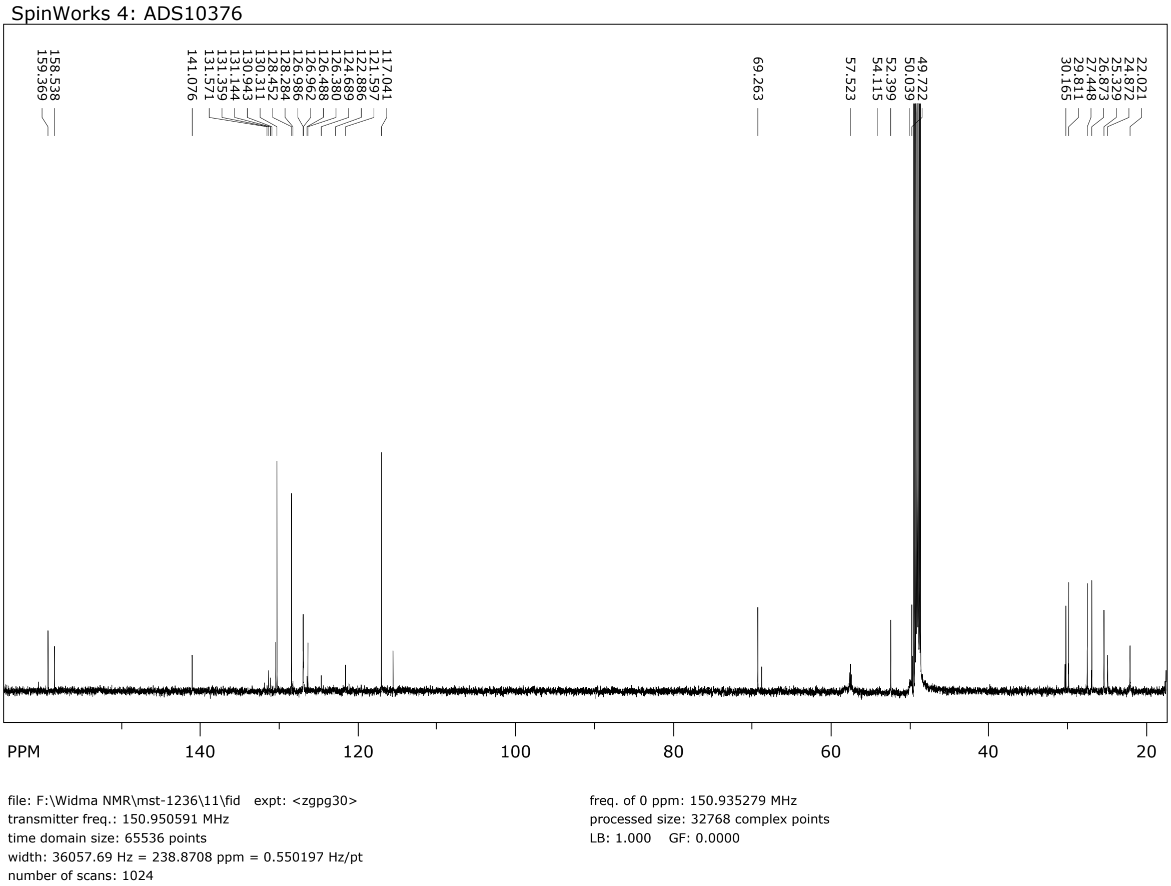

Figure S18. ^{13}C **NMR spectra of compound ADS10376.**

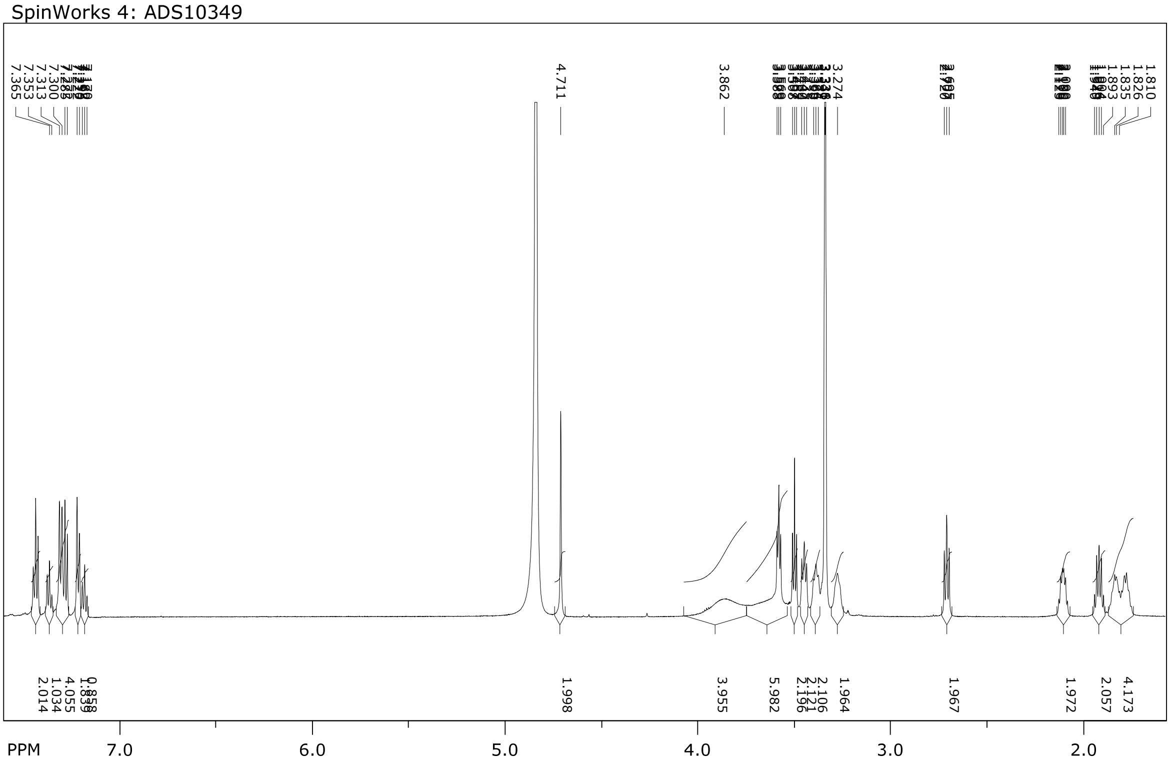

file: F:\Widma NMR\mst-1239\10\fid expt: <zg30>
transmitter freq.: 600.263707 MHz
time domain size: 65536 points
width: 12335.53 Hz = 20.5502 ppm = 0.188225 Hz/pt
number of scans: 16

freq. of 0 ppm: 600.259998 MHz
processed size: 32768 complex points
LB: 0.300 GF: 0.0000

Figure S19.^{1}H NMR spectra of compound ADS10349.

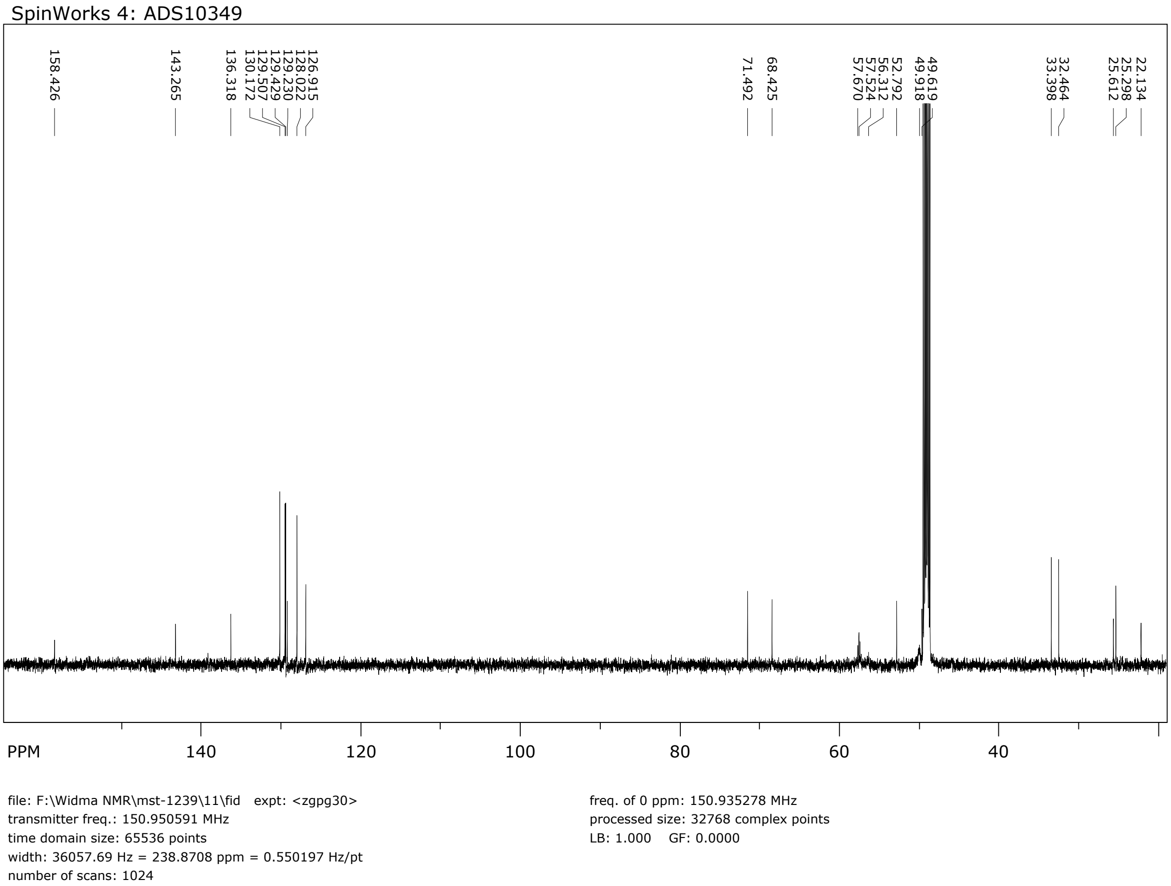

Figure S20. ^{13}C NMR spectra of compound ADS10349.

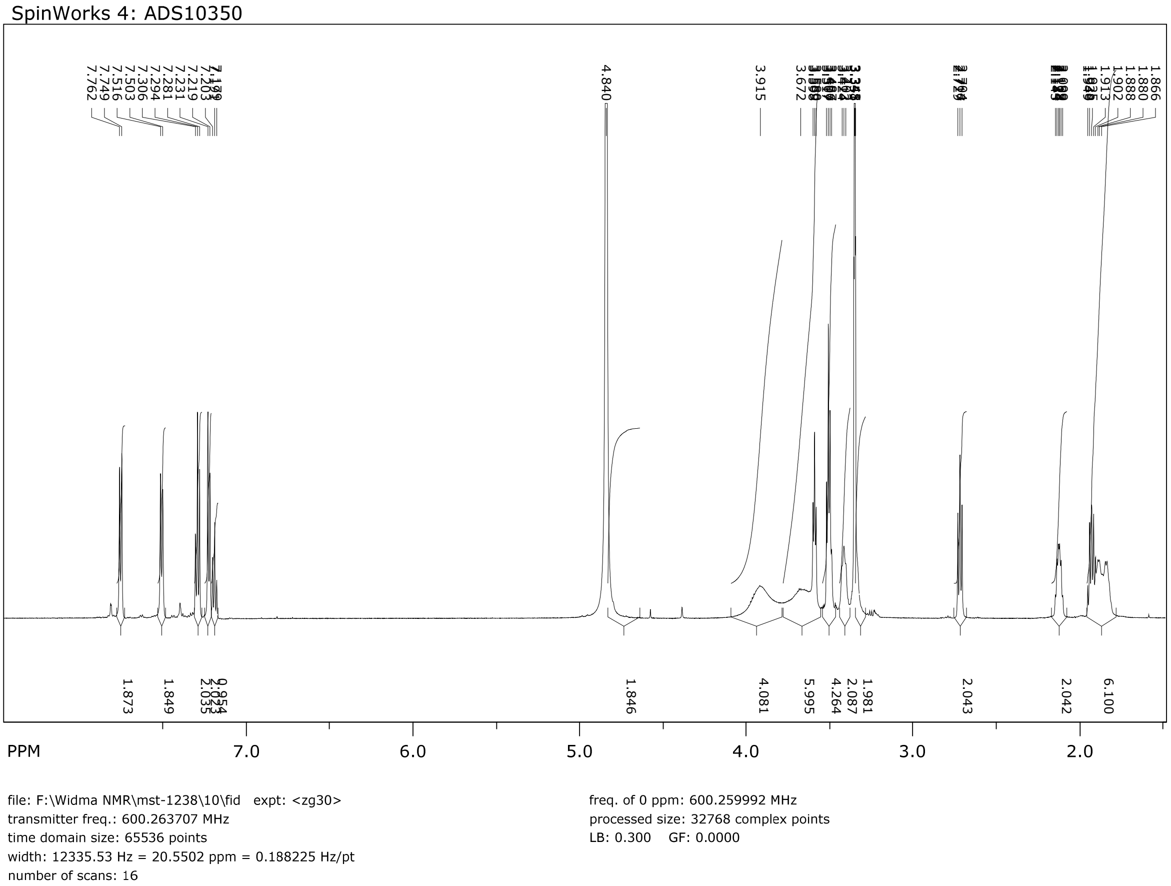

Figure S21.^{1}H NMR spectra of compound ADS10350.

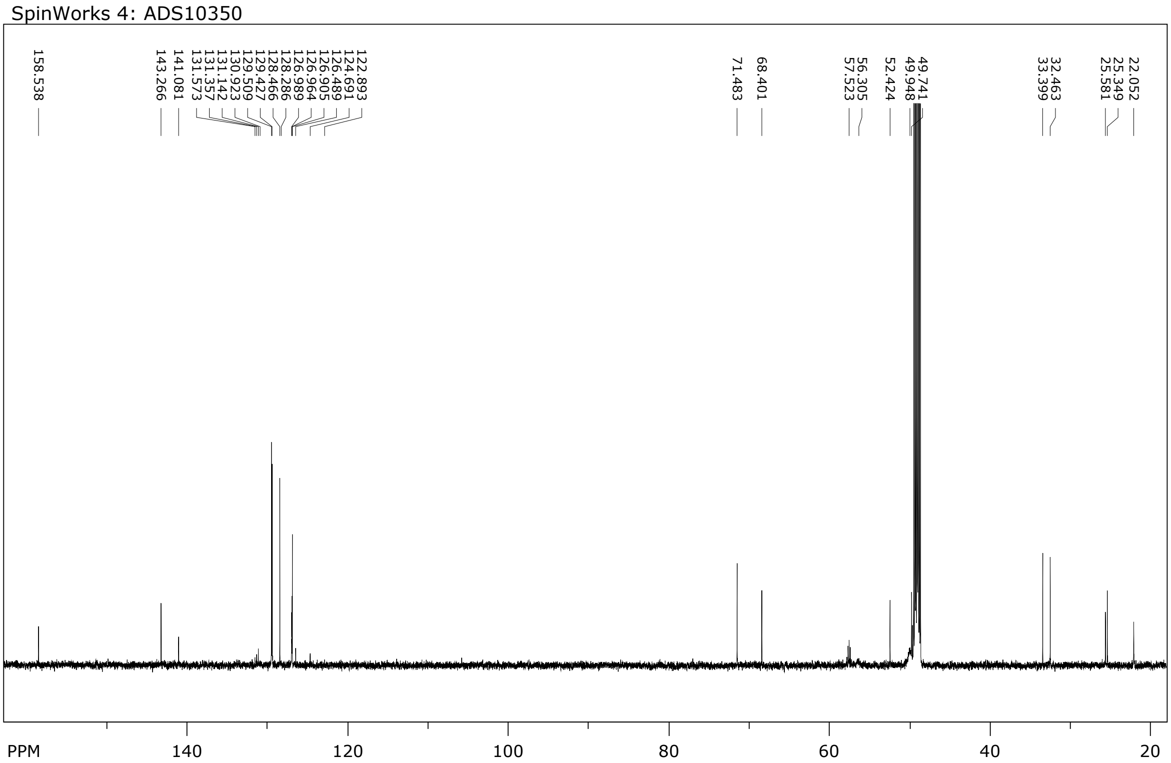

file: F:\Widma NMR\mst-1238\11\fid expt: <zgpg30>
transmitter freq.: 150.950591 MHz
time domain size: 65536 points
width: 36057.69 Hz = 238.8708 ppm = 0.550197 Hz/pt
number of scans: 1024

freq. of 0 ppm: 150.935279 MHz
processed size: 32768 complex points
LB: 1.000 GF: 0.0000

Figure S22. ^{13}C NMR spectra of compound ADS10350.

SpinWorks 4: ADS10278

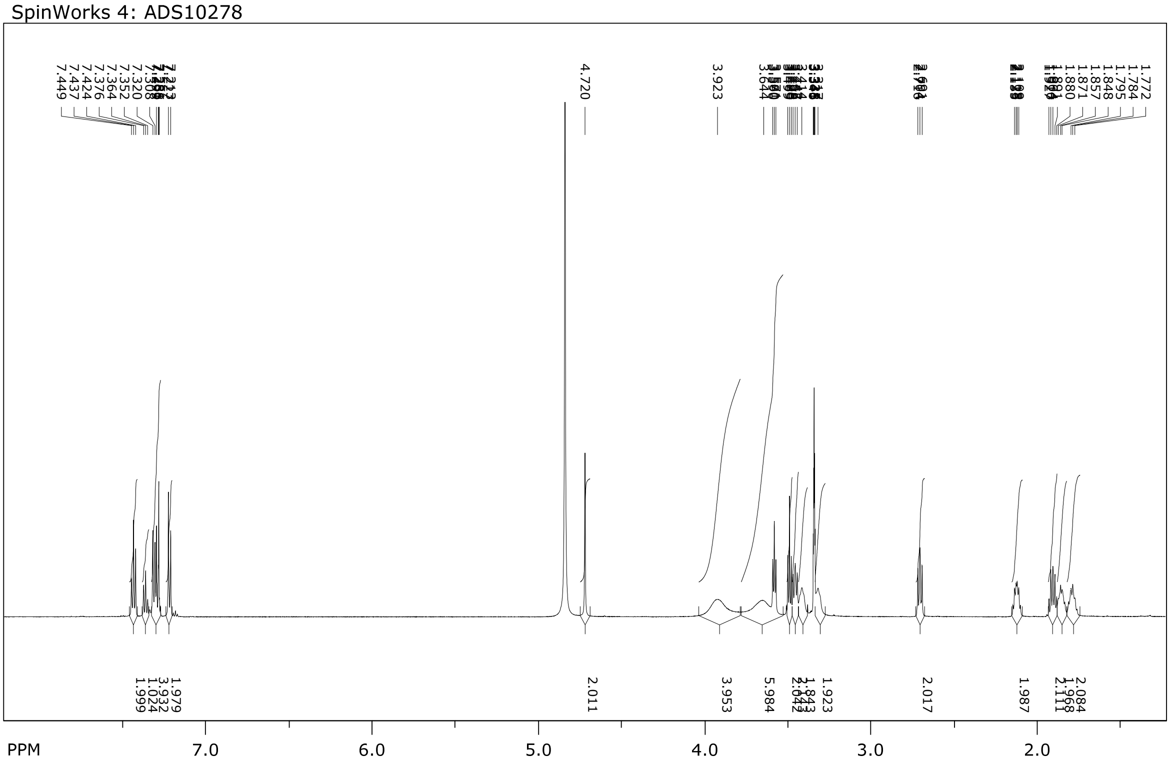

file: F:\Widma NMR\mst-0978\1\fid expt: <zg30>
transmitter freq.: 600.263707 MHz
time domain size: 65536 points
width: 12335.53 Hz = 20.5502 ppm = 0.188225 Hz/pt
number of scans: 16

freq. of 0 ppm: 600.259997 MHz
processed size: 32768 complex points
LB: 0.000 GF: 0.0000

Figure S23.^{1}H NMR spectra of compound ADS10278.

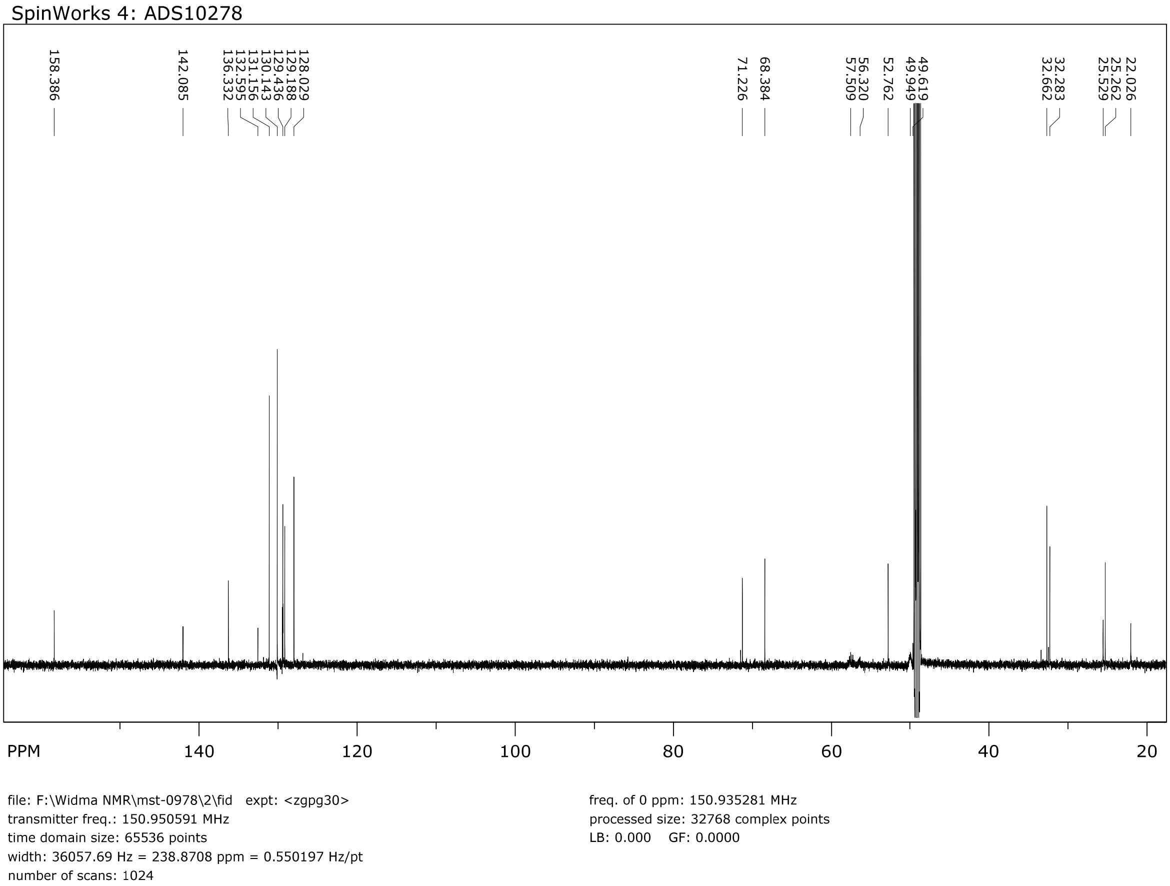

Figure S24. ^{13}C NMR spectra of compound ADS10278.

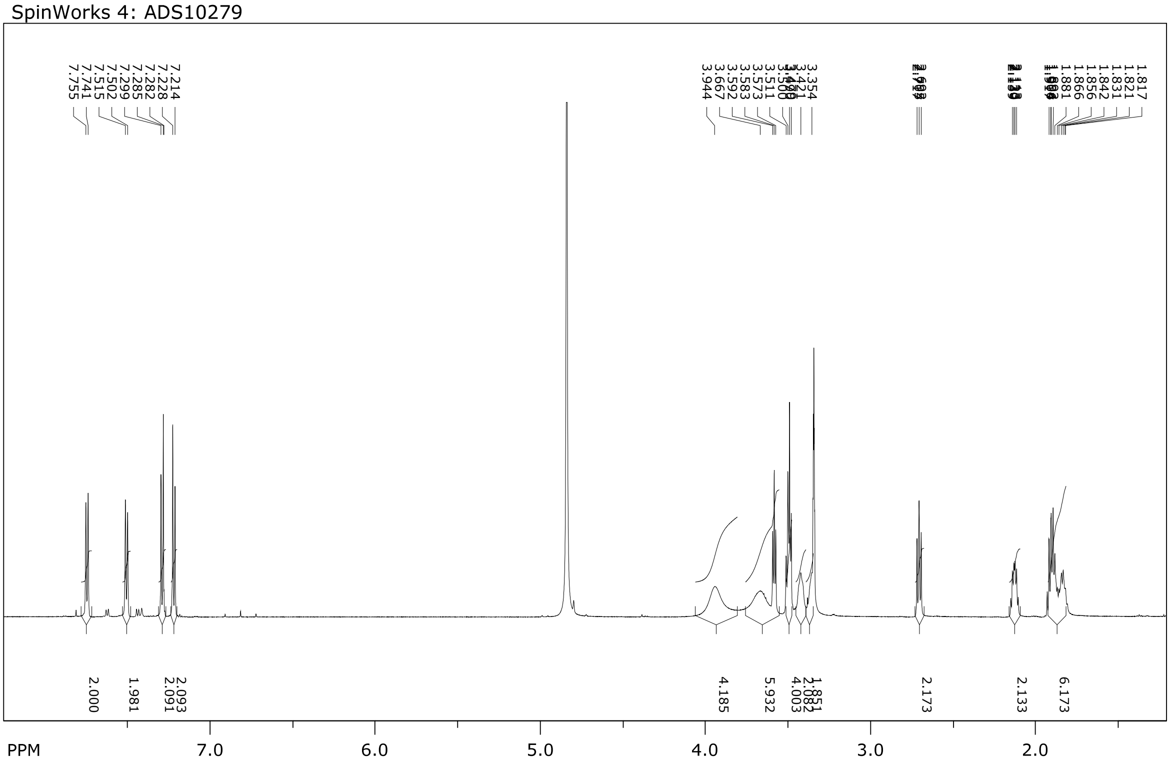

file: F:\Widma NMR\mst-0977\1\fid expt: <zg30>
transmitter freq.: 600.263707 MHz
time domain size: 65536 points
width: 12335.53 Hz = 20.5502 ppm = 0.188225 Hz/pt
number of scans: 16

freq. of 0 ppm: 600.259996 MHz
processed size: 32768 complex points
LB: 0.000 GF: 0.0000

Figure S25.^{1}H NMR spectra of compound ADS10279.

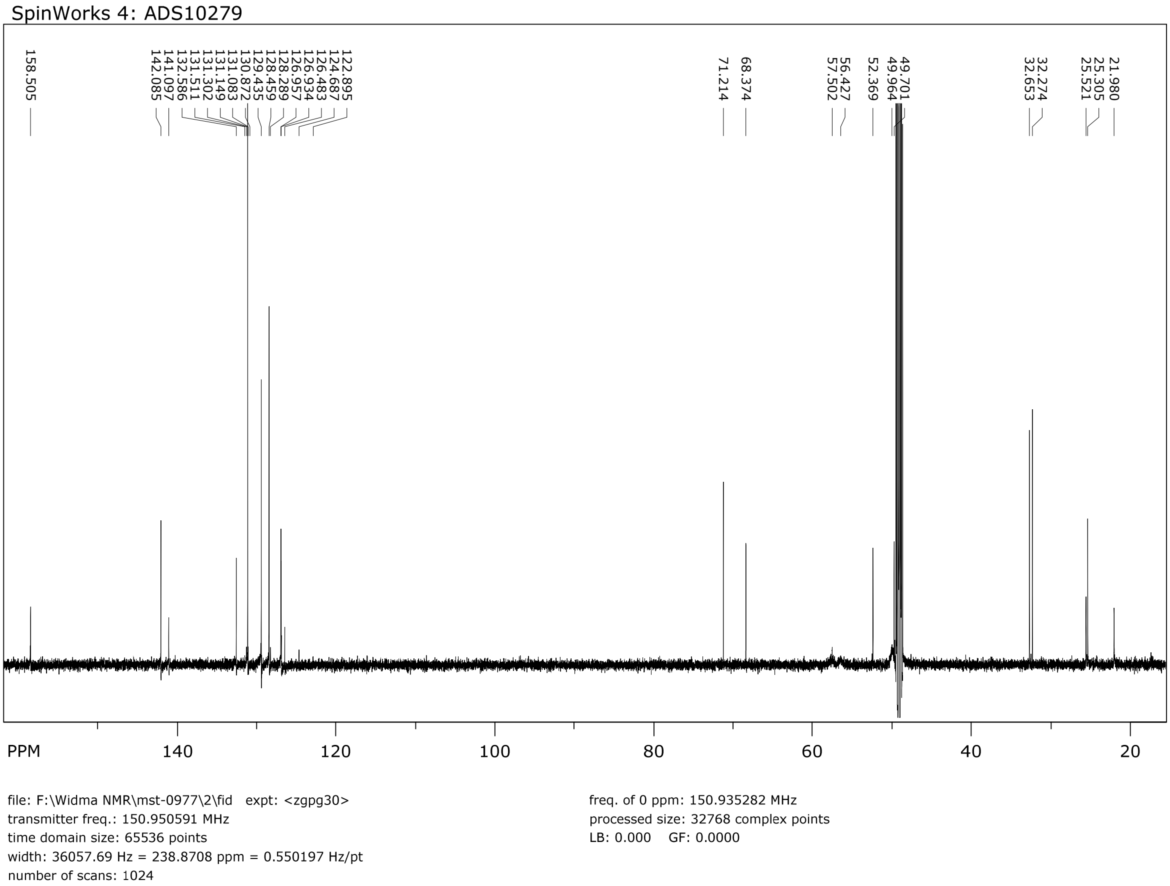

Figure S26. ^{13}**C NMR spectra of compound ADS10279.**

3. Pharmacological assay results.

3.1. *Ex vivo* assay for histamine H_3R receptor antagonists on guinea pig ileum.

Male guinea pigs, weighing 300-400 g were euthanized by a blow to the neck. Following this, a 20-30 cm length of the distal ileum, apart from the terminal 5 cm was rapidly removed and placed in phosphate buffer at room temperature (pH 7.4) containing (mM) NaCl (136.9); KCl (2.6); KH_2PO_4 (1.47); Na_2HPO_4 (9.58) and indomethacin (Sigma-Aldrich, St. Louis, MO, USA) ($1 \cdot 10^{-6}$ mol/L). The intraluminal content was rinsed and the isolated intestine was cut into 1.5-2 cm segments. The preparations were mounted between two platinum electrodes isotonically in a 20 mL organ bath filled with Krebs buffer: composition (mM) NaCl (118); KCl (5.6); $MgSO_4$ (1.18); $CaCl_2$ (2.5); $NaH_2PO_4 \cdot H_2O$ (1.28); $NaHCO_3$ (25); glucose (5.55) and indomethacin ($3 \cdot 10^{-7}$ mol/L). The solution was continuously bubbled with a 95 % O_2 : 5 % CO_2 mixture and maintained at 37 °C under a constant load of 1.0 g (Hugo Sachs Hebel-Messvorsatz (Tl-2)/HF-modem; Hugo Sachs Elektronik, Hugstetten, Germany) connected to a pen recorder (Kipp & Zonen BD41, Delft, Holland). During an equilibration period of 60 min, the Krebs buffer was changed every 10 min. The preparations were then continuously stimulated at 15-20 V at a frequency of 0.1 Hz for a duration of 0.5 ms, with rectangular-wave electrical pulses (Grass Stimulator S-88; Grass Instruments Co., Quincy, Massachusetts, USA). After about 30min, the twitches were recurrent. Five minutes before (*R*)-(−)-α-methylhistamine (RAMH) (Toronto Research Chemicals Inc., North York, Canada), administration, pyrilamine (Sigma-Aldrich, St. Louis, MO, USA) ($1 \cdot 10^{-5}$ mol/L concentration in organ bath) was added. The first cumulative concentration-response curve was determined to RAMH (10 nM – 10 mM) at an increasing concentrations spaced by three or 3.3-fold. The second to fourth curve was measured against increasing antagonist concentration (incubation time 20 min). The pA_2-values were calculated according to Arunlakshana and Schild. [Br J Pharmacol Chemother. 14 (1959) 48–58] Statistical analysis was carried out with the Students' t-test. In all tests, a $p<0.05$ was considered statistically significant. The pA_2 values were compared with the affinity of thioperamide (Sigma-Aldrich, St. Louis, MO, USA).

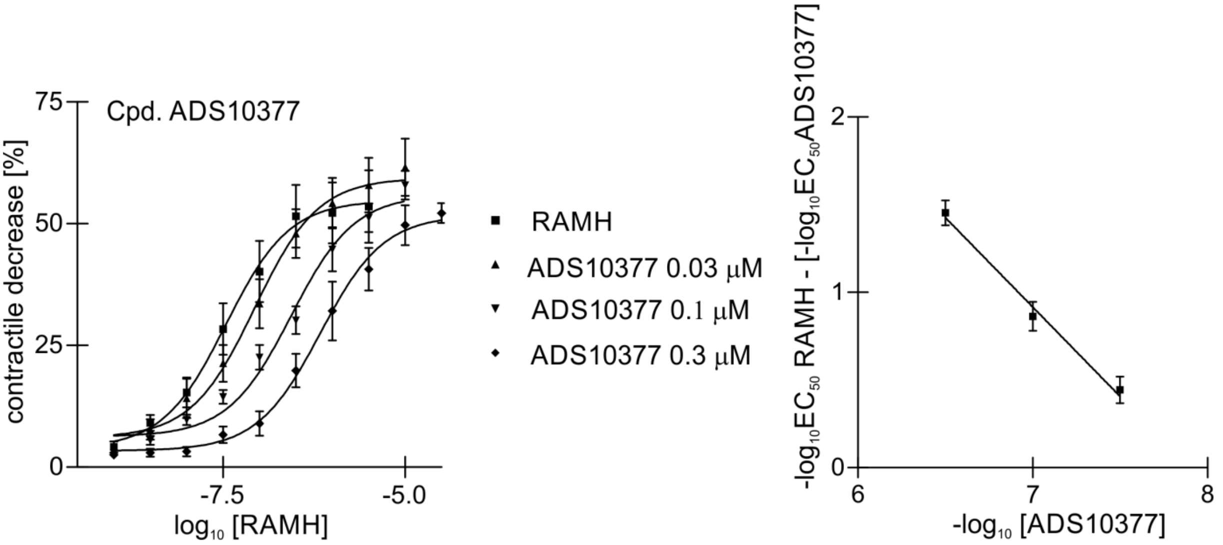

Figure S27. Twitch reduction of electrically stimulated guinea-pig ileum by R-(α)-methylhistamine (RAMH) in the absence (■) and presence (▲,▼, ♦) compound ADS10377.

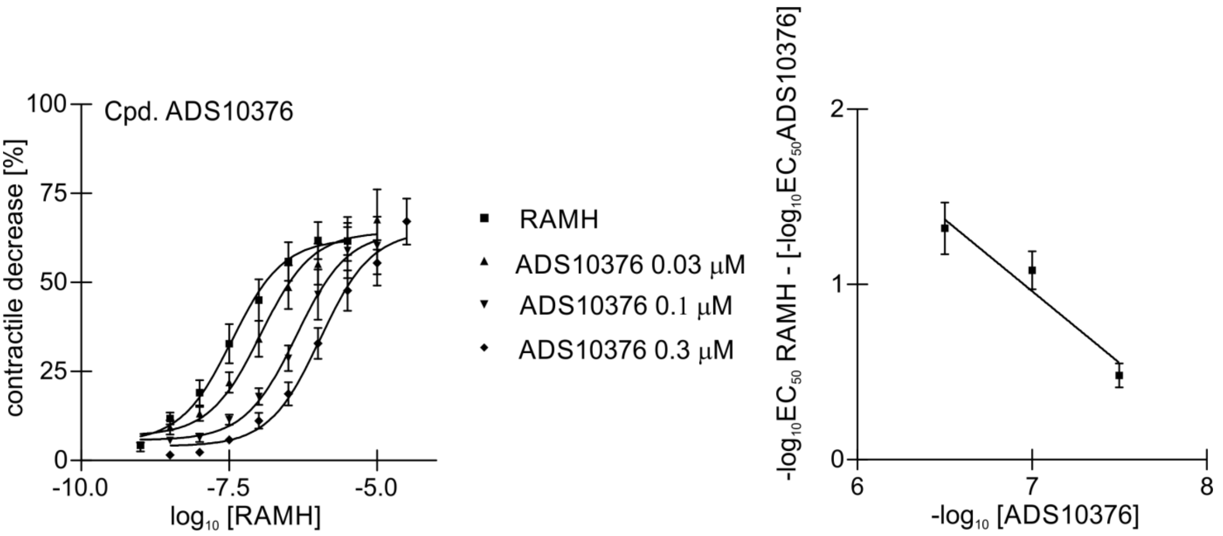

Figure S28. Twitch reduction of electrically stimulated guinea-pig ileum by R-(α)-methylhistamine (RAMH) in the absence (■) and presence (▲,▼, ♦) compound ADS10376.

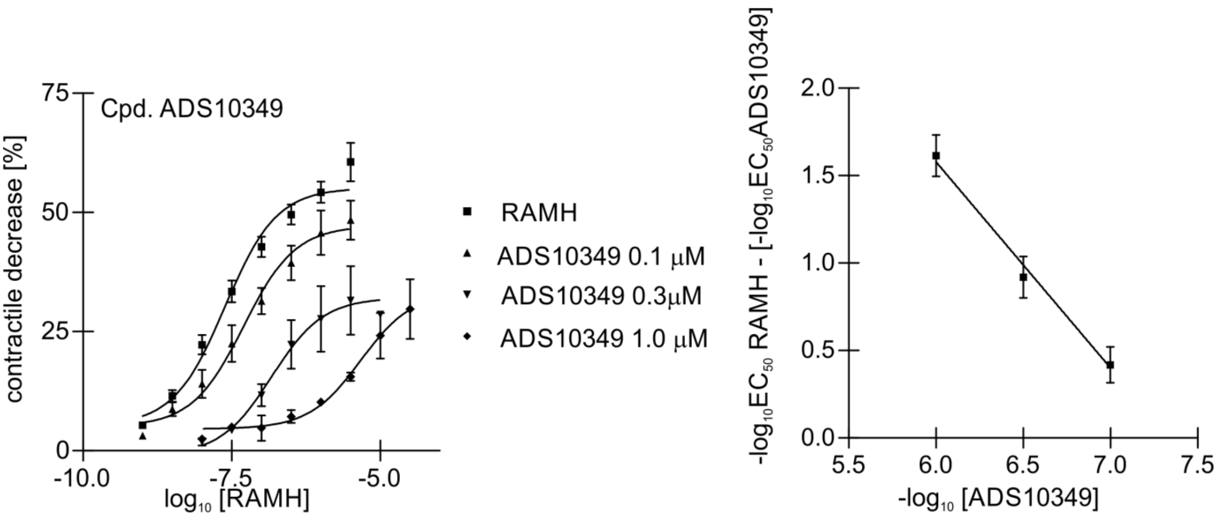

Figure S29. Twitch reduction of electrically stimulated guinea-pig ileum by R-(α)-methylhistamine (RAMH) in the absence (■) and presence (▲,▼, ♦) compound ADS10349.

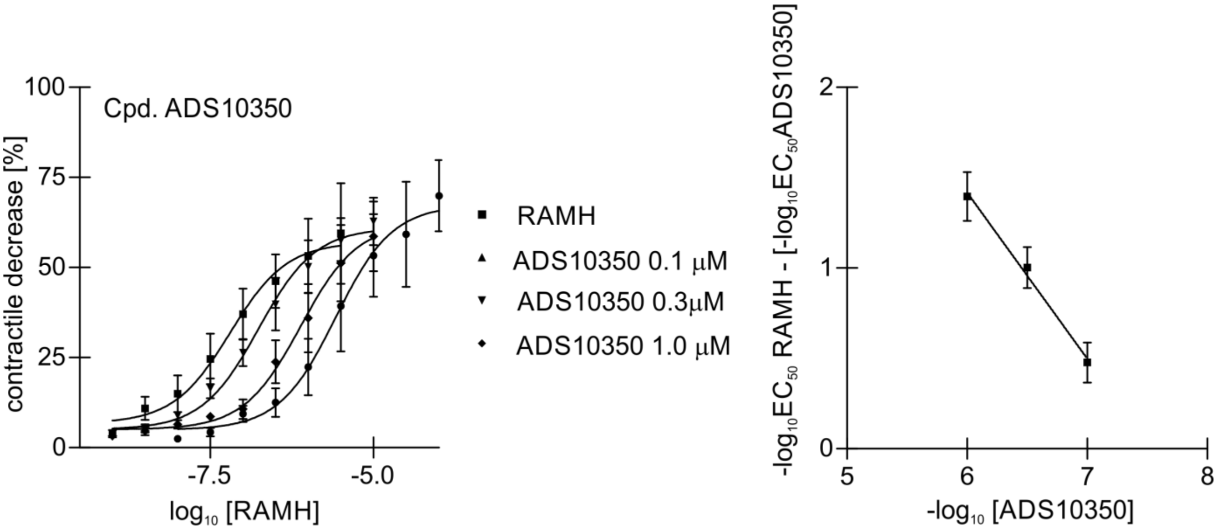

Figure S30. Twitch reduction of electrically stimulated guinea-pig ileum by R-(α)-methylhistamine (RAMH) in the absence (■) and presence (▲,▼, ♦) compound ADS10350.

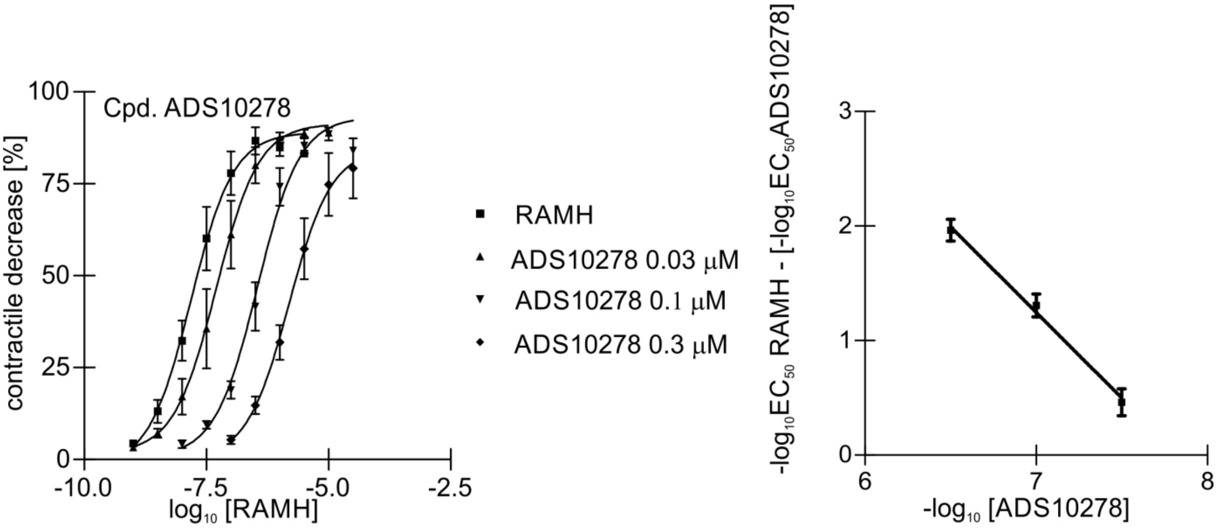

Figure S31. Twitch reduction of electrically stimulated guinea-pig ileum by R-(α)-methylhistamine (RAMH) in the absence (■) and presence (▲,▼, ♦) compound ADS10278.

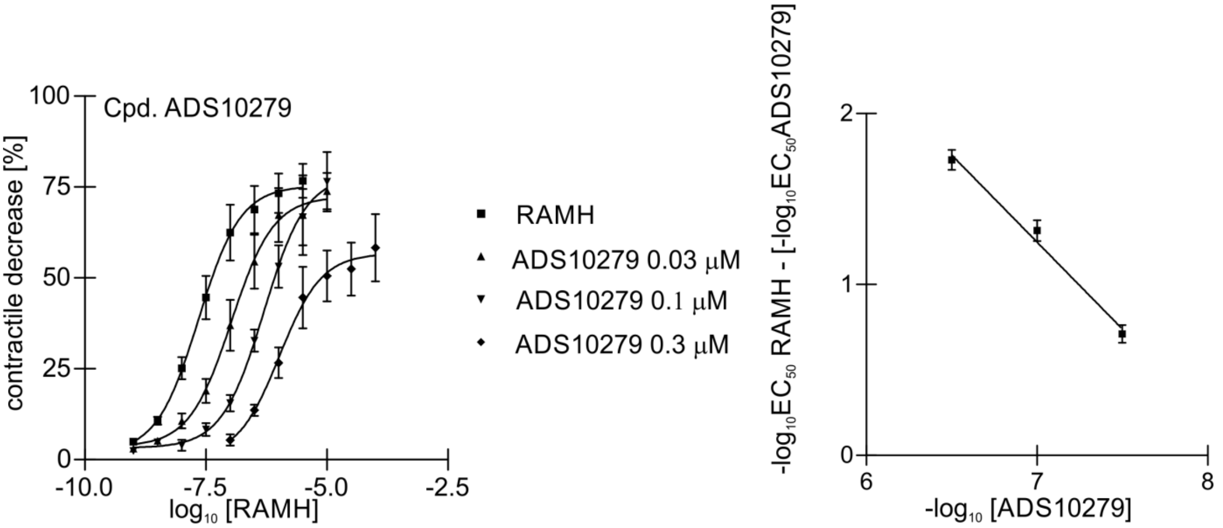

Figure S32. Twitch reduction of electrically stimulated guinea-pig ileum by R-(α)-methylhistamine (RAMH) in the absence (■) and presence (▲,▼, ♦) compound ADS10279.

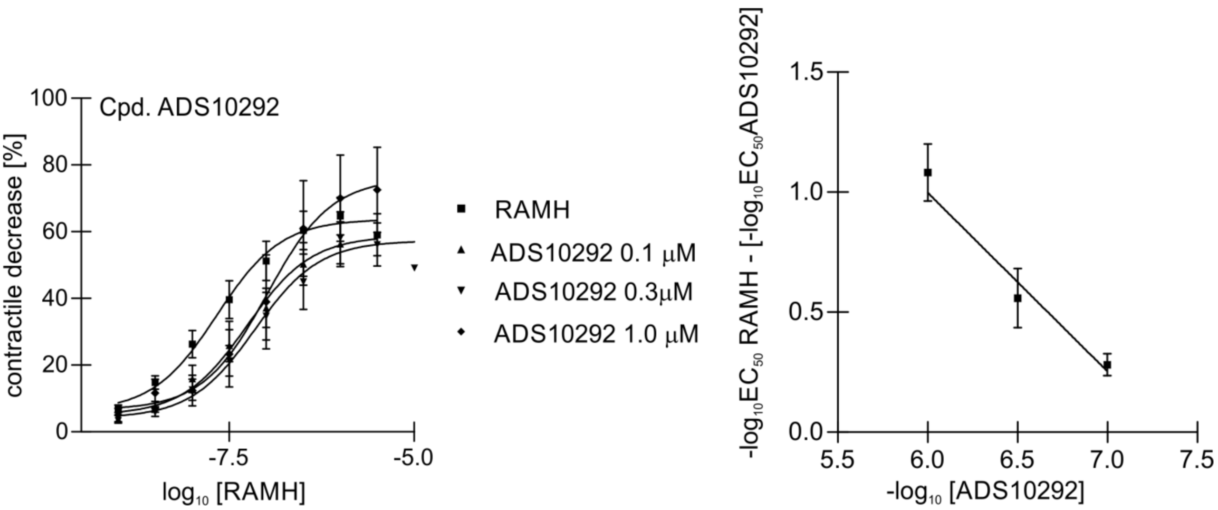

Figure S33. Twitch reduction of electrically stimulated guinea-pig ileum by R-(α)-methylhistamine (RAMH) in the absence (■) and presence (▲,▼, ♦) compound ADS10292.

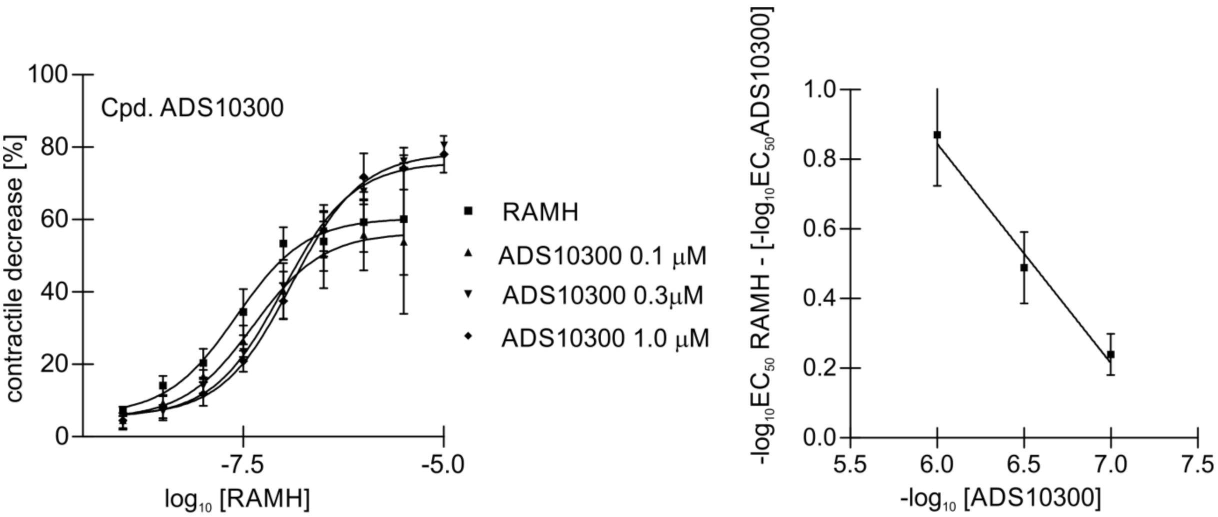

Figure S34. Twitch reduction of electrically stimulated guinea-pig ileum by R-(α)-methylhistamine (RAMH) in the absence (■) and presence (▲,▼, ♦) compound ADS10300.

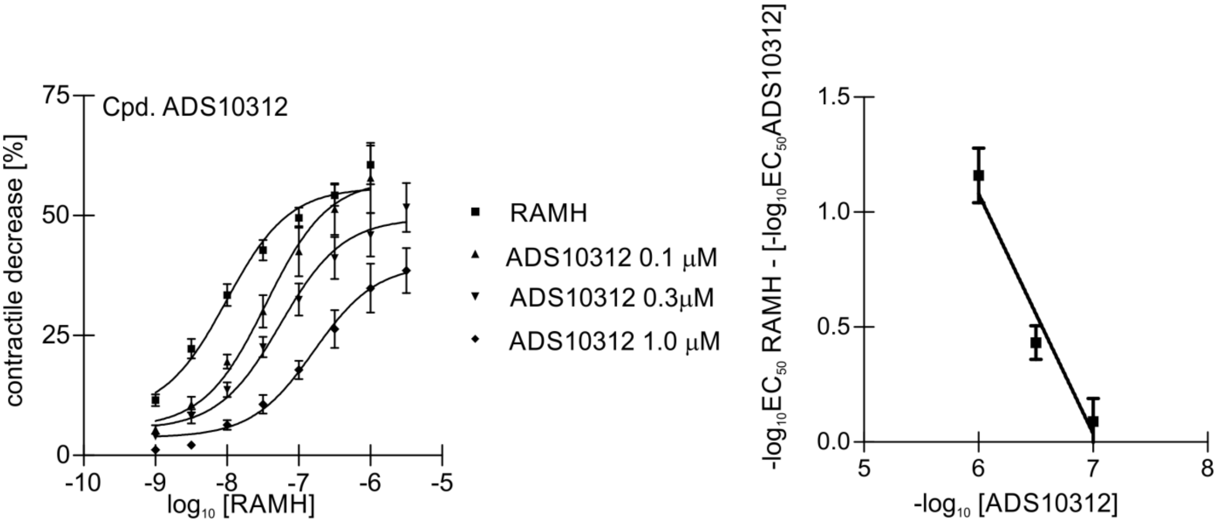

Figure S35. Twitch reduction of electrically stimulated guinea-pig ileum by R-(α)-methylhistamine (RAMH) in the absence (■) and presence (▲,▼, ♦) compound ADS10312.

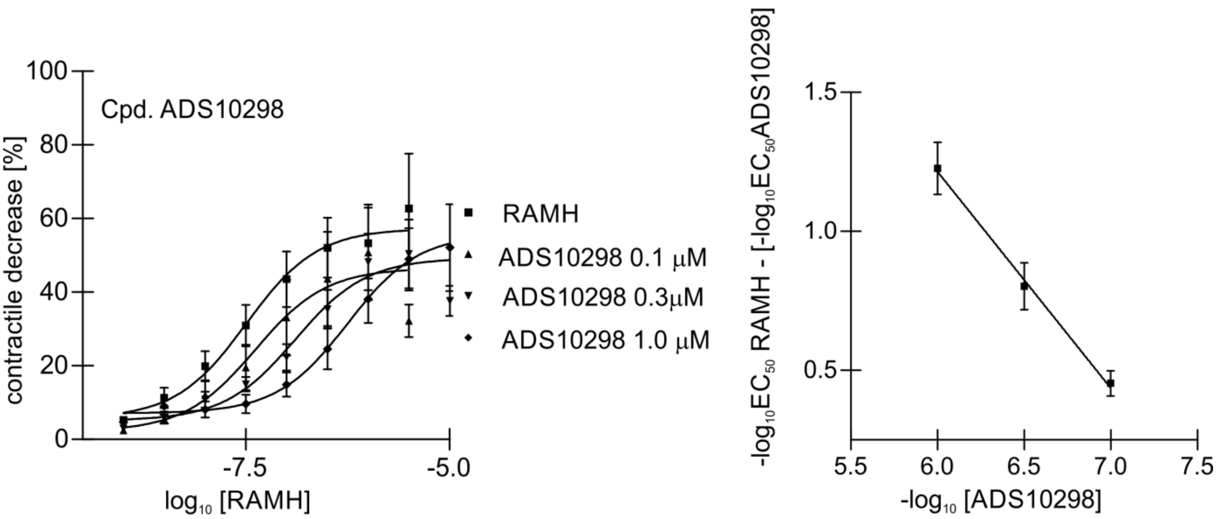

Figure S36. Twitch reduction of electrically stimulated guinea-pig ileum by R-(α)-methylhistamine (RAMH) in the absence (■) and presence (▲,▼, ♦) compound ADS10298.

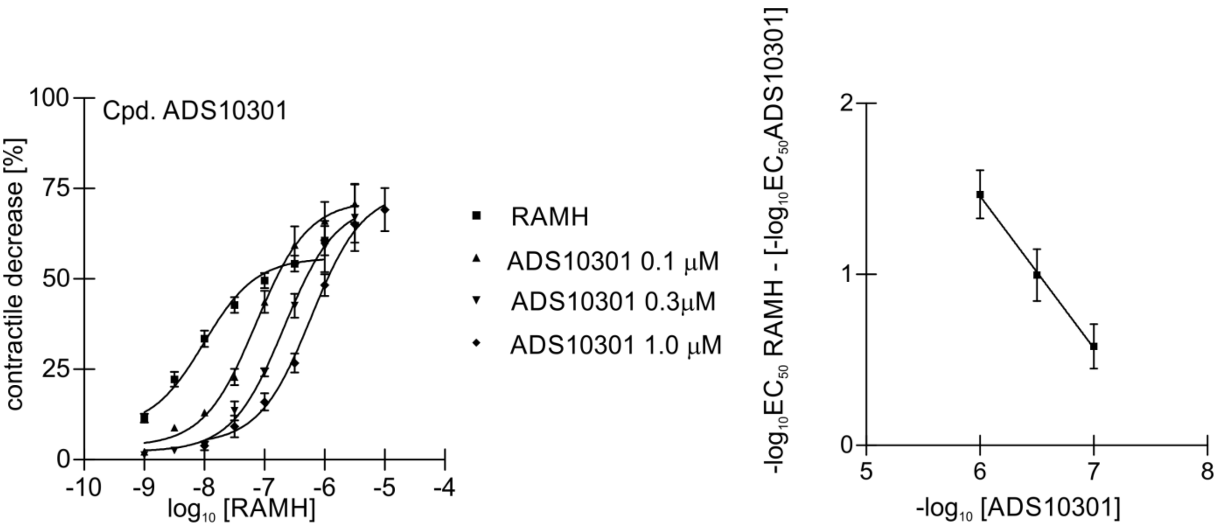

Figure S37. Twitch reduction of electrically stimulated guinea-pig ileum by R-(α)-methylhistamine (RAMH) in the absence (■) and presence (▲,▼, ♦) compound ADS10301.

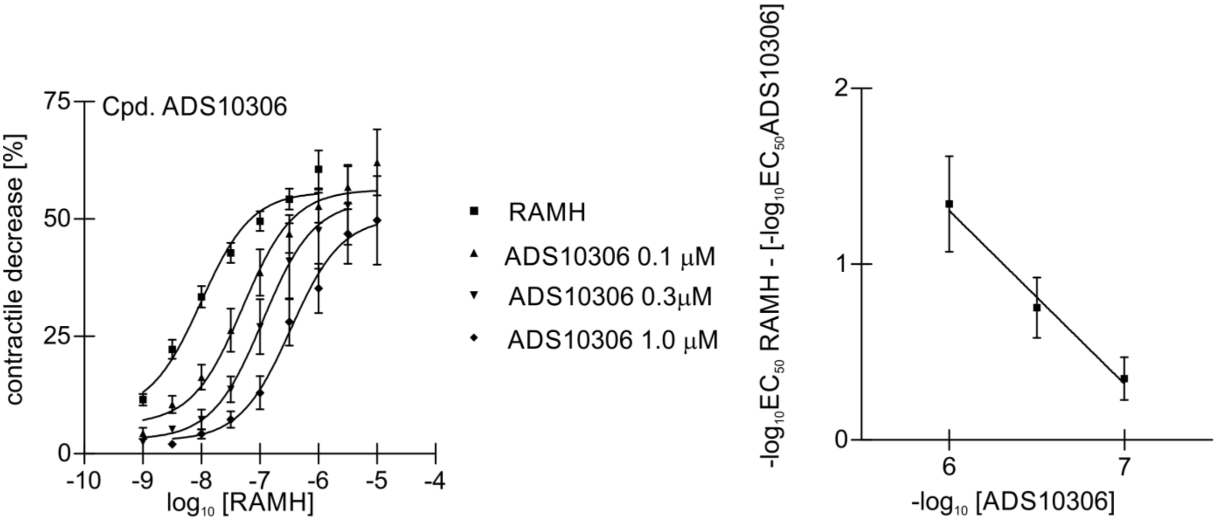

Figure S38. Twitch reduction of electrically stimulated guinea-pig ileum by R-(α)-methylhistamine (RAMH) in the absence (■) and presence (▲,▼, ♦) compound ADS10306.

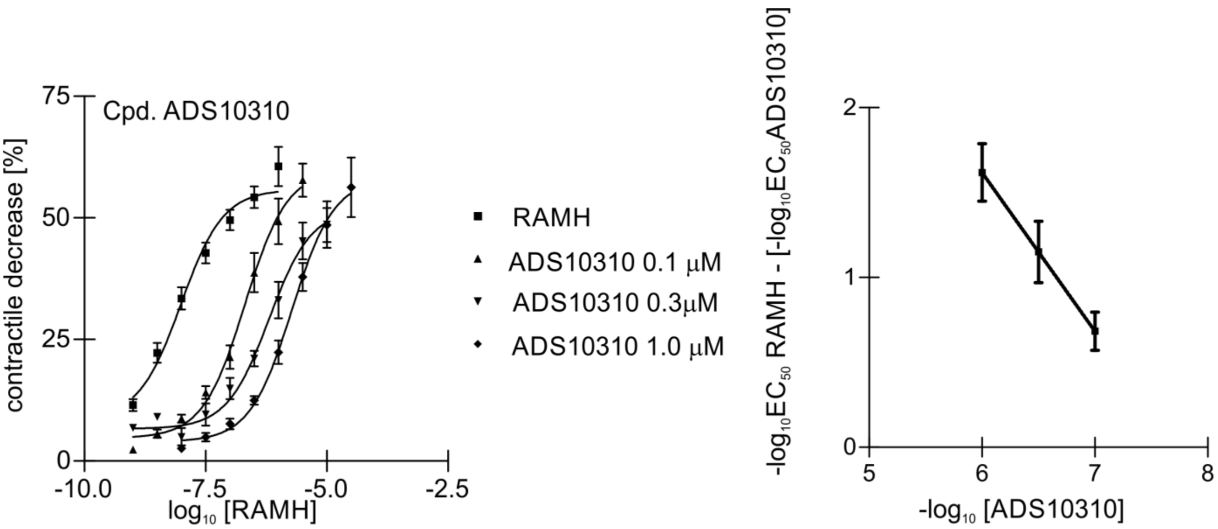

Figure S39. Twitch reduction of electrically stimulated guinea-pig ileum by R-(α)-methylhistamine (RAMH) in the absence (■) and presence (▲,▼, ♦) compound ADS10310.

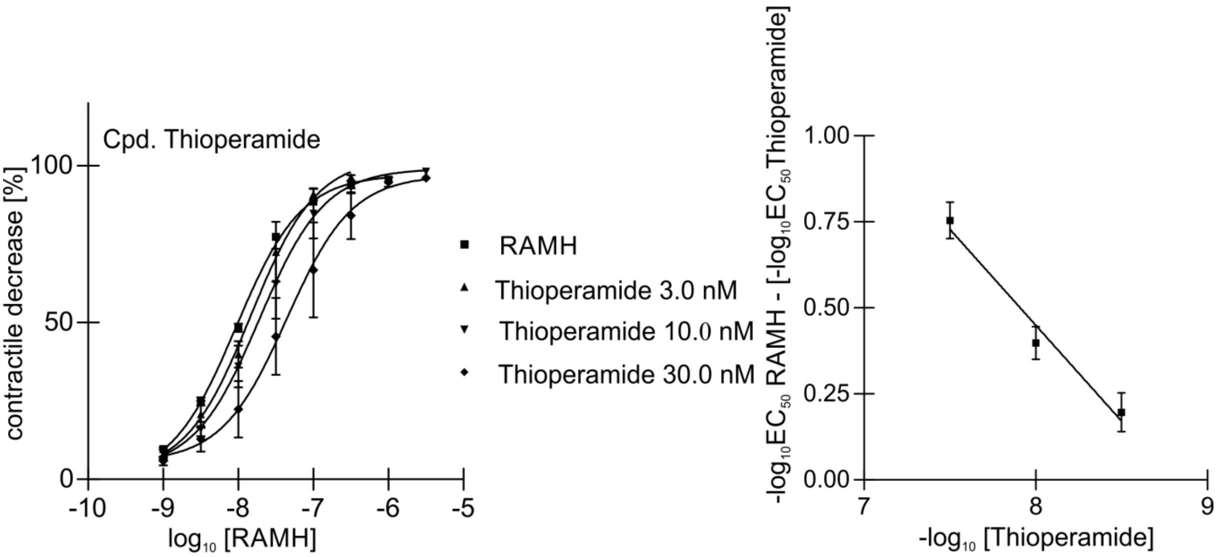

Figure S40. Twitch reduction of electrically stimulated guinea-pig ileum by R-(α)-methylhistamine (RAMH) in the absence (■) and presence (▲,▼, ♦) compound Thioperamide.

3.2 ***Ex vivo*** **assay for histamine H_1R receptor antagonists on guinea pig ileum.**

Male guinea pigs, weighing 300-400 g were euthanized by a blow to the neck. A 20-30 cm length of the distal ileum, apart from the terminal 5 cm was rapidly removed and placed in phosphate buffer at room temperature (pH 7.4) containing (mM) NaCl (136.9); KCl (2.6); KH_2PO_4 (1.47); Na_2HPO_4 (9.58) and indomethacin (Sigma-Aldrich, St. Louis, MO, USA) ($1 \cdot 10^{-6}$ mol/L). The intraluminal content was rinsed and the isolated intestine was cut into 1.5-2 cm segments. The preparations were mounted isotonically in a 20 mL organ bath filled with Krebs buffer: composition (mM) NaCl (118); KCl (5.6); $MgSO_4$ (1.18); $CaCl_2$ (2.5); $NaH_2PO_4 \cdot H_2O$ (1.28); $NaHCO_3$ (25); glucose (5.55), indomethacin ($3 \cdot 10^{-7}$ mol/L), and atropine (Sigma-Aldrich, St. Louis, MO, USA) ($5 \cdot 10^{-8}$ mol/L). The solution was continuously bubbled with a 95 % O_2 : 5 % CO_2 mixture and maintained at 37 °C under a constant load of 0.5 g (Hugo Sachs Hebel-Messvorsatz (Tl-2)/HF-modem; Hugo Sachs Elektronik, Hugstetten, Germany) connected to a pen recorder (Kipp & Zonen BD41, Delft, Holland). During an equilibration period of 40 min, the Krebs buffer was changed every 10 min. The first cumulative concentration-response curve was determined to histamine (Sigma-Aldrich, St. Louis, MO, USA) (10 nM - 10 mM) at increasing concentration spaced by three or 3.3-fold. The second to the fourth (or fifth) curve was measured in the presence of an increasing concentration of antagonist (incubation time 10 min). The pA_2-values were calculated according to Arunlakshana and Schild. [Br J Pharmacol Chemother. 14 (1959) 48–58] Statistical analysis was carried out with the Students' t-test. In all tests, a $p<0.05$ was considered statistically significant. The pA_2 values were compared with the affinity of Pyrilamine (Sigma-Aldrich, St. Louis, MO, USA).

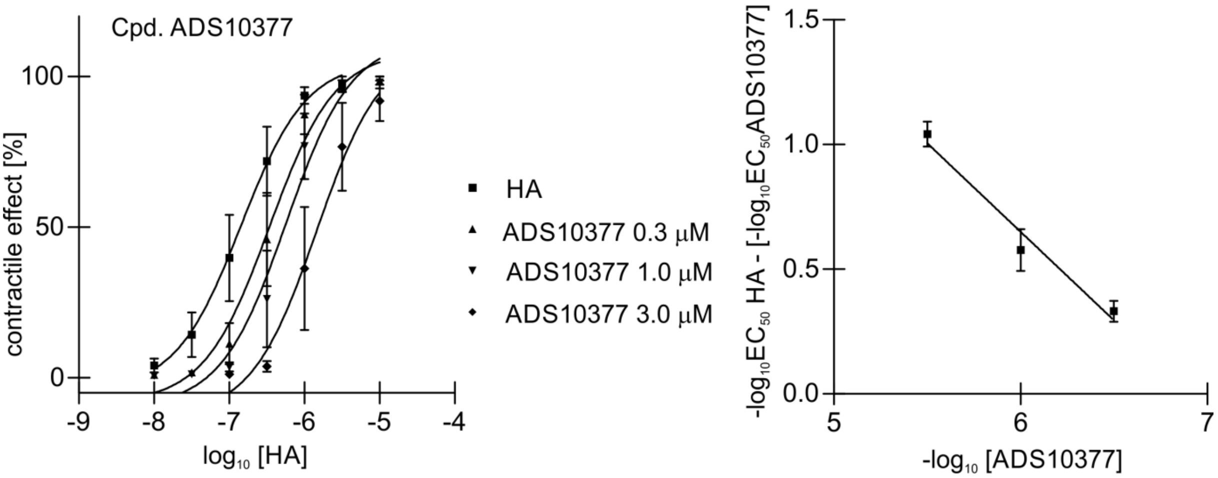

Figure S41. Contraction of guinea-pig ileum by histamine in the absence (■) and presence (▲,▼, ♦) of compound ADS10377

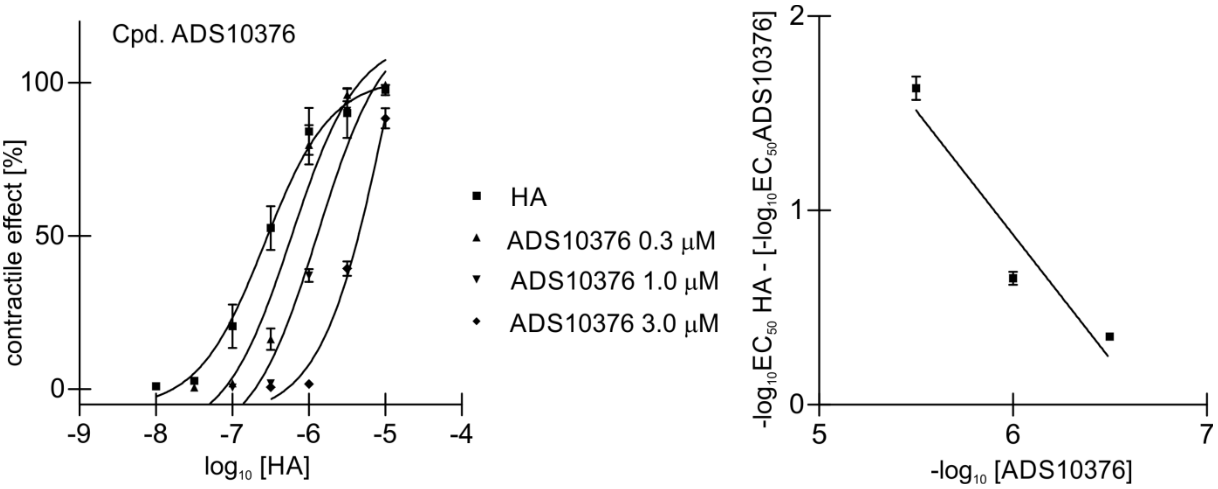

Figure S42. Contraction of guinea-pig ileum by histamine in the absence (■) and presence (▲,▼, ♦) of compound ADS10376

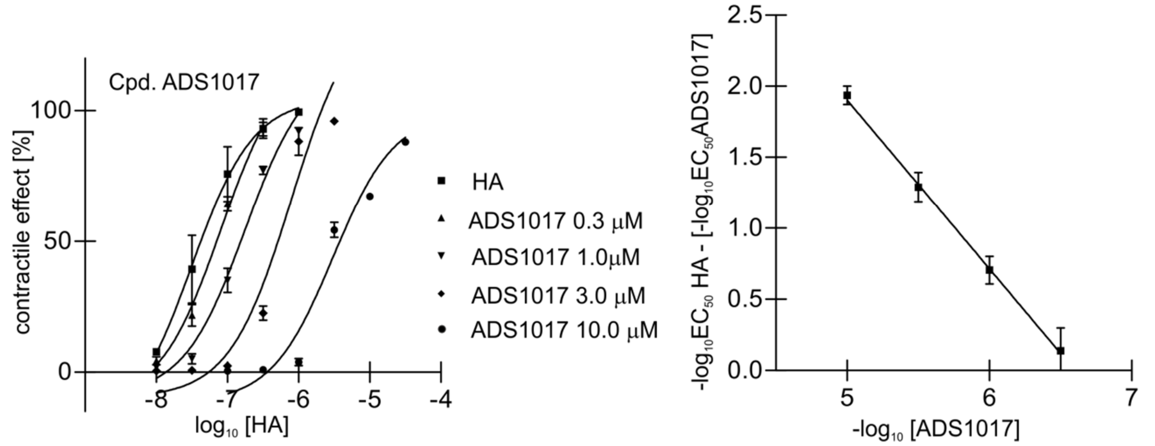

Figure S43. Contraction of guinea-pig ileum by histamine in the absence (■) and presence (▲,▼, ♦, ●) of compound ADS1017

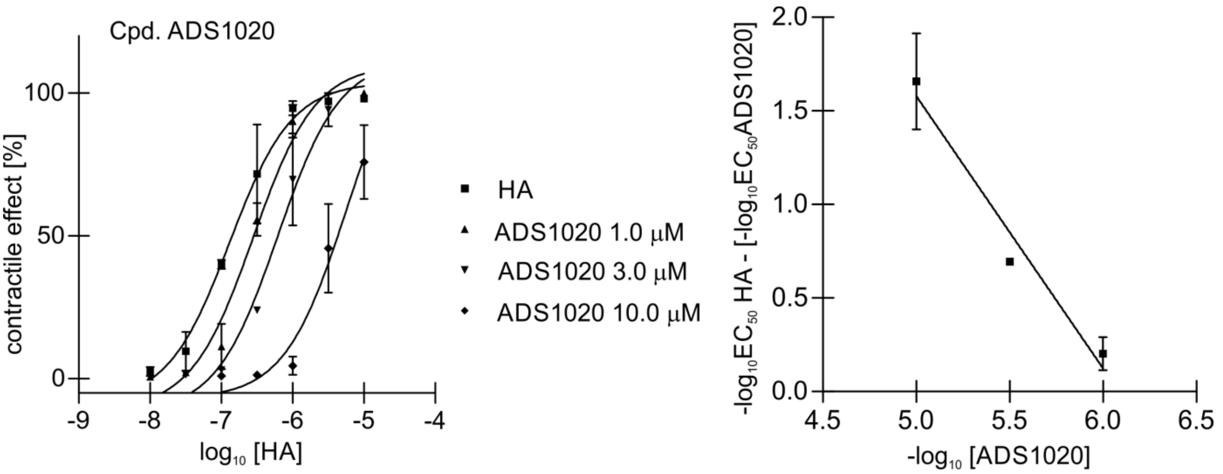

Figure S44. Contraction of guinea-pig ileum by histamine in the absence (■) and presence (▲,▼, ♦) of compound ADS1020

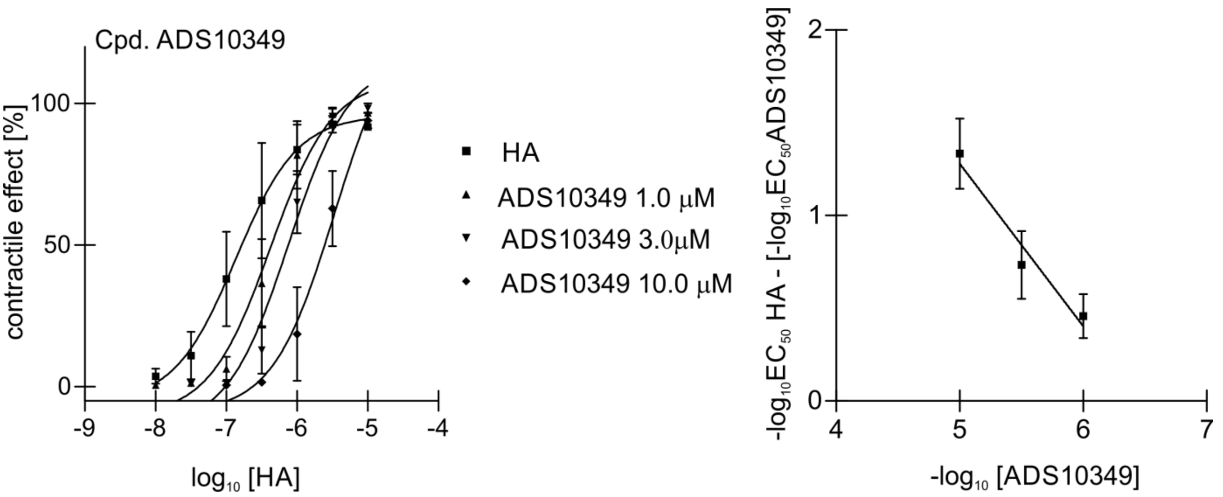

Figure S45. Contraction of guinea-pig ileum by histamine in the absence (■) and presence (▲,▼, ♦) of compound ADS10349

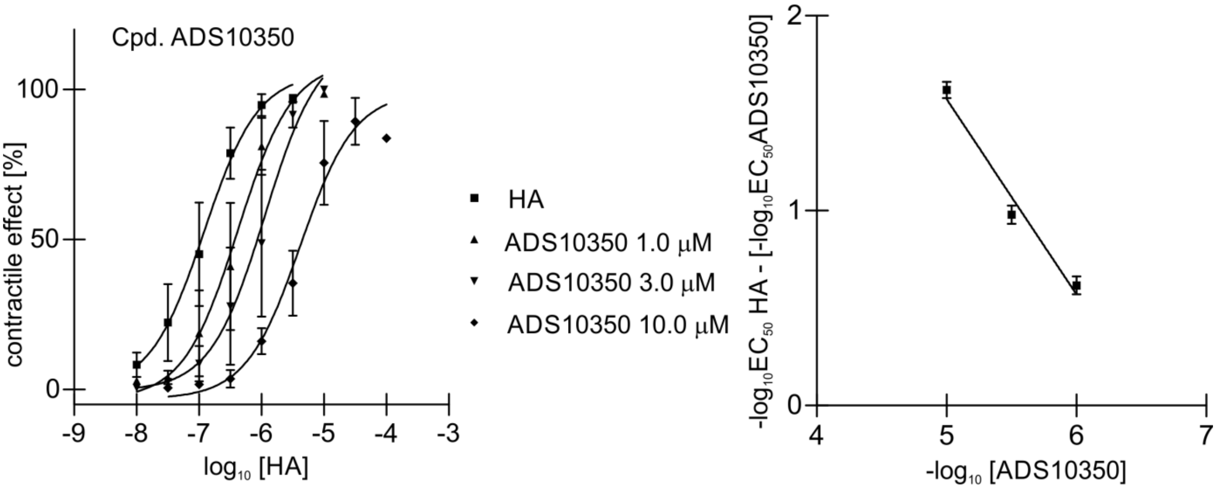

Figure S46. Contraction of guinea-pig ileum by histamine in the absence (■) and presence (▲,▼, ♦) of compound ADS10350

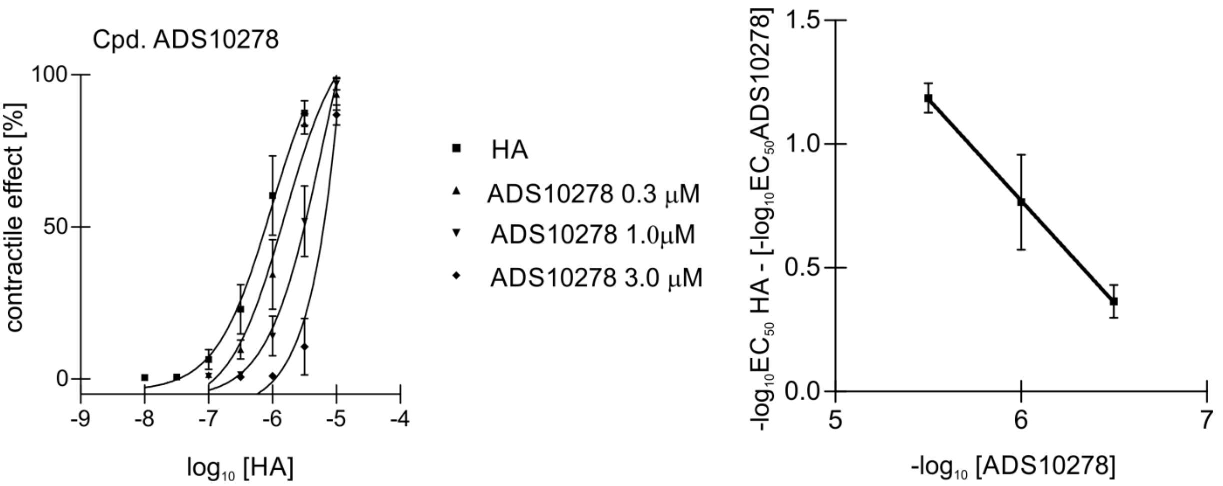

Figure S47. Contraction of guinea-pig ileum by histamine in the absence (■) and presence (▲,▼, ♦) of compound ADS10278

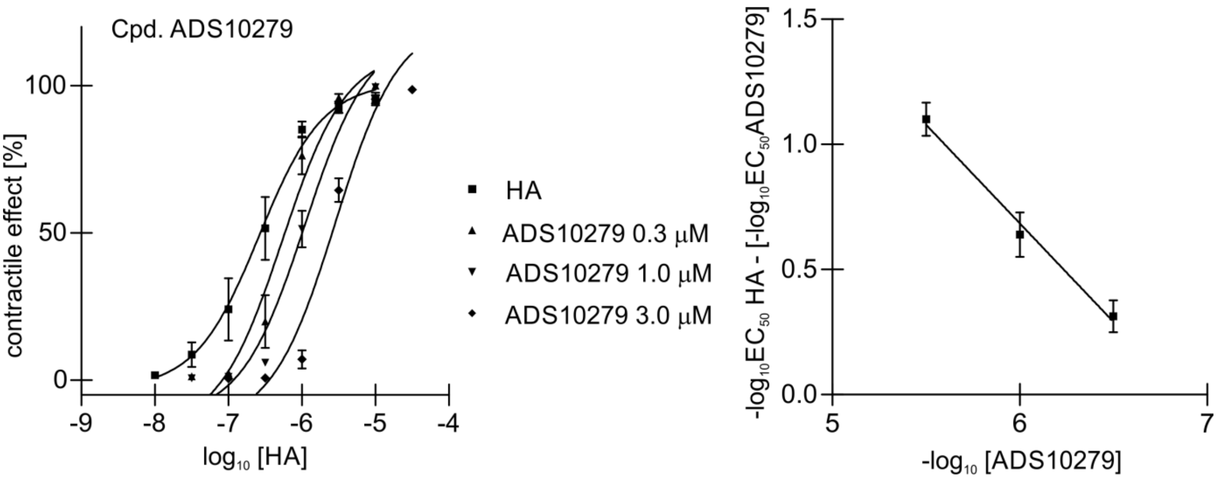

Figure S48. Contraction of guinea-pig ileum by histamine in the absence (■) and presence (▲,▼, ♦) of compound ADS10279

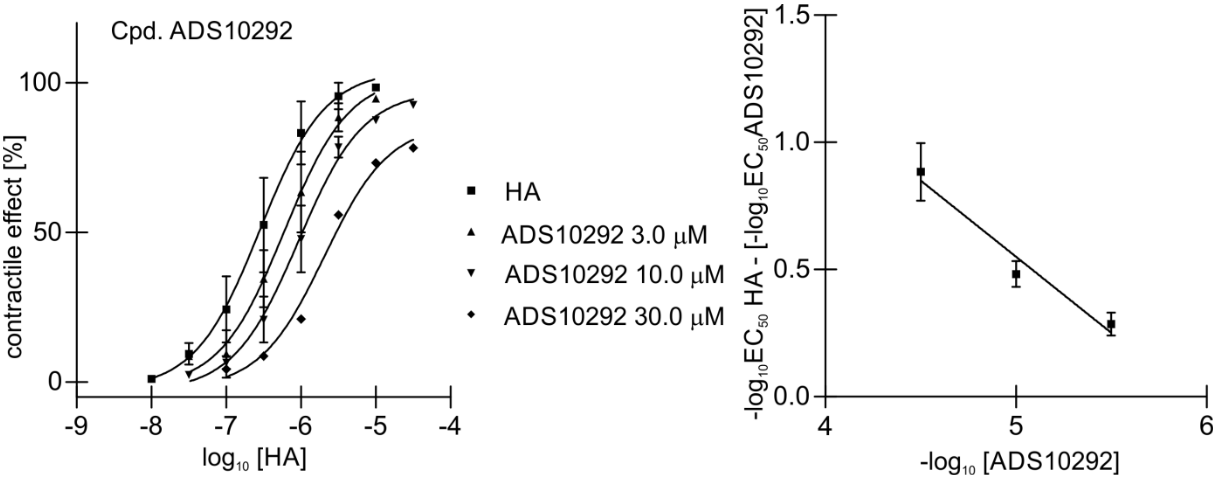

Figure S49. Contraction of guinea-pig ileum by histamine in the absence (■) and presence (▲,▼, ♦) of compound ADS10292

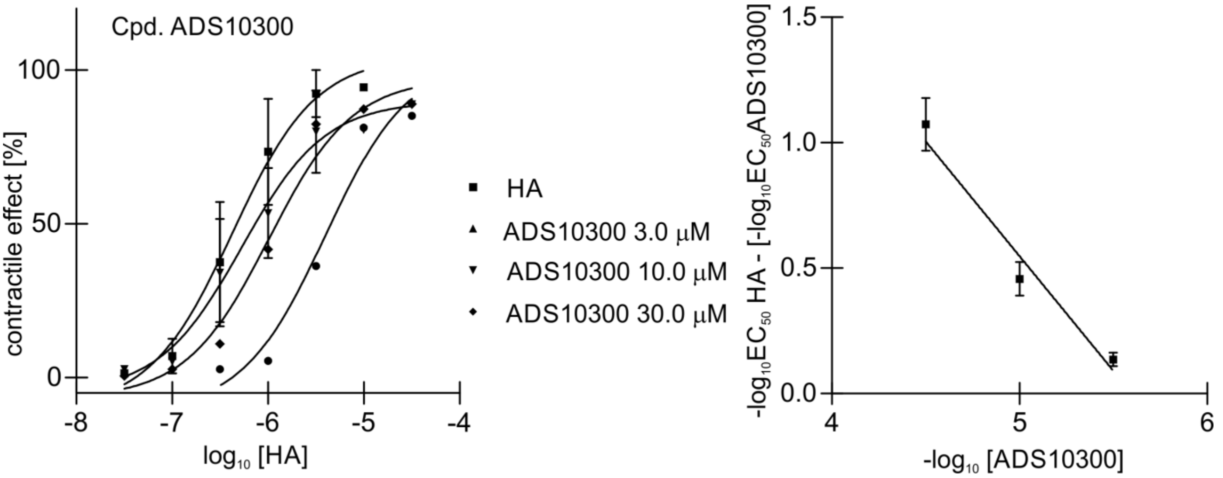

Figure S50. Contraction of guinea-pig ileum by histamine in the absence (■) and presence (▲,▼, ♦) of compound ADS10300

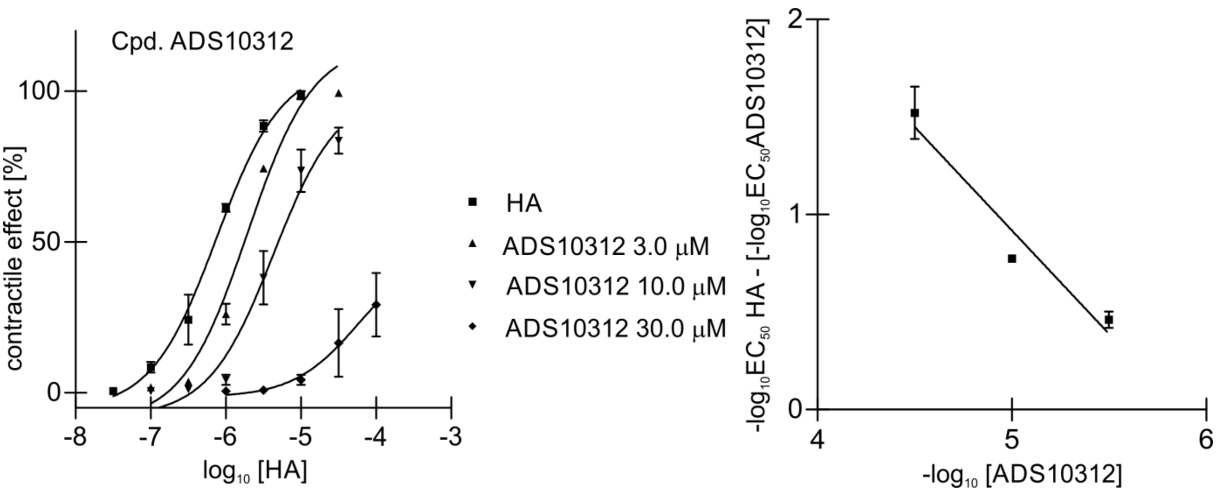

Figure S51. Contraction of guinea-pig ileum by histamine in the absence (■) and presence (▲,▼, ♦) of compound ADS10312

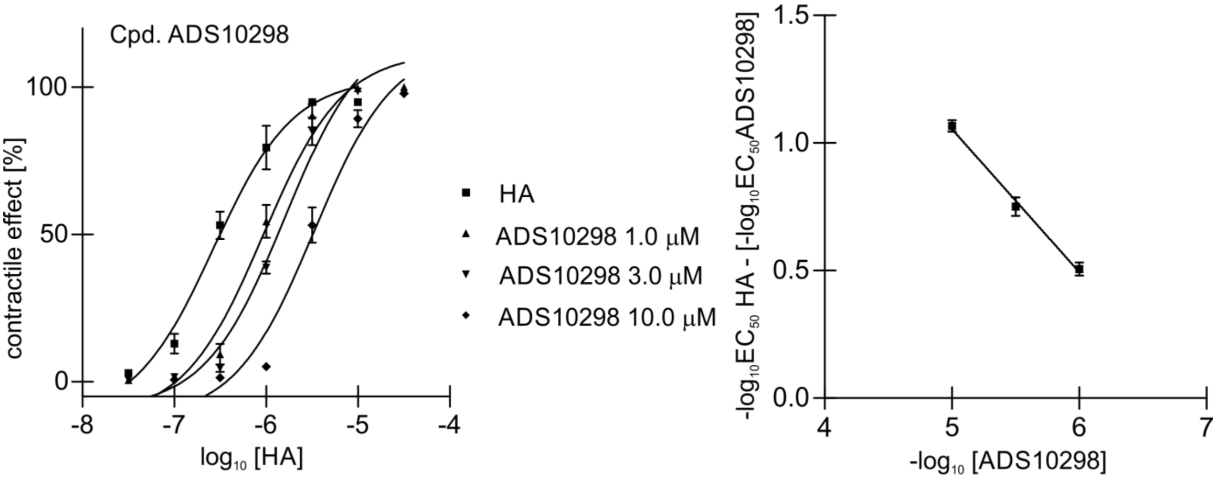

Figure S52. Contraction of guinea-pig ileum by histamine in the absence (■) and presence (▲,▼, ♦) of compound ADS10298

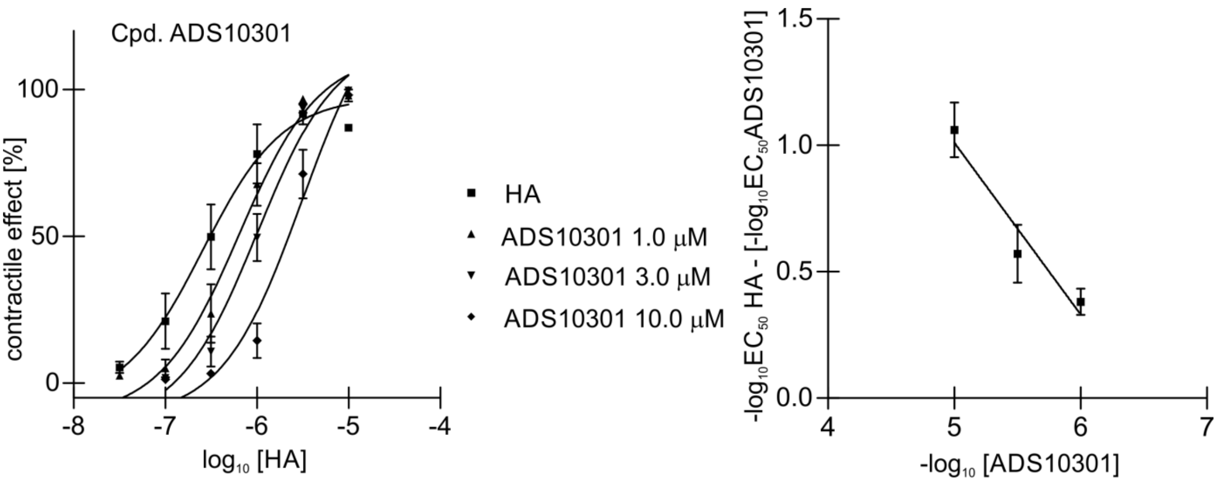

Figure S53. Contraction of guinea-pig ileum by histamine in the absence (■) and presence (▲,▼, ♦) of compound ADS10301

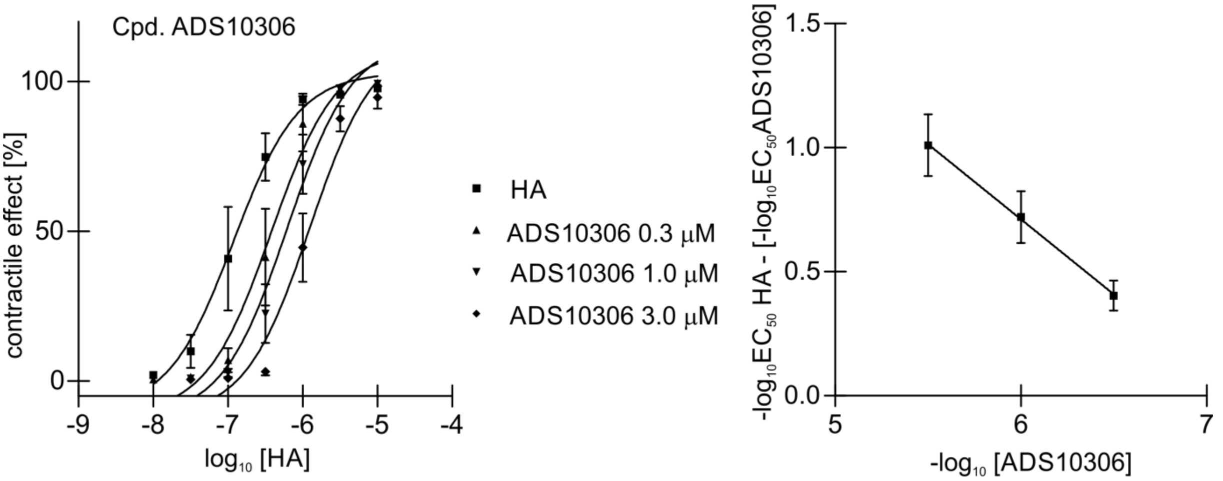

Figure S54. Contraction of guinea-pig ileum by histamine in the absence (■) and presence (▲,▼, ♦) of compound ADS10306

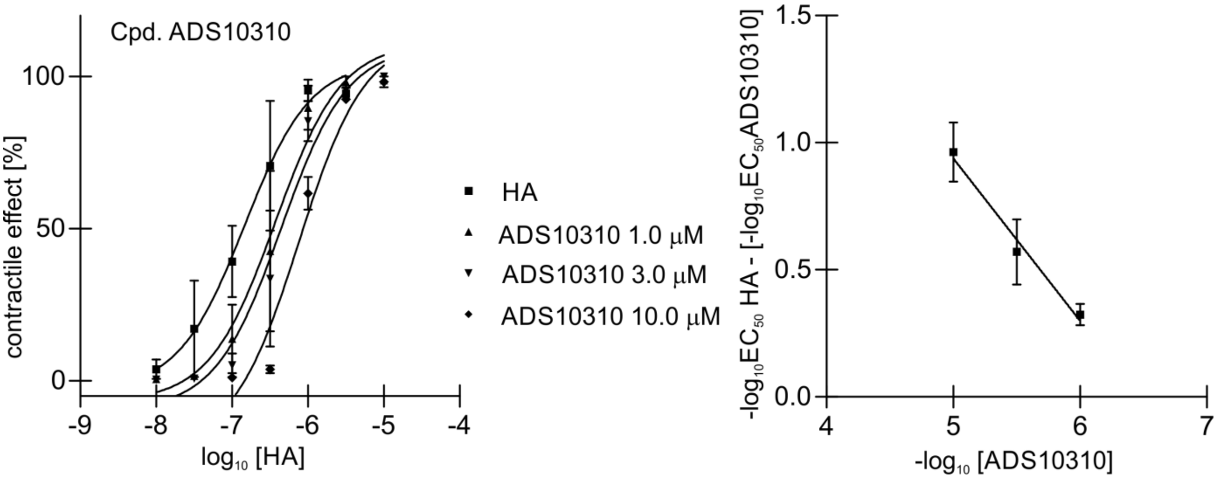

Figure S55. Contraction of guinea-pig ileum by histamine in the absence (■) and presence (▲,▼, ♦) of compound ADS10310

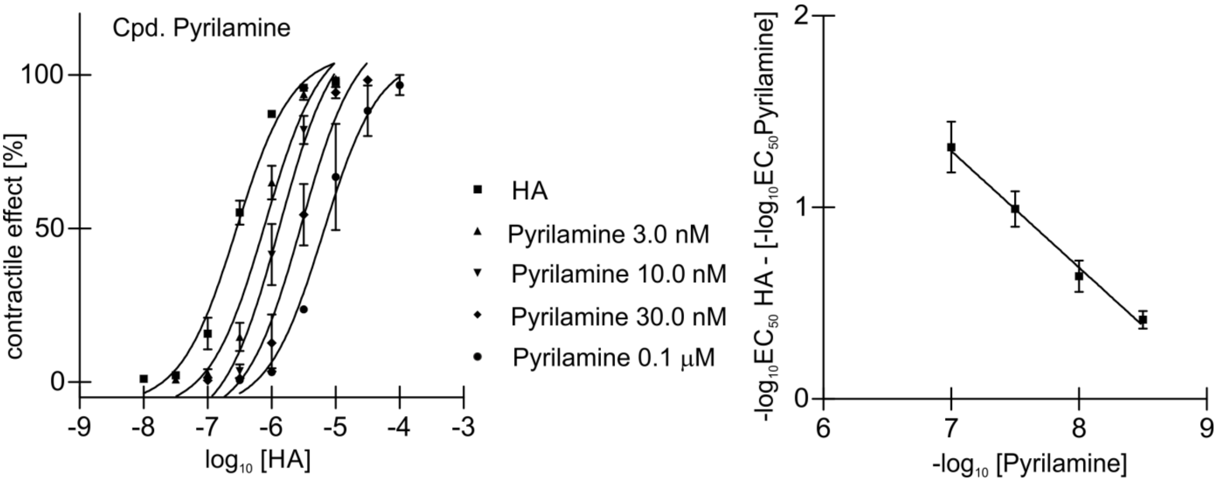

Figure S56. Contraction of guinea-pig ileum by histamine in the absence (■) and presence (▲,▼, ♦, ●) of Pyrilamine

3.3 hH_3R radioligand displacement binding assay.

The radioligand displacement binding assay was performed in membrane fractions of HEK-293 cells stably expressing hH_3R. Cell cultivation and membrane preparation was performed according to Kottke et al. [T. Kottke, K. Sander, L. Weizel, E.H. Schneider, R. Seifert, H. Stark, Eur J Pharmacol. 654 (2011) 200–208.] For the radioligand displacement assay, radioactively labeled [^{3}H]Nα-methylhistamine was used at a final concentration of 2 nM (KD = 3.08 nM). The total assay volume was set to 200 µL. The compounds were tested in several appropriate concentrations between 100 µM and 0.1 nM. Pipetting was partly done by Freedom Evo® (Tecan). Pitolisant was used to determine non-specific binding at a concentration of 10 µM. The membrane fraction (20 µg/well), test compounds and radiolabeled ligand were incubated for 90 minutes at 25 °C while shaking. The bound radioligand was separated from the free radioligand by filtration through GF/B filters pre-treated with 0.3 % (m/v) polyethyleneimine using a cell harvester. Radioactivity was determined by liquid scintillation counting using a MicroBeta® Trilux (Perkin Elmer). The data was obtained in duplicates in at least three independent experiments. Non-specific binding was subtracted from the raw data to calculate specific-binding values. The evaluation was performed with GraphPad Prism 6.1 (San Diego, CA, USA) using non-linear regression (one-site competition with a logarithmic scale). The Ki values were calculated from the IC_{50} values using the Cheng-Prusoff equation. [C. Yung-Chi, W.H. Prusoff, Biochem Pharmacol. 22 (1973) 3099–3108] The statistical calculations were performed on $-\log(K_i)$. The mean values and 95 % confidence intervals were transformed to nanomolar concentrations.

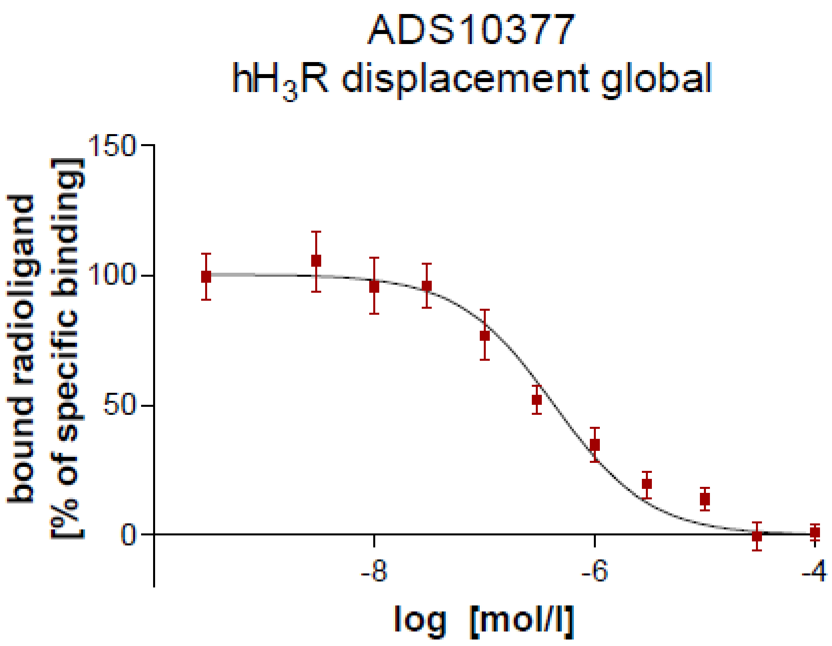

Figure S57. hH3 competition binding curve of compound ADS10377.

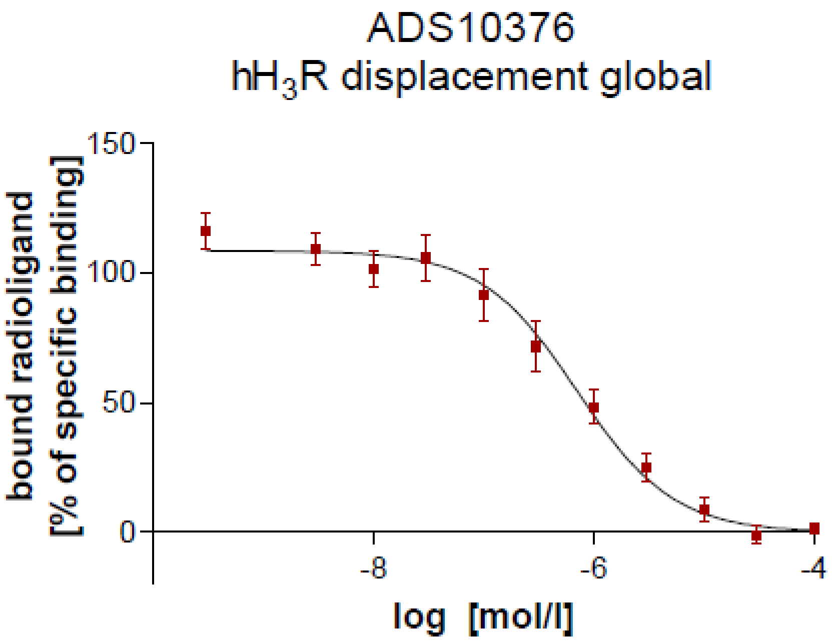

Figure S58. hH3 competition binding curve of compound ADS10376.

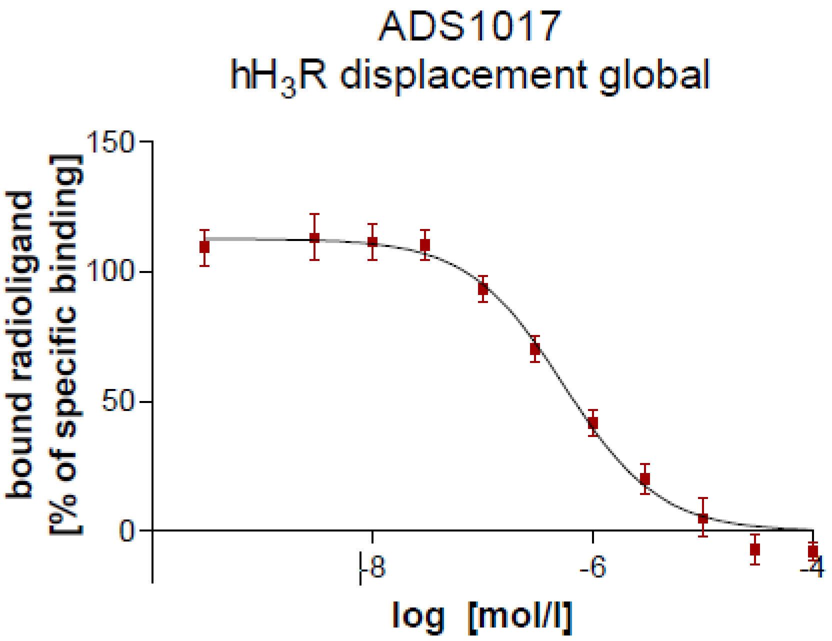

Figure S59. hH3 competition binding curve of compound ADS1017.

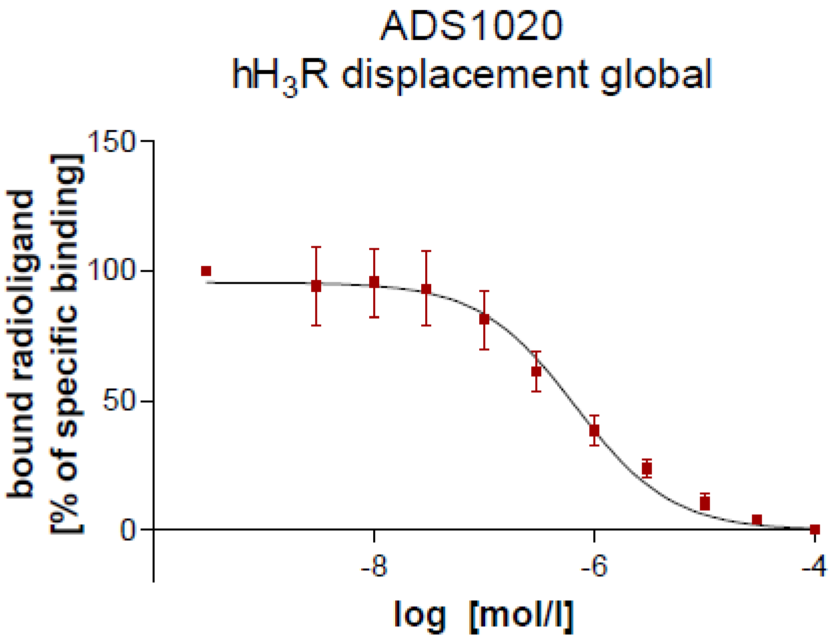

Figure S60. hH3 competition binding curve of compound ADS1020.

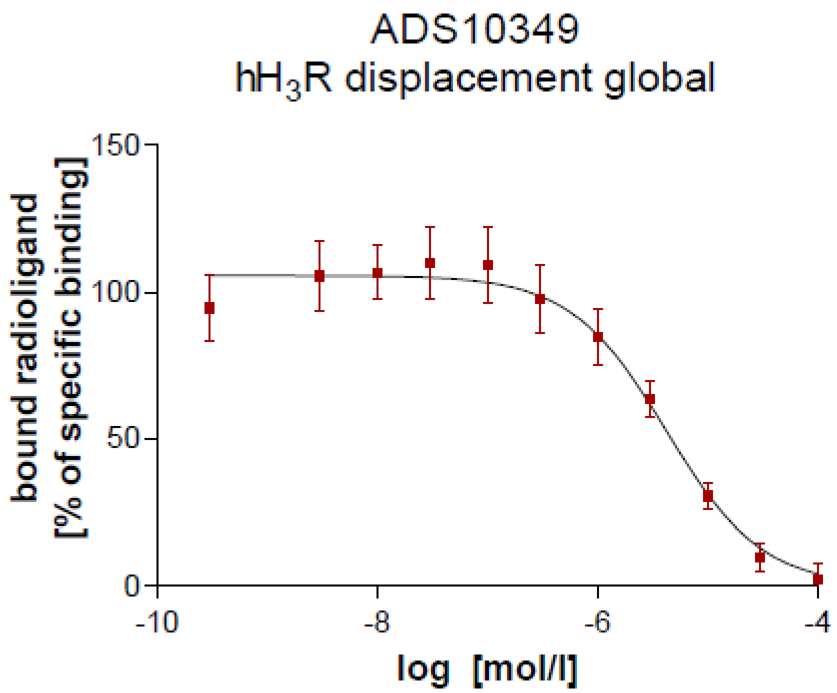

Figure S61. hH3 competition binding curve of compound ADS10349.

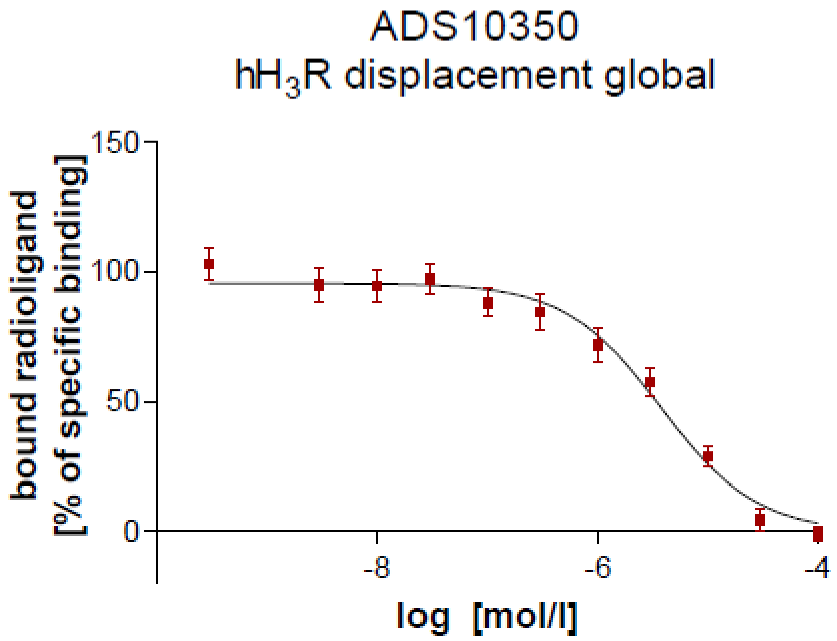

Figure S62. hH3 competition binding curve of compound ADS10350.

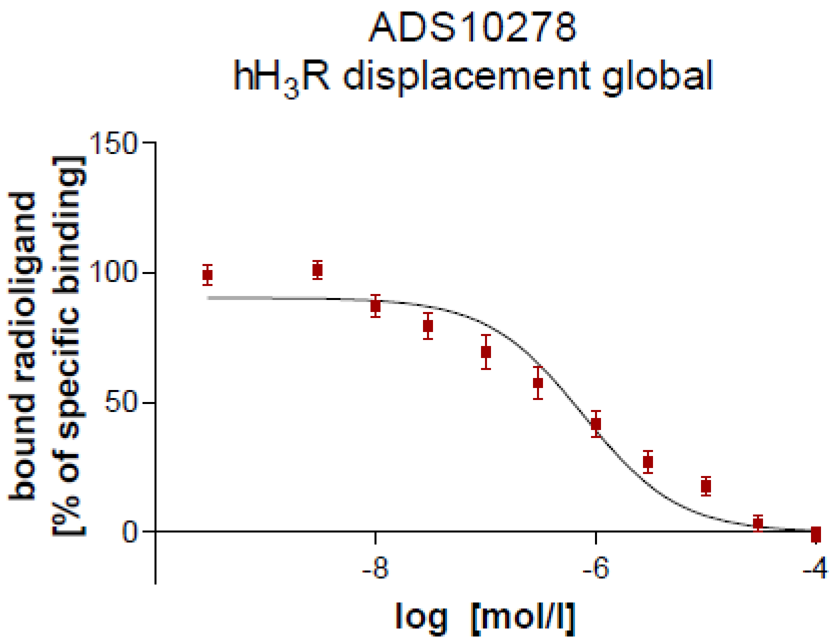

Figure S63. hH3 competition binding curve of compound ADS10278.

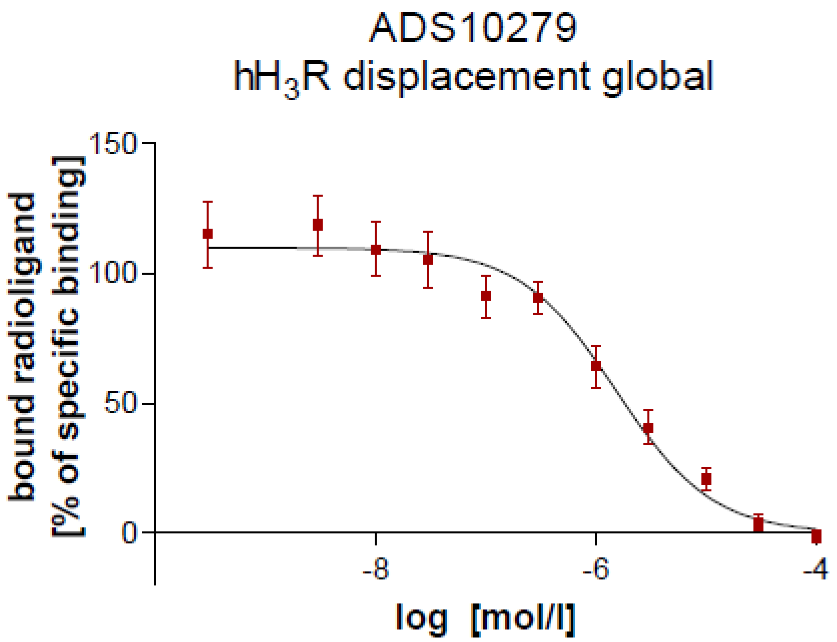

Figure S64. hH3 competition binding curve of compound ADS10279.

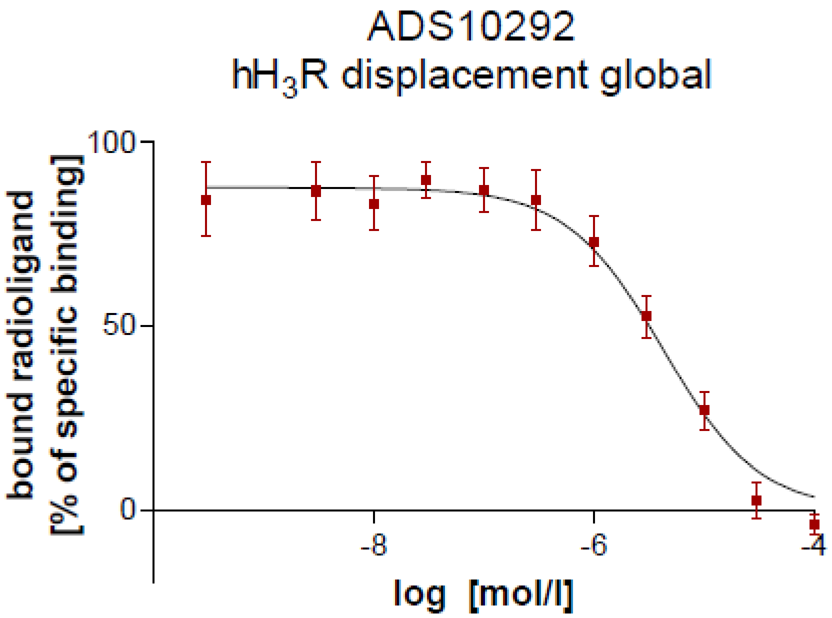

Figure S65. hH3 competition binding curve of compound ADS10292.

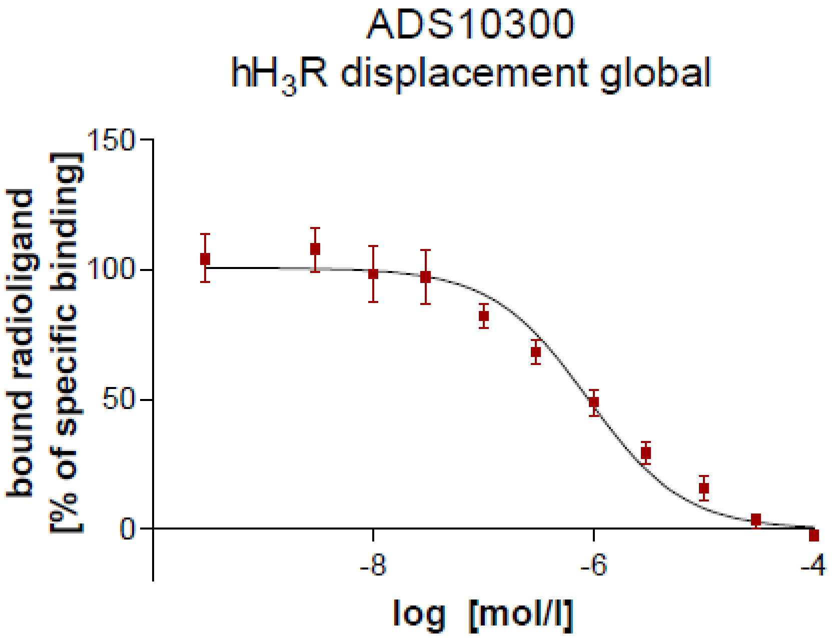

Figure S66. hH3 competition binding curve of compound ADS10300.

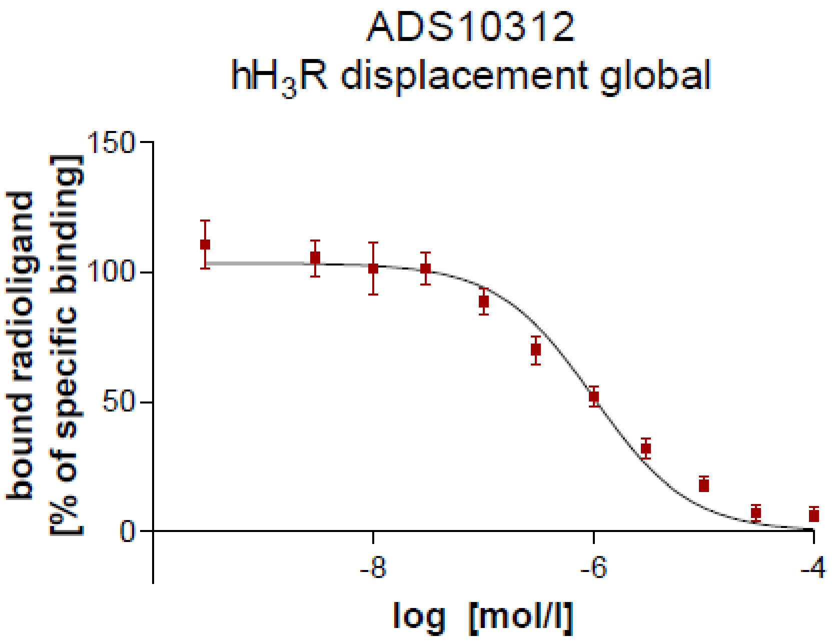

Figure S67. hH3 competition binding curve of compound ADS10312.

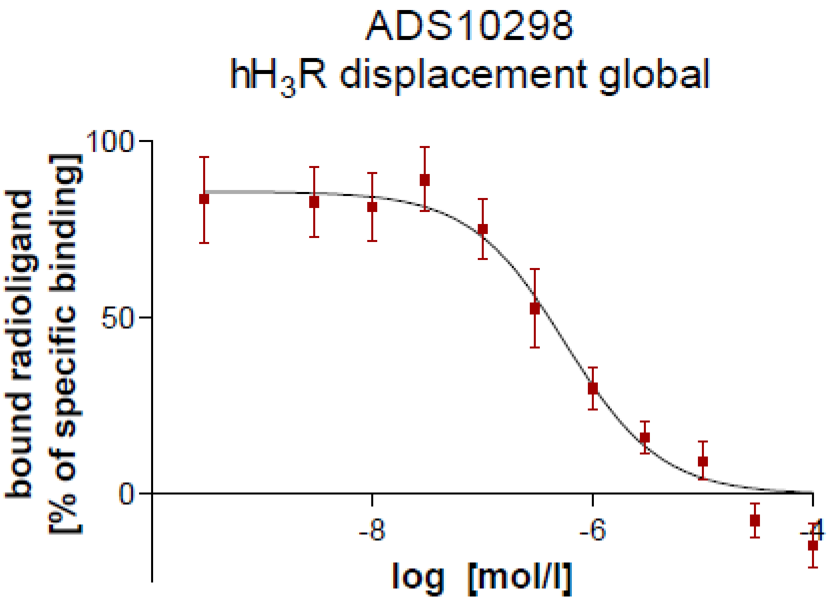

Figure S68. hH3 competition binding curve of compound ADS10298.

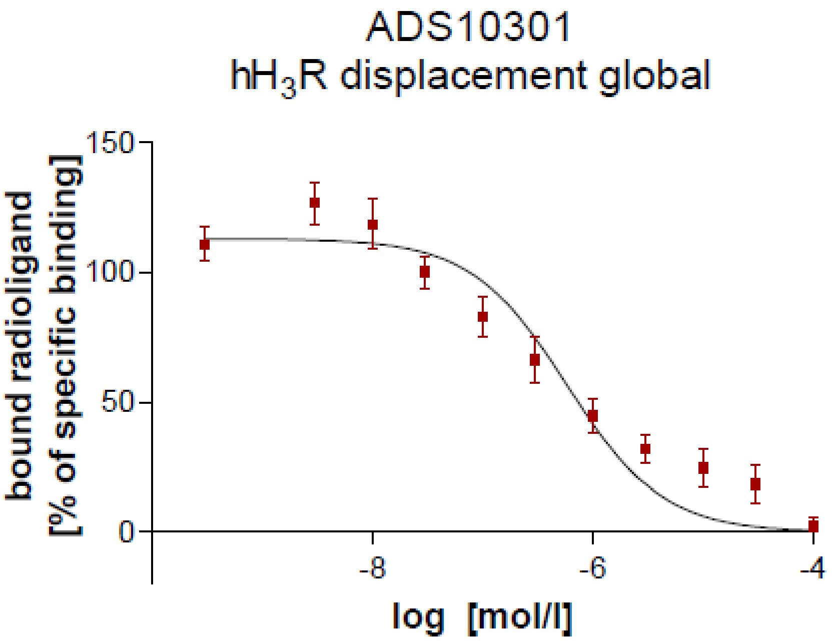

Figure S69. hH₃ competition binding curve of compound ADS10301.

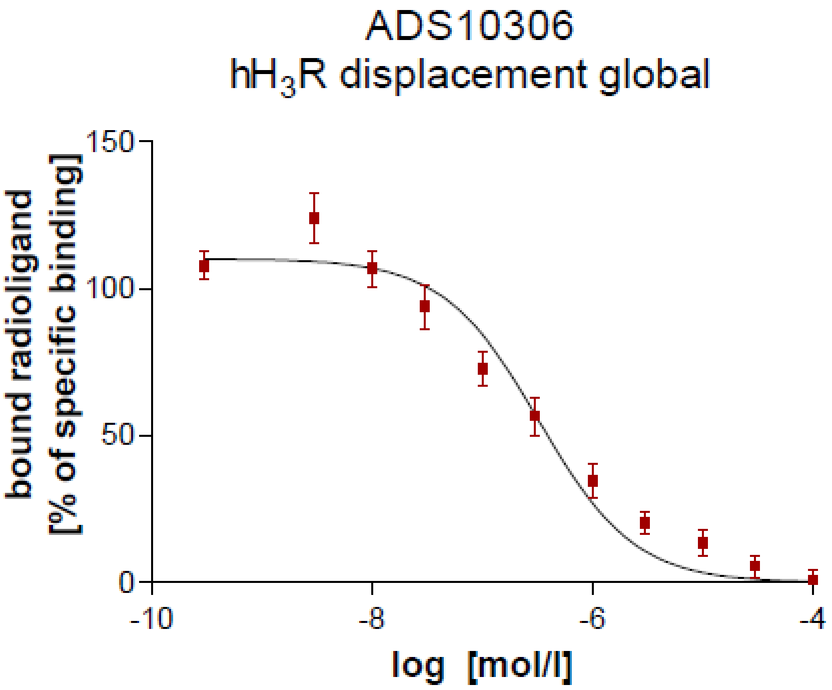

Figure S70. hH₃ competition binding curve of compound ADS10306.

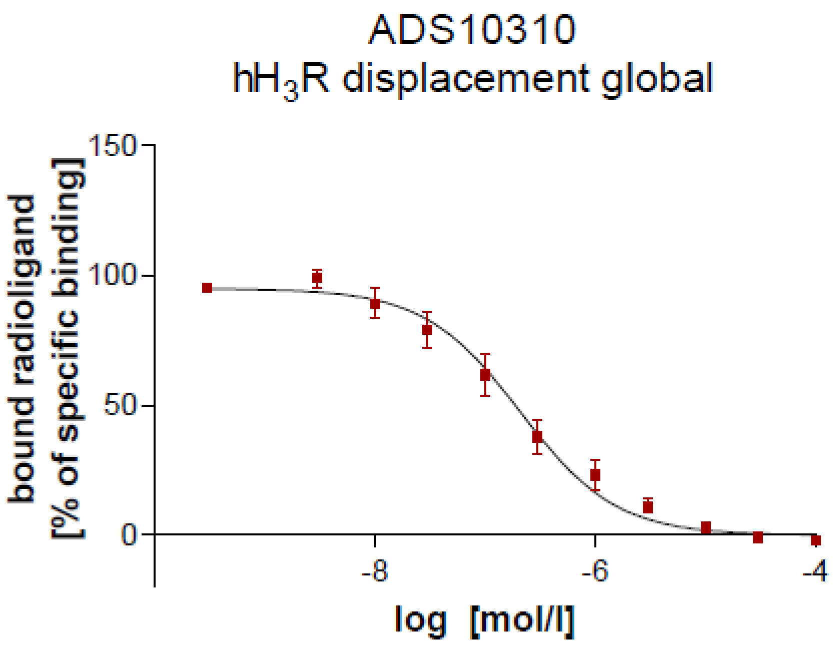

Figure S71. hH$_3$ competition binding curve of compound ADS10310.

3.4. Cell viability.

Table S1. IC_{50} values calculated for ADS10310, ADS1017 and Doxorubicin based on MTT test results after 48 hours treatment of MDA-MB-231 and MCF-7 breast cancer cells and BJ normal skin fibroblast.

Cpd.	IC_{50} [μM]			SI BJ(IC_{50})/MDA-MB-231 (IC_{50})	SI BJ(IC_{50})/MCF-7 (IC_{50})
	MDA-MB-231	MCF-7	BJ		
**ADS1017**	19.86	24.71	38.97	1.96	1.58
**ADS10310**	115.16	82.94	231.47	2.01	2.79
**Doxorubicin**	1.85	2.12	4.87	2.63	2.20

SI – selectivity index

Table S2. Cell viability (%) for ADS10310, ADS1017 based on MTT test results after 48-hour treatment of MDA-MB-231 and MCF-7 breast cancer cells.

Cpd.	Cell line	Cell viability (%) ± SD							
		10 μM	20 μM	25 μM	40 μM	50 μM	75 μM	100 μM	125 μM
ADS1017	**MDA-MB-231**	77.66±1.42	58.93±4.50		23.84±1.90	5.75±0.29			
	**MCF-7**	81.13±4.99	53.83±1.04	48.58±2.70	39.33±5.59	11.65±1.45			
ADS10310	**MDA-MB-231**			93.83±5.27		82.12±1.69	70.83±0.13	62.82±2.15	42.04±2.94
	**MCF-7**			79.57±1.33		60.51±1.86	57.12±1.54	44.21±3.24	39.79±2.76

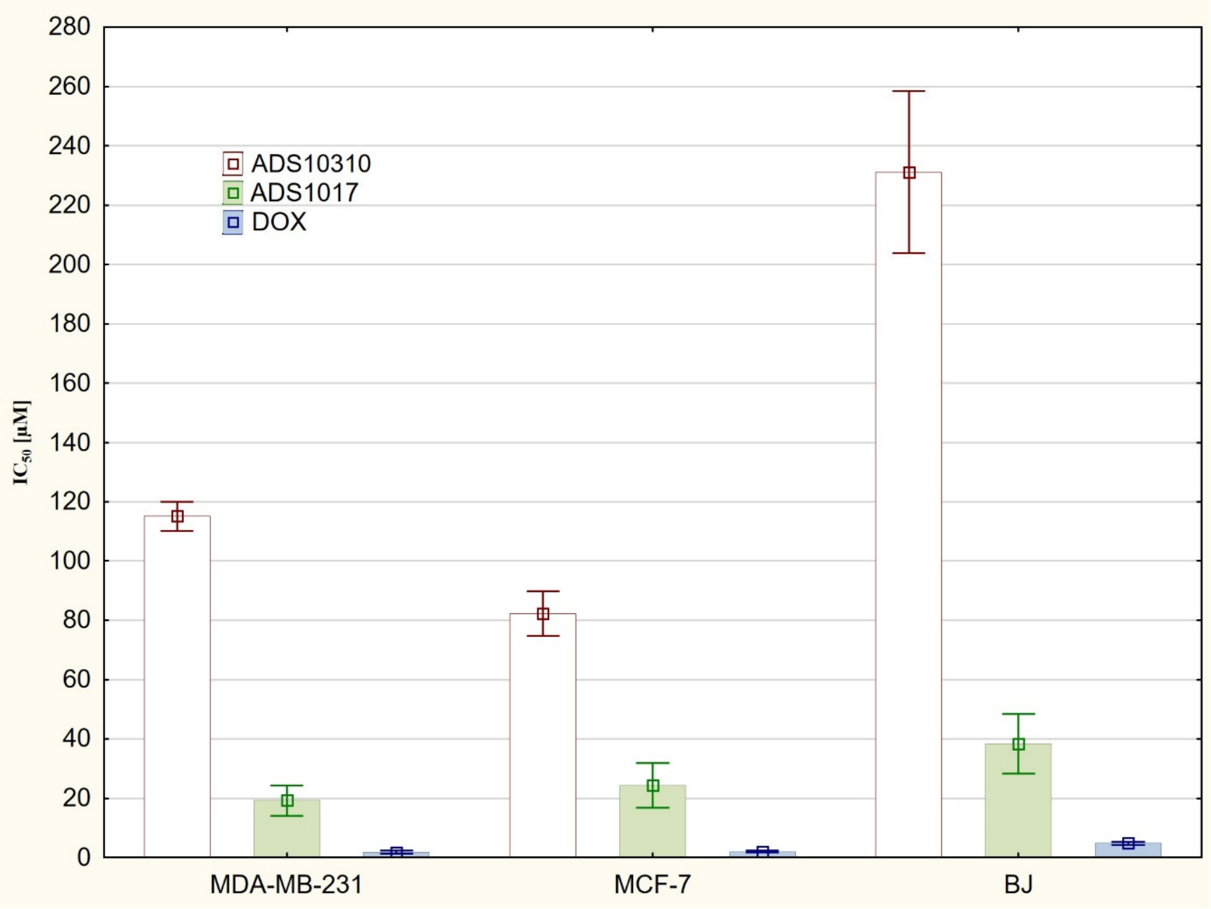

Figure S72. The cytotoxicity of ADS10310, ADS1017 and doxorubicin against MDA-MB-231, MCF7 and BJ cells.

3.5. Inhibition of electric eel AChE and equine serum BuChE

The target compounds were tested for their inhibitory potency against cholinesterases using Ellman's protocol, modified for 96-well microplates. [G.L. Ellman, K.D. Courtney, V. Andres, R.M. Featherstone, Biochem Pharmacol. 7 (1961) 88–95] All the reagents were purchased from Sigma–Aldrich (Steinheim, Germany). The enzymes were prepared as 5 U/mL aqueous stock solutions and diluted before use to a final concentration of 0.384 U/mL. Then 20 μL of prepared enzyme solutions (AChE or BuChE) were added to the reaction mixture in the wells, containing 25 μL of the target compound (or water in case of blank samples), 200 μL of 0.1 M phosphate buffer (pH=8.0) and 20 μL of 5,5'-dithiobis-(2-nitrobenzoic acid) (DTNB) (0.0025M). All those reagents were preincubated for 5 min at 25 °C. The enzymatic reaction was initiated by the addition of 20 μL of substrate acetylthiocholine iodide (ATC) (0.00375M) or butyrylthiocholine iodide (BTC) (0.00375M) solutions (depending on the enzyme used). After 5 min of incubation, changes in absorbance were measured at 412 nm, using EnSpire multimode microplate reader (PerkinElmer, Waltham, MA, USA). Target compounds were tested at a screening concentration of 10 μM. Percent of enzyme inhibition was calculated based on the formula 100-(S/B)x100, where S and B were the respective enzyme activities with and without the test compound, respectively. For the most potent compounds, with at least 50% of the enzyme inhibitory activity, IC_{50} values were determined. Calculations were based on the absorbance measured at six different concentrations of inhibitor, then converted to the % of enzyme inhibition, using the above presented formula. The obtained percentages of enzyme inhibition were plotted against the applied inhibitor concentrations, using nonlinear regression (GraphPad Prism 9; GraphPad Software, San Diego, CA, USA). Tacrine was tested as a reference compound. All the experiments were performed in triplicate.

Table S3. Inhibition of *electric eel* AChE and *equine serum* BuChE

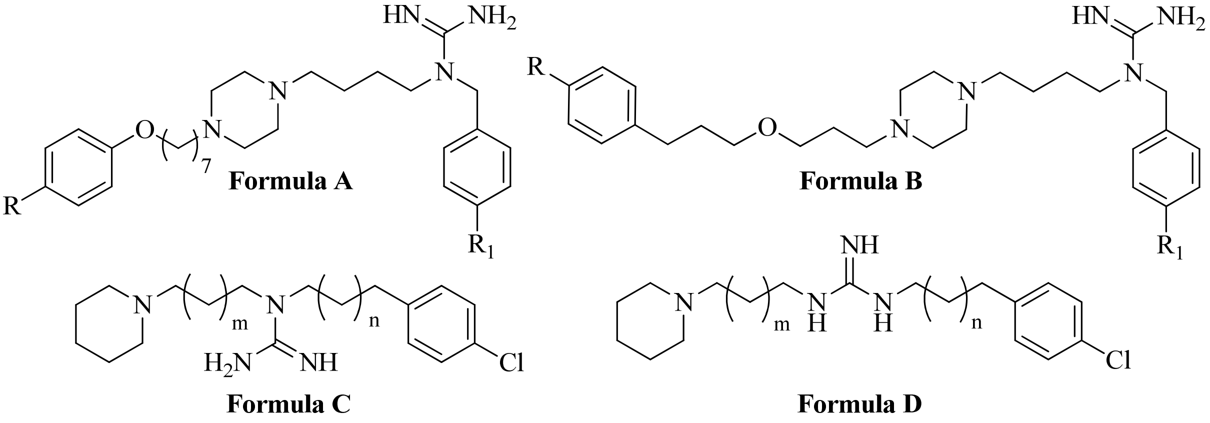

Cpd.	Formula/m/n/R/R_1	*ee*AChE % inh. (10µM) ± SD[a]	*ee*AChE IC_{50} (µM) ± sem[b]	*eq*BuChE % inh. (10 µM) ± SD[a]	*eq*BuChE IC_{50} (µM)) ± sem[c]
**ADS10377**	A/-/-/Cl/H	34.3 ± 2.6		84.6 ± 1.5	1.9 ± 0.1
**ADS10376**	A/-/-/Cl/CF_3	27.8 ± 5.1		71.4 ± 0.6	4.9 ± 0.2
**ADS1017**	A/-/-/H/H	49.7 ± 0.7		70.8±0.6	5.1 ± 0.1
**ADS10349**	B/-/-/H/H	6.4 ± 1.3		32.4 ± 4.2	
**ADS10350**	B/-/-/H/CF_3	1.0 ± 0.2		28.3 ± 3.2	
**ADS10278**	B/-/-/Cl/H	44.0 ± 0.9		76.6±1.3	2.0 ± 0.1
**ADS10279**	B/-/-/Cl/CF_3	47.4 ± 1.2		67.5±0.5	4.8 ± 0.1
**ADS10292**	C/1/1/-/-	50.6 ± 0.8	10.9 ± 0.4	54.3±4.7	8.4 ± 0.2
**ADS10300**	C/2/1/-/-	40.7 ± 1.8		75.2±0.4	2.4 ± 0.1
**ADS10312**	C/1/2/-/-	22.2 ± 3.9		72.6 ± 5.2	3.5 ± 0.1
**ADS10298**	D/1/1/-/-	39.8 ± 2.6		20.3±0.8	
**ADS10301**	D/2/1/-/-	11.3 ± 0.4		60.4 ± 0.7	5.9 ± 0.1
**ADS10306**	D/1/2/-/-	17.8 ± 0.7		89.4 ± 0.1	1.6 ± 0.0
**ADS10310**	D/2/2/-/-	7.3 ± 0.9		83.9 ± 0.1	1.7 ± 0.1
**Tacrine**			0.024 ± 0.001		0.015 ± 0.001

[a] mean value ± standard deviation (SD) of three independent experiments; [b] IC_{50} inhibitory concentration of *electric eel* AChE; mean value ± standard error of the mean (sem) of triplicate independent experiments; [c] IC_{50} inhibitory concentration of BuChE from *equine serum*; mean value ± standard error of the mean (sem) of triplicate independent experiments.

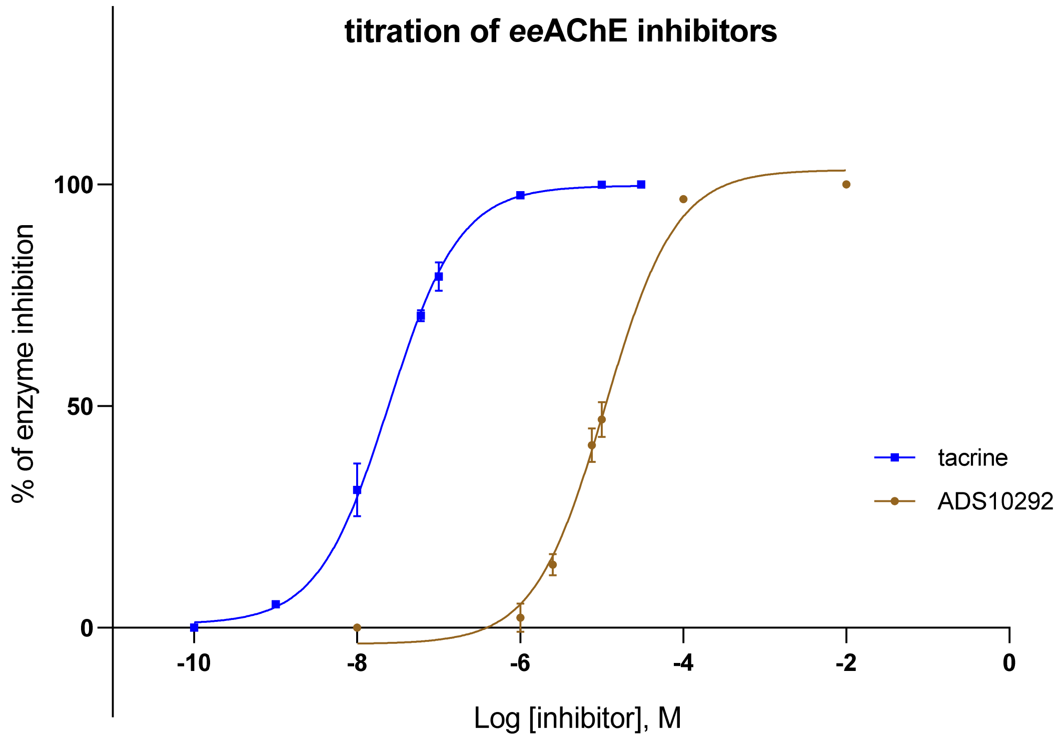

Figure S73. Inhibition of *electric eel* AChE by ADS10310 and tacrine.

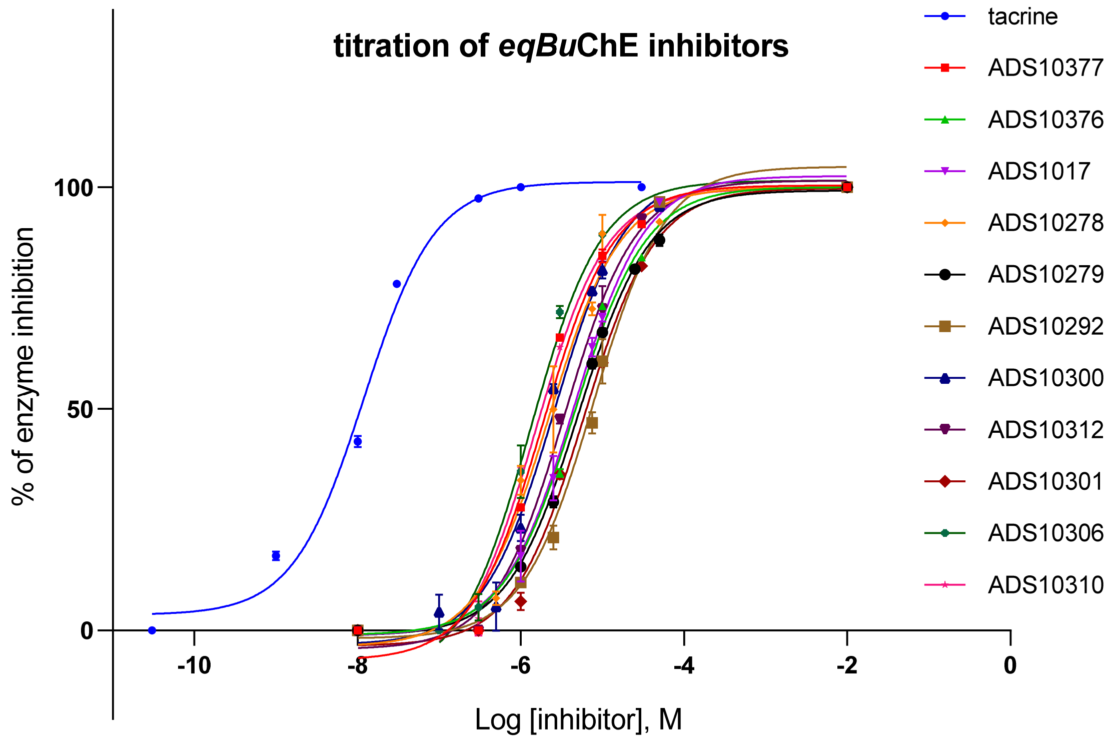

Figure S74. Inhibition of *electric eel* BuChE by compound ADS compounds and tacrine.

4. *In vitro* metabolic stability.

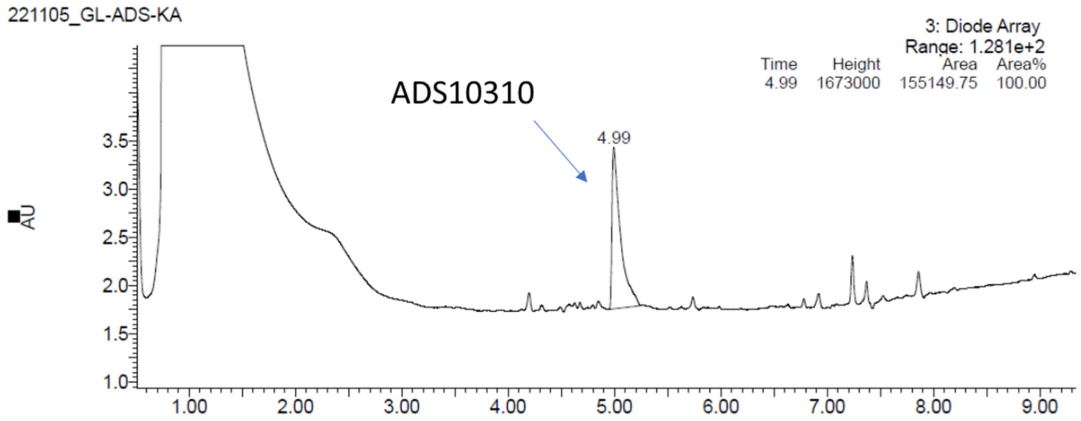

Figure S75. UPLC spectra after 120 min incubation of compound ADS10310 in TRIS buffer pH=7.4 at 37ºC without human liver microsomes (control reaction).

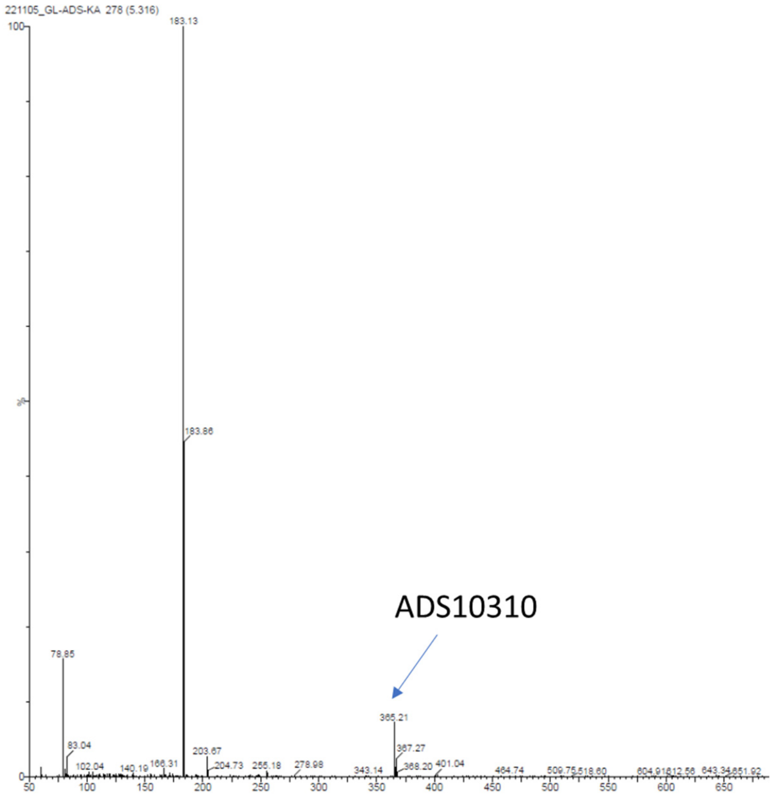

Figure S76. MS analysis of ADS10310 from control reaction.

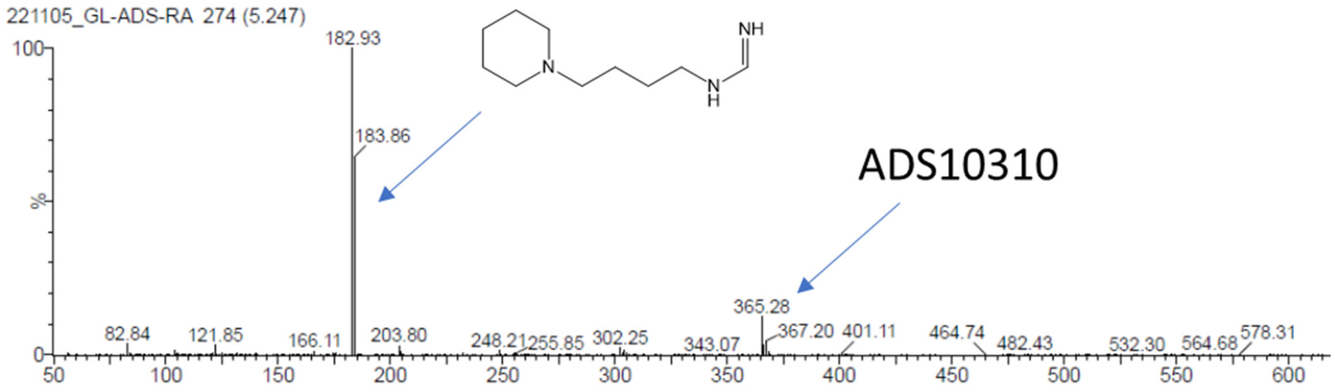

Figure S77. MS spectra of ADS10310 and the most probable structure of its fragment with mass m/z = 182.93 based on analysis conditions.

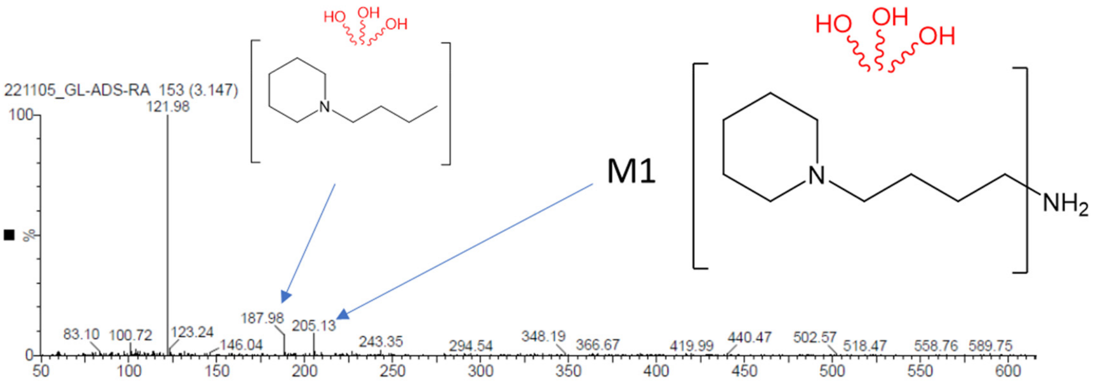

Figure S78. MS spectra of ADS10310 and the most probable structure of metabolite M1 with molecular mass m/z = 205.13. The structure of the fragmented under analysis conditions M1 with mass m/z = 187.98 was also proposed.

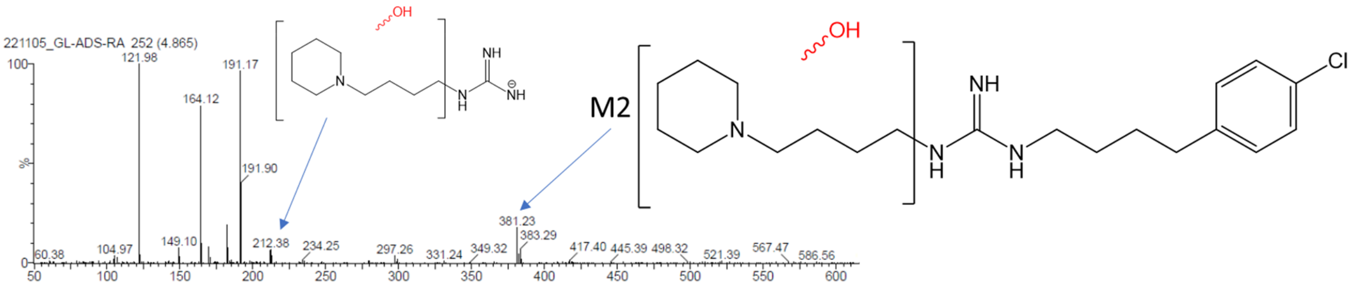

Figure S79. MS spectra of ADS10310 and the most probable structure of metabolite M2 with molecular mass m/z = 381.23. The structure of the fragmented under analysis conditions M2 with mass m/z = 212.38 was also proposed. The most probable site of hydroxylation was marked in brackets.

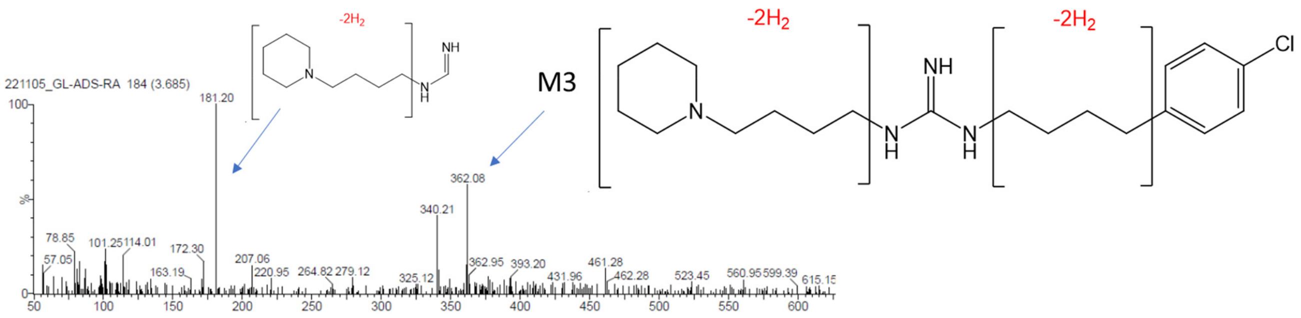

Figure S80. MS spectra of ADS10310 and the most probable structure of metabolite M3 with molecular mass m/z = 362.08. The structure of the fragmented under analysis conditions M3 with mass m/z = 181.20 was also proposed. The most probable site of dehydrogenations was marked in brackets.

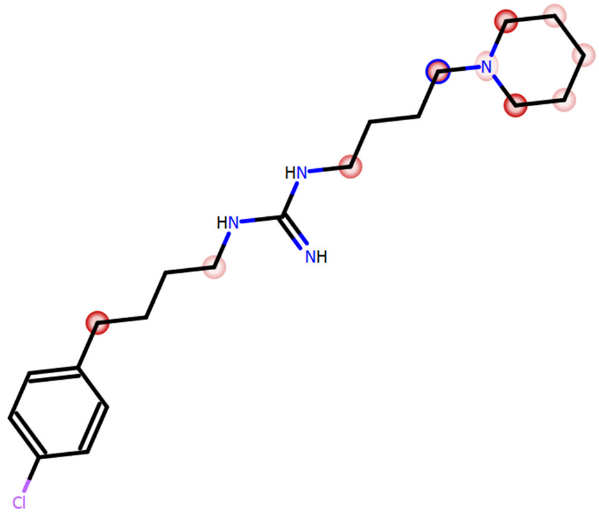

Figure S81. The MetaSite 6.0.1. software prediction of the most probable sites of ADS10310 metabolism. The darker red color - the higher probability to be involved in the metabolism pathway. The blue circle marked the site of compound with the highest probability of metabolic bioconversion.

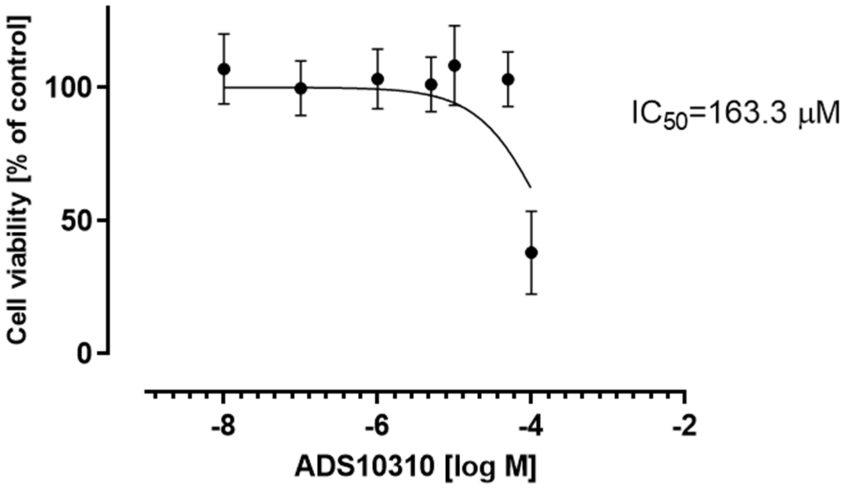

Figure S82. The cytotoxicity of ADS10310 against HepG2 cells.
